# Allogeneic Mesenchymal Stem Cells Ameliorate Aging Frailty: A Phase II Randomized, Double-Blind, Placebo-Controlled Clinical Trial

**DOI:** 10.1093/gerona/glx137

**Published:** 2017-07-17

**Authors:** Bryon A Tompkins, Darcy L DiFede, Aisha Khan, Ana Marie Landin, Ivonne Hernandez Schulman, Marietsy V Pujol, Alan W Heldman, Roberto Miki, Pascal J Goldschmidt-Clermont, Bradley J Goldstein, Muzammil Mushtaq, Silvina Levis-Dusseau, John J Byrnes, Maureen Lowery, Makoto Natsumeda, Cindy Delgado, Russell Saltzman, Mayra Vidro-Casiano, Moisaniel Da Fonseca, Samuel Golpanian, Courtney Premer, Audrey Medina, Krystalenia Valasaki, Victoria Florea, Erica Anderson, Jill El-Khorazaty, Adam Mendizabal, Geoff Green, Anthony A Oliva, Joshua M Hare

**Affiliations:** 1 The Interdisciplinary Stem Cell Institute,; 2 Department of Surgery, and; 3 Department of Medicine, University of Miami Miller School of Medicine, Florida.; 4 EMMES Corporation, Rockville, Maryland.; 5 Longeveron LLC, Miami, Florida.

**Keywords:** Immunomodulation, Tumor necrosis factor-α, Regenerative medicine

## Abstract

**Background:**

Aging frailty, characterized by decreased physical and immunological functioning, is associated with stem cell depletion. Human allogeneic mesenchymal stem cells (allo-hMSCs) exert immunomodulatory effects and promote tissue repair.

**Methods:**

This is a randomized, double-blinded, dose-finding study of intravenous allo-hMSCs (100 or 200-million [M]) vs placebo delivered to patients (*n* = 30, mean age 75.5 ± 7.3) with frailty. The primary endpoint was incidence of treatment-emergent serious adverse events (TE-SAEs) at 1-month postinfusion. Secondary endpoints included physical performance, patient-reported outcomes, and immune markers of frailty measured at 6 months postinfusion.

**Results:**

No therapy-related TE-SAEs occurred at 1 month. Physical performance improved preferentially in the 100M-group; immunologic improvement occurred in both the 100M- and 200M-groups. The 6-minute walk test, short physical performance exam, and forced expiratory volume in 1 second improved in the 100M-group (*p* = .01), not in the 200M- or placebo groups. The female sexual quality of life questionnaire improved in the 100M-group (*p* = .03). Serum TNF-α levels decreased in the 100M-group (*p* = .03). B cell intracellular TNF-α improved in both the 100M- (*p* < .0001) and 200M-groups (*p* = .002) as well as between groups compared to placebo (*p* = .003 and *p* = .039, respectively). Early and late activated T-cells were also reduced by MSC therapy.

**Conclusion:**

Intravenous allo-hMSCs were safe in individuals with aging frailty. Treated groups had remarkable improvements in physical performance measures and inflammatory biomarkers, both of which characterize the frailty syndrome. Given the excellent safety and efficacy profiles demonstrated in this study, larger clinical trials are warranted to establish the efficacy of hMSCs in this multisystem disorder.

**Clinical Trial Registration:**

www.clinicaltrials.gov: CRATUS (#NCT02065245).

There is increasing recognition of the health burden of frailty, a syndrome that increases in incidence with aging. Frailty confers an increased vulnerability to adverse health outcomes and mortality in response to stressors (1,2). Of note, the frailty syndrome is driven mostly by biological aging processes that include inflammation and stem cell dysfunction, as opposed to chronological aging (2–5). Early intervention may improve quality of life, reduce hospitalizations, and nursing home costs (6,7). Therefore, it is increasingly important to recognize the clinical onset of frailty, and to develop effective therapeutic strategies.

There are two main models used to define frailty (7): The deficit and the physical phenotype model. The deficit model accounts for a person’s geriatric syndromes, diseases, psychosocial, physical, and cognitive impairments, and combines them to create a “Frailty Index” (8). The physical phenotype model consists of the identification of at least three factors: weight loss, exhaustion, weakness, slowness, and decreased physical activity, which together comprise an underlying state of multisystem dysregulation (9,10). Despite the use of different criteria for evaluating frailty, both models show evidence that the prevalence of the syndrome increases with age and is higher among women (9.6%) than men (5.2%) (11). In a study of over 44,000 community-dwelling elderly adults, the overall prevalence of frailty was found to be 10.7% (11).

Currently, several multimodal interventions are employed to manage frailty, namely resistance/aerobic exercise, caloric support, vitamin D, and optimization of polypharmacy (7). However, there are no specific medical or biologic treatments that ameliorate or reverse frailty (12,13). Stem cell depletion is a key mechanism postulated to contribute to frailty (14–16). In this regard, we recently conducted a phase I open label study of human allogeneic mesenchymal stem cells (allo-hMSCs) intravenously infused for frailty, which showed that the cells could be safely administered, improved measures of functional capacity, and reduced inflammation (17). Therefore, we conducted the current phase II double-blinded and placebo-controlled study in order to test the hypothesis that exogenous allo-hMSCs could reverse signs and symptoms of frailty in older individuals. Similar approaches have been shown to exert beneficial effects on the cardiovascular system, with functional improvements on various types of heart disease (18–20), endothelial function (21), and systemic inflammation (22). Given their pleiotropic mechanisms of action, which include antifibrotic, anti-inflammatory, proangiogenic properties (23), and their ability to stimulate endogenous progenitor cells (21,24), we hypothesize that their use may offer a novel treatment strategy in frail patients.

## Methods

The Allogenei*C* Human Mesenchymal Stem Cells in Patients with Aging F*RA*il*T*y via Intraveno*US* Delivery (CRATUS) study (#NCT02065245) is a phase II, randomized, double-blinded, placebo-controlled study of allo-hMSCs delivered intravenously (IV) in frail individuals to test the safety and efficacy of allo-hMSCs in reducing markers of inflammation and improving markers of physical and mental functioning and quality of life (15,25).

### Study Design, Stem Cell Procurement and Randomization

**Figure 1.**
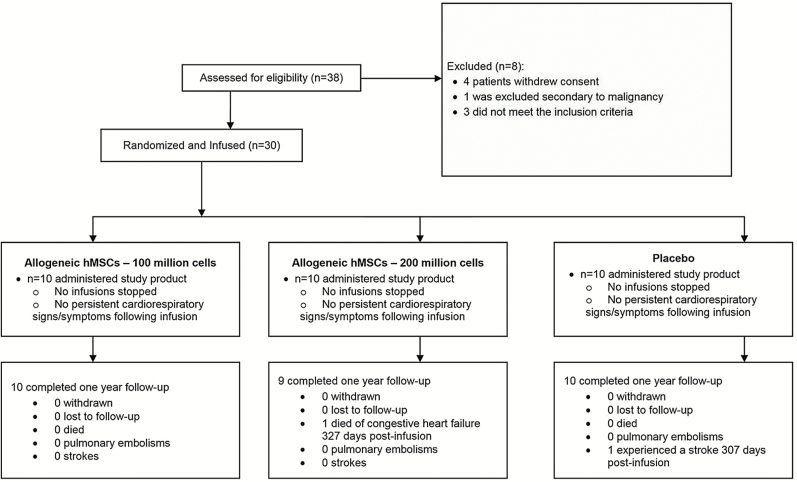
Study flow chart. Patient screening, follow-up, and randomization in a 1:1:1 fashion to either the 100M-group, 200M-group, or placebo. M = Million.

The study design and phase I of the CRATUS study have been recently published (17,26). Screening and patient randomization are outlined in Figure 1 (26), and available in the Supplementary Material.

### Patient Inclusion Criteria and Timeline

The inclusion criteria were as follows: (i) Patients were provided written informed consent, (ii) patients were aged ≥60 and ≤95 years at the time of signing the Informed Consent Form, and (iii) they showed the signs of frailty based on physician assessment, apart from a concomitant condition, by a score between 4 and 7 as denoted by the Canadian Study on Health Aging (25,27,28). Major exclusion criteria and a detailed timeline have been published (26).

### Study Endpoints

The primary endpoint was the safety of allo-hMSCs at 1 month, assessed by treatment emergent-serious adverse events (TE-SAE). TE-SAEs were defined by the following: death, nonfatal pulmonary embolism, stroke, hospitalization for worsening dyspnea, and clinically significant laboratory abnormalities.

The secondary endpoints assessed the efficacy of the therapy. Efficacy was demonstrated by differences in the rate of change of frailty markers as defined by: reduced activity (Community Healthy Activities Model Program for Seniors (CHAMPS) questionnaire), slowing of mobility (6-minute walk test (6MWT), 4-m gait speed test (4MGST), and the short physical performance battery (SPPB) score, comprised of balance tests, gait speed tests, and chair stand tests), weight loss, diminished hand grip strength (dynamometry), exhaustion-multidimensional fatigue inventory (MFI), quality of life assessments (Sexual Quality of Life-Female (SQOL-F) and International Index of Erectile Dysfunction (IIEF) Questionnaires), dobutamine-induced ejection fraction (EF) via echocardiography, C-reactive protein (CRP), IL-6, D-dimer, complete blood cell count (CBC) with differential, and TNF-α.

### Immune Monitoring

Immune biomarkers were measured at baseline and 6 months as described previously (17) and in the Supplementary Material.

### Statistical Analysis

No formal statistical justification was performed to determine sample size for this study. Sample size was determined to be appropriate for an early phase study to assess safety in this population. Due to the early phase nature of this study, no adjustments were made for multiple analyses (26). Statistical analysis was completed by statisticians at the Emmes Corporation and is available in the Supplementary Material.

## Results

### Patient Population

Table 1 shows the baseline characteristics of the enrolled patients. Sixty percent of the patients were White males and the mean age was 75.5 ± 7.3 years.

**Table 1.** Baseline Characteristics

	Treatment Group			Total (*N* = 30) *N* (%)
Characteristics	Allo-100M (*N* = 10) *N* (%)	Allo-200M (*N* = 10) *N* (%)	Placebo (*N* = 10) *N* (%)	
Gender				
Male	6 (60%)	6 (60%)	6 (60%)	18 (60%)
Female	4 (40%)	4 (40%)	4 (40%)	12 (40%)
Ethnicity				
Hispanic or Latino	1 (10%)	1 (10%)	2 (20%)	4 (13%)
Not Hispanic or Latino	9 (90%)	9 (90%)	8 (80%)	26 (87%)
Race				
American Indian/Alaskan Native	0 (0%)	1 (10%)	0 (0%)	1 (3%)
White American	10 (100%)	9 (90%)	10 (100%)	29 (97%)
Age at infusion (years)	75.0 ± 7.4	76.3 ± 8.4	75.3 ± 6.8	75.5 ± 7.3
Infusion status				
Yes	10 (100%)	10 (100%)	10 (100%)	30 (100%)
No	0 (0%)	0 (0%)	0 (0%)	0 (0%)
Unknown	0 (0%)	0 (0%)	0 (0%)	0 (0%)
Canadian Clinical Frailty Score				
4	5 (50%)	7 (70%)	5 (50%)	17 (57%)
5	3 (30%)	1 (10%)	5 (50%)	9 (30%)
6	2 (20%)	2 (20%)	0 (0%)	4 (13%)
7	0 (0%)	0 (0%)	0 (0%)	0 (0%)
Mini-mental state examination	29.3 ± 0.8	28.5 ± 1.1	29.5 ± 1.0	29.1 ± 1.0
Hemoglobin level (g/dL)	14.1 ± 1.2	13.5 ± 1.3	14.3 ± 1.2	14.0 ± 1.3
WBC count (cells/mm^3^)	7,160 ± 2,438	6,600 ± 1,304	7,070 ± 2,215	6,943 ± 1,989
Platelet count (cells/mm^3^)	207,000 ± 64,389	194,500 ± 37,936	194,500 ± 57,880	198,667 ± 52,999
AST (U/L)	24.5 ± 7.6	20.7 ± 3.6	29.3 ± 11.1	24.8 ± 8.5
ALT (U/L)	23.0 ± 16.2	16.5 ± 6.0	31.9 ± 15.6	23.8 ± 14.5
Six-min walk test (m)	345.9 ± 103.4	390.6 ± 148.9	385.8 ± 83.1	374.1 ± 112.9
FEV1 (L)	2.5 ± 0.7	2.3 ± 0.9	2.3 ± 0.5	2.4 ± 0.7
FEV1 (percent predicted)	90.6 ± 10.4	86.9 ± 25.4	87.9 ± 15.2	88.5 ± 17.6
Tumor necrosis factor-α (pg/mL)	3.2 (2.8, 3.8)	3.2 (2.6, 3.4)	2.4 (1.1, 3.1)	3.1 (2.1, 3.4)

*Note*: Values are mean ± *SD*, *N* (%), or median (interquartile range [IQR]). FEV1 (Liters) = Forced Expiratory Volume in one second. Hemoglobin (grams/deciliter). WBC (cells/millimeters = White blood cells. AST (U/L) = Aspartate Aminotransferase (units/liter). ALT = Alanine Aminotransferase. Six-min walk test distance (m, meters). Tumor necrosis factor-α (pg/mL, picogram/milliliter).

### Safety

No TE-SAEs occurred in any of the three groups in the first 30 days. Similarly, there were no cumulative treatment-related SAEs in either group throughout the duration of the study (Table 2). None of the patients showed any signs of adverse cardiopulmonary reaction following the intravenous infusion. There were no clinically significant changes in basic hematologic and chemistry laboratory tests throughout the duration of the study.

**Table 2.** Safety Summary

System Organ Class	MedDRA Preferred Term	Treatment Group							
		Allo-100M (*N* = 10)		Allo-200M (*N* = 10)		Placebo (*N* = 10)		Total (*N* = 30)	
		Events	Patients	Events	Patients	Events	Patients	Events	Patients
		*N* (%)	*N* (%)	*N* (%)	*N* (%)	*N* (%)	*N* (%)	*N* (%)	*N* (%)
General disorders and administration site conditions	Death			1 (50%)	1 (10%)			1 (10%)	1 (3%)
Hepatobiliary disorders	Cholecystitis					1 (17%)	1 (10%)	1 (10%)	1 (3%)
Infections and infestations	Gastroenteritis					1 (17%)	1 (10%)	1 (10%)	1 (3%)
Musculoskeletal and connective tissue disorders	Flank pain	1 (50%)	1 (10%)					1 (10%)	1 (3%)
	Spinal column stenosis					1 (17%)	1 (10%)	1 (10%)	1 (3%)
	Spondylolisthesis			1 (50%)	1 (10%)			1 (10%)	1 (3%)
Neoplasms benign, malignant and unspecified (incl cysts and polyps)	Glioblastoma					1 (17%)	1 (10%)	1 (10%)	1 (3%)
Renal and urinary disorders	Ureteric stenosis	1 (50%)	1 (10%)					1 (10%)	1 (3%)
Vascular disorders	Aneurysm					1 (16%)	1 (10%)	1 (10%)	1 (3%)
	Hypotension					1 (17%)	1 (10%)	1 (10%)	1 (3%)
Total		2 (100%)	1 (10%)	2 (100%)	2 (20%)	6 (100%)	4 (40%)	10 (100%)	7 (23%)

*Note*: There were no TE-SAEs at 1 month or SAEs in either group. One patient died in the 200M-group unrelated to the treatment. There were seven hospitalizations, two in the 100M-group, and five in the placebo. None were related to the procedure. Details of the hospitalizations are located in the supplementary material. SAE = Serious adverse events; TE-SAE = Treatment-emergent serious adverse events.

### Long-term Adverse Events

One patient in the 200M-group died of an unrelated event prior to the 12-month follow-up. Additionally, one patient in the placebo had an unrelated stroke 307 days postinfusion. The proportion of patients with adverse events at 12 months did not differ between groups at the 6- and 12-month time points (*p* = .300 and *p* = .141, respectively).

### Hospitalization

There were four patients who required hospitalization within the 12-month follow-up. Two of the hospitalizations were reported in one patient in the 100M-group, both of which were moderate in severity; however, none of the hospitalizations were secondary to the procedure. No patients in the 200M-group were hospitalized. The remaining three patients belonged to the placebo group; one patient had two moderate hospitalizations and one severe, another had a hospitalization that was moderate in severity, and another had one severe hospitalization. None of the hospitalizations were related to the procedure.

### Functional Status, Quality of Life, and Pulmonary Function

**Figure 2.**
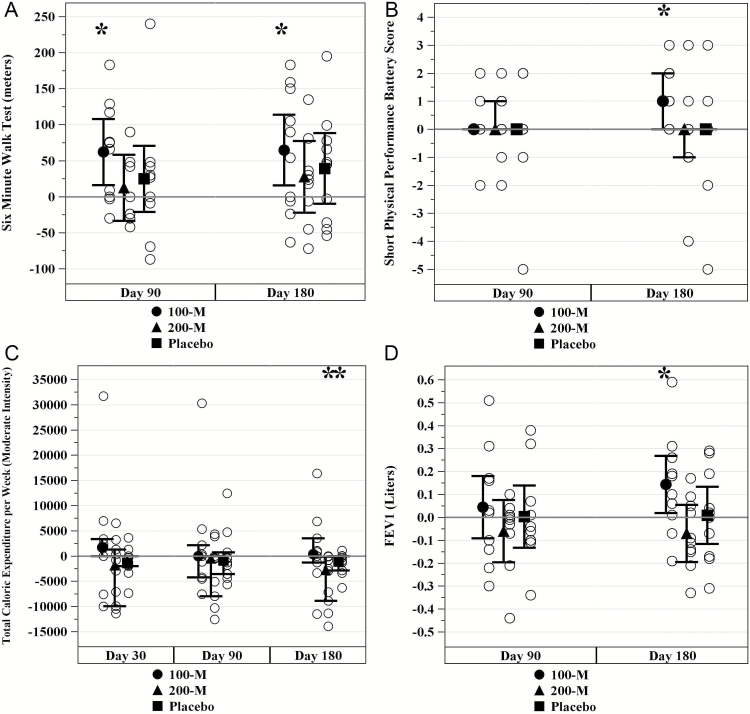
Physical markers of frailty. (**A**) Six-minute walk test (6MWT) increased in mean meters walked in the 100M-group from baseline to 6 months (*p* = .011) but not the 200M-group (*p* = .263) or placebo (*p* = .112). (**B**) Short physical performance battery (SPPB) was significant for an overall improvement in the median total score in the 100M-group from baseline to 6 months (*p* = .031) but not in the 200M-group (*p* = .812) or placebo (*p* = .875). (**C**) Community Healthy Activities Model Program for Seniors (CHAMPS) questionnaire was significant for a reduced median total caloric expenditure per week at moderate intensity from baseline to 6 months in the 200M-group (*p* = .008) and placebo (*p* = .039), but not in the 100M-group (*p* = .641). (**D**) Forced expiratory volume after 1 second (FEV1) improved in mean liters from baseline to 6 months in the 100M-group (*p* = .025) without changes noted in the 200M-group (*p* = .259) or placebo (*p* = .883). * indicates *p* ≤ .05.

Quality of life and functional status were monitored throughout the study. These outcomes preferentially improved in patients randomized to receive 100M allo-hMSCs. The 6MWT increased in the 100M-group from baseline to 6 months (345.9 ± 103.4 to 410.7 ± 155.4 m, *p* = .011; Figure 2A). There was no significant change at 6 months (*p* = .263) in either the 200M-group or placebo (*p* = .112). The 4MGST showed no significant differences among groups (*p* = .659) at 6 months. Consistent with the improvement in 6MWT, the SPPB total score was significantly improved in the 100M-group from baseline to 6 months (median 10.5, IQR 9.0, 12.0 to 12.0, IQR 11.0, 12.0; *p* = .031; Figure 2B). However, there were no significant differences in the 200M-group (*p* = .812) or placebo (*p* = .875). The CHAMPS-questionnaire showed a reduced total caloric expenditure per week at moderate intensity in the 200M-group from baseline to 6 months (median 5,118.8, IQR 1,470.0, 1,4542 to 1,509.4, IQR 472.5, 6,090.0; *p* = .008) and placebo (median 3,386.3, IQR 1,286.3, 4,042.0 to 2,021.3, IQR 682.5, 3,150.0; *p* = .039; Figure 2C). Conversely, there was no significant reduction in the 100M-group at 6 months (*p* = .641). There were no differences between groups in weight loss (*p* = .7599), MFI, which assessed mental fatigue (*p* = .548), and handgrip strength as assessed via the average of dominant hand scores (*p* = .676). Ejection fraction, assessed by dobutamine stress echo, remained stable throughout the study in all patients. FEV1 improved in the 100M-group from baseline to 6 months (2.5 ± 0.66 to 2.6 ± 0.77 L/min, *p* = .025) without significant changes in the 200M-group (*p* = .259) or placebo (*p* = .883; Figure 2D).

### Immune Biomarkers

**Figure 3.**
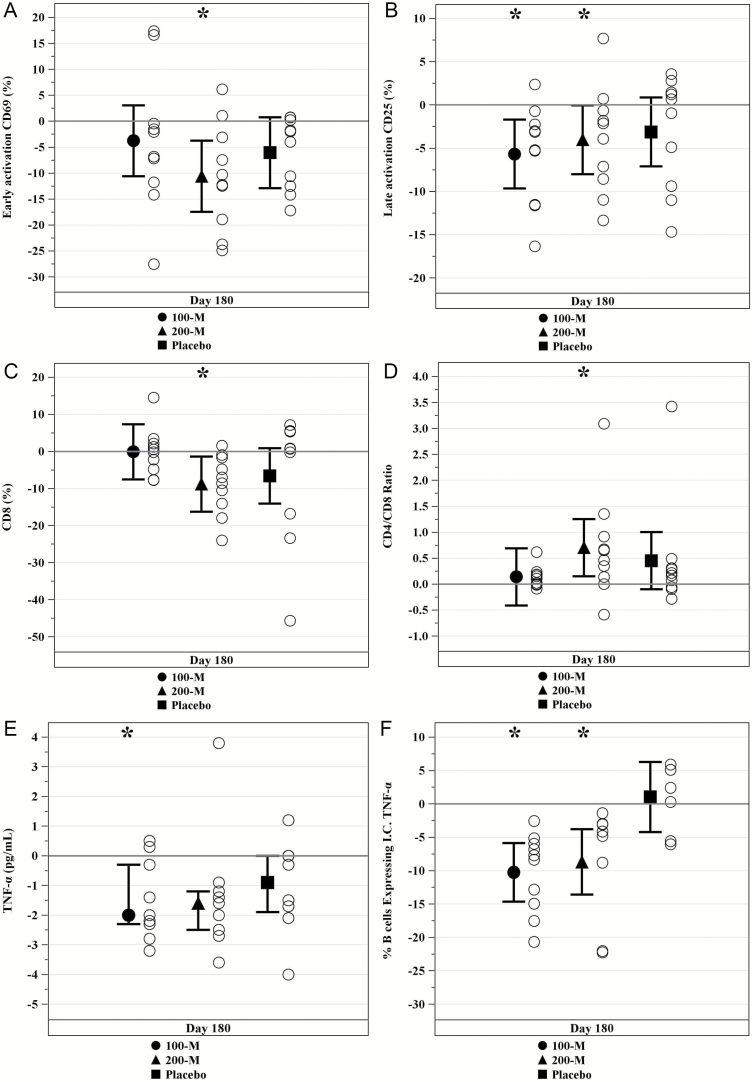
Immune biomarkers in frailty. All time points are from baseline to 6 months except for TNF-α which begins on Day 1 (infusion) through 6 months. (**A**) Early T-cell activation (CD3, CD69) were reduced as a percent change from baseline to 6 months in the 200M-group (*p* = .004), but not the 100M-group (*p* = .269) or placebo (*p* = .0797). (**B**) Late T-cell activation (CD3, CD25) was reduced as a percent change from baseline to 6 months in the 100M and 200M-groups (*p* = .007 and *p* = .048 respectively), but not in the placebo (*p* = .119). (**C**) % CD8 T-cells decreased from baseline to 6 months in the 200M-group (*p* = .022) and no changes were noted in the 100M-group (*p* = .978) or placebo (*p* = .0797). (**D**) CD4/CD8 ratio increased from baseline to 6 months in the 200M-group (*p* = .014) and no changes were found in the 100M-group (*p* = .609) or placebo (*p* = .104). (**E**) Serum TNF-α decreased in pg/mL from baseline to 6 months in the 100M-group (*p* = .031) without a change in the 200M-group (*p* = .129) or placebo (*p* = .094). (**F**) %B cells expressing intracellular TNF-α decreased from baseline to 6 months in the 100M (*p* < .0001) and 200M-groups (*p* = .002) without a significant change in placebo (*p* = .869). * indicates *p* ≤ .05.

Immunotolerability was assessed using a calculated PRA (cPRA) measured at baseline and 6 months postinfusion on each patient. Three patients had a mild/moderate increase in donor specific antibodies (one mild in the 100M- and two moderate in the 200M-group). There were two other patients in the 200M-group that had a mild/moderate increase in cPRA but were not donor-specific reactions (Table 3). There were no clinically significant immune reactions reported. Both the 100M and 200M doses were effective in modulating immune parameters whereas placebo was not. Reduction in the early-activation CD69 cells was noted in the 200M-group (27.0 ± 4.30 to 16.4 ± 7.25%, *p* = .004; Figure 3A) at 6 months. There were no reductions in the 100M-group (*p* = .269) or placebo (*p* = .0797; Figure 3A). There was a reduction in the late-activation CD25 cells in both the 100M-group (12.6 ± 6.87 to 6.9 ± 3.30%, *p* = .007) and 200M-groups (12.0 ± 6.68 to 8.0 ± 4.64%, *p* = .048) from baseline to 6 months. No significant reduction was noted in the placebo (*p* = .119; Figure 3B). The CD8 T-cell marker decreased significantly in the 200M-group from baseline to 6 months (28.7 ± 15.04 to 19.9 ± 10.03%, *p* = .022; Figure 3C). This is a crucial finding as aging is marked by an expansion of CD8 cells (29). There were no significant changes in the 100M-group (*p* = .978) or placebo at 6 months (*p* = .0797; Figure 3C). There were no significant changes in CD4 cells in the 200M-group (*p* = .052), 100M-group (*p* = .135), or placebo (*p* = .540). The CD4/CD8 ratio appropriately increased in the 200M-group at 6 months (1.2 ± 1.05 to 1.9 ± 1.17, *p* = .014), however there were no changes in the 100M-group (*p* = .609) or placebo (*p* = .104; Figure 3D).

**Table 3.** Calculated Panel Reactive Antibodies (cPRA)

cPRA	Treatment Group		
% Increase in donor specific cPRA (Baseline to 6 mo)	Allo-100M (*N* = 10)	Allo-200M (*N* = 10)	Placebo (*N* = 10)
Negative (0–10%)	9	8	10
Mild (11–20%)	1	0	0
Moderate (21–79%)	0	2	0
High (≥80%)	0	0	0

*Note*: cPRAs are from baseline to 6 months, and showed that nine out of ten patients in the 100M-group had no reaction and 1 had a mild cPRA of 19% that was donor specific for class I. Eight out of 10 patients in the 200M-group had no reaction and 2 had a moderate reaction (one patient developed a 29% cPRA which was donor specific for 1 class II, and another patient developed 36% cPRA which was not donor specific and all were class I). There were no panel reactive antibodies in the 10 patients in the placebo. Values are the number of patients in each cPRA category (negative, mild, moderate, and high).

Serum TNF-α decreased in the 100M-group at 6 months (median 3.2, IQR 2.8, 3.8 to 1.2, IQR 1.0, 2.8, *p* = .031), whereas it did not significantly change in the 200M-group (*p* = .129) or placebo (*p* = .094; Figure 3E). Similarly, B cell intracellular TNF-α significantly decreased in both the 100M- and 200M-groups (17.3 ± 1.8 to 7.0 ± 1.0, *p* < .0001, and 17.1 ± 2.0 to 8.4 ± 1.1, *p* = .001, respectively; Figure 3F) with no improvement in placebo at 6 months (*p* = .69). The reductions in both the 100M- and 200M-groups were significant compared to placebo (*p* < .00001 and *p* = .00002; Figure 3F). Finally, there were no significant changes noted in IL-6, CRP, D-dimer, CBC, or fibrinogen at 6 months in any group (data not shown).

### Sexual Quality of Life

**Figure 4.**
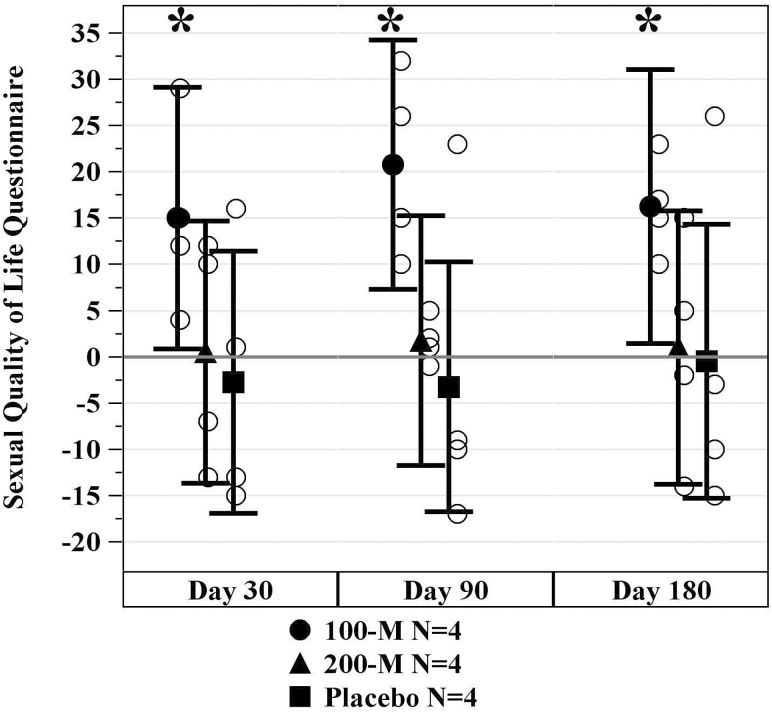
Sexual quality of life-female (SQOL-F) questionnaire. There was a mean increase in the 100M-group (*p* = .0348) from baseline to 6 months as compared to the 200M-group (*p* = .882) and placebo (*p* = .941). * indicates *p* ≤ .05.

Among female patients, the SQOL-F exhibited a remarkable increase in the 100M-group at 6 months (59.8 ± 15.3 to 76.0 ± 12.9, *p* = .035), but no changes were observed in the 200M-group (*p* = .882) or placebo (*p* = .941; Figure 4). Conversely, there were no differences among male participants in the IIEF from baseline to 6 months (*p* = .666).

## Discussion

The CRATUS trial is a randomized, double-blind, placebo-controlled evaluation of allo-hMCSs to treat the signs and symptoms of frailty. The results support the safety and feasibility of administering allo-hMSCs in this population. With regard to efficacy, there was a preferential effect towards improvement of functional capacity and patient reported outcome measures in patients receiving lower dose MSCs, although immunologic bioactivity was evident with both doses. Together, these findings suggest that allo-hMSCs may be an effective biological modifier of aging frailty, and support ongoing investigation of allo-hMSCs alone or as an adjunct to current physical training strategies for aging frailty.

These findings are in agreement with a recently completed dose-finding phase I safety study (17). In that study, two important sets of observations were made. First, a constellation of physical performance measures improved with cell therapy, and second, 100M cells represented the peak responsiveness dose, with a plateau and/or reduction in efficacy being noted with 200M cells. Accordingly, this study was based, in part, on phase I. Importantly, randomizing patients in a double-blind fashion to 100M cells, 200M cells, or placebo was performed to validate the results of the earlier study and to confirm both the constellation of physical performance findings and the dose–response.

The findings here replicate in large part the results of the earlier open label study, support the concept that MSCs have bioactivity against aging frailty, and confirm the fact that 100M represents a superior dose level compared to 200M. The reasons underlying the inverse dose relationship noted here remain incompletely understood. The 100M dose group produced significant improvements in both physiologic and immunologic markers of frailty, while the high dose group solely demonstrated positive immunomodulatory effects. It is important to note that there is a precedent for this in earlier studies, and a number of stem cell-based clinical trials exhibit greater effects with lower doses (19,30). However, the available preclinical and clinical evidence regarding dose relationship in stem cell therapy is conflicting (31), with some studies reporting that lower cell dosage and/or infusion cell concentration may provide the most benefit (19,32), while others finding either a direct or nonlinear relationship (33).

There are several factors that could contribute to nonlinear dose response curves with cell-based therapy. These include variation in functional activity of the cells rather than the absolute number of cells infused. In this regard, higher cell concentrations could impair cell activity through physical effects such as concentration-dependent cell aggregation, or damage of cells due to excessive shear forces on cells during infusion that could influence the relationship between cell dose and clinical benefit (31). Therefore, studies have recently been focusing on cell activity and or genetic modification to enhance their activity, rather than quantity (34,35). However, as with all progenitor cell types in various disease processes, whether modified or not, exact dosing has yet to be established, and thus is a weakness of this particular study. Given the novel use of MSCs in frailty, a patient population for whom a successful therapy has yet to be developed, dosing was based on safety as established by previous studies (18,19) and phase I (17), and was further investigated in the current study. Importantly, safety was ultimately established in both cell-dose groups. The optimal effective dosing will be investigated in future larger randomized trials.

In the current study, we employed allo-hMSCs, which can target two pathways implicated in the pathogenesis of aging frailty—inflammation and stem cell depletion. The current findings support the idea that biological modification of aging frailty is not only feasible but has the potential to meaningfully impact the physical performance of older individuals with mild to moderate. It is noteworthy that the effects on inflammatory cytokines may have practical value by providing a clinical useful biomarker. Importantly, both circulating TNF-α levels as well as B-cell intracellular TNF-α appear to be candidate biomakers that could be used to index the efficacy of hMSCs in this patient population.

The 6MWT, SPPB-questionnaire, FEV1, and the CHAMPS-questionnaire were among the physical performance measures examined in our study, and the 100M-group in particular produced a meaningful outcome in all measures. The 6MWT was originally designed to evaluate cardiac and pulmonary disorders; recently, its application has expanded to assess an individual’s exercise capacity at various levels of intensity and their ability to walk safely in a community setting (36,37). Similarly, the SPPB is a physical measure utilized to identify an individual’s future risk of disability, institutionalization, and mortality in the elderly adults (38). CHAMPS was created to improve physical activity in the elderly (39). The survey utilizes a series of questions to measure physical activities which are then employed to estimate caloric expenditure per week (39,40). Together with measures of pulmonary function, these factors are of great importance in an individuals’ ability to remain mobile and active in a community setting. Other quality of life measures included sexual function. Although males did not experience any improvement in erectile dysfunction, women significantly improved their scores on the SQOL in the 100M-group. This is a particularly meaningful marker of improved quality of life, as loss of libido in postmenopausal women is intrinsically linked to hypoactive sexual desire disorder (HSDD), a disorder marked by clinically significant personal distress (41).

Frailty in advancing age is associated with a heightened state of inflammation termed “inflammaging” (42). Markers of chronic inflammation, such as TNF-α and leukocytosis, are all associated with aging and age-related diseases (43). TNF-α in particular has been correlated with increased mortality in the elderly adults (44). MSCs harbor immunomodulatory properties and have been shown to decrease inflammatory markers in several studies (45,46). Therefore, it is not surprising that both the 100M- and 200M-cell doses significantly modulated the immune systems of the treated participants. Serum TNF-α was significantly reduced in the 100M-group, while B cell intracellular TNF-α was reduced in both the 100M- and 200M-groups, and as compared to placebo. Furthermore, there was a suppression of the late/chronically activated T-cells (CD25) at 6 months postinfusion. The 200M-group produced clinically significant decreases in markers of early and late/chronic T-cell activation. Most interestingly, allo-hMSCs significantly reduced the percentage of CD8 T-cells. The risk for infection is increased in aging and is marked by a CD4/CD8 ratio less than one (29). Six months post-allo-hMSC treatment, there was a significant improvement in the immune risk phenotype of the 200M-group. These immune responses due to MSCs are likely to contribute salutary effects and could enhance health span in individuals with aging frailty.

Aging is characterized by a diminished reserve in all organ systems, with impaired stem cell production and/or function being implicated as contributing to the body’s inability to repair itself (47). Chronic inflammation in particular is not only associated with frailty, but also creates a detrimental environment for stem cells and their ability to oppose disease processes (48). Currently, most research on frailty has focused on improvements on physiologic reserve, with a focus on the dysregulation of inflammation (49). Utilizing young healthy individuals as donors for hMSCs, this study addresses both physiologic and inflammatory aspects of aging frailty.

This study is limited by a small sample size. The lack of differences between groups, with the exception of intracellular TNF-α, is due to the study’s small size which limits statistical power. Of note, the point estimate between the 100M-group and placebo in the physical performance metric 6MWD would require 30 patients per group for appropriate statistical power to detect a difference between groups. A future larger study is planned to address this.

In summary, the present study indicates that intravenous allo-hMSC delivery is safe in individuals with aging frailty. Given this excellent safety profile coupled with promising indications of efficacy in the 100M cell group, pivotal clinical trials are warranted to further establish the efficacy of allo-hMSCs in this multisystem disorder, to define optimal dosing of MSCs in this population, and to validate the use of inflammatory biomarkers as a useful surrogate of clinical outcome.

## Supplementary Material

Supplementary data is available at *The Journals of Gerontology, Series A: Biological Sciences and Medical Sciences* online.

## Funding

This work was supported by The Soffer Family Foundation and The Starr Foundation.

## Conflict of Interest

J.M.H. has a patent for cardiac cell-based therapy; he holds equity in Vestion Inc.; maintains a professional relationship with Vestion as a consultant and member of the Board of Directors and Scientific Advisory Board; and is a shareholder in Longeveron LLC. E.A., J.E.-K., and A.M. are employees of the EMMES Corporation. D.L.D., A.K., and A.M.L. maintain a professional relationship with Longeveron, LLC as consultants. A.A.O., G.G., and A.M. are employees of Longeveron, LLC. The other authors declare that they have no competing interests.

## Supplementary Material

Supplementary Material 1

Supplementary Material 2

Supplementary Material 3

## References

[1] Walston J, Hadley EC, Ferrucci L Research agenda for frailty in older adults: toward a better understanding of physiology and etiology: summary from the American Geriatrics Society/National Institute on Aging Research Conference on Frailty in Older Adults. J Am Geriatr Soc. 2006;54:991–1001. doi:10.1111/j.1532-5415.2006.00745.x 16776798 10.1111/j.1532-5415.2006.00745.x

[2] Clegg A, Young J, Iliffe S, Rikkert MO, Rockwood K Frailty in elderly people. Lancet. 2013;381:752–762. doi:10.1016/S0140-6736(12)62167-9 23395245 10.1016/S0140-6736(12)62167-9 PMC4098658

[3] Song X, Mitnitski A, Rockwood K Prevalence and 10-year outcomes of frailty in older adults in relation to deficit accumulation. J Am Geriatr Soc. 2010;58:681–687. doi:10.1111/j.1532-5415.2010.02764.x 20345864 10.1111/j.1532-5415.2010.02764.x

[4] López-Otín C, Blasco MA, Partridge L, Serrano M, Kroemer G The hallmarks of aging. Cell. 2013;153:1194–1217. doi:10.1016/j.cell.2013.05.039 23746838 10.1016/j.cell.2013.05.039 PMC3836174

[5] Ahmed AS, Sheng MH, Wasnik S, Baylink DJ, Lau KW Effect of aging on stem cells. World J Exp Med. 2017;7:1–10. doi:10.5493/wjem.v7.i1.1 28261550 10.5493/wjem.v7.i1.1 PMC5316899

[6] Cerreta F, Eichler HG, Rasi G Drug policy for an aging population–the European Medicines Agency’s geriatric medicines strategy. N Engl J Med. 2012;367:1972–1974. doi:10.1056/NEJMp1209034 23171092 10.1056/NEJMp1209034

[7] Morley JE, Vellas B, van Kan GA Frailty consensus: a call to action. J Am Med Dir Assoc. 2013;14:392–397. doi:10.1016/j.jamda.2013.03.022 23764209 10.1016/j.jamda.2013.03.022 PMC4084863

[8] Rockwood K, Mitnitski A Frailty defined by deficit accumulation and geriatric medicine defined by frailty. Clin Geriatr Med. 2011;27:17–26. doi:10.1016/j.cger.2010.08.008 21093719 10.1016/j.cger.2010.08.008

[9] Fried LP, Ferrucci L, Darer J, Williamson JD, Anderson G Untangling the concepts of disability, frailty, and comorbidity: implications for improved targeting and care. J Gerontol A Biol Sci Med Sci. 2004;59:255–263. doi:10.1093/gerona/59.3.M255 15031310 10.1093/gerona/59.3.m255

[10] Fried LP, Tangen CM, Walston J; Cardiovascular Health Study Collaborative Research Group. Frailty in older adults: evidence for a phenotype. J Gerontol A Biol Sci Med Sci. 2001;56:M146–M156. doi:10.1093/gerona/56.3.M146 11253156 10.1093/gerona/56.3.m146

[11] Collard RM, Boter H, Schoevers RA, Oude Voshaar RC Prevalence of frailty in community-dwelling older persons: a systematic review. J Am Geriatr Soc. 2012;60:1487–1492. doi:10.1111/ j.1532-5415.2012.04054.x 22881367 10.1111/j.1532-5415.2012.04054.x

[12] Xue QL The frailty syndrome: definition and natural history. Clin Geriatr Med. 2011;27:1–15. doi:10.1016/j.cger.2010.08.009 21093718 10.1016/j.cger.2010.08.009 PMC3028599

[13] Tompkins BA, Landin AM, Florea V, Natsumeda M, Reiger AC, Balkan W, Schulman IH, Hare JM Allogeneic Mesenchymal Stem Cells as a Treatment for Aging Frailty. In: Frailty and Sarcopenia - Onset, Development and Clinical Challenges, Yannis Dionyssiotis (ed), InTech, Rijeka, 2017. www.intechopen.com

[14] Peffers MJ, Collins J, Loughlin J, Proctor C, Clegg PD A proteomic analysis of chondrogenic, osteogenic and tenogenic constructs from ageing mesenchymal stem cells. Stem Cell Res Ther. 2016;7:133. doi:10.1186/s13287-016-0384-2 27624072 10.1186/s13287-016-0384-2 PMC5022190

[15] Sethe S, Scutt A, Stolzing A Aging of mesenchymal stem cells. Ageing Res Rev. 2006;5:91–116. doi:10.1016/j.arr.2005.10.001 16310414 10.1016/j.arr.2005.10.001

[16] Stolzing A, Jones E, McGonagle D, Scutt A Age-related changes in human bone marrow-derived mesenchymal stem cells: consequences for cell therapies. Mech Ageing Dev. 2008;129:163–173. doi:10.1016/j.mad.2007.12.002 18241911 10.1016/j.mad.2007.12.002

[17] Golpanian S, DiFede DL, Khan A Allogeneic human mesenchymal stem cell infusions for aging frailty. Epub ahead of print. J Gerontol A Biol Sci Med Sci. 2017. doi:10.1093/gerona/glx056 10.1093/gerona/glx056 PMC5861970 28444181

[18] Hare JM, Traverse JH, Henry TD A randomized, double-blind, placebo-controlled, dose-escalation study of intravenous adult human mesenchymal stem cells (prochymal) after acute myocardial infarction. J Am Coll Cardiol. 2009;54:2277–2286. doi:10.1016/j.jacc.2009.06.055 19958962 10.1016/j.jacc.2009.06.055 PMC3580848

[19] Hare JM, Fishman JE, Gerstenblith G Comparison of allogeneic vs autologous bone marrow-derived mesenchymal stem cells delivered by transendocardial injection in patients with ischemic cardiomyopathy: the POSEIDON randomized trial. JAMA 2012;308:2369–2379. doi:10.1001/jama.2012.25321 23117550 10.1001/jama.2012.25321 PMC4762261

[20] Hare JM, DiFede DL, Rieger AC Randomized comparison of allogeneic versus autologous mesenchymal stem cells for nonischemic dilated cardiomyopathy: POSEIDON-DCM trial. J Am Coll Cardiol. 2017;69:526–537. doi:10.1016/j.jacc.2016.11.009 27856208 10.1016/j.jacc.2016.11.009 PMC5291766

[21] Premer C, Blum A, Bellio MA Allogeneic mesenchymal stem cells restore endothelial function in heart failure by stimulating endothelial progenitor cells. EBioMedicine. 2015;2:467–475. doi:10.1016/j.ebiom.2015.03.020 26137590 10.1016/j.ebiom.2015.03.020 PMC4485912

[22] Weiss DJ, Casaburi R, Flannery R, LeRoux-Williams M, Tashkin DP A placebo-controlled, randomized trial of mesenchymal stem cells in COPD. Chest. 2013;143:1590–1598. doi:10.1378/chest.12-2094 23172272 10.1378/chest.12-2094 PMC4694112

[23] Karantalis V, Hare JM Use of mesenchymal stem cells for therapy of cardiac disease. Circ Res. 2015;116:1413–1430. doi:10.1161/circresaha.116.303614 25858066 10.1161/CIRCRESAHA.116.303614 PMC4429294

[24] Hatzistergos KE, Quevedo H, Oskouei BN Bone marrow mesenchymal stem cells stimulate cardiac stem cell proliferation and differentiation. Circ Res. 2010;107:913–922. doi:10.1161/circresaha.110.222703 20671238 10.1161/CIRCRESAHA.110.222703 PMC3408082

[25] Rockwood K, Song X, MacKnight C A global clinical measure of fitness and frailty in elderly people. CMAJ. 2005;173:489–495. doi:10.1503/cmaj.050051 16129869 10.1503/cmaj.050051 PMC1188185

[26] Golpanian S, DiFede DL, Pujol MV Rationale and design of the allogeneiC human mesenchymal stem cells (hMSC) in patients with aging fRAilTy via intravenoUS delivery (CRATUS) study: a phase I/II, randomized, blinded and placebo controlled trial to evaluate the safety and potential efficacy of allogeneic human mesenchymal stem cell infusion in patients with aging frailty. Oncotarget. 2016;7:11899–11912. doi:10.18632/oncotarget.7727 26933813 10.18632/oncotarget.7727 PMC4914257

[27] Ritt M, Ritt JI, Sieber CC, Gaßmann KG Comparing the predictive accuracy of frailty, comorbidity, and disability for mortality: a 1-year follow-up in patients hospitalized in geriatric wards. Clin Interv Aging. 2017;12:293–304. doi:10.2147/CIA.S124342 28223787 10.2147/CIA.S124342 PMC5308479

[28] Koller K, Rockwood K Frailty in older adults: implications for end-of-life care. Cleve Clin J Med. 2013;80:168–174. doi:10.3949/ccjm.80a.12100 23456467 10.3949/ccjm.80a.12100

[29] McElhaney JE, Effros RB Immunosenescence: what does it mean to health outcomes in older adults? Curr Opin Immunol. 2009;21:418–424. doi:10.1016/j.coi.2009.05.023 19570667 10.1016/j.coi.2009.05.023 PMC2725188

[30] Kawamoto A, Katayama M, Handa N Intramuscular transplantation of G-CSF-mobilized CD34(+) cells in patients with critical limb ischemia: a phase I/IIa, multicenter, single-blinded, dose-escalation clinical trial. Stem Cells. 2009;27:2857–2864. doi:10.1002/stem.207 19711453 10.1002/stem.207

[31] Golpanian S, Schulman IH, Ebert RF; Cardiovascular Cell Therapy Research Network. Concise review: review and perspective of cell dosage and routes of administration from preclinical and clinical studies of stem cell therapy for heart disease. Stem Cells Transl Med. 2016;5:186–191. doi:10.5966/sctm.2015-0101 26683870 10.5966/sctm.2015-0101 PMC4729551

[32] Hamamoto H, Gorman JH 3rd, Ryan LP Allogeneic mesenchymal precursor cell therapy to limit remodeling after myocardial infarction: the effect of cell dosage. Ann Thorac Surg. 2009;87:794–801. doi:10.1016/j.athoracsur.2008.11.057 19231391 10.1016/j.athoracsur.2008.11.057 PMC3021253

[33] Schuleri KH, Feigenbaum GS, Centola M Autologous mesenchymal stem cells produce reverse remodelling in chronic ischaemic cardiomyopathy. Eur Heart J. 2009;30:2722–2732. doi:10.1093/eurheartj/ehp265 19586959 10.1093/eurheartj/ehp265 PMC2777027

[34] Siddiqi S, Sussman MA Cell and gene therapy for severe heart failure patients: the time and place for Pim-1 kinase. Expert Rev Cardiovasc Ther. 2013;11:949–957. doi:10.1586/14779072.2013.814830 23984924 10.1586/14779072.2013.814830 PMC4140652

[35] Madonna R, Van Laake LW, Davidson SM Position paper of the European Society of Cardiology Working Group Cellular Biology of the Heart: cell-based therapies for myocardial repair and regeneration in ischemic heart disease and heart failure. Eur Heart J. 2016;37:1789–1798. doi:10.1093/eurheartj/ehw113 27055812 10.1093/eurheartj/ehw113 PMC4912026

[36] ATS Committee on Proficiency Standards for Clinical Pulmonary Function Laboratories. ATS statement: guidelines for the six-minute walk test. Am J Respir Crit Care Med. 2002;166:111–117. doi:10.1164/ajrccm.166.1.at1102 12091180 10.1164/ajrccm.166.1.at1102

[37] Enright PL, McBurnie MA, Bittner V; Cardiovascular Health Study. The 6-min walk test: a quick measure of functional status in elderly adults. Chest. 2003;123:387–398. doi:10.1378/chest.123.2.387 12576356 10.1378/chest.123.2.387

[38] Guralnik JM, Ferrucci L, Pieper CF Lower extremity function and subsequent disability: consistency across studies, predictive models, and value of gait speed alone compared with the short physical performance battery. J Gerontol A Biol Sci Med Sci. 2000;55:M221–M231. doi:10.1093/gerona/55.4.M221 10811152 10.1093/gerona/55.4.m221 PMC12149745

[39] Stewart AL, Mills KM, King AC, Haskell WL, Gillis D, Ritter PL CHAMPS physical activity questionnaire for older adults: outcomes for interventions. Med Sci Sports Exerc. 2001;33:1126–1141. 11445760 10.1097/00005768-200107000-00010

[40] Mak S, Soicher JE, Mayo NE, Wood-Dauphinee S, Bourbeau J Cross-cultural adaptation of the CHAMPS questionnaire in French Canadians with COPD. Can Respir J. 2016;2016:9304505. doi:10.1155/2016/9304505 27445570 10.1155/2016/9304505 PMC4906179

[41] Goldstein I, Kim NN, Clayton AH Hypoactive sexual desire disorder: International Society for the Study of Women’s Sexual Health (ISSWSH) Expert Consensus Panel Review. Mayo Clin Proc. 2017;92:114–128. doi:10.1016/j.mayocp.2016.09.018 27916394 10.1016/j.mayocp.2016.09.018

[42] de Gonzalo-Calvo D, Neitzert K, Fernández M Differential inflammatory responses in aging and disease: TNF-alpha and IL-6 as possible biomarkers. Free Radic Biol Med. 2010;49:733–737. doi:10.1016/j.freeradbiomed.2010.05.019 20639132 10.1016/j.freeradbiomed.2010.05.019

[43] Kanapuru B, Ershler WB Inflammation, coagulation, and the pathway to frailty. Am J Med. 2009;122:605–613. doi:10.1016/j.amjmed.2009.01.030 19559159 10.1016/j.amjmed.2009.01.030 PMC5999023

[44] Bruunsgaard H, Andersen-Ranberg K, Hjelmborg Jv, Pedersen BK, Jeune B Elevated levels of tumor necrosis factor alpha and mortality in centenarians. Am J Med. 2003;115:278–283. doi:10.1016/S0002-9343(03)00329-2 12967692 10.1016/s0002-9343(03)00329-2

[45] Perico N, Casiraghi F, Gotti E Mesenchymal stromal cells and kidney transplantation: pretransplant infusion protects from graft dysfunction while fostering immunoregulation. Transpl Int. 2013;26:867–878. doi:10.1111/tri.12132 23738760 10.1111/tri.12132

[46] Golpanian S, Wolf A, Hatzistergos KE, Hare JM Rebuilding the damaged heart: mesenchymal stem cells, cell-based therapy, and engineered heart tissue. Physiol Rev. 2016;96:1127–1168. doi:10.1152/physrev.00019.2015 27335447 10.1152/physrev.00019.2015 PMC6345247

[47] Rando TA, Wyss-Coray T Stem cells as vehicles for youthful regeneration of aged tissues. J Gerontol A Biol Sci Med Sci. 2014;69(suppl 1):S39–S42. doi:10.1093/gerona/glu043 10.1093/gerona/glu043 PMC4022127 24833585

[48] Raggi C, Berardi AC Mesenchymal stem cells, aging and regenerative medicine. Muscles Ligaments Tendons J. 2012;2:239–242. 23738303 PMC3666525

[49] Ershler WB, Keller ET Age-associated increased interleukin-6 gene expression, late-life diseases, and frailty. Annu Rev Med. 2000;51:245–270. doi:10.1146/annurev.med.51.1.245 10774463 10.1146/annurev.med.51.1.245

