# Supplementary material for: Allogeneic Mesenchymal Stem Cells Ameliorate Aging Frailty: A Phase II Randomized, Double-Blind, Placebo-Controlled Clinical Trial
Source: J Gerontol A Biol Sci Med Sci. 2017 Jul 17;72(11):1513–22. doi: 10.1093/gerona/glx137 (PMC5861900; doi:10.1093/gerona/glx137)
Supplement: Supplementary Material 1 [file glx137_suppl_supplementary_material_1.docx]

**Supplementary Material:**

**Methods**

*Study design, stem cell procurement and randomization*

All patients provided written informed consent. MSCs were allogeneic, human bone marrow-derived, and manufactured at the University of Miami. There were a total of 7 donors, 6 of which were male. The ages of the donors ranged from 19-27. Each of the donor’s bone marrow-derived MSCs were isolated, characterized, and expanded in vitro. Each donor’s MSCs were > 95% positive for CD105 (cut off > 80%) and ≤ 1% for CD45 (cut off < 2%). MSCs were free from aerobic/anaerobic bacteria, fungus, mycoplasma and endotoxin. The details of the donor’s eligibility and cell harvesting have been published.[^25^](#_ENREF_25) The randomization of placebo vs. allo-hMSC doses was accomplished using the Emmes Corporation’s Internet Data Entry System (IDES). As each patient was enrolled, IDES provided the blinded treatment assignment from the randomization scheme prepared by Emmes statisticians. Thirty patients were randomized in a 1:1:1 fashion to either one of two doses of allo-hMSCs vs. placebo. Group A consisted of 10 subjects that received 100M cells, group B (10 subjects) received 200M cells, and Group C (10 subjects) received placebo.

*Immune Monitoring*

Calculated panel reactive antibodies (cPRA), early and late/chronic markers of T-cell activation, Immune Risk Phenotype (CD4/CD8 ratio), serum TNF-α, and percent of B cells expressing intracellular TNF-α were measured at baseline and 6-months using a panel of color-coded microbeads coated with HLA antigens for Class I and II and measured using the Luminex-200. T-cell activation was monitored by early and late/chronic markers of T-cell activation (CD3-FITC, CD25-APC, CD69-PE-Cy7). The Immune Risk Phenotype (CD4/CD8 ratio) was also assessed (FITC, CD3-FITC, CD4-APC, CD8-PE, CCR7-AlexaFluor700 and CD45RA-PacBlue). Utilizing the human TNF-α ELISA high sensitivity kit (eBiosciences), the serum TNF-α was quantified. The percent of B cells expressing intracellular TNF-α were stained with surface CD19-PE and intracellular TNF-α APC and were gated on CD19^+^. All antibodies used were from BD Pharmigen and isotype controls were used for proper gating. All stained cells were acquired with the LSR-Fortessa-HTS analyzer (BD Pharmigen) and analyzed with the FlowJo-V10 software.

*Statistical analysis*

No formal statistical justification was performed to determine sample size for this study. Sample size was determined to be appropriate for an early phase study to assess safety in this population. Due to the early phase nature of this study, no adjustments were made for multiple analyses.[^25^](#_ENREF_25) Under the assumption that α=0.05 level of significance, all two-sided statistical tests were performed with 95% confidence intervals. Categorical variables are summarized using counts (n) and percents (%). Baseline continuous data are summarized with mean and standard deviation. Data that are highly skewed was reported using the median, 25^th^ and 75^th^ percentiles, the minimum and maximum values, and the number of patients with non-missing data. A mixed model for repeated measures was used to compare treatment groups where outcomes are collected at baseline and follow-up visits. Model estimated within-group tests were also performed. Data that are non-normal was analyzed using rank analysis of covariance at each follow-up time point adjusting for baseline. Within-group tests for non-normal data were computed using Wilcoxon signed rank sum tests. All available data were used in analysis with no imputations for missing values. All data analyses and statistical computations were conducted with SAS, version 9.3 (Cary, NC), with the exception of intracellular TNF-α that utilized GraphPad Prism to conduct a Kruskal-Wallis test.

Frequency, severity, organs affected, and relationship to exposure were presented for all adverse events. A Fisher’s exact test was used to compare AEs and SAEs between each of the three groups. Descriptive measurements were utilized to summarize lung functionality. Each questionnaire contained guidelines that were used to summarize outcome data as reported by each subject.

**Hospitalizations:**

There were a total of 7 hospitalizations, 2 were in the 100M-group, and 5 were in the placebo. The reasons for the hospitalizations were as follows: Patient 36 (100M-group) was hospitalized for abdominal pain secondary to an incidental renal cell carcinoma and was subsequently rehospitalized for a nephrectomy. Patient 16 (placebo) was hospitalized for cholecystitis which was followed by another hospitalization for hypotension and was rehospitalized for a glioblastoma. Patient 21 (placebo) was hospitalized for gastroenteritis. Patient 22 (placebo) was hospitalized for a brain aneurysm. None of the hospitalizations were related to the treatment.
