# Supplementary material for: Allogeneic Mesenchymal Stem Cells Ameliorate Aging Frailty: A Phase II Randomized, Double-Blind, Placebo-Controlled Clinical Trial
Source: J Gerontol A Biol Sci Med Sci. 2017 Jul 17;72(11):1513–22. doi: 10.1093/gerona/glx137 (PMC5861900; doi:10.1093/gerona/glx137)
Supplement: Supplementary Material 2 [file glx137_suppl_supplementary_material_2.pdf]

This supplement contains the following items:

1. Original Protocol
2. Final Protocol
3. Summary of Changes

Interdisciplinary Stem Cell Institute

University of Miami/ Miller School of Medicine

Clinical Research Protocol

Study Title: A Phase I/II, Randomized, Blinded and Placebo-controlled Trial to Evaluate the Safety and Potential Efficacy of Allogeneic Human Mesenchymal Stem Cell Infusion in Patients with Aging Frailty.

***A*llogenei*C*HumanMesench*y*mal*S*tem C*e*lls (hMSC) in Patients with Aging F*RA*il*T*y via Intraveno*US*Delivery (CRATUS)**

Study Product: Allogeneic Human Mesenchymal Stem Cells (hMSCs)

Indication: Aging Frailty

FDA IND No.: BB-IND #TBD

Principal Investigator: Joshua M. Hare, M.D. Telephone: 305-243-5579

Protocol Version: July 22, 2013, Version 1.0

Protocol Agreement Signature:

Joshua M. Hare, M.D(Sponsor)

7/22/13
Date

CONFIDENTIALITY STATEMENT

This document is confidential and proprietary to the Interdisciplinary Stem Cell Institute University of Miami/Miller School of Medicine and its affiliates. Acceptance of this document constitutes agreement by the recipient that no unpublished information contained herein will be reproduced, published, or otherwise disseminated or disclosed without prior written approval of the Interdisciplinary Stem Cell Institute University of Miami or its affiliates, except that this document may be disclosed in any medium to appropriate clinical investigators, Institutional Review Boards, and others directly involved in the clinical investigation that is the subject of this information under the condition that they keep the information strictly confidential.

Table of Contents

List of Abbreviations 6
Protocol Synopsis 8
1. INTRODUCTION 11
1.1. Background 11
1.2. Mesenchymal Stem Cells 13
1.3. Mesenchymal Stem Cells: Preclinical Experience 14
1.4. Allogeneic Mesenchymal Stem Cells: Previous Experience in Humans 27
1.5. Pharmacology and Toxicology Studies of Mesenchymal Stem Cells 34
2.. Secondary Objectives 36
2.1. Study Endpoints **36**
2.1.1 Primary Endpoints (Safety) **36**
2.1.2. Secondary Endpoints (Efficacy) **36**
3. STUDY DESIGN 37
3.1 Description of the Study 37
3.2. RANDOMIZATION STUDY 37
4. SUBJECT SELECTION 38
4.1 Inclusion Criteria 38
4.2 Exclusion Criteria 38
4.3 Concomitant Treatments, Procedures, and Nondrug Therapies 39
4.4 Withdrawal Criteria 39
5. MESENCHYMAL STEM CELL DONORS 40
5.1 Bone Marrow Aspiration for Generation of MSCs 40
5.2 Normal Donor Eligibility 40
5.3 Donor Consent 42
5.4 Follow-up Schedule for Donors 42
6. TREATMENT OF PATIENTS 42
6.1 Study Investigational Product 42
6.2 Dosing 42
6.3 Dosage Rationale 43
6.4 Administration Rate 43

6.5 Concomitant Therapy 44
6.5.1 Permitted therapy 44
6.5.2 Excluded therapy 44
6.5.3. Subject monitoring 44
6.6 Blinding and Unblinding 44
6.7 Study Investigational Therapy Management 44
6.7.1 Investigational Product Labeling and Storage 45
6.7.2 Investigational Product Accountability Procedures 45
7. STUDY PROCEDURES 45
7.1. Time and Events Schedule 45
Time and Events Table Key: 47
7.2.1 Screening Visit 48
7.2.2 Baseline Visit 48
7.2.3 Day 1 Visit 48
7.2.5 Month 12 Visit 49
7.2.6 Immune Monitoring for Graft Rejection 49
8. SAFETY 50
8.1 Safety Variables 50
8.2 Laboratory Evaluations 50
8.2.2 Pulse Oximetry 50
8.2.3 Pregnancy 51
8.2.4 Determination of Infusional Toxicity 51
8.2.5 Subject Stopping Guidelines 51
8.3 Definition of an Adverse Event 53
8.4 Definition of Adverse Reaction 53
8.5 Definition of Suspected Adverse Reaction 54
8.6 Definition of Serious 54
8.7 Definition of Unexpected 54
8.8 Clinical Laboratory Assessments and Other Abnormal Assessments as Adverse Events and Serious Adverse Events 55
8.9 Recording of Adverse Events and Serious Adverse Events 55
8.10 Intensity of Adverse Events and Serious Adverse Events 55
8.11 Causality of Adverse Events and Serious Adverse Events 56
8.12 Follow-Up of Adverse Events and Serious Adverse Events 57

8.13 Timeframes for Submitting SAE Reports 57
8.14 Post-Study Adverse Events and Serious Adverse Events 58
8.15 Regulatory Aspects of Adverse Event Reporting 58
9. STATISTICAL ANALYSIS 59
9.1 Determination of Sample Size and Analysis Population 59
9.2 General Statistical Methods 59
9.3 Interim Analyses 59
9.4 Data Safety Monitoring Board (DSMB) 59
9.4.1. ROLE OF THE DSMB 59
9.4.2. Purpose of the DSMB 60
9.4.4 DATA FLOW 64
9.4.5 COMMUNICATION 65
10. STUDY ADMINISTRATION 66
10.1 Regulatory Authority Approval 66
10.2 Ethics Approval 66
10.3 Patient Informed Consent 66
10.4 Confidentiality of Information 67
10.5 Payments to Patients 68
APPENDIX 1: Infusion Guidelines 69
11. References 71

Authorship Team (alphabetical order)

The following individuals provided substantial input during protocol development:

Darcy L. DiFede, RN, BSN	University of Miami
Joshua M. Hare, MD*	University of Miami
Marietsy V. Pujol, MBA	University of Miami

* Principal investigator

List of Abbreviations

AE	Adverse event
ahMSCs	Allogeneic human Mesenchymal Stem Cells
Allo	allogeneic
ALT	Alanine aminotransferase
AST	Aspartate aminotransferase
BDI	Monoclonal Antibody Against Human Bladder Carcinoma
BM	Bone Marrow
CBC	Complete Blood Count
CFR	Code of Federal Regulations
CMV	Cytomegalovirus
CPL	Cell Processing Laboratory
CRP	C-Reactive Protein
CT	Computed tomography
DLCO	Diffusing Capacity
DMSO	Dimethyl sulfoxide
DSMB	Data Safety Monitoring Board
ECC	Eluerian Circumferential strain
EDV	End-diastolic volume
EPCs	Endothelial progenitor cells
ESR	Expedited safety report
ESV	End-systolic volume
FBS	Fetal Bovine Serum
FDA	Food and Drug Administration
FEV – 1	Forced expiratory volume in 1 second
FSH	Follicle stimulating hormone
FVC	Forced vital capacity
GCP	Good Clinical Practice
G-CSF	Granulocyte colony stimulating factor
GFP	Green fluorescent protein
GGT	Y-glutamyl transaminase
GM – CSF	Granulocyte Macrophage Colony Stimulating Factor
GVHD	Graft versus host disease
HBcAb	Anti-Hepatitis B core antibody
HCV Ab	Anti-Hepatitis C virus antibody
HIPAA	Health Insurance Portability and Accountability Act Authorization
HIV	Human Immunodeficiency Virus
HLA	Human leukocyte antigen
Has	Human serum albumin
hMSCs	Human mesenchymal stem cell

HSCs	Hematopoietic stem cells
HTLV	Human T-lymphotropic Virus
ICF	Informed Consent Form
ICH	International Conference on Harmonization
IL-6	Interleukin-6
IND	Investigational new drug
IP	Investigational Product
IRB /IEC	Institutional Review Board
IV	Intravenous Infusion
LAD	Left anterior descending
LV	Left ventricular
MI	Myocardial Infarction
MNC	Mononuclear Cell
MRI	Magnetic resonance imaging
MSCs	Mesenchymal Stem Cells
NAT	Nucleic Acid Testing
NIH	National Institute of Health
NMDP	National Marrow Donor Program
PBMC	Peripheral blood mononuclear cells
PFTs	Pulmonary function tests
PSURs	Periodic Safety Update Reports
QOL	Quality of life
RDW	Red blood cell distribution
RPR	Rapid Plasma Raegin
SAE	Serious Adverse Event
SAP	Statistical Analysis Plan
SCA-1	Stem cell factor antigen
SF – 36	Short Form – 36
SGRQ	St. George's Respiratory Questionnaire
TE-SAE	Treatment-emergent serious adverse event
TNF-α	Tumor necrosis factor-alpha
TTC	Triphenyltetrazolium cholride
VEGFR2	Vascular endothelial growth factor
WBC	White blood cell
WNV	West nile virus
6MWT	Six minute walk test

Protocol Synopsis

PRODUCT	Intravenous Allogeneic Adult Human Mesenchymal Stem Cells (MSCs)
PHASE OF DEVELOPMENT	I/II
MAIN CRITERIA FOR INCLUSION	Aging Frailty
STUDY OBJECTIVES	To demonstrate the safety of allogeneic hMSCs administered in patients with Frailty and to explore treatment efficacy (decrease in frailty, frequency of acute exacerbations, change in symptom related quality of life, improved cardiovascular status, decrease in inflammatory biomarkers, and 1 year survival).
STUDY DESIGN	A Phase I/II, Randomized, Blinded and Placebo-controlled
INVESTIGATIONAL PLAN	Before initiating the full randomized study, a Pilot Safety Phase will be performed. The randomized portion of this trial will be conducted after a full review of the safety data from the Pilot Phase by the DSMB.<br><br>Following the Pilot Phase of fifteen (15) subjects, thirty (30) subjects are scheduled to undergo infusion and meeting all inclusion/exclusion criteria will be evaluate at baseline.<br><br><u>Pilot Phase</u> (15 subjects)<br><br><u>Group 1 (5 subjects)</u>:<br><br>Five (5) subjects will be treated with a single administration of allogeneic hMSCs: 2 x 10^7 (20 million) cells delivered via peripheral intravenous infusion.<br><br><u>Group 2 (5 subjects)</u>:<br><br>Five (5) subjects will be treated with a single administration of allogeneic hMSCs: 1 x 10^8 (100 million) cells delivered via peripheral intravenous infusion.<br><br><u>Group 3 (5 subjects)</u>:<br><br>Five (5) subjects will be treated with a single administration of allogeneic hMSCs: 2 x 10^8 (200 million) cells delivered via peripheral intravenous infusion.<br><br>In the randomized phase of allo-hMSCs or matched placebo 30 subjects will be randomized in a 1:1:1 ratio to one of two doses of MSCs versus placebo.

	Treatment Strategies following successful completion of the Pilot Phase.<br><br>Group A (10 subjects) – Allogeneic hMSCs: 100 million cells/ml delivered via peripheral intravenous infusion.<br><br>Group B (10 subjects) – Allogeneic hMSCs: 200 million cells/ml delivered via peripheral intravenous infusion.<br><br>Group C (10 subjects) - Placebo delivered via peripheral intravenous infusion.<br><br>The Allo-hMSCs will be supplied from an allogeneic human mesenchymal stem cell source manufactured by the University of Miami.<br><br>Following infusion, patients will followed at 2 weeks post-infusion, and at three and six months to complete all safety and efficacy assessments. Patients will also have a final twelve-month contact for assessment of vital status and occurrence of hospitalization.
ROUTE OF ADMINISTRATION	Peripheral Intravenous Infusion
DURATION OF STUDY PARTICIPATION	12 months (Follow-up will be at 2 weeks, 3, 6, and 12 months.)
SUBJECT POPULATION	Forty-Five (45) subjects with frailty will be enrolled in the study.
Definition of Endpoints	**Safety (Primary):** Incidence (at one month post-catheterization) of any treatment-emergent serious adverse events (TE-SAEs), defined as the composite of: death, non-fatal MI, stroke, hospitalization.<br><br>**Efficacy (Secondary):**<br>• Difference in rate of change of frailty defined as:<br>- Reduced Activity<br>- Slowing of Mobility<br>- Weight Loss<br>- Diminished handgrip strength<br>- Exhaustion<br><br>• Difference in subject quality of life assessment(s):<br>• Death from any cause.<br>• Change between baseline and 6 months in exercise induced ejection fraction<br>• Change between baseline and 6 months the following panel of inflammatory markers: CRP, IL-6, D-dimer, fibrinogen, CBC with differential, DNA, CMV,

	and TNFα
Safety (Additional)	• During the 12 week follow-up period and each consecutive time-point up until the final visit
Inclusion Criteria	• Diagnosis or symptoms of frailty<br>• Provide written informed consent.<br>• Subjects age 60$\geq$ years at the time of signing the Informed Consent<br>• Female subjects must be surgically sterile or post-menopausal (>1 year).
Exclusion Criteria	• Female subjects capable of childbearing, currently pregnant or nursing.<br>• Inability to perform any of the assessments required for endpoint analysis.<br>• Active listing (or expected future listing) for transplant of any organ.<br>• Clinically important abnormal screening laboratory values.<br>• Serious comorbid illness or any other condition that, in the opinion of the investigator, may compromise the safety or compliance of the patient or preclude successful completion of the study.<br>• Have known allergies to penicillin or streptomyocin.<br>• Hypersensitivity to dimethyl sulfoxide (DMSO).<br>• Be an organ transplant recipient.<br>• Have a clinical history of malignancy within 5 years (i.e., patients with prior malignancy must be disease free for 5 years), except curatively-treated basal cell carcinoma, squamous cell carcinoma, or cervical carcinoma.<br>• Have a non-pulmonary condition that limits lifespan to < 1 year.<br>• Have a history of drug or alcohol abuse within the past 24 months.<br>• Be serum positive for HIV, hepatitis BsAg or Viremic hepatitis C.<br>• Be currently participating (or participated within the previous 30 days) in an investigational therapeutic or device trial.

1. INTRODUCTION

1.1 Background

Frailty in an aging population is defined as a medical syndrome "with multiple causes and contributors that is characterized by diminished strength, endurance, and reduced physiologic function that increases an individual's vulnerability for developing increased dependency and/or death"[1]. Of great importance is that frailty, while not characterized as a disability, per se, does increase the risk of death in affected individuals[2-4]. Moreover, there is a close link between a patient's health and frailty. These patients tend to show a greater risk to frailty when there are other conditions affecting their physical and psychological well-being, such as high blood pressure, cancer, or cognitive impairment. In an aging population frailty is characterized by reduced physical activity, slowing of mobility, weight loss, diminished handgrip strength, and exhaustion[2, 3]. There are several well-validated models to assess frailty [CITE MORLEY]. For example, the FRAIL scoring index provides a clinical diagnosis of frailty when a patient exhibits three or more of these five characteristics[2;5]. The forward trajectory in medical advances, and a more health aware society, indicates that the population is living longer and requiring more care and services than were needed in the past. As such, the increase in frail elderly patients has adversely increased the demand for healthcare services. Currently there is a favorable movement in researching a novel medical therapy for frailty as this remains an unmet need amongst the elderly population.[6] It is widely perceived that frailty can be favorably modified, and in this regard there is a major need for effective management tools and treatment strategies. In this protocol, we will test the impact of a safe cell-based therapeutic on the frailty syndrome.

Frailty and Cardiovascular Performance

The aging cardiovascular system has some very specific phenotypic alterations[7;8]. These include left ventricular hypertrophy and a diminution in exercise induced increase in ejection fraction[9-11]. These characteristic abnormalities are hypothesized to contribute to specific symptoms of the frailty syndrome and to increase the morbidity and mortality from cardiovascular disease in elderly individuals.

Several studies document the increased risk for mortality in frail elderly patients with cardiovascular events such as non-ST-segment elevation myocardial infarction (NSTEMI)[4;12]. Frail individuals have increased disease burden in and therefore more prolonged recuperation versus a non-frail subject[1]. There are additional associations between frailty and other cardiovascular diagnoses including angina, myocardial infarction, congestive heart failure and stroke. Gait speed is one symptom of frailty that is associated with cardiovascular mortality and an increased risk of cardiovascular events specifically STEMI patients [13;14]. The Women's Health Initiative utilized the "Frailty Index" by Fried and colleagues[8] which was validated in the Cardiovascular Health Study[15] and the Womens Health and Aging Study. These studies demonstrated correlations in functional decline, increased risk of institutionalization and mortality with the use of these instruments.

The strong association between frailty and cardiovascular disease and the growing data base documenting safety and potential favorable effects of cell-based therapy in cardiovascular diseases provide justification for the assessment of potential benefits of cell therapy in subjects with frailty.

Inflammation in frailty

In addition and of great importance, there is a growing database of studies that highlight a connection between frailty and inflammation. For example, certain inflammatory markers, such as C-reactive protein (CRP), fibrinogen, interleukin-6 (IL-6), red blood cell distribution (RDW) and D-dimer are more likely to be elevated in frail as compared to non-frail individuals[8]. Importantly, among frail subjects, women exhibit higher concentrations of inflammatory and coagulation factors than men. CRP is an example of one marker studied that shows that women experiencing symptoms of frailty show a higher concentration of CRP. Differential white cell counts on the other hand exhibits an increased risk of frailty in both men and women. Dysregulated inflammation is a considerable key physiological marker in correlation with the frailty syndrome[8] . There is still insufficient data to show which markers specifically affect men or women[5;16]. It is believed that frailty can ultimately be prevented or attenuated, and the link between frailty and inflammation offers a potential therapeutic target, also addressable by cell therapy.

Frailty is assessed with several instruments. For example, the Clinical Frailty Scale[2] is a clinical scale that determines the degree of a patient's frailty based on the physician's judgment and the subject's medical information. This scale consists of 7 variables ranging from fit to complete functional dependence; If a patient displays three or more of the following symptoms: low physical activity, muscle weakness, slowed performance, fatigue, unintentional weight loss, then the patient is considered to be classified as frail.[2;4]

Stem Cells in Frailty

An individual's endogenous stem cell production decreases with age; this decrease in an aging subject likely contributes to reduced ability to regenerate and repair organs and tissues. Several investigators have proposed that a regenerative treatment strategy could ameliorate signs and symptoms of aging frailty[8;16]. Currently, the FDA does not have any specific approved treatment for frail patients and therefore no established standard of care. In many cases frailty can be masked by other physical or psychological conditions affecting well-being and functional status. The ultimate goal is to extend the health and ability of a patient to regenerate functionality. Allogeneic Human Mesenchymal Stem Cells are known to hone to sites of injury, reduce inflammation, and assist in cellular repair. Here we propose to study Allo-hMSCs (ahMSCs) as a novel therapy for treating patients experiencing frailty.

There are specific features of the frailty syndrome that support a potential role of ahMSCs to ameliorate or improve frailty. Notably, ahMSCs are shown to improve cardiovascular status in patients with acute MI[17] and heart failure[18]. In addition, ahMSCs are anti-inflammatory and reduce CRP levels in a sustained manner[19]. Importantly, data from heart failure studies show that ahMSCs are safe in patients irrespective of age[18]. For these reasons, this protocol will test the safety of intravenous infusion (IV) of ahMSCs in individuals of advanced age with frailty, and will assess cardiovascular status and inflammatory markers in this population at increased risk for morbidity and mortality.

Cells derived from adult bone marrow

Bone marrow harbors a variety of cells that may contribute to vasculogenesis or cardiomyogenesis, either directly, or by facilitating endogenous repair mechanisms. Bone marrow cells have been prepared on the basis of being 1.) endothelial precursor cells that are $CD34^+$, 2.) MSCs purified without an antigen panning technique on the basis of their fibroblast morphology, ability to divide in culture and to differentiate into mesodermal lineages[20], and 3.) cells that express stem cell factor receptor, c-Kit[21;22]. Endothelial progenitor cells (EPCs) express the surface markers CD34, CD133, c-kit, and the vascular endothelial growth factor receptor-2 (VEGFR2; KDR; Flk-1)[23-28]. Hematopoietic stem cells (HSCs) exhibit self-renewal and differentiation. Their cell-surface phenotype is $CD34^+$, stem cell factor antigen $(SCA\text{-}1)^+$, $c\text{-}kit^+$, and Lin^- (review[29]). While there has been controversy regarding the ability of bone marrow-derived cells to transdifferentiate into cardiomyocytes[30], clinical trials of bone marrow therapies continue to suggest potential benefit in terms of improving a patient's well-being.

1.2 Mesenchymal Stem Cells

Mesenchymal stem cells (MSCs) are multipotent cells capable of differentiating into a number of different cell lines. Because of their unique combination of multipotency, migratory ability, and immunopriveleged state (MSCs do not express major histocompatibility factor-II making allogeneic transplant possible)[31], interest has abounded regarding their potential therapeutic and regenerative applications. In fact, MSCs have been shown to hold promise as a novel therapeutic agent in multiple disease processes. Treatment with MSCs has been shown to ameliorate severe graft versus host disease[31], contribute to pancreatic islet and renal glomerular repair in diabetes[32], attenuate sepsis[33], reverse fulminant hepatic failure[34], protect against ischemic acute renal failure[35], reverse remodeling[36-38] and improve cardiac function after myocardial infarction[17], to be a potential source of multiple cell types for use in tissue engineering[39;40], and to be capable of tissue regeneration after spinal cord trauma, stroke, and connective tissue injury[41-43].

In the lung, MSCs have been shown to contribute to tissue regeneration after elastase-induced emphysema[44], home to sites of asbestos induced lung injury[45], contribute to tissue remodeling in a rat monocrotaline model of pulmonary hypertension[46], decrease chronic airway inflammation in a murine ovalbumin model of asthma[47], and to restore alveolar fluid balance after endotoxin induced acute lung injury[48].

Tracking of radioactively labeled cells shows that when administered intravenously, MSCs localize primarily to the lung, followed by the liver, and then other organs[49]. A number of studies show that MSCs preferentially home to sites of injury in the lung and contribute to tissue regeneration and repair[50-56]. Using Y-chromosome fluorescence in-situ hybridization, Y-chromosome positive male MSCs can be found at sites of lung injury in transplanted female mice[51;52;55]. These male MSCs appear to adopt anepithelial cell morphology, suggesting that they contribute to tissue regeneration either by fusion with resident epithelial cells or by mesenchymal to epithelial transition[51].

The ability of MSCs to differentiate towards an epithelial lineage was established by studies showing that they are capable of differentiation not only to cells of mesodermal origin, but also to cells of endodermal and ectodermal (including epithelial) origin[57;58]. MSCs cultured in airway growth media differentially express lung specific epithelial markers like Clara cell secretory protein, surfactant protein-C, and thyroid transcription factor-1[57;58]. In addition, in-situ hybridization studies with co-staining for green fluorescent protein (GFP) and epithelial markers shows that GFP-labeled MSCs assume an epithelial phenotype at sites of lung injury and contribute to tissue repair[56;59]. It is worth noting, however, that not all authors agree, with some suggesting that technical difficulties associated with immunofluorescence microscopy have led to the false conclusion that MSCs contribute to alveolar epithelium[60;61].

1.3 Mesenchymal Stem Cells: Preclinical Experience

Several cell-based therapies results propose that infusion of mesenchymal stem cells is a safe and novel approach believed to be an effective strategy to decrease symptoms of frailty. Below we review the impact of MSCs on the cardiovascular system following injury; this provides support for an impact of MSCs in individuals with frailty.

A porcine model of anterior myocardial infarction was used to characterize the impact of cellular cardiomyoplasty on cardiac structure and function using hemodynamic, imaging, and histological analyses. A pig model was selected because of its anatomic similarity to the human heart. The following sections describe the safety and efficacy results obtained with this model[62]. Two distinct sets of studies were conducted, representing the early treatment of acute myocardial infarction, as well as the treatment of chronic ischemic cardiomyopathy.

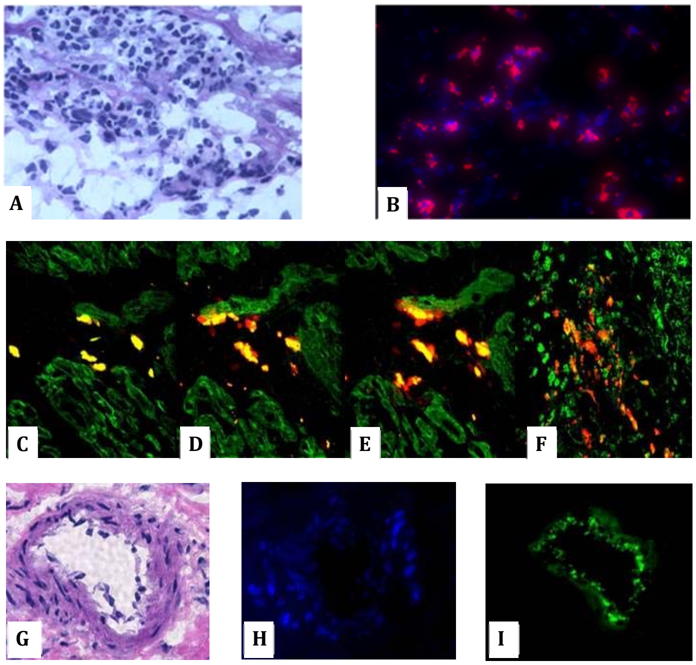

Figure 1. MSC engraftment and differentiation.MSC engraftment and muscle-specific protein-expression. DAPI and Di-I labeled MSCs (blue staining nuclei and red staining membranes, respectively) and fluorescent muscle protein-specific antibodies (green). (A) Hematoxylin and eosin (H&E)–stained section and corresponding fluorescent detection of cellular labels (B) depicts a cluster of MSCs in proximity to host myocardium. Several muscle-specific proteins are detected by immunofluorescence including α-actinin (C), phospho- lamban (D), tropomyosin (E) and troponin T (F). Yellow fluorescence indicates colocalization of immunofluorescent antibodies and DiI. (G) H&E stained sections of vascular structures at the border of the infarcted myocardium. Corresponding sections depict DAPI stained MSC nuclei (H) with immunofluorescent detection of factor 8 (I).

***Allogeneic mesenchymal stem cell transplantation improves global cardiac function in a swine model of acute myocardial infarction*:** Previously published work demonstrated that autologous MSC transplantation in post-MI pigs improved cardiac function, with histological evidence of robust engraftment at 8 weeks, and

differentiation to a myocyte-like phenotype[63]. Based on *in vitro* observations that MSCs lack the B-7 costimulatory molecule and may therefore be immune-privileged, the impact of *allogeneic* MSC transplantation in porcine MI was assessed. A 14 pig randomized, placebo-controlled study (MSCs vs. placebo) using the BioCardia Helical Infusion Catheter was performed to assess safety and efficacy of allogeneic transendocardial injections[62]. Farm pigs were chronically instrumented to measure left-ventricular pressure, dimension, and oxygen consumption, and were randomized to active treatment or placebo groups. Three days following MI, placebo (n=7) or $2X10^8$ allogeneic MSCs (n=7) labeled with Di-I and DAPI (both fluorescent dyes to aid histochemical identification) were injected percutaneously into infarcted myocardium of the left ventricular cavity using a helical injection needle catheter inserted through a steerable guide catheter (BioCardia, Inc.). All animals tolerated the catheter-based injections well. Animals were then studied on a weekly basis for 8 weeks to assess hemodynamics and to examine ventricular architecture. In treated animals, MSCs engrafted within the MI (Figure 1 a, b) and expressed several myocyte proteins, including α-actinin, phospholamban, tropomyosin, and troponin T (Figure 1 c, d, e, f). In addition, there was evidence of stem-cell differentiation or incorporation into vascular structures within the infarct area (Figure 1 g, h, i). MSCs were detected in vascular structures as they expressed VEGF and vonWillebrand Factor, suggesting that they are capable of differentiating into vascular smooth muscle and/or endothelium. That the cells did

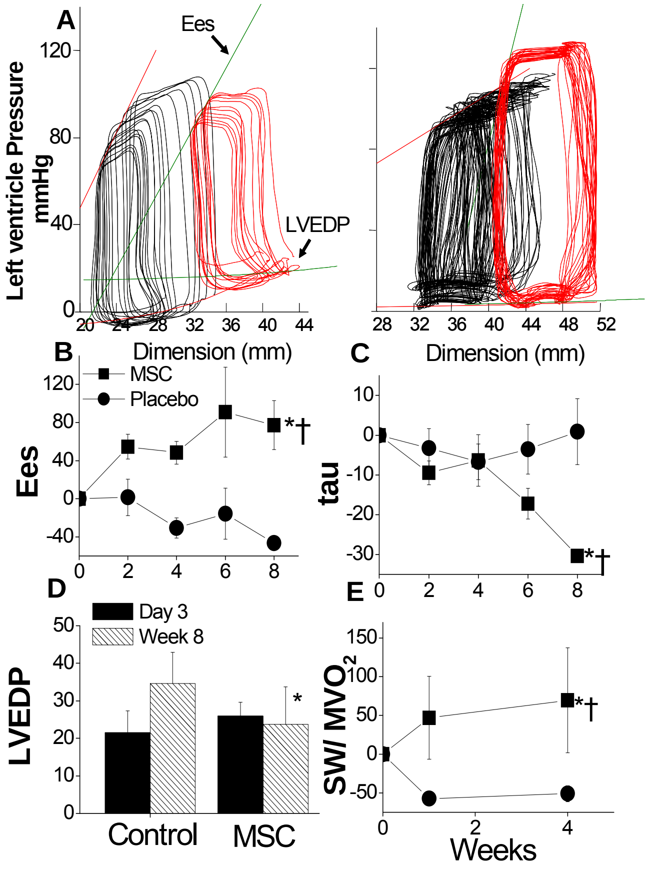

Figure 2.Physiologic impactof MSCs delivered with the BioCardia Catheter following anterior myocardial infarction (MI) in pigs. **(A)** Pressure-dimension (PD) data from placebo (left) and an MSC-treated (right) pig obtained 3 days (black loops) and 8 weeks (red loops) following MI. Placebo animals exhibit an increase in left-ventricular end-diastolic pressure (LVEDP) and dimension. Both myocardial contractility, measured by the slope of the end systolic pressure-dimension relationship (ventricular elastance, Ees), and ventricular stroke work, pressure-dimension loop area, decline in controls. In MSC-treated animals, Ees and stroke work increase to normal. **(B-E)** Average hemodynamic responses over 8 weeks showing divergent responses in cardiac function in MSC vs. placebo treated animals. **(B)**Ees declines in placebo-treated pigs but increases in the MSC group. **(C)**Isovolemic ventricular relaxation (τ), reduces to normal in MSC pigs but remains unchanged in placebo. **(D)** LVEDP increases in placebo but remains unchanged in MSC pigs. **(E)** Stroke work declines in placebo-treated animals while myocardial oxygen consumption (MVO2) increases (81±10.4%), leading to reduced SW/MVO_2. In contrast, in MSC-treated pigs, stroke work increases 89.8±15.3%, MVO_2 decreases 48.9±16.7%, resulting in augmented SW/MVO_2 and restoration of mechanoenergetic coupling toward normal. *p<0.05 *vs.* placebo and †P<0.05 vs. 3-day following MI, by ANOVA.

not elicit rejection, despite the absence of immunosuppressive drug therapy, was supported by the lack of a significant inflammatory response. (Note that cells surrounding vessel in Figure 1g and 1i are of MSC origin, as indicated by DAPI positivity in Figure 1h). The number of MSCs persisting in the myocardium decreased over time. Nonetheless, MSC injection produced a wide range of benefits, including improved regional and global ventricular function, reduced myocyte apoptosis, and improved tissue perfusion.

In terms of functional responses, anterior MI caused dramatic deterioration of systolic and diastolic ventricular function, and impaired cardiac energy metabolism ($p<0.05$ vs. pre-MI values). Compared with injection of placebo, MSC cardiomyoplasty resulted in profound improvements in myocardial function and efficiency (Figure 2). Figure 2a depicts representative examples of pressure-dimension data from animals in either group. As shown, MSC treatment led to a pattern of LV recovery over a 2-3 month period marked by a substantial increase in stroke work (SW, the area within the loops). In the placebo-treated group, impaired cardiac function evident 3 days post infarction either persisted or worsened over 8 weeks of follow-up: indices of myocardial contraction fell and end-diastolic pressure rose (Figure 2 a,b,c,d). In marked contrast, LV end diastolic pressure increased to normal 8 weeks after MSC treatment (*$p<0.05$ vs. placebo). MSCs caused myocardial performance to recover to normal, both in systolic (Ees rose to 13.9±2.7 mmHg/mm and peak +dP/dt to 2465±575 mmHg/sec) and diastolic function (Tau fell to□37±3.8 msec).

Heart failure and the aging cardiovascular system are characterized by mechanoenergetic uncoupling: decreased efficiency of work per unit oxygen consumption. In placebo-treated animals, SW decreased substantially during the 8-weeks following infarction, and there was a paradoxical increase in myocardial oxygen consumption, resulting in decreased ratio of SW/MVO_2. Conversely, MSC-injected animals' follow-up was marked by improving myocardial efficiency, both because of increasing SW (from 374.4±59.3 to 654.4±129.9 mmHg.mm at 8 weeks) and because of decreasing MVO_2 (from10.3±2 to 3.7±1.8 J/beat), both toward normal (Figure 2 e). Thus, MSC therapy exerts favorable effects on the damaged heart that extend to improvements in cellular energy metabolism. The SW/MVO_2 ratio increased from 2.5±0.6 at 3 days post-MI to a normal ratio of 10±5.6 ($p<0.05$ *vs.* placebo) at 4 weeks. This improvement in mechanoenergetics was the earliest observable benefit of MSC treatment, preceding changes in global cardiac function. Improved mechanoenergetic coupling in the MSC group is consistent with several possible mechanisms, including reduced native tissue

Figure 3. MRI image of swine myocardium obtained after myocardial infarction and injection of Feridex labeled mesenchymal stem cells. Feridex labeled cells can be seen as dark hypoenhancing regions in the epicardium (arrows) using an ECG-gated, fast gradient echo (fgre) pulse sequence. As shown, Feridex labeling remains evident for up to eight weeks after stem cell injection.

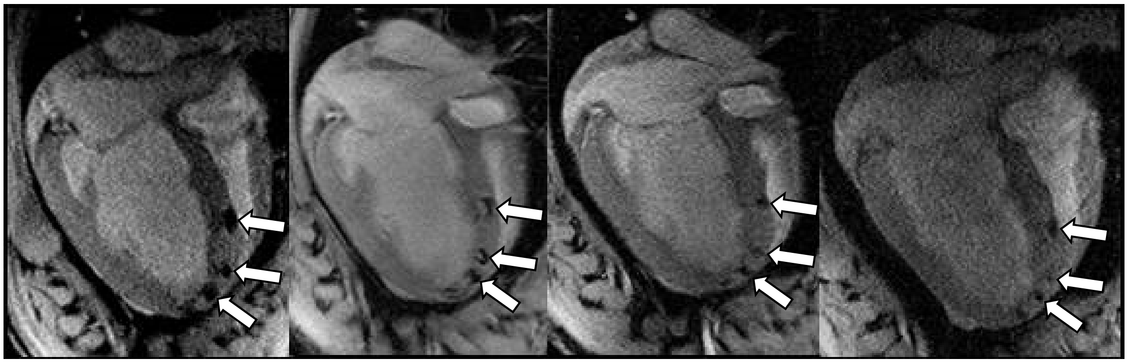

death[64], new tissue formation[42;65], or stimulation of endogenous repair mechanisms[66;67].

To further investigate the mechanisms of MSC-mediated cardiac repair, both MRI and computed tomography (CT) were used to image and quantify myocardial infarcts in MSC- and placebo-injected swine. Infarct size measurement *in vivo* by MRI and CT correlated tightly to that determined by triphenyltetrazolium chloride (TTC) staining post-mortem. Furthermore, using a 32 slice multidetector CT, the same endocardial rim of viable, non-infarcted myocardium observed in the first series of post-mortem hearts (Figure 3, Figure 4) was identified by *in vivo* imaging. These data not only speak to the therapeutic potential of MSC cardiomyoplasty, but also establish that noninvasive imaging techniques can be used to measure the effects of cardiomyoplasty, and to study the mechanisms underlying these effects. These results in this pig model provide strong rationale for the development of MSC-based cellular cardiomyoplasty strategies and support ongoing human studies.

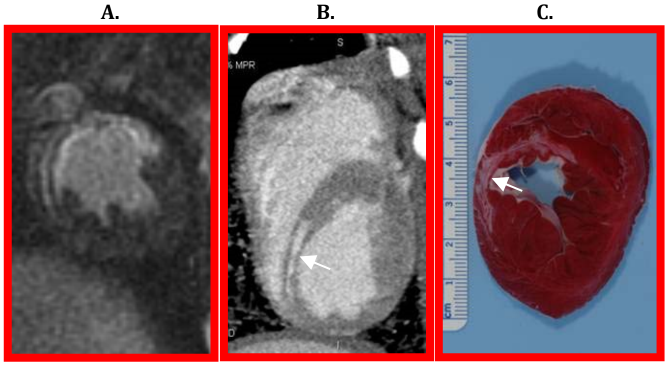

Figure 4. Comparison of infarct size using MRI (A), CT (B), and TTC (C). Images were obtained 8 weeks after closed-chest infarction in a pig and demonstrate subendocardial myocardial infarction as hyperenhancing region (~7-11 o'clock). TTC nonstaining areas (e.g., lack of brick red staining) in post-mortem slices (bottom) demonstrate concordance of infarct location and size with MRI and CT. Infarct region is notable for rim of noninfarcted myocardium along the endocardial border seen with CT and TTC staining. (arrows)

MSCs injected intravenously home to and engraft in infarcted myocardium conferring functional benefit: Preliminary studies were conducted on the efficacy of MSCs administered intravenously (*I.V.*) in a rat model of permanent left anterior descending (LAD) artery occlusion. Echocardiography was used to assess LV function at baseline, in the peri-infarct period, and four weeks after MI. MSC injection in Wistar rats led to dramatic improvement in LV function, with increased

myocardial thickening and contractility in treated animals (Figure 5A and 5B). Labeled cells were identified within the infarct (Figure 6a), and were shaped like fibroblasts but expressed the cardiac protein, α-actinin, albeit at lower levels than native cardiomyocytes (Figure 6b). These labeled cells were most evident at the endocardial rim of the infarct, a finding similar to that seen in the porcine studies above.

MSCs delivered intravenously (I.V.) distributed to the heart in response to an injury signal: MSCs injected *I.V.* at the time of coronary reperfusion homed to the myocardium, while cells injected *I.V.* two weeks after reperfusion were more likely to engraft in the bone marrow (Figure 7). Determination of SDF-1 and CXCR4 levels revealed not only that both are expressed by MSCs, but also that serum levels are up regulated immediately post infarct and remain elevated for at least 2 weeks (Figure 8).

Myocardial Function can be Determined *in vivo* by MRI

The clinical research team has extensive experience using tagged MRI scanning to detect and quantify alterations in regional myocardial mechanics in animal models of ischemic heart disease[68;69]. The team has also developed non-surgical, MRI-compatible animal models for studying cardiac mechanics, perfusion, and interventional procedures[70-73]. The team has recently developed new MR imaging and analysis methods that enable the rapid determination of myocardial function in infarction and ischemia[70]. This new technique, Harmonic Phase (HARP) MRI[74;75] is based on tagged MRI techniques. Computationally, the analysis of HARP MRI can be performed much more rapidly than traditional tag tracking in tagged MRI. “Real-time” HARP imaging exploits the concept that only one of the spectral peaks must be acquired for motion in one direction; this in turn accelerates the image acquisition in addition to the rapid analysis already available in HARP. An example of the use of real-time HARP to monitor the onset of ischemia in a canine model of coronary artery stenosis is depicted (Figure 9)[104]. Using this technique, we can identify abnormal regional myocardial contraction after the onset of ischemia 20 seconds earlier than can be done using conventional cine wall motion studies and one minute earlier than ECG changes. HARP MRI, similar to tagged MRI, yields quantitative motion and strain parameters on a regional basis that can be used for comparison across patients or at serial time points after intervention. Thus, HARP MRI and analysis represents a rapid and repeatable method to assess left ventricular function serially in a quantitative manner. HARP provides fast, accurate assessment of myocardial strains in humans with and without coronary artery disease[76]. Similar techniques have been used to assess structural and functional changes after myocardial infarction in rats[77;78].

Noninvasive Determination of Infarct Size

Using an intravenous injection of Gd-DTPA, the study team is able to determine infarct size non-invasively with T1-weighted contrast-enhanced MRI (i.e., “Delayed Contrast-Enhanced MRI”). Short-axis image slices, which span the entire left ventricle, can be obtained using multiple breath-holds to yield images with the highest spatial resolution. The size of the area of hyper enhancement measured on such images has been shown to be within 10% of the infarct size measured by post-mortem TTC staining (Figure 10). Alternately, using new imaging techniques, one can obtain the entire 3D left ventricular volume in a single breath hold (~16 heartbeats). It was recently shown that the 3D

technique is in concordance with infarct size as measured by traditional 2D multi-slice techniques (Figure 10)[89,90]. Thus, infarct size and location can be accurately determined in less than one minute of scanning time, in order to determine the size of an infarct prior to therapy and over time.

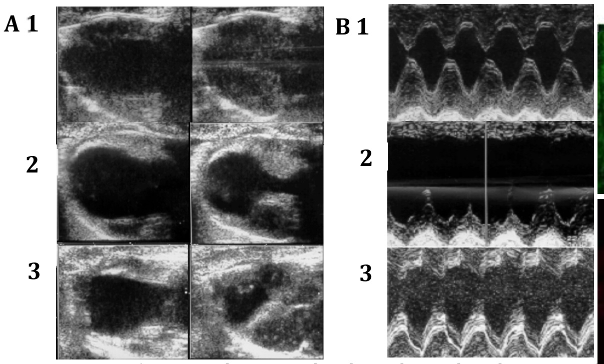

Figure 5.A: Two-dimensional echocardiography showing end diastole (left column) and end systole (right column) in a treated rat 1) before infarction 2) after infarction and prior to treatment and 3) 4 weeks after treatment with MSCs. **B:** M-Mode from same animal showing fractional shortening at the papillary level at the same time points as in A.

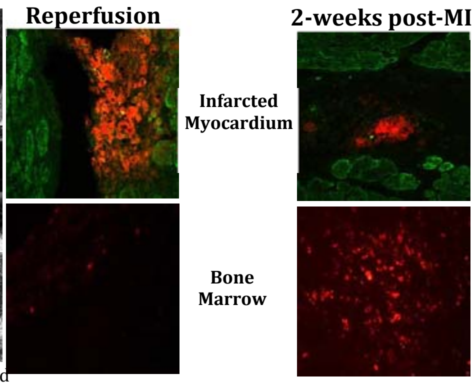

Figure 7. Confocal images of infarcted myocardium (top row) and bone marrow (bottom row) illustrating the degree of allogeneic MSC engraftment observed when 5 million MSCs were delivered via tail vein at reperfusion (left columns) or 2 weeks post-reperfusion (right column). As shown, at reperfusion cells home to the heart and not bone marrow; two weeks later, trafficking to the heart is reduced, and cells now migrate and engraft in the bone marrow. All tissues were harvested 4 weeks post-implantation. A FITC-conjugated antibody directed against desmin (green) was used to assess the myogenic differentiation.

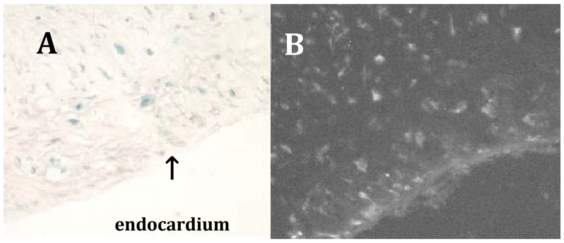

Figure 6: A: β-galactosidase positive (blue) cells are visible at 20X magnification within the infarct in young rats. These cells form a band along the endocardial surface. **B:** These cells show evidence of α-actinin expression on immunofluorescence, also at 20X magnification (bright appearing cells, B&W image). **C:** Quantification demonstrates 10-fold higher engraftment in young relative to old ($p<0.001$).

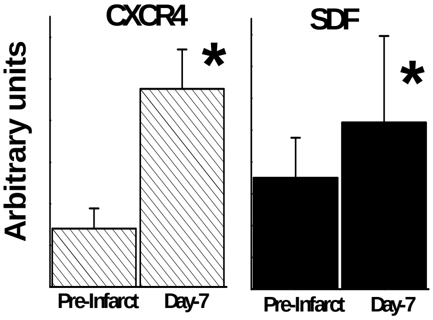

Figure 8: Both SDF-1 and CXCR4 levels are elevated following myocardial infarction. $P<0.05$ vs. pre infarct

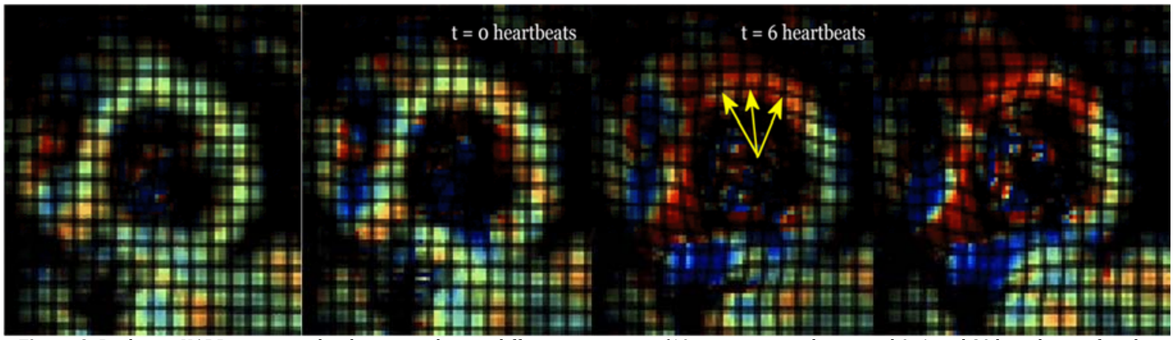

Figure 9: Real-time HARP images in the short-axis plane at different time points (10 sec prior to ischemia and 0, 6, and 20 heartbeats after the onset of ischemia from left to right) in a canine closed-chest model of acute LAD coronary artery occlusion. Overlaid on the tagged images is a pseudo-color map of circumferential shortening where green is uniform shortening, red is decreased shortening or stretching, and blue is increased shortening. At 6 heartbeats after ischemic insult, stretching of the ischemic myocardium is observed in the LAD bed whereas wall motion abnormalities by cine MRI could not be appreciated until 30 sec post-occlusion.

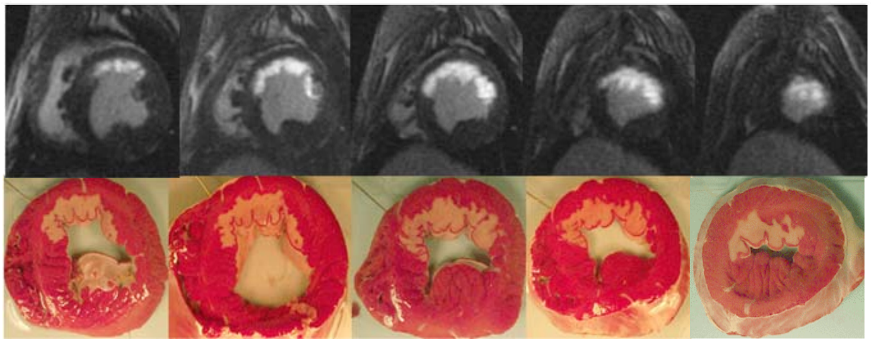

Figure 10. 2D delayed CE MRI in short axis plane (top) acquired within the first 24 hrs after closed-chest infarction demonstrating subendocardial myocardial infarction as hyperenhancing region (~11-1 o'clock) in a dog. TTC nonstaining areas (e.g., lack of brick red staining) in post-mortem slices (bottom) demonstrate concordance of infarct location and size with MRI.

Autologous Mesenchymal Stem Cells Produce Reverse Remodeling in Chronic Ischemic Cardiomyopathy: In addition to the studies outlined above using models of acute MI in the pig, we have also developed a model of chronic MI in the Gottingen mini-swine. We have used both autologous and allogeneic MSCs, with surgical and catheter delivery strategies, and have developed sufficient experience to translate the therapy from the laboratory bench to clinical trials. Together our results indicate that bone marrow derived MSCs stimulate cardiac recovery by engrafting, forming new blood vessels that increase tissue perfusion in hypoperfused areas, forming new cardiac myocytes, and importantly interacting with endogenous precursor cells to also contribute to new cardiac myocyte formation. From an immunologic perspective, MSCs may be safely used as an allogeneic graft, and have been done so extensively in clinical trials[79-81].

Our experience with a 53 patient, 10-center, phase I study under the sponsorship of Osiris therapeutics, which demonstrated safety and provisional efficacy of allogeneic MSC therapy in patients with acute infarction is outlined below[17]. In animal studies conducted in mini-swine, MSC injection via catheter into infarcted tissue reduces myocardial infarct size (Figs. 11 and 12), improves global and regional LV function, normalizes cardiac energetics, and restores tissue perfusion[63;82]. These results form the basis for our approval to conduct the CRATUS study.

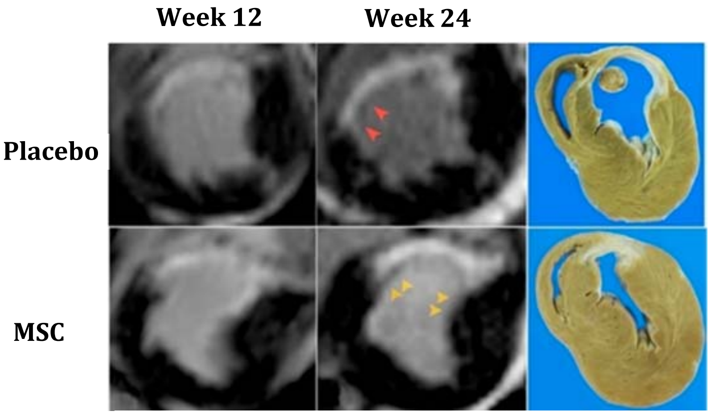

Figure 11. Myocardial regeneration in our porcine model of MI. Delayed gadolinium-enhanced MRI images depicting chronic (week 12 post MI) myocardial scar before treatment and (week 24) 12 weeks following injection of MSCs or placebo. Infarct tissue appears bright white, and healthy myocardium appears black. Comparable gross heart sections are shown adjacent to the MR images. Infarct size is reduced by MSC treatment, and cardiac performance improves.

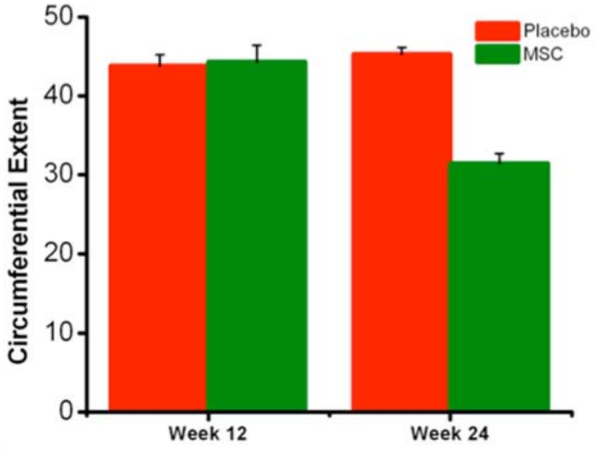

Figure 12. Infarct circumferential extent of the LV before and 12 weeks after injection in swine. Infarct size was significantly reduced by MSC treatment. $p<0.05$ MSCvs. placebo, $p<0.05$ MSC week 12 vs. week 24. [n=10]

Our work in large animal models with fully healed scars after MI showed that MSC administration can significantly improve left ventricular structural and functional indices, indicating meaningful repair. Using sophisticated imaging techniques we tracked phenotypic improvements triggered by implantation of MSCs in a porcine model of chronic ischemic cardiomyopathy and quantified these changes morphometrically. MI was created in swine; after 12 weeks, the infarct segment had thinned, leaving a transmural scar (Figure 11). Autologous MSCs were expanded from each animal[83], and these cells or placebo were delivered to the infarct and surrounding border zone at this time. During

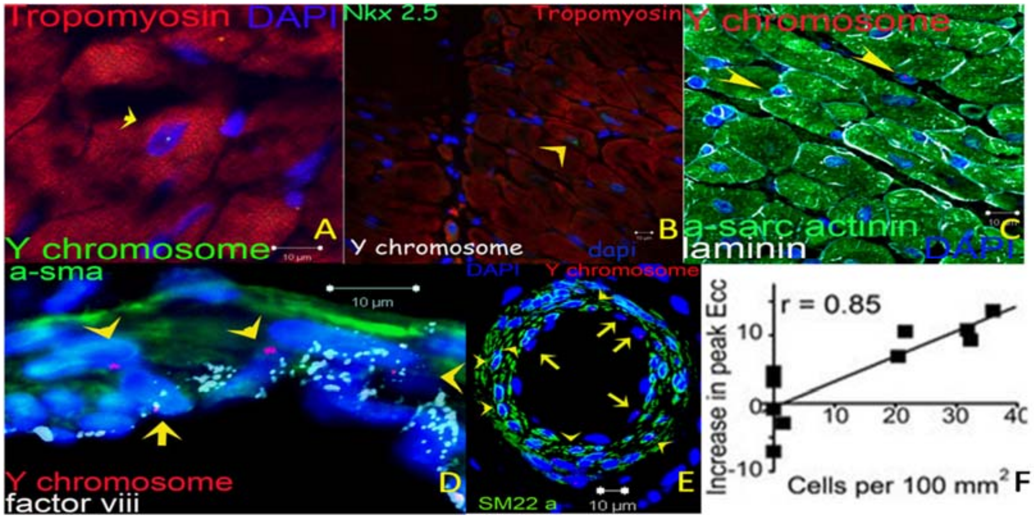

a further 12-week follow up period, cardiac MRI revealed that intramyocardial injections of MSCs not only reduced the scar burden (as a percentage of LV mass) by 21.8±3.9% ($p<0.05$ vs. placebo and week 12 vs. week 24) (Figure 11, 12), but also significantly improved regional contractility, global LV function, ejection fraction, and myocardial blood flow. Importantly, the therapy produced reverse remodeling and reduced the circumferential extent of the infarct scar (Figure 12). This constellation of effects suggests highly effective repair of ischemic cardiomyopathy. We subsequently confirmed reverse remodeling in a pilot study of 8 patients with ischemic cardiomyopathy (Figure 21)[43].

Figure 13. Engraftment of MSCs in chronic myocardial infarction. (**A**) Colocalization Y chromosome and troponin, indicative of an MSC differentiated into a cardiomyocyte. (**B**) Evidence of cardiac commitment in the transplanted cardiomyocyte by the colocalization of cardiac transcription factor Nkx2.5 (green, arrow). (**C**) Ypos cells also reside in the interstitial compartment (arrows) of border myocardium in a non-differentiated stage. (**D**) Ypos cells that colocalize with smooth muscle actinin (arrowheads) and factor viii-related antigen (white, arrows) demonstrating vascular smooth muscle and endothelial commitment, respectively. (**E**) Confirmation of Ypos cells commitment into vascular structures as depicted by co-localization with SM22-alpha (**F**) The functional outcomes (i.e. infarct size reduction, increase in

contractility and increase in tissue perfusion) showed related interaction with the magnitude of transplanted cells detected.

Allogeneic Mesenchymal Stem Cells Restore Cardiac Function in Chronic Ischemic Cardiomyopathy Via Trilineage Differentiating Capacity:

We tested the hypothesis that MSC based cardiac repair regenerates the heart via mechanisms comprising long-term engraftment and by differentiation into both myocardial and vascular elements. We generated allogeneic MSCs from a male swine donor, and administered sex mismatched cells by transendocardial injection into female swine 12 weeks post-MI. Animals were followed with serial MRI, and 12 weeks later the hearts were collected for immunohistological evaluation. The fate of the male donor cells was determined by co-localization of Y-chromosome (Y^{pos}) cells with markers of cardiac, vascular, and endothelial lineages. MSCs engrafted in infarct and border zones and differentiated into cardiomyocytes (Figure 14) as ascertained by co-localization with GATA-4, Nkx2.5, and α-sarcomeric actin markers. In addition, Y^{pos}MSCs exhibited vascular smooth muscle and endothelial cell differentiation, contributing to large and small vessel formation. The number of cells engrafting correlated with the functional changes that occurred (Figure 14F). Thus, MSCs could engraft and repair hearts in chronic ischemic cardiomyopathy[84].

Ventricular remodeling is a progressive disease causing the myocardium to reorganize itself from an elliptical to a spherical shape. During its reorganization the heart will become enlarged and ventricular dimensions increase, assisting in reshaping a patient's heart.In swine models, MSCs show that the reduction in scar size supports reverse remodeling, which show a reduction in scar size as quickly as 3 days after injection. MSCs have shown as a promising cell-based therapy where previously there was limited means of improving a patient's heart function and survival[85].

Figure 14. Merging CMR angiography and electroanatomical mapping to guide transendocardial mesenchymal stem cell injection.

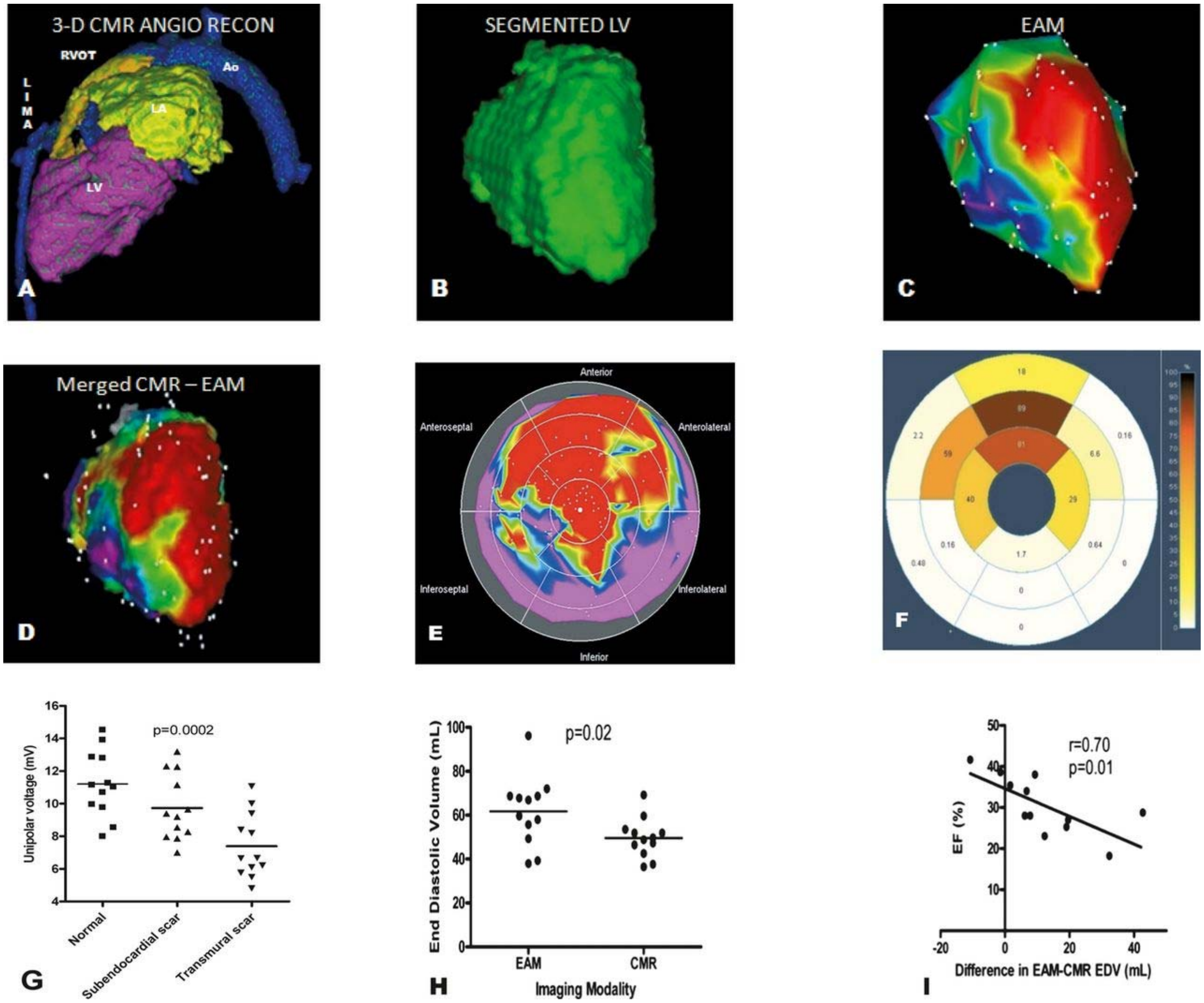

Figure 15: Durable and progressive scar size reduction due to intramyocardial injection of allogeneic bone marrow MSCs. A, Delayed-enhancement CMR scar size images show durable reduction in scar size with MSC therapy.

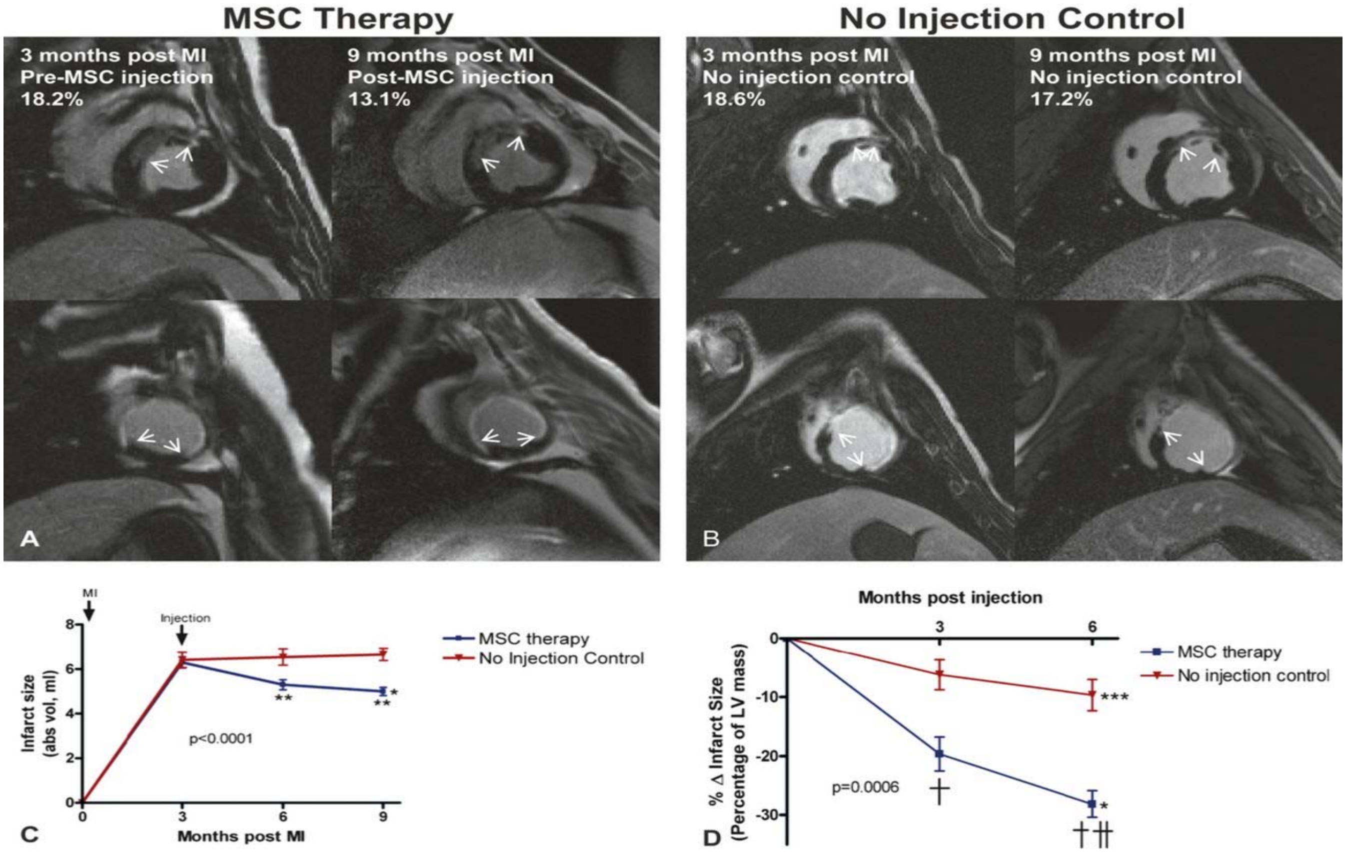

Figure 16: Allogeneic MSC therapy reverses remodeling in ischemic cardiomyopathy.

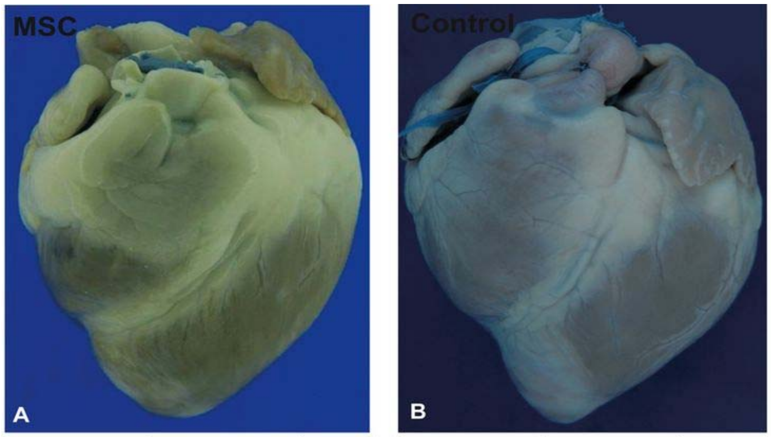

	3 mo	9 mo
EDV (mL)	37.6	37.7
ESV (mL)	24.3	19.3
Infarct size (% LV mass)	21.1	14.5

	3 mo	9mo
EDV (mL)	38.6	52.9
ESV (mL)	26.3	39.3
Infarct size (% LV mass)	20.3	18.2

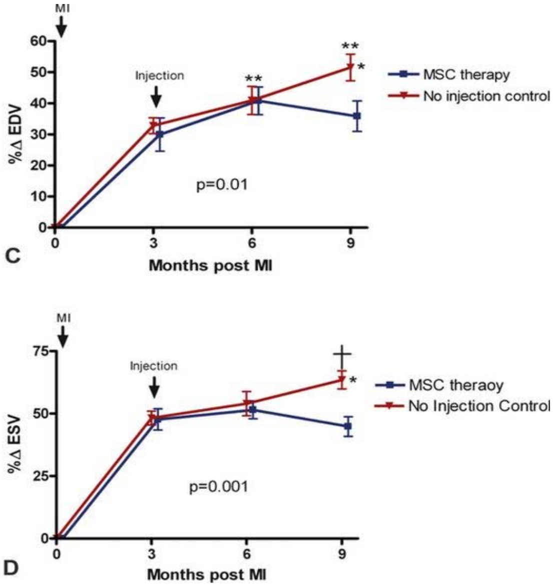

Figure 17: Allogeneic MSC therapy improves LV sphericity index.

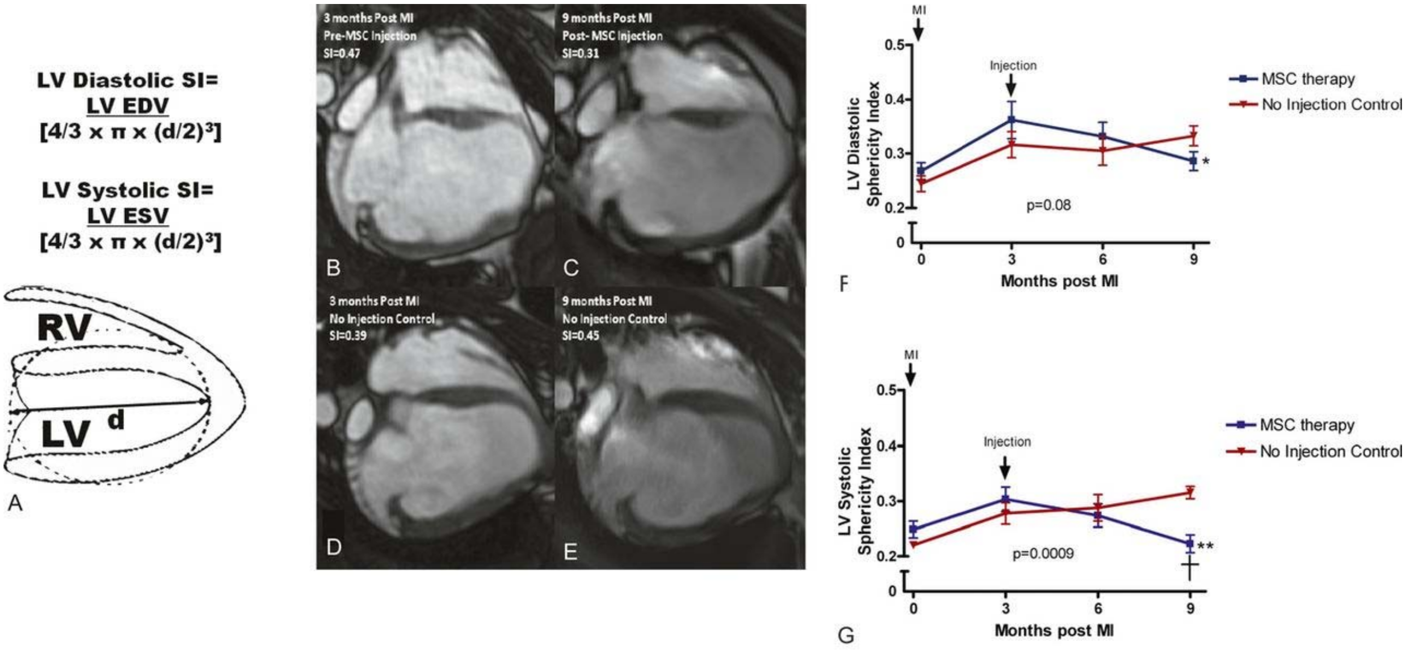

Figure 18. Progressive improvement in LV function with allogeneic MSC therapy.

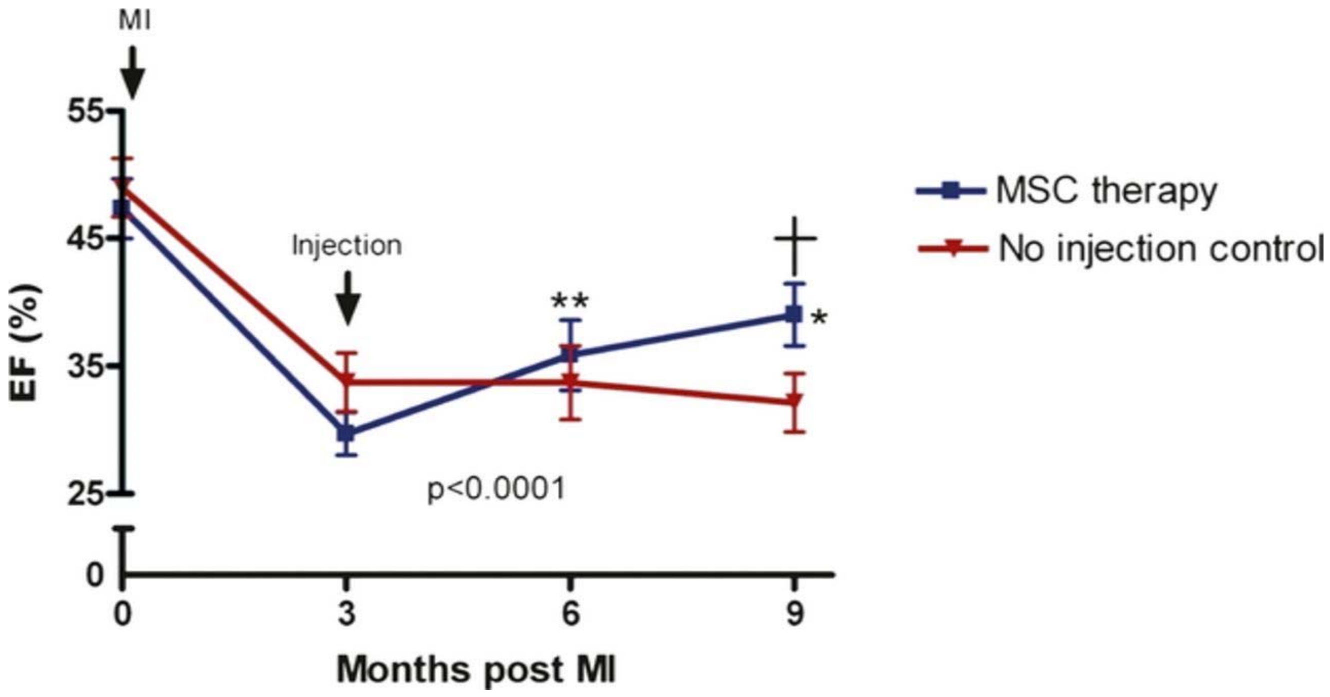

1.4 Allogeneic Mesenchymal Stem Cells: Previous Experience in Humans

The use of allogeneic cellular products typically requires matching of the graft HLA to the donor in order to avoid graft rejection and graft versus host disease. However, because MSCs do not express HLA, they represent a unique immunopriveleged cell population which can be used for allogeneic cellular therapy. In addition, MSCs fail to induce proliferation of allogeneic lymphocytes *in vitro* and suppress proliferation of T cells activated by allogeneic cells or mitogens. MSCs have also been shown to exert anti-proliferative, immunomodulatory and anti-inflammatory effects. Many patients have received allogeneic MSCs and infusions have all been well tolerated.

A multi-center, randomized, double-blind, placebo-controlled study was performed to evaluate the safety and preliminary efficacy of allogeneic MSCs administered after myocardial infarction[17]. In this study, 53 patients were treated with one of three cell-dose levels of allogeneic MSCs (0.5, 1.6 and 5.0 cells/kg body weight) or placebo administered intravenously. No HLA matching was performed in this study and administration was found to be safe and well tolerated at all dose levels (with 5.3 adverse events per patient in the MSC-treated group vs. 7.0 in the placebo group). No deaths were reported and no serious adverse events were attributed to MSC administration. Improvements were seen in patients receiving MSCs as compared with those receiving placebo in the frequency of arrhythmic events and premature ventricular contractions, post-event ejection fraction for patients with major anterior wall infarctions, overall clinical status, and notably post-infusion pulmonary function as measured by FEV1 percent predicted (increased 17% in the MSC-treated group vs. 6% for placebo group, $p < 0.05$).

Allogeneic MSC infusion has also been studied in a phase II trial of MSCs for the treatment of severe acute graft versus host disease. In this study, 55 patients received 1-5 intravenous infusions of 1.4 x 10^6 cells/kg body weight from HLA matched and mis-matched donors. A complete response was seen in 30 patients and improvement was seen in 9 patients. Of note, response rates were not associated with HLA-matching. No infusion related side effects were noted and no long-term adverse events were observed.

The safety and efficacy of allogeneic MSCs for the treatment of refractory lupus has also been explored[86]. Fifteen patients received a single intravenous infusion of 1 x 10^6 cells/kg body weight. MSCs were derived from family members but were not HLA-matched. At 12 months, all patients had improvement in disease activity as measured by 24 hour proteinuria (decreased from 2505.0±1323.9 to 858.0±800.7 mg/24hr, $p<0.05$) and SLE Disease Activity Index scores (decreased from 12.2±3.3 to 3.2±2.8, $p<0.05$). No serious adverse events were noted in any of the patients.

The Poseidon trial (IND #13568; NCT01087996), has provided additional validity that MSCs reduce infarct size and reverse remodeling of the myocardium[85]. Study data also reveal that MSCs are immunoprivileged and immunosuppressive, and do not cause acute immunogenic reactions. In this regard, no patient reported symptoms or indications of a reaction. Only one of 15 patients receiving ahMSCs mounted a donor specific alloreaction, of low antibody titer[18]. Furthermore, patients treated with allogeneic MSCs suffered fewer AE's versus autologous MSCs patients. It is also hypothesized that the function of autologous MSCs could be impaired in patients with co-mobility or advanced age[87;88]. Reverse cardiac remodeling was a prominent factor patients showed as assessed by the study CT scan results[18]. Allogeneic MSCs were shown in this trial to be a safer and more effective form of cell-therapy than autologous MSCs. Figures 19 and 20 depict functional and quality of life improvements in patients received both allogeneic and autologous MSCs.

We randomized 30 patients in an open-label study of either allogeneic or autologous hMSCs at 3 doses; 20, 100, or 200 million total hMSCs. The study was designed as a pilot study to compare the safety and efficacy of allogeneic versus autologous hMSCs in patients with chronic ischemic left ventricular dysfunction secondary to myocardial infarction. Autologous MSCs were derived from a sample of the patient's bone marrow approximately 4-6 weeks prior to cardiac catheterization. Allogeneic MSCs were supplied from a human MSC source manufactured by the University of Miami. All 30 patients tolerated the procedure well and have been followed-up with serial cardiac CT at 13 months post injection. The serious adverse event rate (SAE) was lower among patients treated with allogeneic MSCs as compared to autologous MSCs (6-months: 20%-allogeneic MSCs and 40%-autologous MSCs). The injection of MSCs into the hearts of these patients produced reverse remodeling, with reductions in EDV of ~23 ml and reduction in spherecity index of 0.8.

Together, these preliminary data support our hypothesis that allogeneic MSC administration is a safe and successful option for cellular cardiomyoplasty. We have demonstrated long-term MSC survival, engraftment, and differentiation into myocardial,

vascular, and endothelial lineages following transplantation into chronically scarred porcine myocardium. These cells' capacity for cardiomyogenesis and vasculogenesis both likely contribute to their ability to repair chronically scarred myocardium.

Figure 19: Functional Outcomes

A) Six-Minute Walk Test (meters)

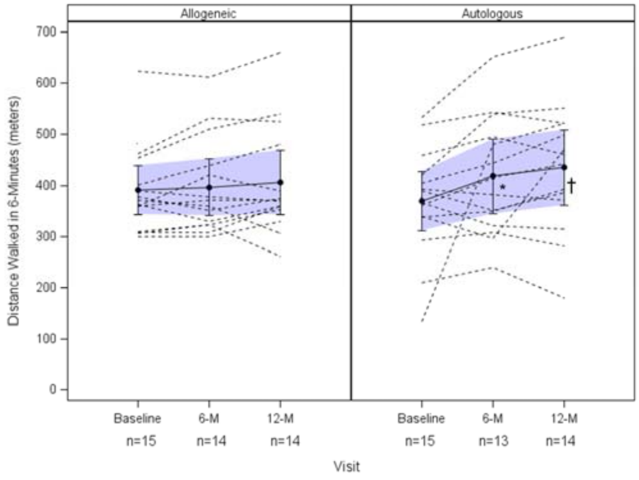

B) Peak VO_2 (ml/kg/min)

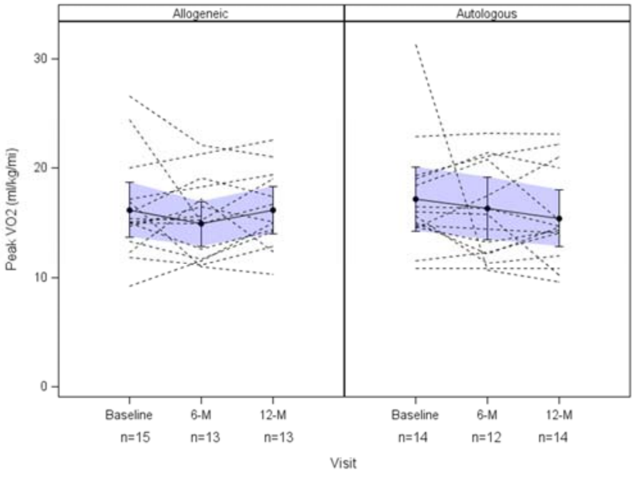

C) Minnesota Living with Heart Failure

D) New York Heart Association Class

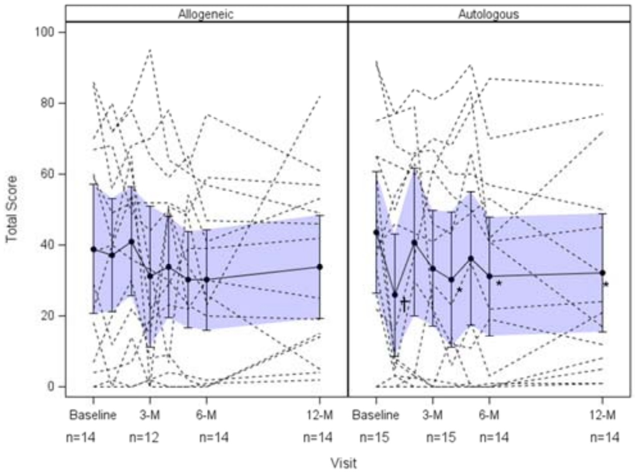

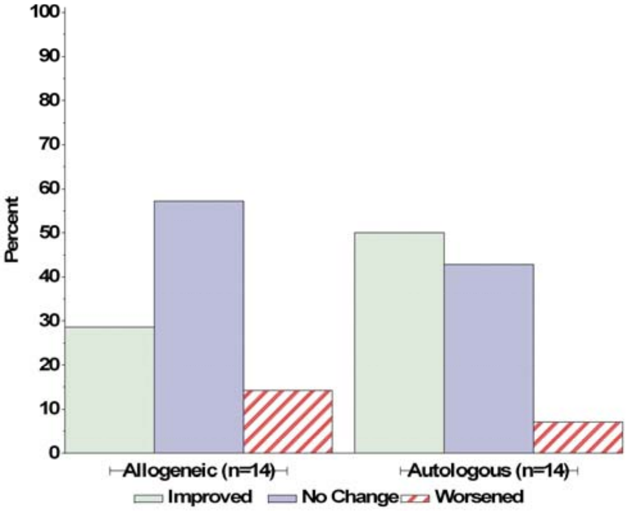

Figure 20: CT Parameters Change from Baseline

A) Left Ventricular Ejection Fraction (%)

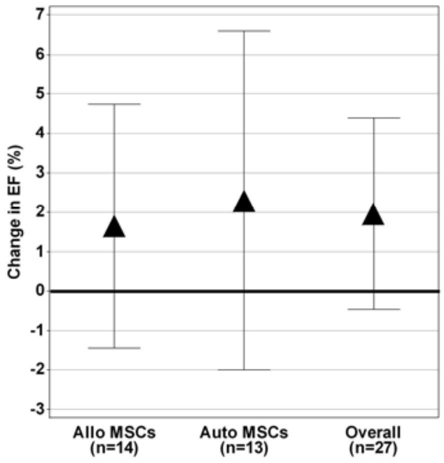

B) End Diastolic Volume (ml)

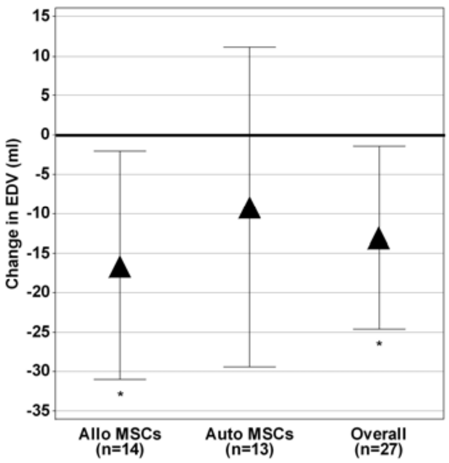

C) End Systolic Volume (ml)

D) End Diastolic Myocardial Volume (cm^3)

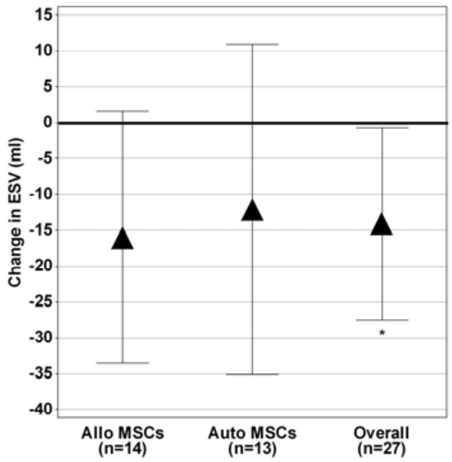

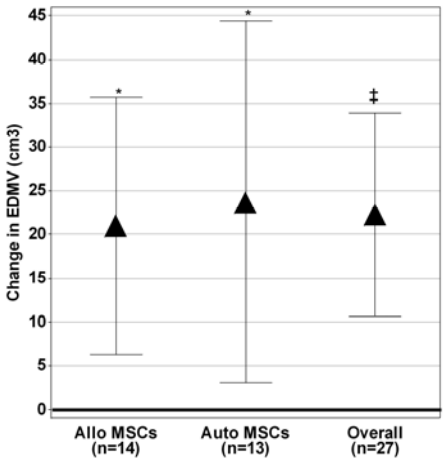

E) Sphericity Index

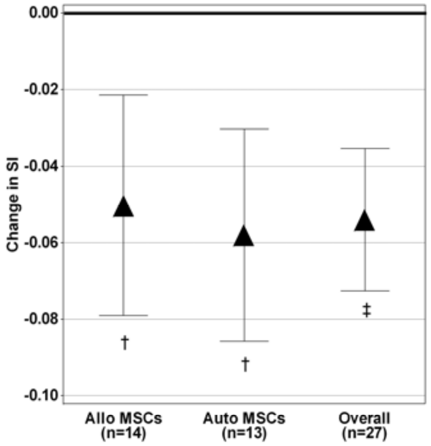

F) MI Size (Early enhancement defect; grams)

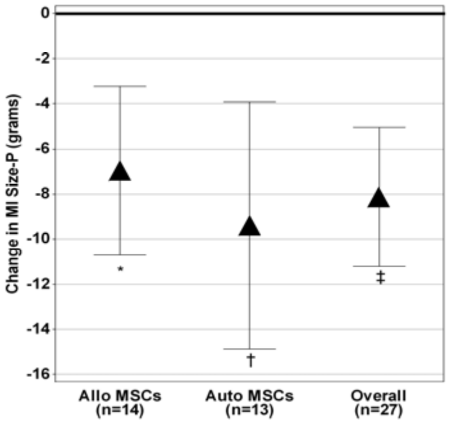

G)

 CONFIDENTIAL AND PROPRIETARY

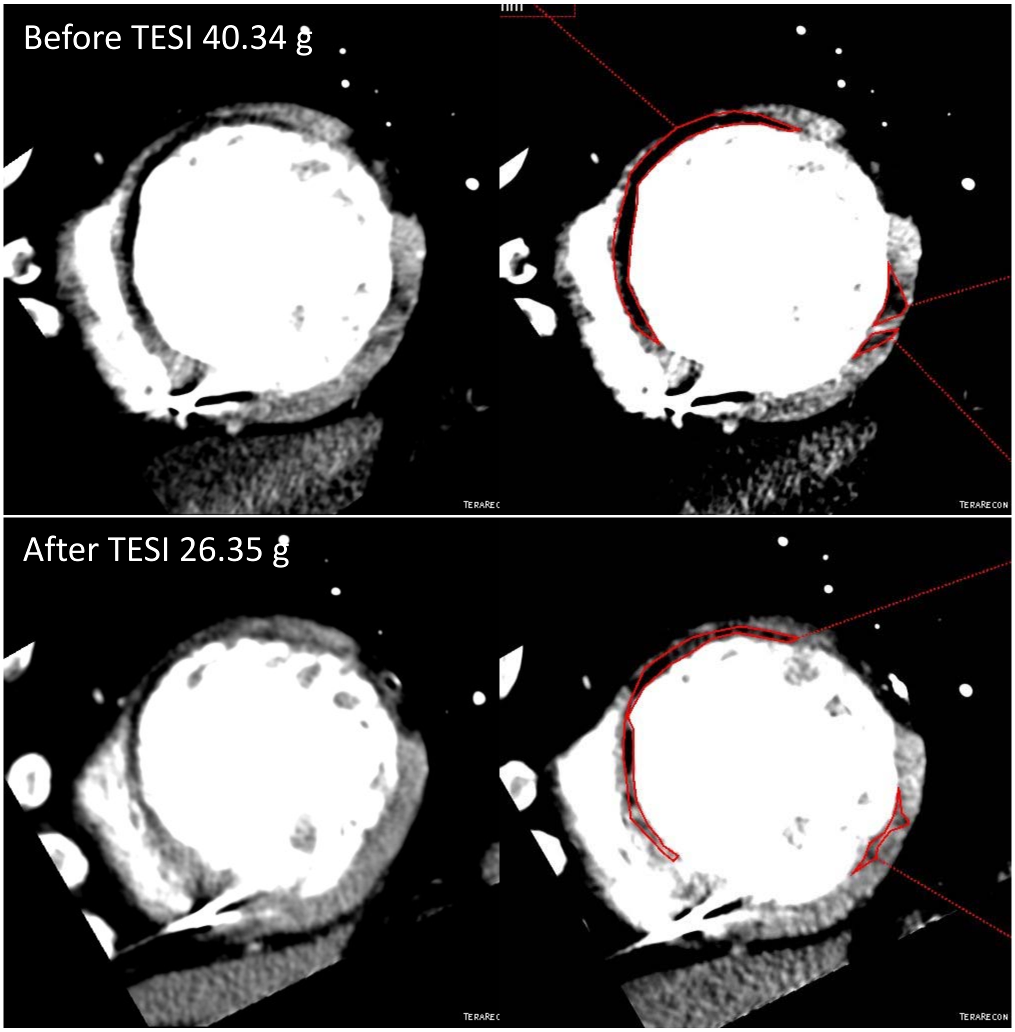

The study by Weiss et al, which investigated ahMSC therapy for COPD, reflected similar trial results indicating as the Poseidon trial that allogeneic MSCs did not cause acute immuogeneic reactions in patients participating in the trial. Importantly, ahMSCs produced a decrease in the inflammatory marker CRP. Systemic administration of MSCs appears to be safe and decrease inflammation in an older and co-morbid population of patients with compromised lung function due to moderate COPD[19].

Preliminary Results from the Transendocardial Autologous Cells in Ischemic Heart Failure Trial (TAC-HFT): We enrolled 8 patients in an open-label run-in phase of the TAC-HFT trial to assess the safety and preliminary efficacy of bone marrow mononuclear cells (MNCs) and MSCs in patients with ischemic cardiomyopathy[24, 25]. At baseline each patient underwent a bone marrow aspiration and transendocardial injection (Helix Infusion Catheter; Biocardia, Inc., CA) of bone marrow derived MNCs or MSCs to the infarct and border zone using biplane fluoroscopic guidance. All 8 patients tolerated the procedure well and have been followed-up with serial cardiac MRI at 3, 6, and 12 months post injection (Figure 21). The injection of bone marrow derived cells into the hearts of these patients produced reverse remodeling, with reductions in EDV of ~12% and improved regional function as measured by –Ecc, a cardiac MRI derived index (Figure 21). As

described above, we have also treated patients with ahMSCs in the POSEIDON trial and have demonstrated an outstanding safety profile.

A.

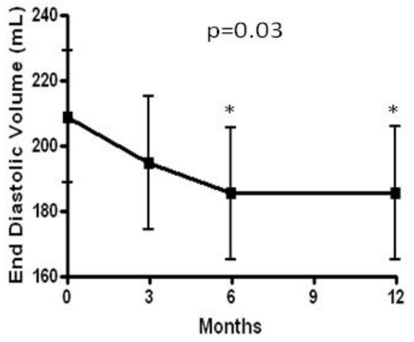

B.

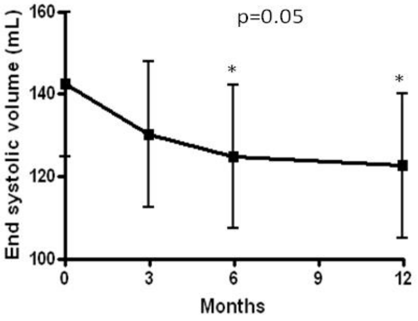

C.

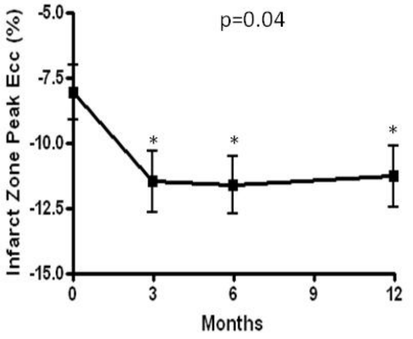

D.

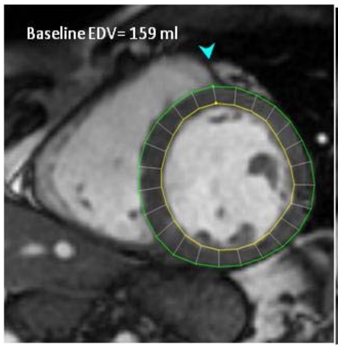

E.

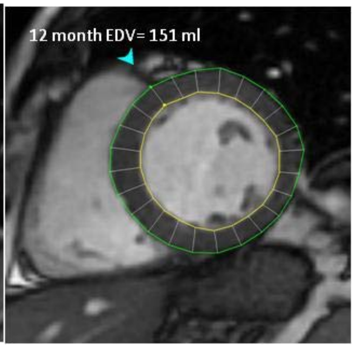

F.

G.

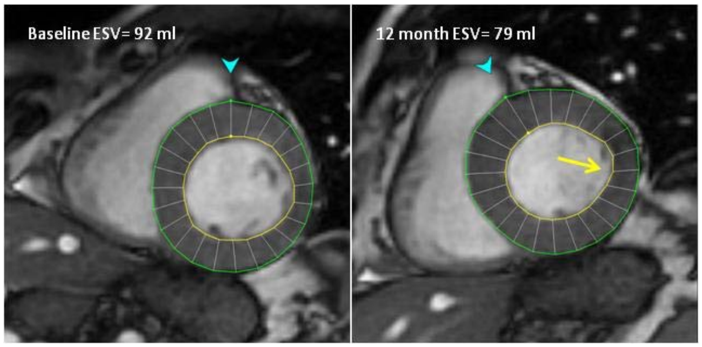

Figure 21. Cardiac MRI follow-up data of (A) end-diastolic volume, (B) end-systolic volume, and (C) peak Eulerian circumferential strain (Ecc) of the infarct zone from tagged imaging. Example cardiac MRI images of change in EDV from (D) baseline to (E) 1 year, and change in ESV from (F) baseline to (G) 1 year after stem cell injection. As depicted, EDV and ESV are reduced by 6 months following injection, and do not return towards baseline by 12 months. Peak Ecc is dramatically reduced by 3 months following injection (the more negative Ecc corresponds to *improving* regional LV function). *$p<0.05$ in post-hoc analysis compared to baseline. Yellow Arrow: Note improved systolic thickening in lateral wall, the site of cell therapy in this example.

1.5. Pharmacology and Toxicology Studies of Mesenchymal Stem Cells

Preclinical data suggest that MSCs may safely be used in the treatment of Frailty. Using the murine bleomycin model, several groups have shown that the administration of stem cells ameliorates bleomycin induced lung injury with no significant adverse effects.

Lee et al intravenously administered $1x10^6$ bone marrow derived MSCs to rats treated with bleomycin and found a decrease in bleomycin induced lung edema, neutrophil infiltration, collagen deposition, and overall mortality with no adverse effects reported[55]. Similarly, Ortiz et al intravenously administered 5×10^5 bone marrow derived MSCs to mice with no adverse effects reported. They found that after bleomycin exposure, MSCs home to sites of lung injury, lead to decreased fibrosis and extracellular matrix collagen deposition, and contribute to tissue repair[51]. Rojas et al intravenously administered 5×10^5 allogeneic bone marrow derived MSCs to mice treated with bleomycin and observed that lung injury attracts bone marrow derived MSCs via the production of soluble factors like G-CSF and GM-CSF which lead to MSC proliferation and migration and ultimately improved survival with no reported adverse effects[56].

These findings appear to extend beyond bone marrow derived MSCs. Cargnoni et al administered fetal membrane derived cells (both allogeneic murine and xenogeneic human) to bleomycin treated mice and found that placental derived stem cells, like MSCs, localize to the lung and reduce tissue damage associated with bleomycin exposure regardless of source or route of administration (intravenous, intraperitoneal, or intratracheal)[50]. They did find that intraperitoneal and intratracheal administration of cells led to mild to moderate lung inflammation but no fibrosis in the absence of bleomycin injury. Importantly, this was not seen with intravenous administration of cells. In another murine model, xenogeneic human umbilical cord derived MSCs were also shown to home to sites of bleomycin induced lung injury, inhibit the production of pro-inflammatory cytokines, and reduce lung injury and collagen deposition with no adverse effects reported[52].

Table 1. Results of preclinical animal studies of mesenchymal stem cell therapy for IPF.

Study	Model	Cell Type	Cell Delivery and Dose	Safety Results	Efficacy Results
Lee at al, 2006	Rat	Allogeneic<br>BM-MSCs	Intravenous, 1 x 10^6 cells	No adverse effects reported.	Reduced edema, neutrophil infiltratration, collagen deposition.<br>Improved survival.
Ortiz et al, 2003	Mouse	Allogeneic<br>BM-MSCs	Intravenous, 5 x 10^5 cells	No adverse effects reported.	Reduced inflammation and collagen deposition.
Rojas et al, 2005	Mouse	Allogeneic<br>BM-MSCs	Intravenous, 5 x 10^5 cells	No adverse effects reported.	Reduced pro-inflammatory cytokines.<br>Improved survival.
Cargnoni et al, 2009	Mouse	Fetal membrane derived<br>(Allogeneic murine and xenogeneic human)	Intra-peritoneal,<br>4 x 10^6 cells<br>Intratracheal, 1 x 10^6 cells<br>Intravenous, 1 x 10^6 cells	Mild to moderate lung inflammation but no fibrosis induced by intraperitoneal or intratracheal administration.<br>No adverse effects reported with I.V. administration.	Decreased neutrophil infiltration and fibrosis regardless of route of administration or source of cells.
Moodley et al, 2009	Mouse	Xenogeneic human umbilical cord derived MSCs	Intravenous, 1 x 10^6 cells	No adverse effects reported.	Reduced inflammation, pro-inflammatory cytokine production, and collagen deposition.

In addition to safety data from preclinical animal studies, many patients have received allogeneic MSCs in clinical trials and infusions have all been well tolerated.

A multi-center, randomized, double-blind, placebo-controlled study was performed to evaluate the safety and preliminary efficacy of allogeneic MSCs administered after myocardial infarction[17]. In this study, 53 patients were treated with one of three cell-dose levels of allogeneic MSCs (0.5, 1.6 and 5.0 cells/kg body weight) or placebo administered intravenously. No HLA matching was performed in this study and administration was

found to be safe and well tolerated at all dose levels (with 5.3 adverse events per patient in the MSC-treated group vs. 7.0 in the placebo group). No deaths were reported and no serious adverse events were attributed to MSC administration. Improvements were seen in patients receiving MSCs as compared with those receiving placebo in the frequency of arrhythmic events and premature ventricular contractions, post-event ejection fraction for patients with major anterior wall infarctions, overall clinical status, and notably post-infusion pulmonary function as measured by FEV1 percent predicted (increased 17% in the MSC-treated group vs. 6% for placebo group, $p < 0.05$).

Allogeneic MSC infusion has also been studied in a phase II trial of MSCs for the treatment of severe acute graft versus host disease[31]. In this study, 55 patients received 1-5 intravenous infusions of 1.4×10^6 cells/kg body weight from HLA matched and mismatched donors. A complete response was seen in 30 patients and improvement was seen in 9 patients. Of note, response rates were not associated with HLA-matching. No infusion related side effects were noted and no long term adverse events were observed.

The safety and efficacy of allogeneic MSCs for the treatment of refractory lupus has also been explored[86]. Fifteen patients received a single intravenous infusion of 1×10^6 cells/kg body weight. MSCs were derived from family members but were not HLA-matched. At 12 months, all patients had improvement in disease activity as measured by 24 hour proteinuria (decreased from 2505.0±1323.9 to 858.0±800.7 mg/24hr, $p<0.05$) and SLE Disease Activity Index scores (decreased from 12.2±3.3 to 3.2±2.8, $p<0.05$). No serious adverse events were noted in any of the patients.

2.1.2. Secondary Objectives

- To explore effects of allo-hMSCs on symptom related quality of life, cardiovascular performance, and inflammation.

1.5 Study Endpoints

1.5.1 Primary Endpoints (Safety)

- Safety (Primary): Incidence (at one month post infusion) of any treatment-emergent serious adverse events (TE-SAEs), defined as the composite of: death, non-fatal pulmonary embolism, stroke, hospitalization for worsening dyspnea and clinically significant laboratory test abnormalities.

  - Serum chemistry: sodium, potassium, chloride, bicarbonate, BUN, creatinine, glucose, calcium, phosphate, AST/SGOT, ALT/SGPT, total bilirubin (fractionate if total >1.5 times normal), alkaline phosphatase, GGT (γ-glutamyl transaminase), albumin,
  - Hematology (CBC): hemoglobin, hematocrit, platelets, WBC, WBC differential

2.2.2. Secondary Endpoints (Efficacy)

- Difference in rate of decline of Frailty defined as:
  - Reduced Activity
  - Slowing of Mobility
  - Weight Loss
  - Diminished handgrip strength
  - Exhaustion
- Difference in subject quality of life assessment(s):
- Death from any cause.
- Exercise change in ejection fraction
- The following panel of inflammatory markers: CRP, IL-6, D-dimer, fibrinogen, CBC with differential, DNA, CMV, and TNFα

3. STUDY DESIGN

3.1 Description of the Study

A Pilot Phase will be performed to test the safety of dose and volume escalation of cells administered via peripheral intravenous infusion. The randomized portion of the study will be conducted after a full review of the safety data from the Pilot Phase by the DSMB.

Following the pilot study thirty (30) patients will be scheduled to undergo peripheral intravenous infusion and meeting all inclusion/exclusion criteria will be evaluated at baseline.

3.2. RANDOMIZATION STUDY

This Phase I, randomized, blinded, placebo-controlled study is designed to evaluate the safety and tolerability of allo-hMSCs in patients with Frailty and to explore potential efficacy over 4 weeks.

Approximately fifteen (15) subjects will be enrolled in the pilot phase and thirty (30) subjects with Frailty will be enrolled for a total of forty-five (45) subjects. Subjects will then be enrolled and randomized 1:1:1 to an active arm or placebo. Additional subjects may be enrolled if deemed appropriate.

Eligible subjects must have a diagnosis or symptoms of frailty as defined by the Canadian Study on Health &Aging[2]. Following informed consent before or at the screening visit, the diagnosis of FRAILTY will be confirmed by investigator review of medical history.

In the randomized phase of the trial, manual randomization will be performed using sealed, opaque envelopes and communicated to cellular laboratory personnel who have no contact with the investigators or subjects. At the time of administration, opaque tubing

will be used to maintain double blinding. Treatments will be administered once and will consist of 1 x 10^8allo-hMSCs (100 million cells), 2 x 10^8 allo-hMSCs (200 million cells), or placebo. After each infusion, patients will be monitored for immediate complications.

Continued safety and tolerability with review of adverse events (AEs) will be monitored at each visit. Efficacy parameters (pulmonary function tests, 6MWT, and QOL questionnaires) will be assessed every 12 weeks until study completion. Clinical laboratory tests to assess safety will be performed at every visit.

4. SUBJECT SELECTION

4.1 Inclusion Criteria

In order to participate in this study, a patient MUST:

- Provide written informed consent.
- Subjects age $\geq$60 and $\leq$95 years at the time of signing the Informed Consent Form.
- Show signs of frailty apart from a concomitant condition as assessed using clinical frailty scale.

4.2 Exclusion Criteria

In order to participate in this study, a patient MUST NOT:

- Inability to perform any of the assessments required for endpoint analysis (report safety or tolerability concerns, perform PFTs, undergo blood draws, read and respond to questionnaires.
- Active listing (or expected future listing) for transplant of any organ.
- Clinically important abnormal screening laboratory values, including but not limited to: hemoglobin <8 g/dl, white blood cell count <3000/mm^3, platelets<80,000/mm^3, INR > 1.5, aspartate transaminase, alanine transaminase, or alkaline phosphatase > 3 times upper limit of normal, total bilirubin > 1.5 mg/dl.
- Serious comorbid illness that, in the opinion of the investigator, may compromise the safety or compliance of the patient or preclude successful completion of the study. Including, but not limited to: HIV, advanced liver or renal failure, class III/IV congestive heart failure, myocardial infarction, unstable angina, or cardiac revascularization within the last six months, or severe obstructive ventilatory defect.
- Any other condition that, in the opinion of the investigator, may compromise the safety or compliance of the patient or preclude successful completion of the study.

- Have known allergies to penicillin or streptomyocin. Be an organ transplant recipient.
- Have a clinical history of malignancy within 5 years (i.e., patients with prior malignancy must be disease free for 5 years), except curatively-treated basal cell carcinoma, squamous cell carcinoma, or cervical carcinoma.
- Have a non-pulmonary condition that limits lifespan to < 1 year.
- Have a history of drug or alcohol abuse within the past 24 months.
- Be serum positive for HIV, hepatitis BsAg or Viremic hepatitis C.
- Be currently participating (or participated within the previous 30 days) in an investigational therapeutic or device trial.
- Be a female who is pregnant, nursing, or of childbearing potential while not practicing effective contraceptive methods. Female patients must undergo a blood or urine pregnancy test at screening and within 36 hours prior to injection.
- Female subjects must have an FSH < 25.8 IU/L
- Have hypersensitivity to dimethyl sulfoxide (DMSO)

4.3 Concomitant Treatments, Procedures, and Nondrug Therapies

All concomitant medications (prescription or over-the counter) as well as procedures or nondrug therapies (e.g. continuous positive airway pressure, pulmonary rehabilitation) will be recorded at the initial screening visit and updated at each subsequent visit. Except for other experimental treatments or medications with putative disease modifying effects in FRAILTY, subjects will continue all prior concomitant medications for comorbid diseases to ensure optimal general medical care.

4.4 Withdrawal Criteria

Patients will be informed that they have the right to withdraw from the study at any time and for any reason without prejudice to future or continued medical care. Patients must be withdrawn for the following reasons:

- Patient request.
- Patient is unable or unwilling to comply with the protocol.
- Medical reasons, at the discretion of the investigator.

Reason for withdrawal will be recorded in the subject's case report form. In order to adequately monitor for safety and potential efficacy outcomes, subjects who are withdrawn for any reason after receiving the first infusion should be encouraged to return for all assessments through the end of the study period. All efforts should be made to continue to record safety data and lung function parameters for all withdrawn subjects. Patients who withdraw for reasons unrelated to the study or study drug (e.g. withdrawal

of consent or loss to follow-up) may be replaced if deemed necessary to meet study objectives. Replacement subjects will be assigned unique identification numbers.

5. MESENCHYMAL STEM CELL DONORS

The availability of allogeneic hMSCs (allo-hMSC) offers the potential for an “off the shelf” product for patients. Significant data has been generated to demonstrate that the allogeneic hMSCs are immunoprivileged and can be infused without immune rejection despite disparate HLA phenotypes.

Screening of allogeneic donors will follow standard transplant practices and all allogeneic donors will meet allogeneic donor eligibility criteria as outlined in 21 CFR Part 1271.

Allogeneic donor testing will include anti-HIV-1/2, anti-HTLV I/II, anti-HCV, HIV-1 nucleic acid testing, HCV nucleic acid testing, HBsAg, anti-HBc(IgG and IgM), anti-CMV, West Nile Virus nucleic acid, *T. cruzi* ELISA (Chagas), and RPR. Potential donors testing positive for any of these infectious diseases will be ineligible. Bone marrow (BM) aspirates will be obtained from a maximum of 15 normal individuals and allo-hMSCs will be isolated and expanded.

5.1 Bone Marrow Aspiration for Generation of MSCs

A total of 120ml of BM will be obtained from each normal volunteer. BM will be aspirated from the posterior iliac crest into heparinized syringes. The mononuclear cell fraction will be isolated using a density gradient with Lymphocyte Separation Media (specific gravity 1.077). The low-density cells will be collected and washed with Plasma-LyteA containing 1% HSA. The washed cells will be sampled and viable cell numbers determined. The BM mononuclear cells will be seeded into 225 cm^2 tissue culture flasks in alpha MEM containing 20% FBS. After 14 days of culture, passage zero (P0) cells will be harvested by trypsin treatment and expanded into 60 flasks. These flasks are incubated for a further 7 to 10 days and then the MSCs are harvested by trypsin treatment (P1 cells).

5.2 Normal Donor Eligibility

Male donors between the ages of 20 to 35 will be screened as potential BM donors. Women will not be eligible to be normal donors because women who have had prior pregnancies may acquire antibodies to male antigens. A maximum of 15 subjects and will be evaluated by history and physical examination. The history will include:

- History of malignancy
- Bleeding abnormalities
- Prior deep venous thrombosis
- Known cardiac or pulmonary conditions
- Prior blood transfusions
- Vaccinations
- Questions to identify persons at risks of infectious disease transmission

- Questions to identify persons at risk of transmitting hematological or immunological disease
- A physician will administer the National Marrow Donor Program (NMDP) Questionnaire (a donor health history screening questionnaire).

The physical examination will include evaluation for potential risks associated with the BM aspiration procedure. Prospective donors will have infectious disease testing including:

- Hepatitis B surface antigen (HBsAg)
- Anti-Hepatitis B core antibody (HBcAb)
- Anti-Hepatitis C virus antibody (HCV Ab)
- Anti-Human Immunodeficiency Virus (HIV) antibody (HIV 1/2)
- Cytomegalovirus antibody (CMV)
- HCV/HIV Nucleic Acid test
- West Nile Virus Nucleic Acid test
- Rapid Plasma Reagin (RPR)
- Human T-lymphotropic Virus I/II (HTLV I/II)
- *T. cruzi* ELISA test (Chagas disease)

Prospective donors will also have the following blood tests:

- Complete blood count with differential
- Complete metabolic panel, magnesium, calcium, and uric acid

Eligibility Criteria for Normal Donors will include:

- Male gender
- No history of malignancy
- No active coagulopathy and/or hypocoagulable state
- No history of cardio/pulmonary conditions
- Negative tests for Hepatitis B, Hepatitis C, RPR, Chagas, HIV ½, HTLV I/II and NAT for HCV, HIV, and WNV.
- Hemoglobin $\geq$ 13.0 g/dL
- Platelet count 140,000 to 440,000/ul
- WBC 3.0 to 11.0 K/ul
- No anomalies on the CBC and differential suggestive of a hematopoietic disorder
- Creatinine$\leq$1.5 mg/dL
- ALT$\leq$ 112 IU/L
- Bilirubin < 1.5 mg/dL
- No diabetes
- Systolic blood pressure $\leq$ 170
- Diastolic blood pressure $\leq$ 90
- No history of autoimmune disorders

5.3 Donor Consent

Informed consent will be obtained from all potential donors. The procedure will be explained in terms the donor can understand, and will include information about the significant risks of the procedure. Potential donors will have an opportunity to ask questions, the right to refuse or with draw consent, and access to the results of all tests.

5.4 Follow-up Schedule for Donors

After discharge from the hospital, the bone marrow donor will be contacted by the study team with periodic follow-up telephone calls over a period of one year to determine the well-being and health status of the donor. The donor will be provided with contact telephone numbers in the consent form for any questions or comments.

6. TREATMENT OF PATIENTS

6.1 Study Investigational Product

The investigational agent consists of hMSCs obtained from donor bone marrow. Screening of allogeneic donors will follow standard transplant practices and all allogeneic donors will meet allogeneic donor eligibility criteria as outlined in 21 CFR Part 1271. BM will be obtained from normal volunteers with 120 ml aspirated from the posterior iliac crest. The BM will be aspirated into heparinized syringes. The MNC fraction will be isolated using a density gradient with Lymphocyte Separation Media (specific gravity 1.077). The low-density cells will be collected and washed with Plasma-LyteA containing 1% HSA. The washed cells will be samples and viable cell numbers determined. The BM MNC will be seeded into 175 cm^2 tissue culture flasks in alpha MEM containing 20% FBS. After 14 days of culture, passage zero (P0) cells will be harvested by trypsin treatment and expanded into 60 flasks (P1 cells). After 7 to 10 days P1 cells are harvested by trypsin treatment (P1 cells). Cells from P1 will be cultured for 7 to 10 days and harvested by trypsin treatment and expanded into 180 flasks (P2 cells). After 7-10 days P2 cells are harvested by trypsin treatment and cryopreserved.

6.2 Dosing

During the pilot phase of the study 15 subjects will receive a single infusion of hMSCs

Group 1 will consist of Five (5) subjects and will be treated with a single administration of allogeneic hMSCs: 2 x 10^7 (20 million) cells delivered via peripheral intravenous infusion.

Group 2 will consist of Five (5) subjects and will be treated with a single administration of allogeneic hMSCs: 1 x 10^8 (100 million) cells delivered via peripheral intravenous infusion.

Group 3 will consist of Five (5) subjects and will be treated with a single administration of allogeneic hMSCs: 2 x 10^8 (200 million) cells delivered via peripheral intravenous infusion.

In the randomized phase of allo-hMSCs or matched placebo 15 subjects will be randomized in a 1:1:1 ratio to one of two Treatment Strategies or placebo following successful completion of the Pilot Phase.

Group A will consist of 10 subjects that will receive 100 million Allogeneic hMSCs delivered via peripheral intravenous infusion.

Group B will consist of 10 subjects that will receive 200 million Allogeneic hMSCs delivered via peripheral intravenous infusion.

Group C will consist of 10 subjects who will receive placebo via peripheral intravenous infusion.

The Allo-hMSCs will be derived from approximately 15 normal donors meeting criteria for allogeneic unrelated human bone marrow stem cell source manufactured by the University of Miami.

6.3 Dosage Rationale

A safety profile for IV infusion of hMSCs was based on results from previous completed toxicology results[17]. The results from previous studies demonstrate that the product can be administered intravenously without toxic events at up to 65 x 10^6hMSC/kg dose delivered in one bolus infusion or at 100 x 10^6hMSC/kg cumulative dose delivered by 5 infusions (20 x 10^6hMSC/kg per infusion).

The evidence supports the conclusion that it is feasible to dose patients in this study based on a standard dose of hMSCs rather than per kilogram of body weight. The total cell number corresponds to a range of 1.3 - 4.4 x 10^6 hMSCs per kg per infusion for patients with 45 to 150kg body weight, the weight range for this study.

Therefore, results from previous trials support the rationale on the safety and potential efficacy of the selected maximum dose of 100 x10^6 allohMSCs.

6.4 Administration Rate

Prior clinical trials have used rates up to 30×10^6 hMSC/min where no infusion related toxicity was observed.

In the proposed study, the cell dose to be delivered is 1x 10^8 , and 2×10^8hMSC/infusion, reconstituted in a total volume of 25ml, delivered at a rate of 2 ml/min, and delivered at a maximum rate of 16×10^6 hMSC/minute and will last approximately 12.5 minutes +/- 25

minutes. The infusion bag will be flushed with an additional 25 ml of 0.9% normal saline at the completion of allo-hMSC infusion and delivered at a rate of 2ml/min.

6.5 Concomitant Therapy

6.5.1 Permitted therapy

Concomitant medications will be recorded on the case report form (CRF), which includes all FDA-approved medications and therapies.

6.5.2 Excluded therapy

Medications and therapies not approved by the FDA are prohibited for the duration of this trial, including participation with any investigational drug or device.

6.5.3. Subject monitoring

All aspects of the study will be conducted in accordance with Good Clinical Practice (GCP) as described in the ICH Guideline (CFR ICH Selected Regulations and Guidance for Drug Studies, CFR Title 21 Food and Drugs Revised as of April 1, 2002) all applicable national and local regulations. Monitoring will be conducted by a qualified outside source at the study site.

Monitoring of key safety endpoints will be conducted. If rates significantly exceed the pre-set threshold, then the DSMB will be advised.

6.6 Blinding and Unblinding

Subjects will be randomized into active groups. Only designated technicians in the ISCI Cell Processing Laboratory will be unblinded to treatment. The investigator, study staff, subject and anyone involved in the care of the subject will not be made aware of the assigned treatment regimen. The designated cell-processing technicians will prepare both the allogeneic hMSCs infusions. The investigational agent infusions will be prepared in identical infusion bags and labeled with the identical investigational drug labels. The designated technicians in the ISCI Cell Processing Laboratory (or designee) will be responsible for maintaining the investigational agent records including randomized treatment assignments by subject identification.

If for important medical reasons unblinding is thought to be necessary, the Investigator may identify the treatment assignment by obtaining the randomization assignment by contacting the Director of Experimental and Clinical Cell Based Therapies at ISCI who is responsible for maintaining randomization records for all patients.

6.7 Study Investigational Therapy Management

6.7.1 Investigational Product Labeling and Storage

The product label contains the elements required by the CFR and other national and local authorities for investigational products. ISCI GMP will directly store and deliver the designated cell processing technologist in the CPL, and will be kept cryopreserved in liquid nitrogen vapor phase until shortly before administration must be stored in a securely locked enclosure. Access is strictly limited to unblinded CPL personnel prior to preparation for infusion. After preparation for infusion, the Investigator and his or her designees are permitted to administer the Investigational Agent only to subjects participating in this protocol.

6.7.2 Investigational Product Accountability Procedures

In accordance with all applicable regulatory requirements, the Cell Processing Laboratory will maintain a record of the investigational agents hMSCs received, dispensed, administered, destroyed, or returned. The final disposition of all unused, empty, and partially used Cryocyte™ bags will be handled in accordance with the drug preparation manual. An independent unblinded clinical research associate (monitor) or auditor will perform compliance monitoring during the study.

Only unblinded personnel may access accountability records until the study blind has been broken.

7. STUDY PROCEDURES

7.1. Time and Events Schedule

The <u>Time and Events Schedule</u> for the conduct of this study is shown in Table 2

Schedule of Assessments

Table 2: Time and Events Table

VISIT	Screening<br><br>Day -56<br><br>± 28 days	Baseline<br><br>Day -14<br><br>(-2 to - 4 weeks)	Day 1	Week 2<br><br>(Day 14)	Month 1<br>(Week 4)<br><br>(±/-2 weeks)	Month 3<br>(Week 12)<br><br>(±/-2 weeks)	Month 6<br>(Week 24)<br><br>(+/-2 weeks)	Month 12<br>(Week 48)<br><br>(±/-2 weeks)<br><br>*Phone Call Follow-up
Informed Consent	x							
Full Medical History	x							
Physical Exam	x	x	x	x	x	x	x	
12-lead (ECG)	x	x	x	x	x	x	x	
Concomitant Medications	x	x	x	x	x	x	x	x
Randomization		x						
Infusion Treatment (IP)			x					
Dobutamine Stress Echo Test	x							
Bone Density Scan		x						
FEV-1		x				x	x	
6 Minute Walk Test		x				x	x	
Dynamometer (handgrip)		x				x	x	
IIEF, SQOL-F Questionnaires		x			x	x	x	
QOL Questionnaires (ICECAP, EQ-5D, SF-36)		x			x	x	x	
Urinalysis	x				x	x	x	
Hemat., Chem., CBC, LFTs, INR, and other labs*	x		x	x	x	x	x	
HIV 1, HIV 2, and Hep. B & C	x							
Serum or Urine Pregnancy Test#	x		x					
Donor Screening Tests	x							
Review Adverse Events			x	x	x	x	x	x
Immune Monitoring			x				x	
Biomarker Assessment			x				x	

 CONFIDENTIAL AND PROPRIETARY

Time and Events Table Key:

* - The minimal laboratory requirements for hematological, liver function and renal function include:

Hematology Tests: white blood cell count, platelet count, hemoglobin and hematocrit.

Liver Function Tests: Albumin, alkaline phosphatase, alanine transaminase, asparate aminotransferase, prothrombin time / activated partial thromboplastin time, and bilirubin.

Renal Function Tests:creatinine, creatinine clearance, blood urea nitrogen (BUN), glomerular filtration rate, sodium, potassium, chloride, bicarbonate, and glucose.

Serum Uric Acid, Pro-BNP, and C-reactive protein (CRP), IL6, fibrinogen, D-Dimer,

# - A serum or urine pregnancy test will be completed within 36 hours prior to injection for females of childbearing potential.

‡ - The following biomarkers will be analyzed:

- **Cell-surface markers**: CXCR4, C-Kit, &Connexin 43
- **Transcriptomic/Proteome:** RNA, miRNA, protein samples, and telomerase, akt
- **Growth factors**: Sdf-1, notch,
- **Functional Assays:** cell growth rate, and CFU assay

X - Immune monitoring for graft rejection. The following markers will be used for analysis to assess for activated T-cells based upon a $CD3^+CD25^+$ or $CD3^+CD69^+$ phenotype:

- CD3, CD25, CD69

7.2 Study Phases and Visits

7.2.1 Screening Visit

See Table 2 for the procedures and assessment to be performed during the screening visit of the study. All screening visit test and procedures will occur upon signing the informed consent form (ICF). No screening exams will take place until the patient is fully informed of the research and signs the consent form.

Physical exam

A complete physical examination will include general appearance, skin, head and neck, Lymph nodes, musculoskeletal/extremities, cardiovascular, chest/lungs, abdomen, and neurological assessment. At screening information about the physical examination and any significant findings must be recorded in the source documentation at the study site. Weight should be measured at each physical exam on a calibrated scale with the subject wearing only light clothing and no shoes. Height (in bare feet or wearing only thin socks) will be measured at the Screening visit.

Vital signs

Vital sign measurements will be performed at least once on each study visit up to time of discharge. These measurements will consist of respiratory rate, heart rate, blood pressure, and temperature. Respiratory rate, heart rate, and blood pressure should be measured in a sitting position after 5 minutes of rest.

7.2.2 Baseline Visit

See Table 2 for the procedures and assessment to be performed during the baseline visit of the study. Once all screening exams are completed and it has been determined that the patient remains eligible for the study, patients will be enrolled into the study. The baseline visit will take place within four weeks from the screening visit.The listed procedures should all be performed as soon as practicable. Bone density Dexa scan will be performed at the baseline visit (if not done within three months prior to enrollment).

7.2.3 Day 1 Visit

See Table 2 for the procedures and assessment to be performed during the Day 1 visit of the study. The Day 1 visit will occur after all baseline tests are completed and it has been determined that the patient remains eligible. Once the patient is deemed eligible to continue in the study the patient will be administered the investigational product.

7.2.4 Week 2 – Month 6 Visits

See Table 2 for the procedures and assessment to be performed for week 2 through month 6 visit of the study. Outpatient visits should be completed as close to the

scheduled visit dates as possible. There will be a +/- window of 2 weeks for the week 2 through month 6 study visits.

7.2.5 Month 12 Visit

See Table 2 for the procedures and assessments to be performed for Month 12 visit. This visit will be conducted via a phone interview with the patient. There will be a +/- window of 2 weeks for this visit.

7.2.6 Immune Monitoring for Graft Rejection

The studies planned in the Cratus protocol will utilize allogeneic mesenchymal stem cells (MSC) in patients with frailty syndrome. The use of an allogeneic graft raises the potential of graft rejection through immune cells resulting in failure of the therapy. MSCs are ideal candidates for allogeneic transplantation because they show minimal MHC class II and ICAM expression and lack B-7 co-stimulatory molecules necessary for T-cell mediated immune responses[57, 58]. Indeed MSCs do not stimulate a proliferative response from alloreactive T-cells even when the MSCs have differentiated into other lineages or are exposed to proinflammatory cytokines. Previous studies have demonstrated that MSCs have significant immunomodulatory effects, inhibiting T-cell proliferation, prolonging skin allograft survival, and decreasing graft-versus-host disease (GVHD). Recently human MSCs were shown to alter the cytokine secretion profile of dendritic cells, T cells, and natural killer cells in vitro, inhibiting secretion of proinflammatory cytokines (e.g. TNF-a, IFN-γ) and increasing expression of suppressive cytokines (e.g.IL-10), possibly via a prostaglandin E2 mediated pathway.

In vivo studies of the fate of MSCs have shown that, when transplanted into fetal sheep, human MSCs engraft, undergo site-specific differentiation into various cell types, including myocytes and cardiomyocytes and persist in multiple tissues for as long as 13 months after transplantation in non-immunosuppressed immunocompetent hosts. Further, in vivo studies using rodents, dogs, goats, and baboons demonstrate that allogeneic MSCs can be engrafted into these species without stimulating systemic alloantibody production or eliciting a proliferative response from recipient lymphocytes. These findings, coupled with our demonstration of efficacy of these cells for cardiac repair, solidify the notion of using MSCs as an allograft for successful tissue regeneration.

As part of the Cratus protocol we will obtain peripheral blood samples from all patients to evaluate the presence of activated T cells. Two heparinized (green top) vacutainer tubes (approx. 15 cc total blood) will be collected at different time points during the study: at day one prior to infusion of MSC and at month 6. Peripheral blood mononuclear cells (PBMC) will be isolated from heparinized blood by ficoll sedimentation and will be viably cryopreserved for planned assessments of T cell activation.

Two of the best-accepted markers of T cell activation are CD69 and CD25 (IL-2 receptor □). We will monitor the activation of T cells by flow cytometric analysis of CD3+CD25+CD69+ cells in thawed PBMC. CD69 is an immediate/early marker of CD3+ T cell activation while CD25 expression increases within 1-2 days of activation

and remains sustained over the intermediate-long term during chronic immune activation. Given the differences in the kinetics of CD69 and CD25 up regulation, assessment of both activation phenotypes (CD3+CD69+ and CD3+CD25+) will maximize the sensitivity of detection of T cell activation following autologous or allogeneic MSC infusion.

8. SAFETY

8.1 Safety Variables

- Vital signs
- Physical examination
- Clinical laboratory tests
- Adverse events

8.2 Laboratory Evaluations

At screening, the HIV-1 and HIV-2 tests, hepatitis screen and β-HCG serum pregnancy tests (only for women of child bearing potential) will be performed locally at the study site. Laboratory safety tests will consist of the following:

Serum chemistry: sodium, potassium, chloride, bicarbonate, BUN, creatinine, glucose, calcium, phosphate, AST/SGOT, ALT/SGPT, total bilirubin (fractionate if total >1.5 times normal), alkaline phosphatase, GGT (γ-glutamyl transaminase), albumin, fibrinogen, IL6, D-Dimer, Coagulation studies

Hematology (CBC): hemoglobin, hematocrit, platelets, WBC, WBC differential

The Investigator will review all clinically relevant laboratory results requested in the protocol. The diagnosis associated with any clinically significant laboratory deviations should be recorded as an AE and should indicate the underlying abnormality or diagnosis (such as renal insufficiency) as opposed to the observed deviation in laboratory results (such as elevated creatinine). If there is no underlying abnormality linked to a clinically significant abnormal laboratory value, the observed deviation should be reported as the AE.

8.2.2 Pulse Oximetry

Pulse oximetry will be used to observe oxygen saturation when measuring vital signs at screening. Pulse oximetry will also be used throughout infusions and 2 hours following infusions. At Screening, oxygen saturation must be ≥93% to meet inclusion criterion in order to minimize risk of 93% infusional toxicity. Subjects must have a resting oxygen saturation of ≥93% in order to be randomly assigned. Patients requiring oxygen, need the peripheral artery oxygen saturation (SaO_2) to be ≥93% when given a maximum of 2L/minute supplemental O_2 via nasal cannula. Infusion toxicity will be assessed based

on decreases in oxygen saturation during infusion. The infusion must be stopped, if the subject's oxygen saturation decreases to less than 90% over a continual period of 3–5 minutes during IA infusion. If this occurs then subjects will be admitted to the hospital for observation.

8.2.3 Pregnancy

There is no information allogeneic hMSCs and its effects or potential risks to a fetus or unborn child. The Principal Investigator and DSMB must be notified within twenty-four hours of investigator's awareness of the pregnancy via facsimile if a study subject becomes pregnant during the study. Males and females of non-childbearing potential ≥ 40 to ≤ 86 years of age at the time of signing the Informed Consent Form with documented FRAILTY. Any one of the enumerated contraceptive items will be acceptable for meeting the studies contraceptive requirements as listed in section 8.2.2 Females will be defined as non-childbearing potential if surgically sterilized (i.e. bilateral tubal litigation, bilateral oophorectomy, or complete hysterectomy) or post-menopausal (defined as 12 months no menses with an alternative medical cause and with a follicle stimulating hormone (FSH) > 25.8 IU/L). Non-sterilized males who are sexually active with a female partner of childbearing potential must use any one of the enumerated contraceptive items as listed in section 8.2.2 throughout the study.

Acceptable forms of contraception include: 1) abstinence, 2) condoms (male or female) with a spermicidal agent, 3) diaphragm or cervical cap with spermicidal agent, 4) intrauterine device (IUD), 5) oral contraceptive, 6) injectable or transdermal hormonal contraceptive, 7) successful vasectomy with resulting azoospermia or azoospermia for any other reason, and 8) hysterectomy, bilateral oophorectomy, or tubal ligation.

Prior to study enrollment, women of childbearing potential must be advised of the importance of avoiding pregnancy during trial participation and the potential risk factors for a pregnancy. The subject must sign an informed consent and written authorization for use and disclosure of PHI document stating that the above-mentioned risk factors and the consequences were discussed with her.

8.2.4 Determination of Infusional Toxicity

Infusional toxicity will be evaluated by continuously monitoring the subject's vital signs and O_2 saturation by pulse oximetry from the time of allogeneic hMSCs administration until two hours after infusion is complete. Since there is no specific or antidotal therapy for AEs arising from allogeneic hMSCs, any toxicity that may arise during a subject's participation in this study should be managed with supportive measures at the discretion of the treating physician.

8.2.5 Subject Stopping Guidelines

This guideline is to be used to indicate boundaries requiring discussion by the Data and Safety Monitoring Board (DSMB) and is designed to assist the independent DSMB in overseeing the study. The DSMB may also request additional interim analyses and develop other criteria including provision for monitoring of potential late effects to determine when to intervene in the enrollment or treatment of patients in the study.

The following are subject stopping guidelines:

- Any subject who develops persistent (that is, still existing more than 3 hours after the end of IP infusion) cardiorespiratory signs or symptoms (for example, shortness of breath, tachypnea, tachycardia, hypotension, or palpitations) will continue with all scheduled follow-up if such follow-up is considered safe in the opinion of the Investigator.

- Any subject whose infusion is stopped due to cardiorespiratory distress will receive no further IP infusions but will continue with all scheduled follow-up if such follow-up is considered safe in the opinion of the Investigator.

- Any subject who develops any sign or symptom that, at the discretion of the Investigator, warrants the discontinuation of infusion will receive no further IP infusions but will continue with all scheduled follow-up if such follow-up is considered safe in the opinion of the Investigator.

- Infusion of the IP may be stopped if there is an adverse event that the Investigator believes is related to the IP or if there is an issue with the IP infusion.

- The proportion of patients experiencing TE-SAE as defined in Section 2.2.1 will be monitored within 30 days of injection. This guideline is designed to assist the independent DSMB in overseeing the study and indicate boundaries needing discussion by the DSMB. The DSMB may also request additional interim analyses and develop other criteria including provision for monitoring of potential late effects to determine when to intervene in the enrollment or treatment of patients in the study.

- Monitoring of key safety endpoints will be conducted. If rates significantly exceed the pre-set threshold, then the DSMB will be advised.

- The stopping guidelines serve as a mechanism for consultation with the DSMB for additional review, and are not formal “stopping rules” that would mandate automatic closure of study enrollment.

8.2.6 Subject observation and discontinuation after IP administration

The IP administration guidelines in **Appendix 1** list the study requirements for subject observation and discharge after IP administration.

8.3 Definition of an Adverse Event

An Adverse Event (AE) is any untoward medical occurrence in a patient or clinical investigation subject temporally associated with the use of a medicinal product, whether or not considered related to the medicinal product. The occurrence does not necessarily have to have a causal relationship to the treatment received in the study. An AE can therefore be any unfavorable and unintended sign (including an abnormal laboratory finding, for example), symptom, or disease (new or exacerbated) temporally associated with the use of a medicinal product, whether or not considered related to the medicinal product.

Examples of an AE include:

- Exacerbation of a chronic or intermittent pre-existing condition including either an increase in frequency or intensity of the condition.
- Significant or unexpected worsening or exacerbation of the condition/indication under study.
- A new condition detected or diagnosed after study therapy administration even though it may have been present prior to the start of the study.
- Pre- or post-treatment events that occur as a result of protocol-mandated procedures (e.g., invasive protocol-defined procedures, modification of a patient's previous treatment regimen).

An AE does **not** include:

- Medical or surgical procedures (e.g., colonoscopy, biopsy). The medical condition that leads to the procedure is an AE.
- Social or convenience hospital admissions where an untoward medical occurrence did not occur.
- Day to day fluctuations of pre-existing disease or conditions present or detected at the start of the study that do not worsen.
- The disease/disorder being studied or expected progression, signs, or symptoms of the disease/disorder being studied unless more severe than expected for the patient's condition.

8.4 Definition of Adverse Reaction

An adverse reaction is any adverse event caused by a drug. Adverse reactions are a subset of all suspected adverse reactions for which there is reason to conclude that the drug caused the event.

8.5 Definition of Suspected Adverse Reaction

Suspected adverse reaction means any adverse event for which there is a reasonable possibility that the drug caused the adverse event. For the purposes of IND safety reporting, 'reasonable possibility' means there is evidence to suggest a causal relationship between the drug and the adverse event. A suspected adverse reaction implies a lesser degree of certainty about causality than adverse reaction, which means any adverse event caused by a drug.

8.6 Definition of Serious

An adverse event (AE) or suspected adverse reaction is considered "serious" if it:

1. results in death
2. is life-threatening (at risk of death at the time of the event)
3. requires in inpatient hospitalization or prolongation of existing hospitalization

   NOTE: Complications that occur during hospitalization are AEs. If a complication prolongs hospitalization or fulfills any other serious criteria, the event is serious. Hospitalization for elective treatment of a pre-existing condition that did not worsen from baseline is not considered to be an AE.
4. results in disability/incapacity

   NOTE: The term disability means a substantial disruption of a person's ability to conduct normal life functions. This definition is not intended to include experiences of relatively minor medical significance such as uncomplicated headache, nausea, vomiting, diarrhea, influenza, accidental trauma (i.e., sprained ankle) that may interfere or prevent everyday life functions but do not constitute a substantial disruption.
5. Is a congenital anomaly/birth defect.

Important medical events that may not result in death, be life-threatening, or require hospitalization may be considered an SAE when, based upon appropriate medical judgment, they may jeopardize the patient or subject and may require medical or surgical intervention to prevent one of the outcomes listed in the above definition.

8.7 Definition of Unexpected

An adverse event or suspected adverse reaction is considered "unexpected" if it is not listed in the investigator brochure or is not listed at the specificity or severity that has been

observed; or, if an investigator brochure is note required or available, is not consistent with the risk information described in the general investigational plan or elsewhere in the current application.

8.8 Clinical Laboratory Assessments and Other Abnormal Assessments as Adverse Events and Serious Adverse Events

Abnormal laboratory findings (e.g. clinical chemistry, hematology) or other abnormal assessments (e.g., vital signs) that are judged by the Investigator as clinically significant will be recorded as AEs or SAEs if they meet the definition of an AE as defined in Section 8.3 (“Definition of an Adverse Event”) or SAE, as defined in Section 8.6 (“Definition of a Serious Adverse Event”). Clinically significant abnormal laboratory findings or other abnormal assessments that are detected during the study or are present at screening and significantly worsen following the start of the study will be reported as AEs or SAEs. However, clinically significant abnormal laboratory findings or other abnormal assessments that are associated with the disease being studied, unless judged by the Investigator as more severe than expected for the patient’s condition, or that are present or detected at the start of the study but do not worsen, will not be reported as AEs or SAEs.

The Investigator will exercise medical judgment in deciding whether abnormal laboratory values are clinically significant.

8.9 Recording of Adverse Events and Serious Adverse Events

The Investigator should review all documentation (e.g., hospital progress notes, laboratory, or diagnostic reports) relative to the event being reported. The Investigator will then record all relevant information regarding an AE/SAE into the electronic data system. It is not acceptable for the Investigator to send photocopies of the patients’ medical records in lieu of completion of the appropriate AE/SAE pages.

The Investigator will attempt to establish a diagnosis of the event based on signs, symptoms, and/or other clinical information. In such cases, the diagnosis should be documented as the AE/SAE and not the individual signs and symptoms.

SAEs will be reported to the IRB within 10 working days or within 24 hours if the event is life-threatening or results in death.

Pregnancies

Patient pregnancy must be reported to the Principal Investigator within 1 working day of knowledge of the event. Any patient who becomes pregnant during the study must be promptly withdrawn from the study. Follow-up information regarding the outcome of the pregnancy and any postnatal sequelae in the infant will be required.

8.10 Intensity of Adverse Events and Serious Adverse Events

The Investigator will make an assessment of intensity for each AE and SAE reported during the study. The assessment will be based on the Investigator's clinical judgment. The intensity of each AE and SAE should be assigned to one of the following categories:

Mild:	An event that is easily tolerated by the patient, causing minimal discomfort and not interfering with everyday activities.
Moderate:	An event that is sufficiently discomforting to interfere with normal everyday activities.
Severe:	An event that prevents normal everyday activities.
Life-threatening:	Immediate risk of death.

An AE that is assessed as severe should not be confused with an SAE. Severity is a category utilized for rating the intensity of an event; and both AEs and SAEs can be assessed as severe. An event is described as 'serious' when it meets one of the pre-defined outcomes as described in Section 8.6, "Definition of Serious."

8.11 Causality of Adverse Events and Serious Adverse Events

The Investigator is obligated to assess the causality between study therapy and the occurrence of each AE/SAE. The Investigator will use clinical judgment to determine if there is a reasonable possibility that the biological action of the study therapy was responsible for AE/SAE being reported. Alternative causes such as natural history of the underlying diseases, concomitant therapy, other risk factors, and the temporal relationship of the event to the study therapy will be considered and investigated. The Investigator will also consult the Clinical Investigator's Brochure and/or Product Information, for marketed products, in the determination of his/her assessment.

The Investigator will use the following questions when assessing causality of an adverse event to study therapy.

Is there a reasonable possibility that the study therapy caused the event? Reasonable possibility implies that there is evidence that the vent was caused by the study product. An affirmative answer designates the event as a suspected adverse reaction.

There may be situations when an SAE has occurred and the Investigator has minimal information to include in the initial report. However, it is very important that the Investigator always make an assessment of causality.

The relationship between AEs and the study exposure will be classified by the investigator as:

- None: No relationship. Related to other known etiologies, conditions, or exposures.
- Unlikely: Current knowledge suggests that a relationship is unlikely.

- Possible: A plausible temporal sequence or response pattern exists but the AE may be related to other known etiologies, conditions, or exposures.
- Probable: A plausible temporal sequence or response pattern exists and the AE cannot be related other known etiologies, conditions, or exposures.

- Definite: A plausible temporal sequence or response pattern exists and the AE can be confirmed by re-challenge or with other supporting data.

8.12 Follow-Up of Adverse Events and Serious Adverse Events

After the initial AE/SAE report, the Investigator is required to proactively follow each patient and provide further information on the patient's condition. All AEs and SAEs documented at a previous visit/contact that are designated as ongoing will be reviewed at subsequent visits/contacts.

Adverse events and SAEs will be followed until resolution, until no further changes in the event are expected (i.e. the point at which a patient experiencing a critical adverse event is treated successfully and stabilized even though they may continue to experience lingering sequelae that may never resolve), until the patient is lost to follow-up, or until it is agreed that further follow-up of the event is not warranted (e.g. non-serious, study therapy unrelated, mild or moderate adverse events ongoing at a patient's final study visit). If a patient dies during participation in the study or during a recognized follow-up period, the Investigator will provide a copy of any post-mortem findings, including histopathology.

New or updated information will be recorded by modifying the AE forms in the electronic data system

8.13 Timeframes for Submitting SAE Reports

Once an Investigator becomes aware that an SAE has occurred in a study patient, he/she will record the information in the electronic data record within 48 hours. Any fatal or life-threatening event must be reported within 24 hours. If the Investigator does not have all information regarding an SAE, he/she will not wait to receive additional information before recording the event in the data system and completing as much information known at the time of the submission. The reporting timeframes for any SAE occurring during the study are summarized in Table 3.

<u>TABLE 3</u>
Serious Adverse Event Reporting Requirements

	**Initial Reports**		**Follow-Up Reports**
**Type of SAE**	Fatal or Life-Threatening	Other SAEs	Any SAE
**Reporting Timeframes**	24 hours	48 hours	48 hours
**Documents Required**	24 hours:<br>Complete as much information in the electronic data system that is known.<br>48 hours:<br>Fully complete all AE forms	Fully completed AE forms	Updated AE Forms

8.14 Post-Study Adverse Events and Serious Adverse Events

The Investigator should report any death or SAE occurring at any time after a patient has completed or terminated a clinical trial, when such death or SAE may reasonably be related to the study therapy used in an investigational trial. Investigators are not obligated to actively seek AEs from former study participants.

8.15 Regulatory Aspects of Adverse Event Reporting

The Investigator will promptly report all SAEs within the timeframes specified in Section 8.13. Prompt notification of SAEs by the Investigator is essential so that UMMSM can meet legal obligations and fulfill ethical responsibilities towards the safety of all patients participating in UMMSM-sponsored investigational trials.

The Investigator will comply with the applicable local regulatory requirements related to reporting of SAEs to his or her Institutional Review Board (IRB) or Independent Ethics Committee (IEC).

This protocol has been filed under an Investigational New Drug (IND) application with the FDA. A given SAE may qualify as an Expedited Safety Report (ESR) if the SAE is both at least possibly attributable to study therapy and unexpected. In this case, all Investigators participating in an IND study will receive an ESR.

The ESRs are prepared according to UMMSM policy and are forwarded to the Investigator as necessary. The purpose of the ESR is to fulfill specific regulatory and Good Clinical Practice (GCP) requirements regarding the product under investigation. Based on previous trials involving intravenous infusion of allogeneic human MSCs, no AEs have been attributed to treatment administration; therefore all AEs will be considered and documented as unexpected AEs.

All AEs occurring at any time during the trial (from the time of informed consent through 28 days after study completion) will be collected, documented, and reported by the

investigator. For each AE, the investigator will provide the date of onset and resolution, intensity, treatment required, outcome, seriousness, and potential causality with regards to the study exposure.

9. STATISTICAL ANALYSIS

9.1 Determination of Sample Size and Analysis Population

No formal statistical justification was performed to determine sample size in the Phase I study. Cohort size was determined based on expected requirements for safety analyses and projected enrollment rates. Study participants will be randomized according to a fixed allocation permuted block randomization schema. The allocation ratio will be 1:1:1 and the block size will be 4. All enrolled subject who received at least one treatment dose will be included in summaries of baseline characteristics, safety, and efficacy. Reasons for study discontinuation will be tabulated.

9.2 General Statistical Methods

All statistical tests will be performed at an $\propto$=0.05 level of significance, using two-sided tests. Because this is a Phase I study with only exploratory efficacy outcomes, no adjustments will be made for multiple analyses. Continuous variables will be presented by descriptive statistics. Categorical variables will be presented by counts. Two sided 95% confidence intervals will be calculated and presented where appropriate.

Analysis of AEs will include tabulation by frequency, severity, organ system affected, and relationship to study exposure. Lung function data will be summarized descriptively. Patient reported outcome data will be summarized according to the guidelines of each questionnaire.

9.3 Interim Analyses

Safety data analysis will be performed on the first 3 patients enrolled at Week 4 after infusion. After the first interim safety analysis, summaries of adverse events will be reviewed each week and detailed safety data will be reviewed every three months by the DSMB.

9.4 Data Safety Monitoring Board (DSMB)

9.4.1. ROLE OF THE DSMB

This study is designed to test the safety of hMSCs in patients with frailty.

The purpose of the data safety monitoring board (DSMB) is to advise the investigators regarding the continuing safety of study patients and those yet to be recruited to the study, as well as the continuing validity and scientific merit of the study.

This section describes the roles, responsibilities and operating procedures of the DSMB, and includes guidelines for communications and interactions between the DSMB and the investigators to schedule and format for meetings; format for presentation of data; specification of who will have access to interim data and who may attend all or part of DSMB meetings; procedures for assessing conflict of interest of potential DSMB members; and the method and timing of providing interim reports to the DSMB.

9.4.2. Purpose of the DSMB

The primary function of the DSMB is to provide unblinded assessments of patient safety throughout the study. This will be accomplished through regularly scheduled formal meetings and/or additional meetings to review interim summaries of safety and efficacy data. The DSMB will make recommendations regarding modification or termination of the study in the event of significant study conduct issues or safety concerns. The DSMB will not stop the study based on efficacy results favorable to hMSCs, other than for all-cause mortality as outlined below. The selected primary and secondary endpoints were chosen to measure major morbidity in patients with FRAILTY, a fatal disease. Given the importance of mortality in FRAILTY, a stopping boundary based on the all-cause mortality rate will be implemented to guide the DSMB. This stopping boundary will not be applied until after all patients have enrolled in the study.

9.4.2.1 DSMBMEMBERSHIP

The DSMB is an independent, multidisciplinary group consisting of one biostatistician and four clinicians who collectively have experience in the management of patients with FRAILTY and in the conduct and monitoring of randomized clinical studies.

The DSMB will meet until the study's database has been locked and a final data review has been completed. If a member withdraws from the DSMB, the DSMB chairperson will be responsible for selecting an appropriate replacement.

9.4.2.2 Financial Disclosure and Conflict of Interest

DSMB membership is restricted to individuals without significant potential or perceived conflicts of interest. The source of these conflicts may be financial, scientific, or regulatory in nature.

Members must disclose to the DSMB chairperson their consultancies (direct or indirect) in excess of $5,000 or financial interests in any pharmaceutical companies, biotechnology companies, or CROs, if these relationships could lead to any conflict of interest or these companies' products involve hMSCs.

The DSMB chairperson will be responsible for deciding whether consultancies or financial interests of the members materially impact their objectivity. This decision is to be based on the reasonable belief that their objectivity is not in doubt. DSMB members will

be responsible for advising the DSMB chairperson of any changes in financial interests in pharmaceutical companies, biotechnology companies, including consultancies, during the course of their membership. Members of the DSMB who develop significant potential or perceived conflicts of interest that may materially impact their objectivity will be asked to resign from the DSMB.

9.4.2.3 DSMB Responsibilities

The DSMB has the responsibility to:

1. Review the protocol and all amendments. The DSMB will also be provided the Investigator's Brochure, as it is updated, that includes preclinical and clinical efficacy and safety data.

2. Review the statistical analysis plan with particular attention to the portions describing the data to be provided to the DSMB.

3. Review Periodic Safety Update Reports (PSURs).

4. Evaluate the conduct of the study including the selection and retention of patients, extent of protocol deviations, treatment adherence, and the quality of the data. The DSMB shall make recommendations regarding study conduct as necessary to protect the scientific integrity of the study. The DSMB shall not make recommendations regarding the efficacy outcomes or associated analyses.

5. Review unblinded periodic safety summaries including adverse events, serious adverse events, discontinuations, and post-baseline laboratory results. In addition, efficacy and mortality data will be reviewed, although the DSMB shall not recommend stopping the study for efficacy.

6. The DSMB will make recommendations regarding modification or termination of the study in the event that significant safety concerns arise during study conduct.

9.4.2.4 Confidentiality

The DSMB will be unblinded in its assessment of safety and efficacy data to ensure that the DSMB is fully informed in its primary mission of safeguarding the interest of participating patients. The DSMB will have sole access to comparative results of safety data aggregated by treatment arm. The DSMB will take all necessary and appropriate steps to safeguard the confidentiality of unblinded treatment information it receives to minimize the potential for premature conclusions regarding the study results as well as the potential for introducing bias into the study.

9.4.2.5 Study Conduct and Termination

The DSMB will provide recommendations following review and assessment of the quality of study conduct. More specifically, the DSMB will review enrollment rates, consistency in complying with eligibility requirements, compliance with the study protocol as well as the completeness of the data. In their review of the data, the DSMB will be responsible for protecting the safety of the enrolled patients. If any potential question of safety arises, the DSMB will use the efficacy data to assess the possible safety risk in the context of the benefit-to-risk profile of study treatment. Based on this information, the DSMB may make recommendations to terminate the study if members believe that an undue risk (relative to benefit) would be incurred by allowing the study to continue to completion. Otherwise the study will be completed to allow investigators to complete the protocol mandated assessments to evaluate the safety of hMSCs in FRAILTY patients.

9.4.2.6 <u>Investigator Responsibilities</u>

The investigator has the responsibility to:

1. Make decisions based on DSMB recommendations in a timely fashion.
2. Notify study centers of the outcome of the DSMB meetings, and any DSMB recommendations addressing actions to be taken to ensure the integrity of the study.
3. Notify regulatory agencies of DSMB recommendations addressing any emerging safety concern not recognized at the start of the study.
4. Ensure that the unblinded DSMB support team is provided with the data necessary for the chosen analyses and reports.
5. Provide DSMB members with the current protocols and Investigator's Brochure.
6. Provide DSMB members with PSURs as published
7. Attend the open session of each DSMB data review meeting.

9.4.3 COMMITTEE MEETINGS

8.4.3.1 <u>Organizational Meeting</u>

At an organizational meeting the DSMB will discuss the operational aspects of the committee. This meeting will include DSMB members and the clinical monitor. The documents to be provided before this meeting are:

1. Study protocol
2. Preliminary DSMB Statistical Analysis Plan (SAP)
3. Preliminary list of tables and listings to be provided for interim assessments
4. Investigator's Brochure
5. Food and Drug Administration Guidance: *Establishment and Operation of Clinical Trial Data Monitoring Committees.*

For all DSMB meetings, a quorum is defined as at least two members of the DSMB in addition to the DSMB chairperson.

9.4.3.2 Review of Periodic Safety Update Reports

As part of ongoing safety review and obligation to regulatory agencies, safety reports will be reported every month. These reports will be available to the DSMB, who shall review them in the context of providing additional information to assist the committee's consideration of patient safety. The DSMB members may choose to discuss the PSURs during an ad hoc teleconference.

9.4.3.3 Data Review Meetings

The first DSMB meeting for safety assessment will occur after enrollment of approximately 25% of patients. Thereafter, DSMB meetings will take place approximately every 3 months until the conclusion of the study. In addition to the scheduled meetings, ad-hoc data review meetings may be called by any DSMB member, the DSMB chairperson, or sponsor at any time in case of an emerging safety concern. Data review meetings will have open and closed sessions. Unblinded data will only be evaluated at closed sessions.

Open session

Blinded data will be provided to the DSMB approximately 1 week before each data review meeting. The report will contain:

1. Protocol status including any protocol changes
2. Data sources and cutoff dates
3. Analysis methods applied specifically to the open session report
4. Patient enrollment by month
5. Protocol deviations
6. Early treatment discontinuations and study withdrawals
7. Demographic and baseline characteristics
8. Duration of follow-up at time of data cutoff

Closed Session

Only the DSMB members will participate in closed sessions. Unblinded data will be presented to the DSMB in closed session and discussed by the DSMB. "Unblinded" means that the name of actual treatment arm is associated with individual patient data listings and summaries of data. At the chairperson's discretion, the DSMB may discuss or vote on potential study conduct recommendations at closed session.

The closed session report will contain data separated and identified by treatment group. This report (hard copy) will be provided to the DSMB approximately 1 week before each data review meeting and will include:

1. Data sources and cutoff dates
2. Analysis methods applied specifically to the closed session report

3. Patient enrollment
4. Protocol deviations
5. Early treatment discontinuations and study withdrawals
6. Demographic and baseline characteristics
7. Primary and secondary efficacy outcome measures
8. Prohibited concomitant medications
9. Adverse events
10. Serious adverse events

Following the closed session, the DSMB chairperson will issue, within 24 hours, one of the following recommendations as determined by the DSMB:

1. Continue the study, with or without modifications.
2. Terminate the study for safety concerns.

Separate meeting minutes for the open and closed sessions will be prepared by the DSMB project manager, then reviewed and issued by the DSMB chairperson. The DSMB support team will maintain copies of the meeting minutes for both the open and closed meetings. To preserve the integrity of the study, the detailed rationale and discussion of comparative unblinded data will be included only in the closed meeting minutes

Follow-Up Open Session

Immediately following the closed session, the DSMB will meet with the investigators to discuss any study conduct concerns. This follow-up open session may be attended by DSMB Members, clinical monitor, and study staff. Potential recommendations from the DSMB regarding study discontinuation or continuation, with or without modification, will not be communicated in this open session.

Closed Executive Session

At the discretion of the DSMB chairperson, a closed executive session may be held. Closed executive sessions will include only DSMB members. Discussion of unblinded study data and potential DSMB recommendations and voting may take place in closed executive session. The DSMB may choose whether to write minutes of the closed executive sessions.

9.4.4 DATA FLOW

9.4.4.1 Communications and Reports

For each DSMB meeting, the open session and closed session reports will be prepared by the DSMB support team. Open session reports will be circulated to all attendees of the open session. The closed session reports will be circulated to DSMB members only. The closed session reports will be retrieved from the DSMB members by the DSMB

support team and destroyed after the closed session. The DSMB support team will maintain copies of all reports from the open and closed sessions.

9.4.4.2 Review of Unblinding Requests

Except as required by regulatory authorities for safety reporting, individual patients' treatment assignments will not be unblinded during the conduct of the study, unless a patient safety issue arises in which unblinding is necessary to ensure optimal patient management. It is not anticipated that unblinding will be necessary, given the hMSCs can be safely discontinued at any time a safety concern arises. The DSMB will be informed in a timely manner of any case for which unblinding was requested and performed.

9.4.4.3 DSMB Additional Analysis Requests

The DSMB may request additional analyses from the statistician if deemed necessary to fulfill the mission of the DSMB. If based on the additional data the DSMB feels there is a need for an unscheduled formal meeting, the DSMB chairperson will arrange.

9.4.4.4 Confidentiality

All documents will be held in strict confidence by the DSMB, and all documents provided to the DSMB will be collected and destroyed at the end of all DSMB meetings by the DSMB support team.

9.4.5 COMMUNICATION

9.4.5.1 DSMB Minutes

The DSMB chairperson is responsible for issuing minutes of the open and closed sessions. Minutes of the open session will be distributed to all open meeting attendees within two weeks of the meeting. Minutes of the closed session will be distributed to the members of the DSMB within two weeks of the meeting. At the conclusion of the study, the DSMB support team will send a complete set of the open and closed reports, minutes of the open and closed sessions with the tables and listings, all presentations and copies of the recommendation forms to the investigators.

9.4.5.2 DSMB Recommendations

If the DSMB recommends modification to, or termination of, the study, the chairperson of the DSMB will contact the investigators within 24 hours of making the decision.

Recommendations for modifications other than termination should be accompanied by the rationale for the recommendation and the minimum amount of data required to make a decision. The investigator will be responsible for promptly reviewing the DSMB

recommendations and determining whether amendments to the protocol or changes regarding the study conduct are required and if reporting to regulatory authorities is warranted (FDA, 2006).

Should there be disagreement between the DSMB and the investigators around the decision to stop or modify the study a separate compliance committee will be appointed. This committee is comprised of individuals who have extensive experience in the pharmaceutical industry and a deep appreciation of the ethical issues surrounding the conduct of clinical studies and are responsible for the investigator's code of ethics. The Compliance Committee is charged with independently evaluating differing opinions that may arise between the investigator and DSMB and applying the highest ethical standards with respect to determining the best interests of patients enrolled in the study.

10. STUDY ADMINISTRATION

10.1 Regulatory Authority Approval

This study will be conducted in accordance with Good Clinical Practice (GCP) requirements described in the current revision of International Conference on Harmonization of Technical Requirements of Pharmaceuticals for Human Use (ICH) Guidelines and all applicable regulations, including current United States Code of Federal Regulations (CFR), Title 21, Parts 11, 50, 54, 56, and 312 and Title 45, Part 164. Compliance with these regulations and guidelines also constitutes compliance with the ethical principles described in the current revision of the Declaration of Helsinki. This study will also be carried out in accordance with local legal requirements.

10.2 Ethics Approval

It is the Investigator's responsibility to ensure that prior to initiating this study; this protocol is reviewed and approved by the appropriate local IRB. The composition and conduct of this committee must conform to the United States CFR.

The IRB/IEC must also review and approve the site's informed consent form (ICF), other written information provided to the patient and all advertisements that may be used for patient recruitment.

If it is necessary to amend the protocol or the ICF during the study, the Investigator will be responsible for ensuring that the IRB/IEC reviews and approves these amended documents. An IRB/IEC approval of the amended protocol and/or ICF must be obtained in writing before implementation of the amended procedures and before new patients are consented to participate in the study using the amended version of the ICF.

10.3 Patient Informed Consent

Before being admitted to the clinical study, all patients must consent in writing to participate. An ICF will be given to each patient, which will contain all United States

federally required elements, all ICH-required elements, and Health Insurance Portability and Accountability Act Authorization (HIPAA) information in language that is understandable to the patient.

The process of obtaining the informed consent will be in compliance with all federal regulations, ICH requirements, and local laws.

The investigator or designee will review the study with each patient. The review will include the nature, scope, procedures, and possible consequences of the patient's participation in the study. The ICF and review must be in a form understandable to the patient. The Investigator or designee and the patient must both sign and date the ICF after review and before the patient can participate in the study. The patient will receive a copy of the signed and dated form, and the original will be retained in the site study files. The Investigator or his/her designee must emphasize to the patient that study participation is entirely voluntary and that consent regarding study participation may be withdrawn at any time without penalty or loss of benefits to which the patient is otherwise entitled.

If the ICF is amended during the study, the Investigator must follow all applicable regulatory requirements pertaining to approval of the amended ICF by the IRB/IEC. The site must use the amended consent form for all new patients and repeat the consent process with the amended ICF for any ongoing patients.

10.4 Confidentiality of Information

Patients' names will remain confidential and will not be included in the database. Only patient number, patient initials, and birth date will be recorded in the data system. If the patient name appears on any other document collected (e.g., hospital discharge summary), the name must be deleted before the document is transmitted. All study findings will be stored in electronic databases. The patients will give explicit permission for representatives of regulatory authorities and the IRB/IEC to inspect their medical records to verify the information collected.

Patients will be informed that all personal information made available for inspection will be handled in the strictest confidence and in accordance with all state, local, and federal data protection/privacy laws, including, without limitation, the HIPAA.

Participants will be asked to voluntarily provide written authorization prior to requesting or disclosing private health information either as part of the written ICF or as a separate authorization form. The authorization will contain all required elements specified by 45 CFR 164, and will allow the site to access study-related private health information until the conclusion of the clinical study. The authorization will remain valid and in full force and effect until the first to occur of (1) the expiration of two years after the study therapy is approved for the indication being studied, or (2) the expiration of two years after the research program is discontinued. Individual patient medical information obtained during this study is confidential and its disclosure to third parties (other than those mentioned in this Section) is strictly prohibited. In addition, medical information

obtained during this study may be provided to the patient's personal physician or to other appropriate medical personnel when required in connection with the patient's continued health and welfare.

The investigator will maintain a personal patient identification list (patient and treatment numbers with the corresponding patient names) to enable records to be identified.

10.5 Payments to Patients

Patients will be reimbursed $25 at the end of each follow-up visit (Month 1 – Month 6) for a total remuneration of $75). These disbursements are meant to cover the time required to complete these study visits and all necessary travel and parking expenses. Normal donors for generation of allo-MSC will be reimbursed $350 at the end of BM aspiration. This payment will compensate donors for lost time, parking, and travel expenses.

APPENDIX 1: Infusion Guidelines

Prior to the start of the infusion the following procedures and assessments will be conducted on the study subject:

1. Vital Signs: Blood pressure, heart rate, respiratory rate, and temperature, will be measured within 15 minutes prior to the initiation of the infusion.
2. Oxygen saturation will be continuously monitored by pulse oximetry for at least 30 minutes prior to initiation of IA infusion.
3. Confirm that IV access is established and that the IV catheter is no smaller than 20 gauge
4. Study personnel needs to verify that the following pre-medications have been administered 30 minutes prior to infusion per protocol:

    - Hydrocortisone 25 – 50 mg IV
    - Diphenhydramine (Benadryl) 25 – 50 mg IV

    Note: No other medications should be given during the infusion unless determined medically necessary by the Investigator.

5. Document pre-medications given prior to infusion on the source documents
6. Required materials as follows:
    - 0.9 % normal saline IV infusion bag
    - IV Pump tubing
    - Three-way stopcock
    - I V extension tubing (unless using a central line)
    - Volumetric infusion pump
    - Gloves
7. Connect stopcock to IV tubing and connect extension tubing to the stopcock.
8. Hang 0.9% normal saline infusion bag and flush the IV line such that it flows directly through a stopcock
9. Connect IV line to subject's IV access.
10. Remove 0.9% normal saline infusion bag and connect IV tubing to the volumetric infusion pump
11. Cover the IV tubing with the blinding material provided with the infusion bag by the drug preparation technician.

During the IA infusion the following procedures and assessments will be conducted on the study subject:

1. Monitor the subject continuously with pulse oximetry
2. Hang the blinded infusion bag. Investigational product (IP) should not be "piggybacked" through another line
3. Intravenously administer the IP at a rate of 2ml/min.

Note: Study personnel administering the IP must be present throughout the infusion process. The Investigator must be available at the site during the infusion process in case an emergency should arise.

4. Record the start time of the infusion bag
5. Gently squeeze the infusion bag several times every 15 minutes to assure uniform dispersion of contents
6. Vital signs and O_2 saturation will be measured every 15 minutes until the end of IP infusion
7. Record the total volume infused from the IP bag
8. Flush the IV line with 25ml of 0.9% normal saline
9. Discard IV tubing according to established guidelines
10. Return the blinded IP infusion bag to the cell-processing technician.

 CONFIDENTIAL AND PROPRIETARY

11. Reference List

(1) Morley JE, Vellas B, Abellan van KG et al. Frailty consensus: a call to action. *J Am Med Dir Assoc* 2013;14:392-397.

(2) Koller K, Rockwood K. Frailty in older adults: implications for end-of-life care. *Cleve Clin J Med* 2013;80:168-174.

(3) Ebrahimi Z, Wilhelmson K, Eklund K, Moore CD, Jakobsson A. Health despite frailty: Exploring influences on frail older adults' experiences of health. *Geriatr Nurs* 2013.

(4) Ekerstad N, Swahn E, Janzon M et al. Frailty is independently associated with 1-year mortality for elderly patients with non-ST-segment elevation myocardial infarction. *Eur J Prev Cardiol* 2013.

(5) Gale CR, Baylis D, Cooper C, Sayer AA. Inflammatory markers and incident frailty in men and women: the English Longitudinal Study of Ageing. *Age (Dordr)* 2013.

(6) Fabbricotti IN, Janse B, Looman WM, de KR, van Wijngaarden JD, Reiffers A. Integrated care for frail elderly compared to usual care: a study protocol of a quasi-experiment on the effects on the frail elderly, their caregivers, health professionals and health care costs. *BMC Geriatr* 2013;13:31.

(7) Fried LP, Tangen CM, Walston J et al. Frailty in older adults: evidence for a phenotype. *J Gerontol A Biol Sci Med Sci* 2001;56:M146-M156.

(8) Kanapuru B, Ershler WB. Inflammation, coagulation, and the pathway to frailty. *Am J Med* 2009;122:605-613.

(9) Marchionni N, Fattirolli F, Fumagalli S et al. Improved exercise tolerance and quality of life with cardiac rehabilitation of older patients after myocardial infarction: results of a randomized, controlled trial. *Circulation* 2003;107:2201-2206.

(10) Roger VL, Weston SA, Redfield MM et al. Trends in heart failure incidence and survival in a community-based population. *JAMA* 2004;292:344-350.

(11) Barker WH, Mullooly JP, Getchell W. Changing incidence and survival for heart failure in a well-defined older population, 1970-1974 and 1990-1994. *Circulation* 2006;113:799-805.

(12) Newman AB, Gottdiener JS, Mcburnie MA et al. Associations of subclinical cardiovascular disease with frailty. *J Gerontol A Biol Sci Med Sci* 2001;56:M158-M166.

(13) Bouillon K, Batty GD, Hamer M et al. Cardiovascular disease risk scores in identifying future frailty: the Whitehall II prospective cohort study. *Heart* 2013;99:737-742.

(14) Matsuzawa Y, Konishi M, Akiyama E et al. Association between gait speed as a measure of frailty and risk of cardiovascular events after myocardial infarction. *J Am Coll Cardiol* 2013;61:1964-1972.

(15) Fried LP, Kronmal RA, Newman AB et al. Risk factors for 5-year mortality in older adults: the Cardiovascular Health Study. *JAMA* 1998;279:585-592.

(16) Raggi C, Berardi AC. Mesenchymal stem cells, aging and regenerative medicine. *Muscles Ligaments Tendons J* 2012;2:239-242.

(17) Hare JM, Traverse JH, Henry TD et al. A randomized, double-blind, placebo-controlled, dose-escalation study of intravenous adult human mesenchymal stem cells (prochymal) after acute myocardial infarction. *J Am Coll Cardiol* 2009;54:2277-2286.

(18) Hare JM, Fishman JE, Gerstenblith G et al. Comparison of allogeneic vs autologous bone marrow-derived mesenchymal stem cells delivered by transendocardial injection in patients with ischemic cardiomyopathy: the POSEIDON randomized trial. *JAMA* 2012;308:2369-2379.

(19) Weiss DJ, Casaburi R, Flannery R, Leroux-Williams M, Tashkin DP. A Placebo-Controlled, Randomized Trial of Mesenchymal Stem Cells in COPD. *Chest* 2013;143:1590-1598.

(20) Pittenger MF, Mackay AM, Beck SC et al. Multilineage potential of adult human mesenchymal stem cells. *Science* 1999;284:143-147.

(21) Ferreira-Martins J, Ogorek B, Cappetta D et al. Cardiomyogenesis in the developing heart is regulated by c-kit-positive cardiac stem cells. *Circ Res* 2012;110:701-715.

(22) Orlic D, Kajstura J, Chimenti S et al. Bone marrow cells regenerate infarcted myocardium. *Nature* 2001;410:701-705.

(23) Salven P, Mustjoki S, Alitalo R, Alitalo K, Rafii S. VEGFR-3 and CD133 identify a population of CD34+ lymphatic/vascular endothelial precursor cells. *Blood* 2003;101:168-172.

(24) Reya T, Morrison SJ, Clarke MF, Weissman IL. Stem cells, cancer, and cancer stem cells. *Nature* 2001;414:105-111.

(25) Asahara T, Murohara T, Sullivan A et al. Isolation of putative progenitor endothelial cells for angiogenesis. *Science* 1997;275:964-967.

(26) Asahara T, Masuda H, Takahashi T et al. Bone marrow origin of endothelial progenitor cells responsible for postnatal vasculogenesis in physiological and pathological neovascularization. *Circ Res* 1999;85:221-228.

(27) Gehling UM, Ergun S, Schumacher U et al. In vitro differentiation of endothelial cells from AC133-positive progenitor cells. *Blood* 2000;95:3106-3112.

(28) Peichev M, Naiyer AJ, Pereira D et al. Expression of VEGFR-2 and AC133 by circulating human CD34(+) cells identifies a population of functional endothelial precursors. *Blood* 2000;95:952-958.

(29) Huss R. Isolation of primary and immortalized CD34-hematopoietic and mesenchymal stem cells from various sources. *Stem Cells* 2000;18:1-9.

(30) Murry CE, Soonpaa MH, Reinecke H et al. Haematopoietic stem cells do not transdifferentiate into cardiac myocytes in myocardial infarcts. *Nature* 2004;428:664-668.

(31) Le BK, Frassoni F, Ball L et al. Mesenchymal stem cells for treatment of steroid-resistant, severe, acute graft-versus-host disease: a phase II study. *Lancet* 2008;371:1579-1586.

(32) Lee RH, Seo MJ, Reger RL et al. Multipotent stromal cells from human marrow home to and promote repair of pancreatic islets and renal glomeruli in diabetic NOD/scid mice. *Proc Natl Acad Sci U S A* 2006;103:17438-17443.

(33) Nemeth K, Leelahavanichkul A, Yuen PS et al. Bone marrow stromal cells attenuate sepsis via prostaglandin E(2)-dependent reprogramming of host macrophages to increase their interleukin-10 production. *Nat Med* 2009;15:42-49.

(34) Parekkadan B, van PD, Suganuma K et al. Mesenchymal stem cell-derived molecules reverse fulminant hepatic failure. *PLoS One* 2007;2:e941.

(35) Togel F, Hu Z, Weiss K, Isaac J, Lange C, Westenfelder C. Administered mesenchymal stem cells protect against ischemic acute renal failure through differentiation-independent mechanisms. *Am J Physiol Renal Physiol* 2005;289:F31-F42.

(36) Iso Y, Spees JL, Serrano C et al. Multipotent human stromal cells improve cardiac function after myocardial infarction in mice without long-term engraftment. *Biochem Biophys Res Commun* 2007;354:700-706.

(37) Schuleri KH, Feigenbaum GS, Centola M et al. Autologous mesenchymal stem cells produce reverse remodelling in chronic ischaemic cardiomyopathy. *Eur Heart J* 2009;30:2722-2732.

(38) Miyahara Y, Nagaya N, Kataoka M et al. Monolayered mesenchymal stem cells repair scarred myocardium after myocardial infarction. *Nat Med* 2006;12:459-465.

(39) Gong Z, Niklason LE. Use of human mesenchymal stem cells as alternative source of smooth muscle cells in vessel engineering. *Methods Mol Biol* 2011;698:279-294.

(40) Price AP, England KA, Matson AM, Blazar BR, Panoskaltsis-Mortari A. Development of a decellularized lung bioreactor system for bioengineering the lung: the matrix reloaded. *Tissue Eng Part A* 2010;16:2581-2591.

(41) Williams AR, Hare JM. Mesenchymal stem cells: biology, pathophysiology, translational findings, and therapeutic implications for cardiac disease. *Circ Res* 2011;109:923-940.

(42) Mangi AA, Noiseux N, Kong D et al. Mesenchymal stem cells modified with Akt prevent remodeling and restore performance of infarcted hearts. *Nat Med* 2003;9:1195-1201.

(43) Williams AR, Trachtenberg B, Velazquez DL et al. Intramyocardial stem cell injection in patients with ischemic cardiomyopathy: functional recovery and reverse remodeling. *Circ Res* 2011;108:792-796.

(44) Ishizawa K, Kubo H, Yamada M et al. Bone marrow-derived cells contribute to lung regeneration after elastase-induced pulmonary emphysema. *FEBS Lett* 2004;556:249-252.

(45) Spees JL, Pociask DA, Sullivan DE et al. Engraftment of bone marrow progenitor cells in a rat model of asbestos-induced pulmonary fibrosis. *Am J Respir Crit Care Med* 2007;176:385-394.

(46) Spees JL, Whitney MJ, Sullivan DE et al. Bone marrow progenitor cells contribute to repair and remodeling of the lung and heart in a rat model of progressive pulmonary hypertension. *FASEB J* 2008;22:1226-1236.

(47) Bonfield TL, Koloze M, Lennon DP, Zuchowski B, Yang SE, Caplan AI. Human mesenchymal stem cells suppress chronic airway inflammation in the murine ovalbumin asthma model. *Am J Physiol Lung Cell Mol Physiol* 2010;299:L760-L770.

(48) Lee JW, Fang X, Gupta N, Serikov V, Matthay MA. Allogeneic human mesenchymal stem cells for treatment of E. coli endotoxin-induced acute lung injury in the ex vivo perfused human lung. *Proc Natl Acad Sci U S A* 2009;106:16357-16362.

(49) Gao J, Dennis JE, Muzic RF, Lundberg M, Caplan AI. The dynamic in vivo distribution of bone marrow-derived mesenchymal stem cells after infusion. *Cells Tissues Organs* 2001;169:12-20.

(50) Cargnoni A, Gibelli L, Tosini A et al. Transplantation of allogeneic and xenogeneic placenta-derived cells reduces bleomycin-induced lung fibrosis. *Cell Transplant* 2009;18:405-422.

(51) Ortiz LA, Gambelli F, McBride C et al. Mesenchymal stem cell engraftment in lung is enhanced in response to bleomycin exposure and ameliorates its fibrotic effects. *Proc Natl Acad Sci U S A* 2003;100:8407-8411.

(52) Moodley Y, Atienza D, Manuelpillai U et al. Human umbilical cord mesenchymal stem cells reduce fibrosis of bleomycin-induced lung injury. *Am J Pathol* 2009;175:303-313.

(53) Ahn MH, Park BL, Lee SH et al. A promoter SNP rs4073T>A in the common allele of the interleukin 8 gene is associated with the development of idiopathic pulmonary fibrosis via the IL-8 protein enhancing mode. *Respir Res* 2011;12:73.

(54) Kim DY, Kwon EY, Hong GU, Lee YS, Lee SH, Ro JY. Cigarette smoke exacerbates mouse allergic asthma through Smad proteins expressed in mast cells. *Respir Res* 2011;12:49.

(55) Lee SH, Jang AS, Kim YE et al. Modulation of cytokine and nitric oxide by mesenchymal stem cell transfer in lung injury/fibrosis. *Respir Res* 2010;11:16.

(56) Rojas M, Xu J, Woods CR et al. Bone marrow-derived mesenchymal stem cells in repair of the injured lung. *Am J Respir Cell Mol Biol* 2005;33:145-152.

(57) Jiang Y, Jahagirdar BN, Reinhardt RL et al. Pluripotency of mesenchymal stem cells derived from adult marrow. *Nature* 2002;418:41-49.

(58) Kotton DN, Ma BY, Cardoso WV et al. Bone marrow-derived cells as progenitors of lung alveolar epithelium. *Development* 2001;128:5181-5188.

(59) Yamada M, Kubo H, Kobayashi S et al. Bone marrow-derived progenitor cells are important for lung repair after lipopolysaccharide-induced lung injury. *J Immunol* 2004;172:1266-1272.

(60) Chang JC, Summer R, Sun X, Fitzsimmons K, Fine A. Evidence that bone marrow cells do not contribute to the alveolar epithelium. *Am J Respir Cell Mol Biol* 2005;33:335-342.

(61) Kotton DN, Fabian AJ, Mulligan RC. Failure of bone marrow to reconstitute lung epithelium. *Am J Respir Cell Mol Biol* 2005;33:328-334.

(62) Amado LC, Saliaris AP, Schuleri KH et al. Cardiac repair with intramyocardial injection of allogeneic mesenchymal stem cells after myocardial infarction. *Proc Natl Acad Sci U S A* 2005;102:11474-11479.

(63) Shake JG, Gruber PJ, Baumgartner WA et al. Mesenchymal stem cell implantation in a swine myocardial infarct model: engraftment and functional effects. *Ann Thorac Surg* 2002;73:1919-1925.

(64) Kocher AA, Schuster MD, Szabolcs MJ et al. Neovascularization of ischemic myocardium by human bone-marrow-derived angioblasts prevents cardiomyocyte apoptosis, reduces remodeling and improves cardiac function. *Nat Med* 2001;7:430-436.

(65) Toma C, Pittenger MF, Cahill KS, Byrne BJ, Kessler PD. Human mesenchymal stem cells differentiate to a cardiomyocyte phenotype in the adult murine heart. *Circulation* 2002;105:93-98.

(66) Hatzistergos KE, Quevedo H, Oskouei BN et al. Bone marrow mesenchymal stem cells stimulate cardiac stem cell proliferation and differentiation. *Circ Res* 2010;107:913-922.

(67) Beltrami AP, Urbanek K, Kajstura J et al. Evidence that human cardiac myocytes divide after myocardial infarction. *N Engl J Med* 2001;344:1750-1757.

(68) Kraitchman DL, Wilke N, Hexeberg E et al. Myocardial perfusion and function in dogs with moderate coronary stenosis. *Magn Reson Med* 1996;35:771-780.

(69) Kraitchman DL, Young AA, Bloomgarden DC et al. Integrated MRI assessment of regional function and perfusion in canine myocardial infarction. *Magn Reson Med* 1998;40:311-326.

(70) Garot J, Bluemke DA, Osman NF et al. Transmural contractile reserve after reperfused myocardial infarction in dogs. *J Am Coll Cardiol* 2000;36:2339-2346.

(71) Kraitchman DL, Bluemke DA, Chin BB, Heldman AW, Heldman AW. A minimally invasive method for creating coronary stenosis in a swine model for MRI and SPECT imaging. *Invest Radiol* 2000;35:445-451.

(72) Serfaty JM, Atalar E, Declerck J et al. Real-time projection MR angiography: feasibility study. *Radiology* 2000;217:290-295.

(73) Yang X, Atalar E. Intravascular MR imaging-guided balloon angioplasty with an MR imaging guide wire: feasibility study in rabbits. *Radiology* 2000;217:501-506.

(74) Osman NF, McVeigh ER, Prince JL. Imaging heart motion using harmonic phase MRI. *IEEE Trans Med Imaging* 2000;19:186-202.

(75) Osman NF, Prince JL. Visualizing myocardial function using HARP MRI. *Phys Med Biol* 2000;45:1665-1682.

(76) Garot J, Bluemke DA, Osman NF et al. Fast determination of regional myocardial strain fields from tagged cardiac images using harmonic phase MRI. *Circulation* 2000;101:981-988.

(77) Jones JR, Mata JF, Yang Z, French BA, Oshinski JN. Left ventricular remodeling subsequent to reperfused myocardial infarction: evaluation of a rat model using cardiac magnetic resonance imaging. *J Cardiovasc Magn Reson* 2002;4:317-326.

(78) Wise RG, Huang CL, Al-Shafei AI, Carpenter TA, Hall LD. Geometrical models of left ventricular contraction from MRI of the normal and spontaneously hypertensive rat heart. *Phys Med Biol* 1999;44:2657-2676.

(79) Liang J, Zhang H, Hua B et al. Allogenic mesenchymal stem cells transplantation in refractory systemic lupus erythematosus: a pilot clinical study. *Ann Rheum Dis* 2010;69:1423-1429.

(80) Le BK, Frassoni F, Ball L et al. Mesenchymal stem cells for treatment of steroid-resistant, severe, acute graft-versus-host disease: a phase II study. *Lancet* 2008;371:1579-1586.

(81) Psaltis PJ, Zannettino AC, Worthley SG, Gronthos S. Concise review: mesenchymal stromal cells: potential for cardiovascular repair. *Stem Cells* 2008;26:2201-2210.

(82) Schuleri KH, Amado LC, Boyle AJ et al. Early improvement in cardiac tissue perfusion due to mesenchymal stem cells. *Am J Physiol Heart Circ Physiol* 2008;294:H2002-H2011.

(83) Schuleri KH, Feigenbaum GS, Centola M et al. Autologous mesenchymal stem cells produce reverse remodelling in chronic ischaemic cardiomyopathy. *Eur Heart J* 2009;30:2722-2732.

(84) Quevedo HC, Hatzistergos KE, Oskouei BN et al. Allogeneic mesenchymal stem cells restore cardiac function in chronic ischemic cardiomyopathy via trilineage differentiating capacity. *Proc Natl Acad Sci U S A* 2009;106:14022-14027.

(85) Williams AR, Suncion VY, McCall F et al. Durable scar size reduction due to allogeneic mesenchymal stem cell therapy regulates whole-chamber remodeling. *J Am Heart Assoc* 2013;2:e000140.

(86) Liang J, Zhang H, Hua B et al. Allogenic mesenchymal stem cells transplantation in refractory systemic lupus erythematosus: a pilot clinical study. *Ann Rheum Dis* 2010;69:1423-1429.

(87) Kinkaid HY, Huang XP, Li RK, Weisel RD. What's new in cardiac cell therapy? Allogeneic bone marrow stromal cells as "universal donor cells". *J Card Surg* 2010;25:359-366.

(88) Zhuo Y, Li SH, Chen MS et al. Aging impairs the angiogenic response to ischemic injury and the activity of implanted cells: combined consequences for cell therapy in older recipients. *J Thorac Cardiovasc Surg* 2010;139:1286-94, 1294.

(89) Castillo E AL, Gerber B et al. Myocardial Infarction size assessment with single breath-hold three-dimensional inversion-recovery-prepared MRI. J Cardiovasc Magn Reson. 2002;4:25.

(90) Foo T CE, Kraitchman DL, Wu K, Bluemke DA, Lima JA. Three dimensional breath-held imaging of myocardial infarction using variable sampling in time (VAST). J Cardiovasc Magn Reson. 2002;4:123

Interdisciplinary Stem Cell Institute

University of Miami/ Miller School of Medicine

Clinical Research Protocol

**Study Title:**	A Phase I/II, Randomized, Blinded and Placebo-controlled Trial to Evaluate the Safety and Potential Efficacy of Allogeneic Human Mesenchymal Stem Cell Infusion in Patients with Aging Frailty.<br><br>***A*llogenei*C* Human Mesenchymal *S*tem Ce*l*ls (hMSC) in Patients with Aging F*RA*il*T*y via Intraveno*US* Delivery (CRATUS)**
**Study Product:**	Allogeneic Human Mesenchymal Stem Cells (hMSCs)
**Indication:**	Aging Frailty
**FDA IND No.:**	BB-IND #15679
**Principal Investigator:**	Joshua M. Hare, M.D. Telephone: 305-243-5579
**Protocol Version:**	November 30, 2016, Version 5.3

Protocol Agreement Signature:

Darcy L. DiFede, RN, BSN, FAHA
Sponsor's Authorized Representative
On Behalf of Joshua M. Hare, MD;
Study Sponsor

11-30-2016
Date

CONFIDENTIALITY STATEMENT

This document is confidential and proprietary to the Interdisciplinary Stem Cell Institute University of Miami/Miller School of Medicine and its affiliates. Acceptance of this document constitutes agreement by the recipient that no unpublished information contained herein will be reproduced, published, or otherwise disseminated or disclosed without prior written approval of the Interdisciplinary Stem Cell Institute University of Miami or its affiliates, except that this document may be disclosed in any medium to appropriate clinical investigators, Institutional Review Boards, and others directly involved in the clinical investigation that is the subject of this information under the condition that they keep the information strictly confidential.

Table of Contents

List of Abbreviations 9
Protocol Synopsis 11
1. INTRODUCTION 14
1.1 Background 14
1.2 Mesenchymal Stem Cells 16
1.3 Mesenchymal Stem Cells: Preclinical Experience 17
1.4 Allogeneic Mesenchymal Stem Cells: Previous Experience in Humans 27
1.5. Pharmacology and Toxicology Studies of Mesenchymal Stem Cells 34
2. STUDY OBJECTIVES AND ENDPOINTS 36
2.1 Study Objectives 36
2.1.1 Primary Objective 36
2.1.2 Secondary Objectives 36
2.2 Study Endpoints 36
2.2.1 Primary Endpoints (Safety) 36
2.2.2. Secondary Endpoints (Efficacy) 37
3. STUDY DESIGN 37
3.1 Description of the Study 37
3.2. RANDOMIZATION STUDY 38
4. SUBJECT SELECTION 38
4.1 Inclusion Criteria 38
4.2 Exclusion Criteria 39
4.3 Concomitant Treatments, Procedures, and Nondrug Therapies 40
4.4 Withdrawal Criteria 40
5. MESENCHYMAL STEM CELL DONORS 40
5.1 Bone Marrow Aspiration for Generation of MSCs 41
5.2 Normal Donor Eligibility 41
5.3 Donor Consent 42
5.4 Follow-up Schedule for Donors 42
5.5 Biomarker Assessment 43
6. TREATMENT OF SUBJECTS 43

6.1 Study Investigational Product 43
6.2 Dosing 43
6.3 Dosage Rationale 44
6.4 Administration Rate 44
6.4.1. Infusion Monitoring 45
6.5 Concomitant Therapy 45
6.5.1 Permitted therapy 45
6.5.2 Excluded therapy 46
6.5.3. Subject monitoring 46
6.6 Blinding and Unblinding 46
6.7 Study Investigational Therapy Management 46
6.7.1 Investigational Product Labeling and Storage 46
6.7.2 Investigational Product Accountability Procedures 47
7. STUDY PROCEDURES 47
7.1. Time and Events Schedule 47
7.2.1 Screening Visit 50
7.2.2 Baseline Visit 50
7.2.3 Day 1 Visit 52
7.2.4 Week 2 Visit 52
7.2.5 Month 1 – Month 6 Visits 52
7.2.6 Month 12 Visit 52
7.2.7 Biomarker Assessment 52
7.2.8 Immune Monitoring for Graft Rejection 53
8. SAFETY 54
8.1 Safety Variables 54
8.2 Laboratory Evaluations 54
8.2.1 Pulse Oximetry 54
8.2.2 Pregnancy 54
8.2.3 Determination of Infusional Toxicity 55
8.2.4 Subject Stopping Guidelines 55
8.3 Definition of an Adverse Event 57
8.4 Definition of Adverse Reaction 57
8.5 Definition of Suspected Adverse Reaction 58
8.6 Definition of Serious 58

8.7 Definition of Unexpected ... 58
8.8 Clinical Laboratory Assessments and Other Abnormal Assessments as Adverse Events and Serious Adverse Events ... 59
8.9 Recording of Adverse Events and Serious Adverse Events ... 59
8.10 Intensity of Adverse Events and Serious Adverse Events ... 59
8.11 Causality of Adverse Events and Serious Adverse Events ... 60
8.12 Follow-Up of Adverse Events and Serious Adverse Events ... 61
8.13 Timeframes for Submitting SAE Reports ... 61
8.14 Post-Study Adverse Events and Serious Adverse Events ... 62
8.15 Regulatory Aspects of Adverse Event Reporting ... 62
9. STATISTICAL ANALYSIS ... 63
9.1 Determination of Sample Size and Analysis Population ... 63
9.2 General Statistical Methods ... 63
9.3 Interim Analyses ... 63
9.4 Data Safety Monitoring Board (DSMB) ... 64
9.4.1. ROLE OF THE DSMB ... 64
9.4.2. Purpose of the DSMB ... 64
9.4.3 COMMITTEE MEETINGS ... 66
9.4.4 DATA FLOW ... 70
9.4.5 COMMUNICATION ... 70
10. STUDY ADMINISTRATION ... 71
10.1 Regulatory Authority Approval ... 71
10.2 Ethics Approval ... 71
10.3 Subject Informed Consent ... 72
10.4 Confidentiality of Information ... 72
10.5 Payments to Subjects ... 73
APPENDIX 1: Infusion Guidelines ... 74
Addendum A: Pilot Subjects Optional Follow-on Phase ... 77
A.1 Rationale and Description of second infusion for first 15 subjects on pilot phase ... 77
A.2 Demonstrated Safety and Increased Efficacy of Multiple Infusions of Allogeneic Mesenchymal Stem Cells ... 77
A.3 STUDY OBJECTIVES AND ENDPOINTS ... 78
A.3.1 Study Objectives ... 78
A.3.1.1 Primary Objective ... 78

A.3.1.2 Secondary Objectives ... 78
A.3.2 Study Endpoints ... 79
A.3.2.1 Primary Endpoints (Safety) ... 79
A.3.2.2 Secondary Endpoints (Efficacy) ... 79
A.4 Inclusion and Exclusion Criteria ... 80
A.4.1 Inclusion Criteria for Follow-on Phase ... 80
A.4.2 Exclusion Criteria for Follow-on Phase ... 80
A.5 Dosing ... 81
A.6 Dosage Rationale ... 82
A.7 Administration Rate ... 82
A.8 Data Safety Monitoring Board (DSMB) ... 82
A.13 Payments to Subjects in the Follow-on Phase ... 83
Time and Events Table Key: ... 85
A.15 Study Visits ... 86
A.15.1 Screening Visit for second infusion (Applicable to pilot subjects) ... 86
A.15.2 Baseline Visit for second infusion (Applicable to pilot subjects) ... 86
A.15.3 Day 1 Visit for second infusion (Applicable to pilot subjects) ... 87
A.15.4 Month 1 Visit for second infusion (Applicable to pilot subjects) ... 87
A.15.5 Month 3 and Month 6 Visit for second infusion (Applicable to pilot subjects) . 87
A.16 Statistical Considerations ... 88
A.17 Safety Monitoring of 30-Day Rate of TE-SAEs ... 88
Addendum B: Penicillin/streptomycin free cell safety study ... 89
B.1 Rationale and Description for penicillin/streptomycin free cell safety study ... 89
B.2 STUDY OBJECTIVES AND ENDPOINTS ... 90
B.2.1 Study Objectives ... 90
B.2.1.1 Primary Objective ... 90
B.2.1.2 Secondary Objectives ... 90
B.2.2 Study Endpoints ... 90
B.2.2.1 Primary Endpoints (Safety) ... 90
B.2.2.2 Secondary Endpoints (Efficacy) ... 90
B.3 Inclusion and Exclusion Criteria ... 91
B.3.1 Inclusion Criteria for penicillin/streptomycin free cell safety study ... 91
B.3.2 Exclusion Criteria for penicillin/streptomycin free cell safety ... 91
B.4 Dosing ... 93

B.5 Dosage Rationale ... 93
B.6 Administration Rate ... 93
B.7 Data Safety Monitoring Board (DSMB) ... 94
B.12 Payments to Subjects in the Follow-on Phase ... 94
Time and Events Table Key: ... 96
B.14 Study Visits ... 97
B.14.1 Screening Visit ... 97
B.14.2 Baseline Visit ... 97
B.14.3 Day 1 Visit ... 98
B.14.4 Month 1 Visit ... 98
B.14.5 Month 3 and Month 6 Visit ... 98
B.15 Statistical Considerations ... 99
B.16 Safety Monitoring of 30-Day Rate of TE-SAEs ... 99
Addendum C: Pilot and Penicillin/Streptomycin Free Subjects Optional Follow-on Phase ... 101
C.1 Rationale and Description of additional infusions for subjects who have received one or two infusions in the phase I open-label phases of this protocol. ... 101
C.2 Demonstrated Safety and Increased Efficacy of Multiple Infusions of Allogeneic Mesenchymal Stem Cells ... 101
C.3 STUDY OBJECTIVES AND ENDPOINTS ... 103
C.3.1 Study Objectives ... 103
C.3.1.1 Primary Objective ... 103
C.3.1.2 Secondary Objectives ... 103
C.3.2 Study Endpoints ... 103
C.3.2.1 Primary Endpoints (Safety) ... 103
C.3.2.2 Secondary Endpoints (Efficacy) ... 104
C.4 Inclusion and Exclusion Criteria ... 104
C.4.1 Inclusion Criteria for Follow-on Phase ... 104
C.4.2 Exclusion Criteria for Follow-on Phase ... 105
C.5 Dosing ... 106
C.6 Dosage Rationale ... 106
C.7 Administration Rate ... 106
C.8 Data Safety Monitoring Board (DSMB) ... 107
C.13 Payments to Subjects ... 107

Time and Events Table Key: 109
C.15 Study Visits 110
C.15.1 Screening Visit for additional infusion 110
C.15.2 Baseline Visit for additional infusion 110
C.15.3 Day 1 Visit for additional infusion 111
C.15.4 Month 1 Visit for additional infusion 111
C.15.5 Month 3 and Month 6 Visit for additional infusion 111
C.16 Statistical Considerations 112
11. Reference List 113

Authorship Team (alphabetical order)

The following individuals provided substantial input during protocol development:

Darcy L. DiFede, RN, BSN University of Miami

Joshua M. Hare, MD* University of Miami

Marietsy V. Pujol, MBA University of Miami

* Principal investigator

List of Abbreviations

AE	Adverse event
ahMSCs	Allogeneic human Mesenchymal Stem Cells
Allo	allogeneic
ALT	Alanine aminotransferase
AST	Aspartate aminotransferase
BM	Bone Marrow
CBC	Complete Blood Count
CFR	Code of Federal Regulations
CHAMPS	Community Healthy Activities Model Program for Seniors questionnaire
CMV	Cytomegalovirus
CPF	Cell Processing Facility
CPL	Cell Processing Laboratory
CRP	C-Reactive Protein
CT	Computed tomography
DLCO	Diffusing Capacity
DMSO	Dimethyl sulfoxide
DSMB	Data Safety Monitoring Board
ECC	Eluerian Circumferential strain
EDV	End-diastolic volume
EPCs	Endothelial progenitor cells
ESR	Expedited safety report
ESV	End-systolic volume
FBS	Fetal Bovine Serum
FDA	Food and Drug Administration
FEV – 1	Forced expiratory volume in 1 second
FSH	Follicle stimulating hormone
FVC	Forced vital capacity
GCP	Good Clinical Practice
G-CSF	Granulocyte colony stimulating factor
GFP	Green fluorescent protein
GM – CSF	Granulocyte Macrophage Colony Stimulating Factor
GVHD	Graft versus host disease
HBcAb	Anti-Hepatitis B core antibody
HCV Ab	Anti-Hepatitis C virus antibody
HIPAA	Health Insurance Portability and Accountability Act Authorization
HIV	Human Immunodeficiency Virus
HLA	Human leukocyte antigen
Has	Human serum albumin
hMSCs	Human mesenchymal stem cell

HSCs	Hematopoietic stem cells
HTLV	Human T-lymphotropic Virus
ICF	Informed Consent Form
ICH	International Conference on Harmonization
IL-6	Interleukin-6
IND	Investigational new drug
INR	International Normalized Ratio
IP	Investigational Product
IRB /IEC	Institutional Review Board
IV	Intravenous Infusion
LAD	Left anterior descending
LFT	Liver Function tests
LV	Left ventricular
MFI	Multi-dimensional Fatigue Inventory
MI	Myocardial Infarction
MMSE	Mini Mental State Examination
MNC	Mononuclear Cell
MRI	Magnetic resonance imaging
MSCs	Mesenchymal Stem Cells
NAT	Nucleic Acid Testing
NIH	National Institute of Health
NMDP	National Marrow Donor Program
PBMC	Peripheral blood mononuclear cells
PFTs	Pulmonary function tests
PSURs	Periodic Safety Update Reports
QOL	Quality of life
RDW	Red blood cell distribution
RPR	Rapid Plasma Raegin
SAE	Serious Adverse Event
SAP	Statistical Analysis Plan
SCA-1	Stem cell factor antigen
SF – 36	Short Form – 36
SGRQ	St. George's Respiratory Questionnaire
SPPB	Standard Physical Performance Battery
TE-SAE	Treatment-emergent serious adverse event
TNF-α	Tumor necrosis factor-alpha
TTC	Triphenyltetrazolium cholride
VEGFR2	Vascular endothelial growth factor
WBC	White blood cell
WNV	West Nile virus
6MWT	Six minute walk test

Protocol Synopsis

PRODUCT	Intravenous Allogeneic Adult Human Mesenchymal Stem Cells (MSCs)
PHASE OF DEVELOPMENT	I/II
MAIN CRITERIA FOR INCLUSION	Aging Frailty
STUDY OBJECTIVES	To demonstrate the safety of allogeneic hMSCs administered in subjects' with Frailty and to explore treatment efficacy (decrease in frailty, frequency of acute exacerbations, change in symptom related quality of life, improved cardiovascular status, decrease in inflammatory biomarkers, endothelial function and 1 year survival).
STUDY DESIGN	A Phase I/II, Randomized, Blinded and Placebo-controlled
INVESTIGATIONAL PLAN	Before initiating the full randomized study, a Pilot Safety Phase will be performed. The randomized portion of this trial will be conducted after a full review of the safety data from the Pilot Phase by the DSMB.<br><br>Following the Pilot Phase of fifteen (15) subjects, thirty (30) subjects are scheduled to undergo infusion and meeting all inclusion/exclusion criteria will be evaluated at baseline.<br><br><u>Pilot Phase</u> (15 subjects)<br><br><u>Group 1 (5 subjects)</u>:<br><br>Five (5) subjects will be treated with a single administration of allogeneic hMSCs: 2×10^7 (20 million) cells delivered via peripheral intravenous infusion.<br><br><u>Group 2 (5 subjects)</u>:<br><br>Five (5) subjects will be treated with a single administration of allogeneic hMSCs: 1×10^8 (100 million) cells delivered via peripheral intravenous infusion.<br><br><u>Group 3 (5 subjects)</u>:<br><br>Five (5) subjects will be treated with a single administration of allogeneic hMSCs: 2×10^8 (200 million) cells delivered via peripheral intravenous infusion.<br><br>At the pilot subjects one year phone call visit, all fifteen (15) subjects will be provided with the option of having additional administrations of allogeneic hMSCs: 1×10^8 (100 million) cells delivered via peripheral intravenous infusion.

	In the randomized phase of allo-hMSCs or matched placebo up to 30 subjects will be randomized in a 1:1:1 ratio to one of two doses of MSCs versus placebo.<br><br>The first three (3) subjects in each treatment group will not be treated less than 5 days apart and will each undergo full evaluation for 5 days to demonstrate there is no evidence of treatment emergent SAE's prior to proceeding with the treatment of further subjects.<br><br><u>Treatment Strategies</u> following successful completion of the Pilot Phase.<br><br><u>Group A</u> (10 subjects) – Allogeneic hMSCs: 100 million cells/ml delivered via peripheral intravenous infusion.<br><br><u>Group B</u> (10 subjects) – Allogeneic hMSCs: 200 million cells/ml delivered via peripheral intravenous infusion.<br><br><u>Group C</u> (10 subjects) - Placebo delivered via peripheral intravenous infusion.<br><br>The Allo-hMSCs will be supplied from an allogeneic human mesenchymal stem cell source manufactured by the University of Miami.<br><br>Following infusion, subjects will be followed at 2 week's post-infusion, and at one, three and six months to complete all safety and efficacy assessments. Subjects will also have a final twelve-month contact for assessment of vital status and occurrence of hospitalization.
ROUTE OF ADMINISTRATION	Peripheral Intravenous Infusion
DURATION OF STUDY PARTICIPATION	12 months (Follow-up will be at 2 weeks, 1, 3, 6, and 12 months.) Pilot subjects will have an optional additional 12 month follow-up period for a second infusion.
SUBJECT POPULATION	Forty-Five (45) subjects with frailty will be enrolled in the study. Only 45 subjects will be included in the statistical analysis plan.
Definition of Endpoints	<u>**Safety (Primary):**</u> Incidence (at one month post-infusion) of any treatment-emergent serious adverse events (TE-SAEs), defined as the composite of: death, non-fatal pulmonary embolism, stroke, hospitalization for worsening dyspnea and clinically significant laboratory test abnormalities determined per the Investigator's judgment.<br><br><u>**Efficacy (Secondary): During the baseline, 3 and 6 month visits.**</u>

	• Difference in rate of change of frailty defined as:<br>- Reduced Activity (assessed via CHAMPS questionnaire)<br>- Slowing of Mobility (assessed via gait speed test and SPPB assessment)<br>- Weight Loss<br>- Diminished handgrip strength (assessed via dynamometer)<br>- Exhaustion (assessed via the MFI questionnaire)<br>- Decrease in subject quality of life assessment(s)<br>• Death from any cause.<br>• Change between baseline and 6 months in dobutamine stress echo induced ejection fraction<br>• Change between baseline and 6 months the following panel of inflammatory markers: CRP, IL-6, D-dimer, fibrinogen, CBC with differential, DNA, and TNFα
Safety (Additional)	• During the 12 week follow-up period and each consecutive time-point up until the final visit
Inclusion Criteria	1. Provide written informed consent.<br>2. Subjects age ≥60 and ≤95 years at the time of signing the Informed Consent Form.<br>3. Show signs of frailty apart from a concomitant condition as assessed by the Investigator with a frailty score of 4 to 7 using the Canadian Clinical Frailty Scale.<br>4. Female subjects must have an FSH ≥ 25.8 mIU/mL, if not currently on hormone replacement therapy.
Exclusion Criteria	1. Score of ≤24 on the Mini Mental State Examination (MMSE)<br>2. Be a female who is pregnant, nursing, or of childbearing potential while not practicing effective contraceptive methods. Female subjects must undergo a blood or urine pregnancy test at screening and within 36 hours prior to infusion.<br>3. Inability to perform any of the assessments required for endpoint analysis.<br>4. Active listing (or expected future listing) for transplant of any organ.<br>5. Clinically important abnormal screening laboratory values.<br>6. Serious comorbid illness or any other condition that, in the opinion of the investigator, may compromise the safety or compliance of the subject or preclude successful completion of the study.<br>7. Hypersensitivity to dimethyl sulfoxide (DMSO).

	8. Be an organ transplant recipient.<br>9. Have a clinical history of malignancy within 3 years (i.e., subjects with prior malignancy must be disease free for 3 years), except curatively-treated basal cell carcinoma, squamous cell carcinoma, melanoma in situ or cervical carcinoma, if recurrence occurs.<br>10. Have a non-pulmonary condition that limits lifespan to < 1 year.<br>11. Have a history of drug or alcohol abuse within the past 24 months.<br>12. Be serum positive for HIV, hepatitis BsAg or Viremic hepatitis C.<br>13. Be currently participating (or participated within the previous 30 days) in an investigational therapeutic or device trial.<br>14. Any other condition that, in the opinion of the investigator, may compromise the safety or compliance of the subject or preclude successful completion of the study.

1. INTRODUCTION

1.1 Background

Frailty in an aging population is defined as a medical syndrome “with multiple causes and contributors that is characterized by diminished strength, endurance, and reduced physiologic function that increases an individual’s vulnerability for developing increased dependency and/or death”[1]. Of great importance is that frailty, while not characterized as a disability, per se, does increase the risk of death in affected individuals[2-4]. Moreover, there is a close link between a subject’s health and frailty. These subjects tend to show a greater risk to frailty when there are other conditions affecting their physical and psychological well-being, such as high blood pressure, cancer, or cognitive impairment. In an aging population frailty is characterized by reduced physical activity, slowing of mobility, weight loss, diminished handgrip strength, and exhaustion[2, 3]. There are several well-validated models to assess frailty[1]For example, the FRAIL scoring index provides a clinical diagnosis of frailty when a subject exhibits three or more of these five characteristics[2;5]. The forward trajectory in medical advances, and a more health aware society, indicates that the population is living longer and requiring more care and services than were needed in the past. As such, the increase in frail elderly subjects has adversely increased the demand for healthcare services. Currently there is a favorable movement in researching a novel medical therapy for frailty as this remains an unmet need amongst the elderly population.[6] It is widely perceived that frailty can be favorably modified, and in this regard there is a major need for effective

management tools and treatment strategies. In this protocol, we will test the impact of a safe cell-based therapeutic on the frailty syndrome.

Frailty and Cardiovascular Performance

The aging cardiovascular system has some very specific phenotypic alterations[7;8]. These include left ventricular hypertrophy and a diminution in exercise induced increase in ejection fraction[9-11]. These characteristic abnormalities are hypothesized to contribute to specific symptoms of the frailty syndrome and to increase the morbidity and mortality from cardiovascular disease in elderly individuals.

Several studies document the increased risk for mortality in frail elderly subjects with cardiovascular events such as non-ST-segment elevation myocardial infarction (NSTEMI)[4;12]. Frail individuals have increased disease burden in and therefore more prolonged recuperation versus a non-frail subject[1]. There are additional associations between frailty and other cardiovascular diagnoses including angina, myocardial infarction, congestive heart failure and stroke. Gait speed is one symptom of frailty that is associated with cardiovascular mortality and an increased risk of cardiovascular events specifically STEMI subjects [13;14]. The Women's Health Initiative utilized the "Frailty Index" by Fried and colleagues[8] which was validated in the Cardiovascular Health Study[15] and the Womens Health and Aging Study. These studies demonstrated correlations in functional decline, increased risk of institutionalization and mortality with the use of these instruments.

The strong association between frailty and cardiovascular disease and the growing data base documenting safety and potential favorable effects of cell-based therapy in cardiovascular diseases provide justification for the assessment of potential benefits of cell therapy in subjects with frailty.

Inflammation in frailty

In addition and of great importance, there is a growing database of studies that highlight a connection between frailty and inflammation. For example, certain inflammatory markers, such as C-reactive protein (CRP), fibrinogen, interleukin-6 (IL-6), red blood cell distribution (RDW) and D-dimer are more likely to be elevated in frail as compared to non-frail individuals[8]. Importantly, among frail subjects, women exhibit higher concentrations of inflammatory and coagulation factors than men. CRP is an example of one marker studied that shows that women experiencing symptoms of frailty show a higher concentration of CRP. Differential white cell counts on the other hand exhibits an increased risk of frailty in both men and women. Dysregulated inflammation is a considerable key physiological marker in correlation with the frailty syndrome[8] . There is still insufficient data to show which markers specifically affect men or women[5;16]. It is believed that frailty can ultimately be prevented or attenuated, and the link between frailty and inflammation offers a potential therapeutic target, also addressable by cell therapy.

Frailty is assessed with several instruments. For example, the Clinical Frailty Scale[2] is a clinical scale that determines the degree of a subject's frailty based on the physician's judgment and the subject's medical information. This scale consists of 7 variables ranging from fit to complete functional dependence; If a subject displays three or more of the following symptoms: low physical activity, muscle weakness, slowed performance,

fatigue, unintentional weight loss, then the subject is considered to be classified as frail.[2;4]

Stem Cells in Frailty

An individual's endogenous stem cell production decreases with age; this decrease in an aging subject likely contributes to reduced ability to regenerate and repair organs and tissues. Several investigators have proposed that a regenerative treatment strategy could ameliorate signs and symptoms of aging frailty[8;16]. Currently, the FDA does not have any specific approved treatment for frail subjects and therefore no established standard of care. In many cases frailty can be masked by other physical or psychological conditions affecting well-being and functional status. The ultimate goal is to extend the health and ability of a subject to regenerate functionality. Allogeneic Human Mesenchymal Stem Cells are known to hone to sites of injury, reduce inflammation, and assist in cellular repair. Here we propose to study Allo-hMSCs (ahMSCs) as a novel therapy for treating subjects experiencing frailty.

There are specific features of the frailty syndrome that support a potential role of ahMSCs to ameliorate or improve frailty. Notably, ahMSCs are shown to improve cardiovascular status in subjects with acute MI[17] and heart failure[18]. In addition, ahMSCs are anti-inflammatory and reduce CRP levels in a sustained manner[19]. Importantly, data from heart failure studies show that ahMSCs are safe in subjects irrespective of age[18]. For these reasons, this protocol will test the safety of intravenous infusion (IV) of ahMSCs in individuals of advanced age with frailty, and will assess cardiovascular status and inflammatory markers in this population at increased risk for morbidity and mortality.

Cells derived from adult bone marrow

Bone marrow harbors a variety of cells that may contribute to vasculogenesis or cardiomyogenesis, either directly, or by facilitating endogenous repair mechanisms. Bone marrow cells have been prepared on the basis of being 1.) endothelial precursor cells that are $CD34^+$, 2.) MSCs purified without an antigen panning technique on the basis of their fibroblast morphology, ability to divide in culture and to differentiate into mesodermal lineages[20], and 3.) cells that express stem cell factor receptor, c-Kit[21;22]. Endothelial progenitor cells (EPCs) express the surface markers CD34, CD133, c-kit, and the vascular endothelial growth factor receptor-2 (VEGFR2; KDR; Flk-1)[23-28]. Hematopoietic stem cells (HSCs) exhibit self-renewal and differentiation. Their cell-surface phenotype is $CD34^+$, stem cell factor antigen $(SCA\text{-}1)^+$, $c\text{-}kit^+$, and Lin^- (review[29]). While there has been controversy regarding the ability of bone marrow-derived cells to transdifferentiate into cardiomyocytes[30], clinical trials of bone marrow therapies continue to suggest potential benefit in terms of improving a subject's well-being.

1.2 Mesenchymal Stem Cells

Mesenchymal stem cells (MSCs) are multipotent cells capable of differentiating into a number of different cell lines. Because of their unique combination of multipotency, migratory ability, and immunoprivileged state (MSCs do not express major histocompatibility factor-II making allogeneic transplant possible)[31], interest has abounded regarding their potential therapeutic and regenerative applications. In fact,

MSCs have been shown to hold promise as a novel therapeutic agent in multiple disease processes. Treatment with MSCs has been shown to ameliorate severe graft versus host disease[31], contribute to pancreatic islet and renal glomerular repair in diabetes[32], attenuate sepsis[33], reverse fulminant hepatic failure[34], protect against ischemic acute renal failure[35], reverse remodeling[36-38] and improve cardiac function after myocardial infarction[17], to be a potential source of multiple cell types for use in tissue engineering[39;40], and to be capable of tissue regeneration after spinal cord trauma, stroke, and connective tissue injury[41-43].

In the lung, MSCs have been shown to contribute to tissue regeneration after elastase-induced emphysema[44], home to sites of asbestos induced lung injury[45], contribute to tissue remodeling in a rat monocrotaline model of pulmonary hypertension[46], decrease chronic airway inflammation in a murine ovalbumin model of asthma[47], and to restore alveolar fluid balance after endotoxin induced acute lung injury[48].

Tracking of radioactively labeled cells shows that when administered intravenously, MSCs localize primarily to the lung, followed by the liver, and then other organs[49]. A number of studies show that MSCs preferentially home to sites of injury in the lung and contribute to tissue regeneration and repair[50-56]. Using Y-chromosome fluorescence in-situ hybridization, Y-chromosome positive male MSCs can be found at sites of lung injury in transplanted female mice[51;52;55]. These male MSCs appear to adopt anepithelial cell morphology, suggesting that they contribute to tissue regeneration either by fusion with resident epithelial cells or by mesenchymal to epithelial transition[51].

The ability of MSCs to differentiate towards an epithelial lineage was established by studies showing that they are capable of differentiation not only to cells of mesodermal origin, but also to cells of endodermal and ectodermal (including epithelial) origin[57;58]. MSCs cultured in airway growth media differentially express lung specific epithelial markers like Clara cell secretory protein, surfactant protein-C, and thyroid transcription factor-1[57;58]. In addition, in-situ hybridization studies with co-staining for green fluorescent protein (GFP) and epithelial markers shows that GFP-labeled MSCs assume an epithelial phenotype at sites of lung injury and contribute to tissue repair[56;59]. It is worth noting, however, that not all authors agree, with some suggesting that technical difficulties associated with immunofluorescence microscopy have led to the false conclusion that MSCs contribute to alveolar epithelium[60;61].

1.3 Mesenchymal Stem Cells: Preclinical Experience

Several cell-based therapies results propose that infusion of mesenchymal stem cells is a safe and novel approach believed to be an effective strategy to decrease symptoms of frailty. Below we review the impact of MSCs on the cardiovascular system following injury; this provides support for an impact of MSCs in individuals with frailty.

A porcine model of anterior myocardial infarction was used to characterize the impact of cellular cardiomyoplasty on cardiac structure and function using hemodynamic, imaging, and histological analyses. A pig model was selected because of its anatomic similarity to the human heart. The following sections describe the safety and efficacy results obtained with this model[62]. Two distinct sets of studies were conducted, representing the early

treatment of acute myocardial infarction, as well as the treatment of chronic ischemic cardiomyopathy.

Allogeneic mesenchymal stem cell transplantation improves global cardiac function in a swine model of acute myocardial infarction*:* Previously published work demonstrated that autologous MSC transplantation in post-MI pigs improved cardiac function, with histological evidence of robust engraftment at 8 weeks, and differentiation to a myocyte-like phenotype[63]. Based on *in vitro* observations that MSCs lack the B-7 costimulatory molecule and may therefore be immune-privileged, the impact of *allogeneic* MSC transplantation in porcine MI was assessed. A 14 pig randomized, placebo-controlled study (MSCs vs. placebo) using the BioCardia Helical Infusion Catheter was performed to assess safety and efficacy of allogeneic transendocardial injections[62]. Farm pigs were chronically instrumented to measure left-ventricular pressure, dimension, and oxygen consumption, and were randomized to active treatment or placebo groups. Three days following MI, placebo (n=7) or $2X10^8$ allogeneic MSCs (n=7) labeled with Di-I and DAPI (both fluorescent dyes to aid histochemical identification) were injected percutaneously into infarcted myocardium of the left ventricular cavity using a helical injection needle catheter inserted through a steerable guide catheter (BioCardia, Inc.). All animals tolerated the catheter-based injections well. Animals were then studied on a weekly basis for 8 weeks to assess hemodynamics and to examine ventricular architecture. In treated animals, MSCs engrafted within the MI (Figure 1 a, b) and expressed several myocyte proteins, including α-actinin, phospholamban, tropomyosin, and troponin T (Figure 1 c, d, e, f). In addition, there was evidence of stem-cell differentiation or incorporation into vascular structures within the infarct area (Figure 1 g, h, i). MSCs were detected in vascular structures as they expressed VEGF and vonWillebrand Factor, suggesting that they are capable of differentiating into vascular smooth muscle and/or endothelium. That the cells did not elicit rejection, despite the absence of immuno-suppressive drug therapy, was supported by the lack of a significant inflammatory response. (Note that cells surrounding vessel in Figure 1g and 1i are of MSC origin, as indicated by DAPI positivity in Figure 1h). The number of MSCs persisting in the myocardium decreased over time. Nonetheless, MSC injection produced a wide range of benefits, including improved regional and global ventricular function, reduced myocyte apoptosis, and improved tissue perfusion.

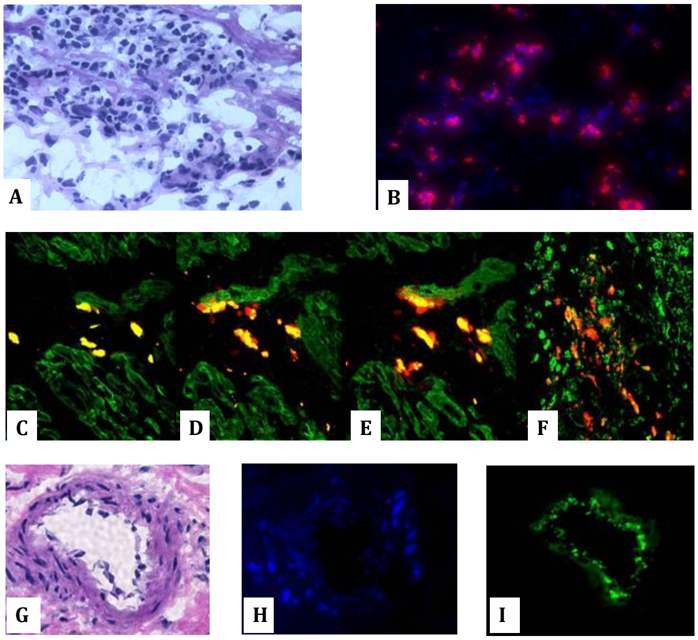

Figure 1. MSC engraftment and differentiation. MSC engraftment and muscle-specific protein-expression. DAPI and Di-I labeled MSCs (blue staining nuclei and red staining membranes, respectively) and fluorescent muscle protein-specific antibodies (green). (A) Hematoxylin and eosin (H&E)–stained section and corresponding fluorescent detection of cellular labels (B) depicts a cluster of MSCs in proximity to host myocardium. Several muscle-specific proteins are detected by immunoflouresence including α-actinin (C), phospho- lamban (D), tropomyosin (E) and troponin T (F). Yellow fluorescence indicates colocalization of immunofluorescent antibodies and DiI. (G) H&E stained sections of vascular structures at the border of the infarcted myocardium. Corresponding sections depict DAPI stained MSC nuclei (H) with immunofluoresecent detection of factor 8 (I).

In terms of functional responses, anterior MI caused dramatic deterioration of systolic and diastolic ventricular function, and impaired cardiac energy metabolism ($p<0.05$ vs. pre-MI values). Compared with injection of placebo, MSC cardiomyoplasty resulted in profound improvements in myocardial function and efficiency (Figure 2). Figure 2a depicts representative examples of pressure-dimension data from animals in either group. As shown, MSC treatment led to a pattern of LV recovery over a 2-3 month period marked by a substantial increase in stroke work (SW, the area within the loops). In the placebo-treated group, impaired cardiac function evident 3 days post infarction either persisted or worsened over 8 weeks of follow-up: indices of myocardial contraction fell and end-diastolic pressure rose (Figure 2 a,b,c,d). In marked contrast, LV end diastolic pressure increased to normal 8 weeks after MSC treatment (*$p<0.05$ vs. placebo). MSCs caused myocardial performance to recover to normal, both in systolic (Ees rose to 13.9±2.7 mmHg/mm and peak +dP/dt to 2465±575 mmHg/sec) and diastolic function (Tau fell to 37±3.8 msec).

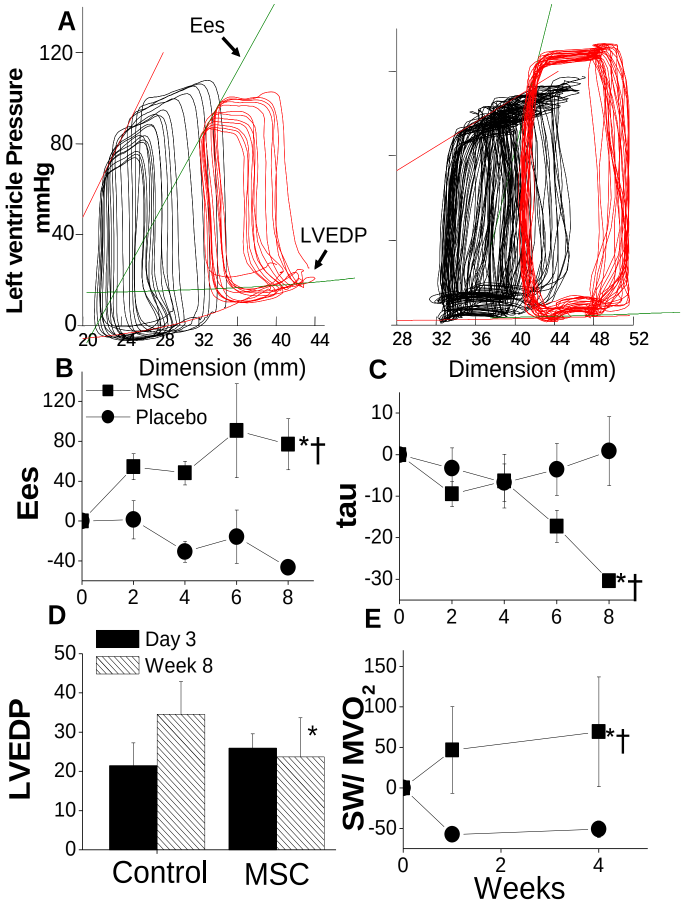

Figure 2. Physiologic impact of MSCs delivered with the BioCardia Catheter following anterior myocardial infarction (MI) in pigs. **(A)** Pressure-dimension (PD) data from placebo (left) and an MSC-treated (right) pig obtained 3 days (black loops) and 8 weeks (red loops) following MI. Placebo animals exhibit an increase in left-ventricular end-diastolic pressure (LVEDP) and dimension. Both myocardial contractility, measured by the slope of the end systolic pressure-dimension relationship (ventricular elastance, Ees), and ventricular stroke work, pressure-dimension loop area, decline in controls. In MSC-treated animals, Ees and stroke work increase to normal. **(B-E)** Average hemodynamic responses over 8 weeks showing divergent responses in cardiac function in MSC vs. placebo treated animals. **(B)** Ees declines in placebo-treated pigs but increases in the MSC group. **(C)** Isovolemic ventricular relaxation (τ), reduces to normal in MSC pigs but remains unchanged in placebo. **(D)** LVEDP increases in placebo but remains unchanged in MSC pigs. **(E)** Stroke work declines in placebo-treated animals while myocardial oxygen consumption (MVO2) increases (81±10.4%), leading to reduced SW/MVO_2. In contrast, in MSC-treated pigs, stroke work increases 89.8±15.3%, MVO_2 decreases 48.9±16.7%, resulting in augmented SW/MVO_2 and restoration of mechanoenergetic coupling toward normal. *$p<0.05$ *vs.* placebo and †$P<0.05$ vs. 3-day following MI, by ANOVA.

Heart failure and the aging cardiovascular system are characterized by mechanoenergetic uncoupling: decreased efficiency of work per unit oxygen consumption. In placebo-treated animals, SW decreased substantially during the 8-weeks following infarction, and there was a paradoxical increase in myocardial oxygen consumption, resulting in decreased ratio of SW/MVO_2. Conversely, MSC-injected animals' follow-up was marked by improving myocardial efficiency, both because of increasing SW (from 374.4±59.3 to 654.4±129.9 mmHg.mm at 8 weeks) and because of decreasing MVO_2 (from10.3±2 to 3.7±1.8 J/beat), both toward normal (Figure 2 e). Thus, MSC therapy exerts favorable effects on the damaged heart that extend to

improvements in cellular energy metabolism. The SW/MVO_2 ratio increased from 2.5±0.6 at 3 days post-MI to a normal ratio of 10±5.6 ($p<0.05$ *vs.* placebo) at 4 weeks. This improvement in mechanoenergetics was the earliest observable benefit of MSC treatment, preceding changes in global cardiac function. Improved mechanoenergetic coupling in the MSC group is consistent with several possible mechanisms, including reduced native tissue death[64], new tissue formation[42;65], or stimulation of endogenous repair mechanisms[66;67].

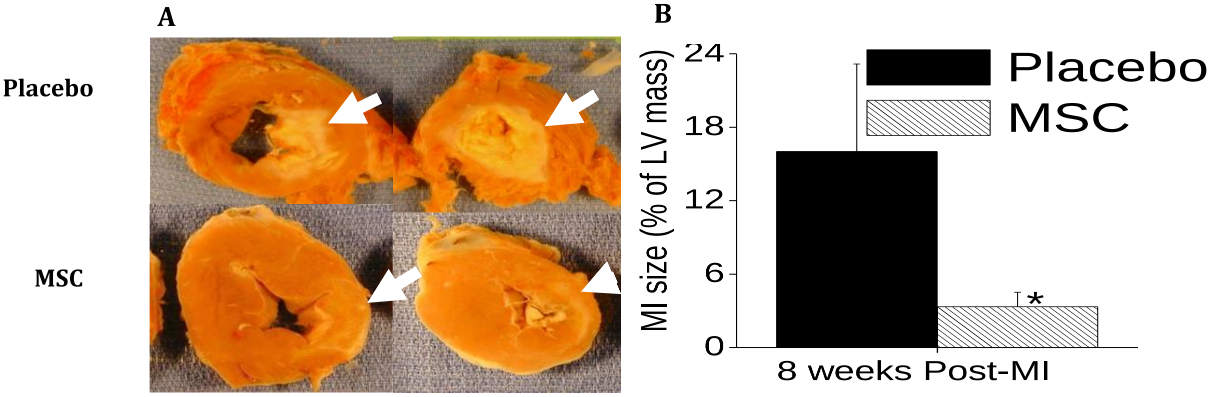

Figure 3. Myocardial infarct size 8 weeks following transient left anterior descending coronary artery occlusion. **A.** Representative example of scar formation due to myocardial infarction in placebo (top) and MSC treated animal (bottom). In placebo-treated animals the area of scar formation is transmural (arrow), while in the MSC group the scar area is barely visible and surrounded by non-scar tissue on both endo- and epicardial sides. **B.** Bar graph depicting scar formation as a percentage of LV mass. *P=0.008.

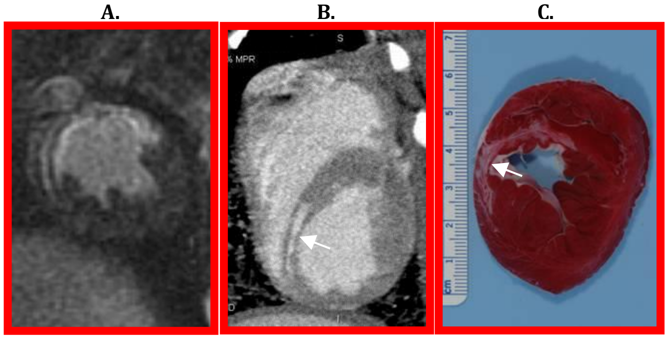

Figure 4. Comparison of infarct size using MRI (A), CT (B), and TTC (C). Images were obtained 8 weeks after closed-chest infarction in a pig and demonstrate subendocardial myocardial infarction as hyperenhancing region (~7-11 o'clock). TTC nonstaining areas (e.g., lack of brick red staining) in post-mortem slices (bottom) demonstrate concordance of infarct location and size with MRI and CT. Infarct region is notable for rim of noninfarctied myocardium along the endocardial border seen with CT and TTC staining. (arrows)

To further investigate the mechanisms of MSC-mediated cardiac repair, both MRI and computed tomography (CT) were used to image and quantify myocardial infarcts in MSC- and placebo-injected swine. Infarct size measurement *in vivo* by MRI and CT correlated tightly to that determined by triphenyltetrazolium chloride (TTC) staining post-mortem. Furthermore, using a 32 slice multidetector CT, the same endocardial rim of viable, non-infarcted myocardium observed in the first series of post-mortem hearts (Figure 3, Figure 4) was identified by *in vivo* imaging. These data not only speak to the therapeutic potential of MSC cardiomyoplasty, but also establish that noninvasive imaging techniques can be used to measure the effects of cardiomyoplasty, and to study the mechanisms underlying these effects. These results in this pig model provide strong rationale for the development of MSC-based cellular cardiomyoplasty strategies and support ongoing human studies.

MSCs injected intravenously home to and engraft in infarcted myocardium conferring functional benefit: Preliminary studies were conducted on the efficacy of

MSCs administered intravenously (*I.V.*) in a rat model of permanent left anterior descending (LAD) artery occlusion. Echocardiography was used to assess LV function at baseline, in the peri-infarct period, and four weeks after MI. MSC injection in Wistar rats led to dramatic improvement in LV function, with increased myocardial thickening and contractility in treated animals (Figure 5A and 5B). Labeled cells were identified within the infarct (Figure 6a), and were shaped like fibroblasts but expressed the cardiac protein, α-actinin, albeit at lower levels than native cardiomyocytes (Figure 6b). These labeled cells were most evident at the endocardial rim of the infarct, a finding similar to that seen in the porcine studies above.

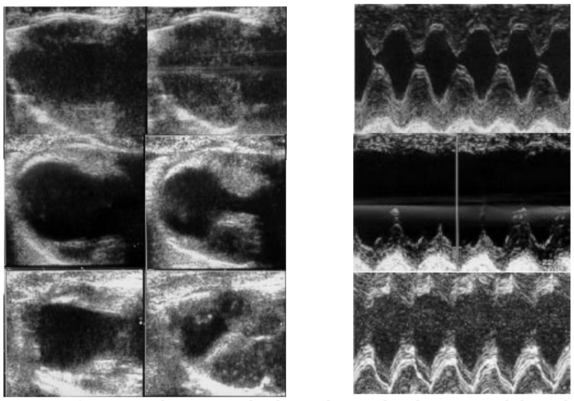

Figure 5. A: Two-dimensional echocardiography showing end diastole (left column) and end systole (right column) in a treated rat 1) before infarction 2) after infarction and prior to treatment and 3) 4 weeks after treatment with MSCs. **B:** M-Mode from same animal showing fractional shortening at the papillary level at the same time points as in A.

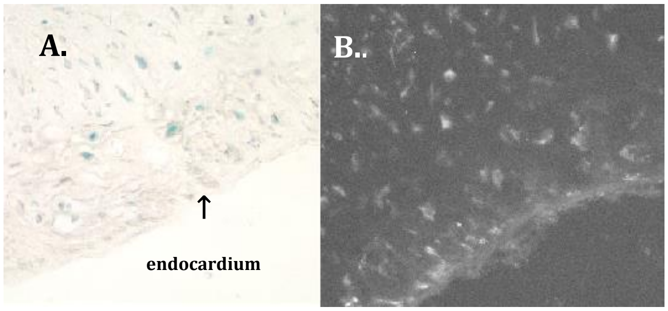

Figure 6: A: β-galactosidase positive (blue) cells are visible at 20X magnification within the infarct in young rats. These cells form a band along the endocardial surface. **B:** These cells show evidence of α-actinin expression on immunofluorescence, also at 20X magnification (bright appearing cells, B&W image). **C:** Quantification demonstrates 10-fold higher engraftment in young relative to old ($p<0.001$).

MSCs delivered intravenously (I.V.) distributed to the heart in response to an injury signal: MSCs injected *I.V.* at the time of coronary reperfusion homed to the myocardium, while cells injected *I.V.* two weeks after reperfusion were more likely to engraft in the bone marrow (Figure 7). Determination of SDF-1 and CXCR4 levels revealed not only that both are expressed by MSCs, but also that serum levels are up regulated immediately post infarct and remain elevated for at least 2 weeks (Figure 8).

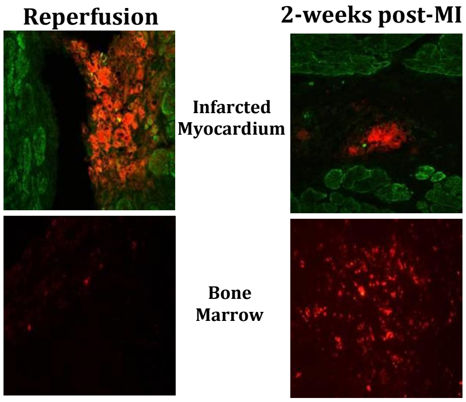

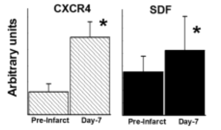

Figure 7. Confocal images of infarcted myocardium (top row) and bone marrow (bottom row) illustrating the degree of allogeneic MSC engraftment observed when 5 million MSCs were delivered via tail vein at reperfusion (left columns) or 2 weeks post-reperfusion (right column). As shown, at reperfusion cells home to the heart and not bone marrow; two weeks later, trafficking to the heart is reduced, and cells now migrate and engraft in the bone marrow. All tissues were harvested 4 weeks post-implantation. A

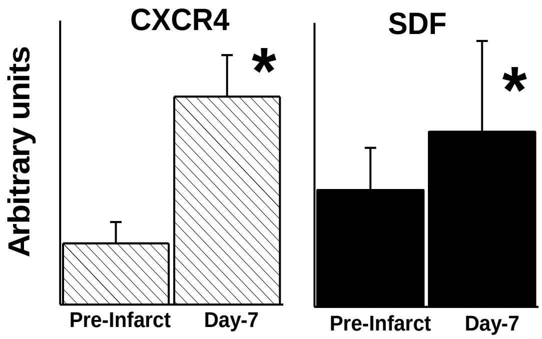

Figure 8: Both SDF-1 and CXCR4 levels are elevated following myocardial infarction. $P<0.05$ vs pre infarct

Myocardial Function can be Determined *in vivo* by MRI

The clinical research team has extensive experience using tagged MRI scanning to detect and quantify alterations in regional myocardial mechanics in animal models of ischemic heart disease[68;69]. The team has also developed non-surgical, MRI-compatible animal models for studying cardiac mechanics, perfusion, and interventional procedures[70-73]. The team has recently developed new MR imaging and analysis methods that enable the rapid determination of myocardial function in infarction and ischemia[70]. This new technique, Harmonic Phase (HARP) MRI[74;75] is based on tagged MRI techniques. Computationally, the analysis of HARP MRI can be performed much more rapidly than traditional tag tracking in tagged MRI. “Real-time” HARP imaging exploits the concept that only one of the spectral peaks must be acquired for motion in one direction; this in turn accelerates the image acquisition in addition to the rapid analysis already available in HARP. An example of the use of real-time HARP to monitor the onset of ischemia in a canine model of coronary artery stenosis is depicted (Figure 9)[104]. Using this technique, we can identify abnormal regional myocardial contraction after the onset of ischemia 20 seconds earlier than can be done using conventional cine wall motion studies and one minute earlier than ECG changes. HARP MRI, similar to tagged MRI, yields quantitative motion and strain parameters on a regional basis that can be used for comparison across subjects or at serial time points after intervention. Thus, HARP MRI and analysis represents a rapid and repeatable method to assess left ventricular function serially in a quantitative manner. HARP provides fast, accurate assessment of myocardial strains in humans with and without coronary artery disease[76]. Similar techniques have been used to assess structural and functional changes after myocardial infarction in rats[77;78].

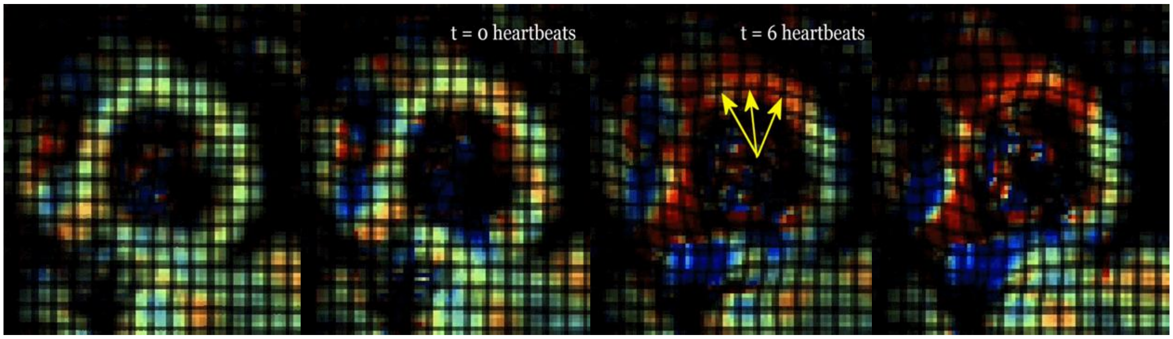

Figure 9: Real-time HARP images in the short-axis plane at different time points (10 sec prior to ischemia and 0, 6, and 20 heartbeats after the onset of ischemia from left to right) in a canine closed-chest model of acute LAD coronary artery occlusion. Overlaid on the tagged images is a pseudo-color map of circumferential shortening where green is uniform shortening, red is decreased shortening or stretching, and blue is increased shortening. At 6 heartbeats after ischemic insult, stretching of the ischemic myocardium is observed in the LAD bed whereas wall motion abnormalities by cine MRI could not be appreciated until 30 sec post-occlusion.

Noninvasive Determination of Infarct Size

Using an intravenous injection of Gd-DTPA, the study team is able to determine infarct size non-invasively with T1-weighted contrast-enhanced MRI (i.e., “Delayed Contrast-Enhanced MRI”). Short-axis image slices, which span the entire left ventricle, can be obtained using multiple breath-holds to yield images with the highest spatial resolution. The size of the area of hyper enhancement measured on such images has been shown

to be within 10% of the infarct size measured by post-mortem TTC staining (Figure 10). Alternately, using new imaging techniques, one can obtain the entire 3D left ventricular volume in a single breath hold (~16 heartbeats). It was recently shown that the 3D technique is in concordance with infarct size as measured by traditional 2D multi-slice techniques (Figure 10)[89,90]. Thus, infarct size and location can be accurately determined in less than one minute of scanning time, in order to determine the size of an infarct prior to therapy and over time.

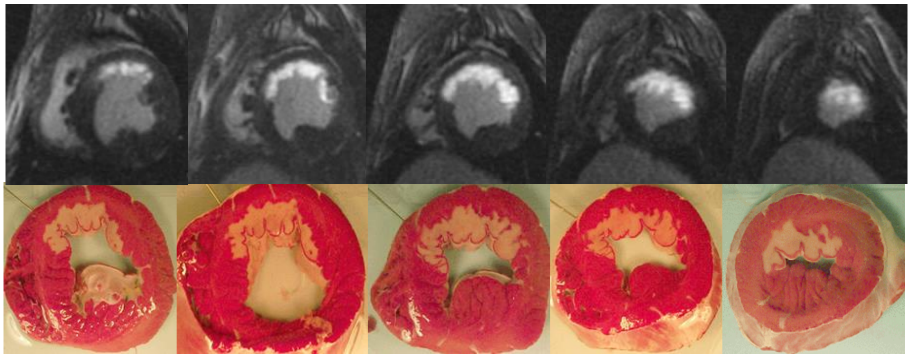

Figure 10. 2D delayed CE MRI in short axis plane (top) acquired within the first 24 hrs after closed-chest infarction demonstrating subendocardial myocardial infarction as hyperenhancing region (~11-1 o'clock) in a dog. TTC nonstaining areas (e.g., lack of brick red staining) in post-mortem slices (bottom) demonstrate concordance of infarct location and size with MRI.

Autologous Mesenchymal Stem Cells Produce Reverse Remodeling in Chronic Ischemic Cardiomyopathy: In addition to the studies outlined above using models of acute MI in the pig, we have also developed a model of chronic MI in the Gottingen mini-swine. We have used both autologous and allogeneic MSCs, with surgical and catheter delivery strategies, and have developed sufficient experience to translate the therapy from the laboratory bench to clinical trials. Together our results indicate that bone marrow derived MSCs stimulate cardiac recovery by engrafting, forming new blood vessels that increase tissue perfusion in hypoperfused areas, forming new cardiac myocytes, and importantly interacting with endogenous precursor cells to also contribute to new cardiac myocyte formation. From an immunologic perspective, MSCs may be safely used as an allogeneic graft, and have been done so extensively in clinical trials[79-81].

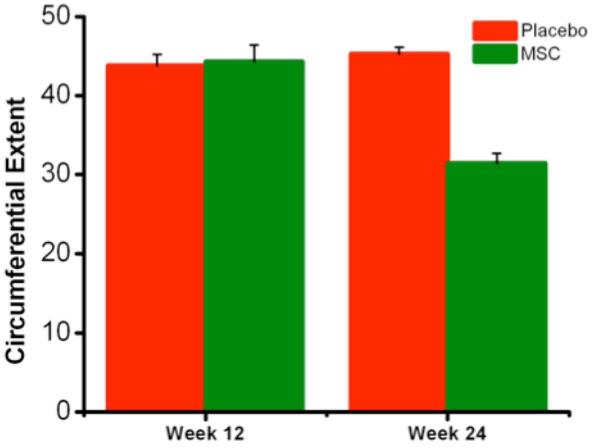

Figure 12. Infarct circumferential extent of the LV before and 12 weeks after injection in swine. Infarct size was significantly reduced by MSC treatment. $p<0.05$ MSC vs. placebo, $p<0.05$ MSC week 12 vs. week 24. [n=10]

Our experience with a 53 subject, 10-center, phase I study under the sponsorship of Osiris therapeutics, which demonstrated safety and provisional efficacy of allogeneic MSC therapy in subjects with acute infarction is outlined below[17]. In animal studies conducted in mini-swine, MSC injection via catheter into infarcted tissue reduces myocardial infarct size (Figs. 11 and 12), improves global and regional LV function, normalizes cardiac energetics, and restores tissue perfusion[63;82]. These results form the basis for our approval to conduct the CRATUS study.

Our work in large animal models with fully healed scars after MI showed that MSC administration can significantly improve left ventricular structural and functional indices, indicating meaningful repair. Using sophisticated imaging techniques, we tracked phenotypic improvements triggered by implantation of MSCs in a porcine model of chronic ischemic cardiomyopathy and quantified these changes morphometrically. MI was created in swine; after 12 weeks, the infarct segment had thinned, leaving a transmural scar (Figure 11). Autologous MSCs were expanded from each animal[83], and these cells or placebo were delivered to the infarct and surrounding border zone at this time. During a further 12-week follow up period, cardiac MRI revealed that intramyocardial injections of MSCs not only reduced the scar burden (as a percentage of LV mass) by $21.8 \pm 3.9\%$ ($p<0.05$ vs. placebo and week 12 vs. week 24) (Figure 11, 12), but also significantly improved regional contractility, global LV function, ejection fraction, and myocardial blood flow. Importantly, the therapy produced reverse remodeling and reduced the circumferential extent of the infarct scar (Figure 12). This constellation of effects suggests highly effective repair of ischemic cardiomyopathy. We subsequently confirmed reverse remodeling in a pilot study of 8 subjects with ischemic cardiomyopathy (Figure 21)[43].

<u>Allogeneic Mesenchymal Stem Cells Restore Cardiac Function in Chronic Ischemic Cardiomyopathy Via Trilineage Differentiating Capacity</u>:

We tested the hypothesis that MSC based cardiac repair regenerates the heart via mechanisms comprising long-term engraftment and by differentiation into both myocardial and vascular elements. We generated allogeneic MSCs from a male swine donor, and administered sex mismatched cells by transendocardial injection into female swine 12 weeks post-MI. Animals were followed with serial MRI, and 12 weeks later the hearts were collected for immunohistological evaluation. The fate of the male donor cells was determined by co-localization of Y-chromosome (Y^{pos}) cells with markers of cardiac, vascular, and endothelial lineages. MSCs engrafted in infarct and border zones and differentiated into cardiomyocytes (Figure 14) as ascertained by co-localization with GATA-4, Nkx2.5, and α-sarcomeric actin markers. In addition, Y^{pos}MSCs exhibited vascular smooth muscle and endothelial cell differentiation, contributing to large and small vessel formation. The number of cells engrafting correlated with the functional changes that occurred (Figure 14F). Thus, MSCs could engraft and repair hearts in chronic ischemic cardiomyopathy[84].

Ventricular remodeling is a progressive disease causing the myocardium to reorganize itself from an elliptical to a spherical shape. During its reorganization the heart will become

enlarged and ventricular dimensions increase, assisting in reshaping a subject's heart. In swine models, MSCs show that the reduction in scar size supports reverse remodeling, which show a reduction in scar size as quickly as 3 days after injection. MSCs have shown as a promising cell-based therapy where previously there was limited means of improving a subject's heart function and survival[85].

Figure 14. Merging CMR angiography and electroanatomical mapping to guide transendocardial mesenchymal stem cell injection.

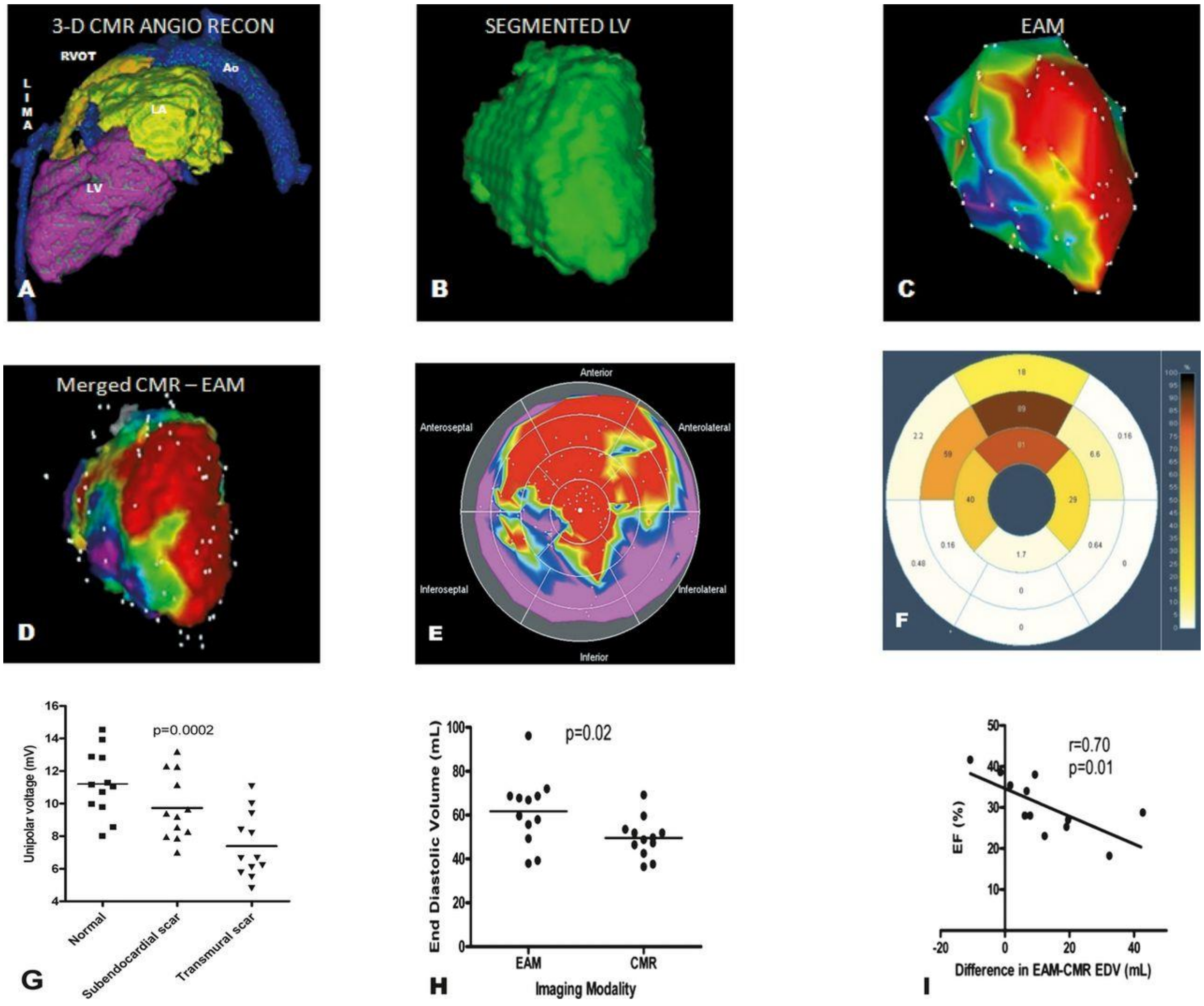

Figure 15: Durable and progressive scar size reduction due to intramyocardial injection of allogeneic bone marrow MSCs. A, Delayed-enhancement CMR scar size images show durable reduction in scar size with MSC therapy.

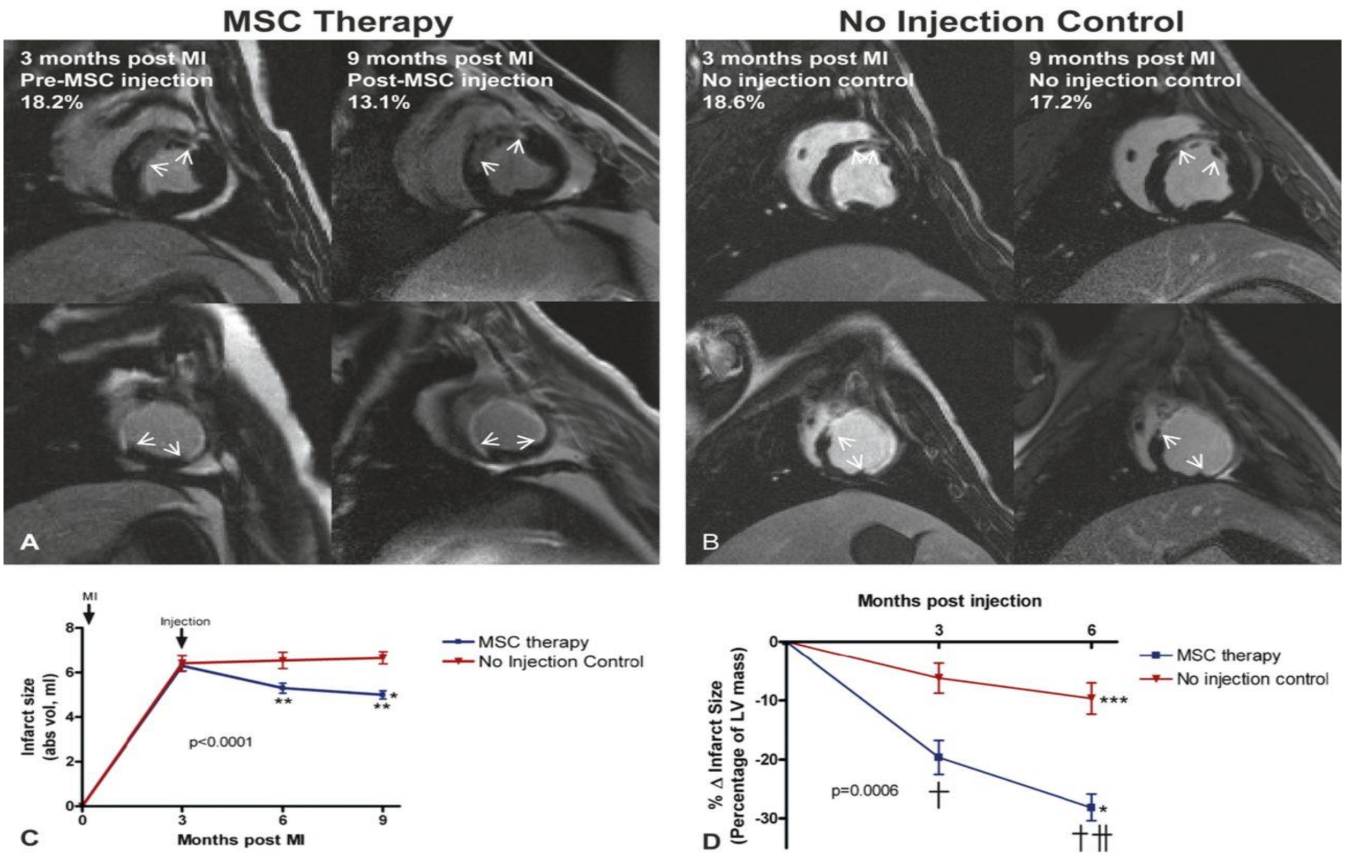

Figure 16: Allogeneic MSC therapy reverses remodeling in ischemic cardiomyopathy.

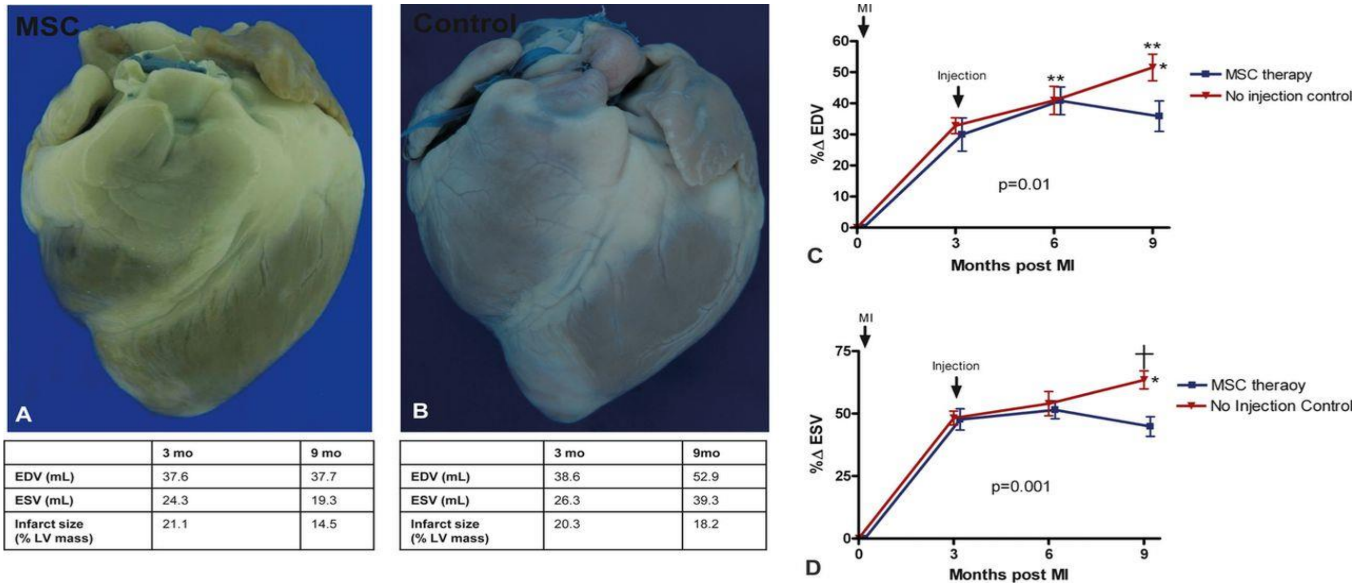

	3 mo	9 mo
EDV (mL)	37.6	37.7
ESV (mL)	24.3	19.3
Infarct size (% LV mass)	21.1	14.5

	3 mo	9mo
EDV (mL)	38.6	52.9
ESV (mL)	26.3	39.3
Infarct size (% LV mass)	20.3	18.2

Figure 17: Allogeneic MSC therapy improves LV sphericity index.

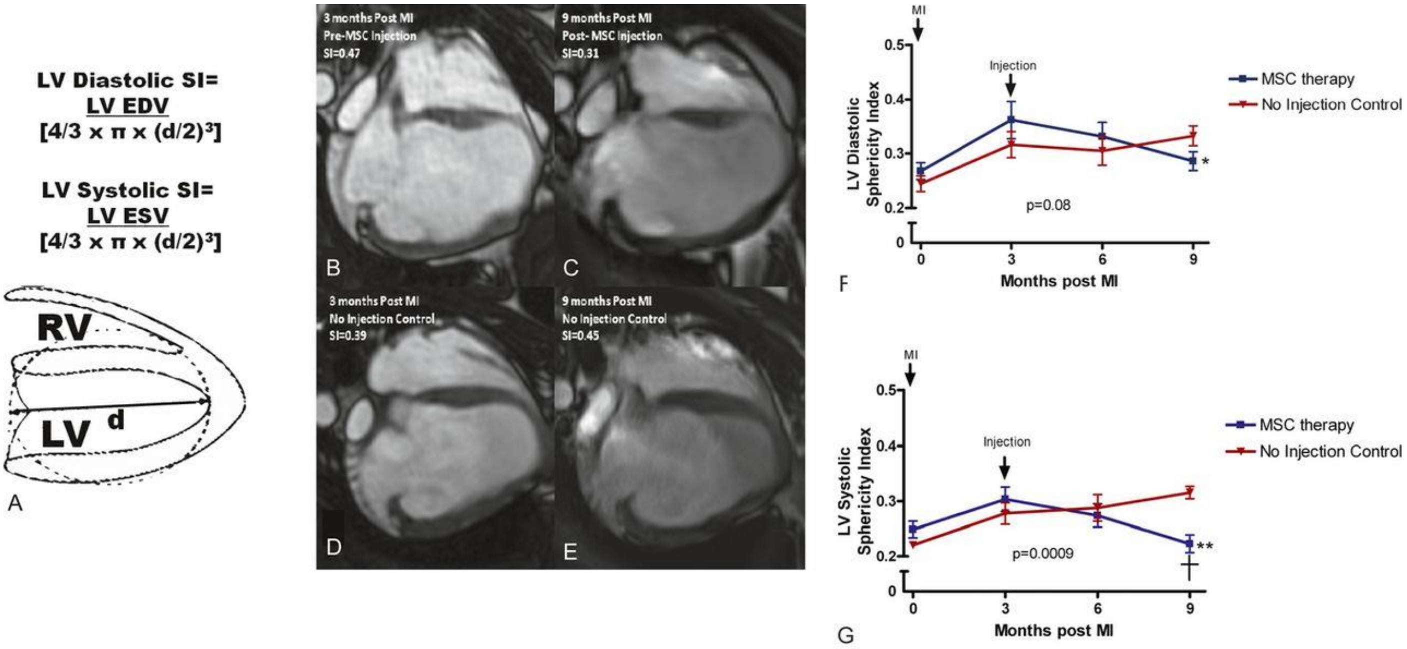

Figure 18. Progressive improvement in LV function with allogeneic MSC therapy.

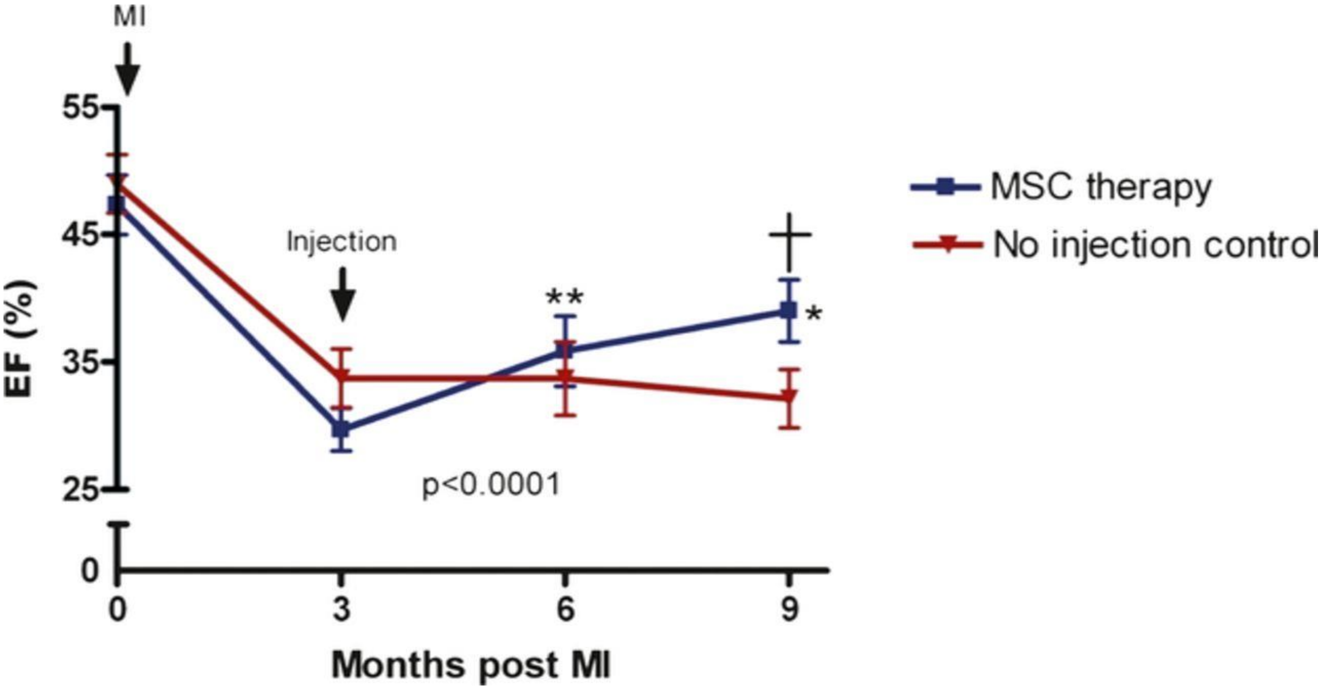

1.4 Allogeneic Mesenchymal Stem Cells: Previous Experience in Humans

The use of allogeneic cellular products typically requires matching of the graft HLA to the donor in order to avoid graft rejection and graft versus host disease. However, because MSCs do not express HLA, they represent a unique immunoprivileged cell population which can be used for allogeneic cellular therapy. In addition, MSCs fail to induce proliferation of allogeneic lymphocytes *in vitro* and suppress proliferation of T cells activated by allogeneic cells or mitogens. MSCs have also been shown to exert anti-proliferative, immunomodulatory and anti-inflammatory effects. Many subjects have received allogeneic MSCs and infusions have all been well tolerated.

A multi-center, randomized, double-blind, placebo-controlled study was performed to evaluate the safety and preliminary efficacy of allogeneic MSCs administered after myocardial infarction[17]. In this study, 53 subjects were treated with one of three cell-dose levels of allogeneic MSCs (0.5, 1.6 and 5.0 cells/kg body weight) or placebo administered intravenously. No HLA matching was performed in this study and administration was found to be safe and well tolerated at all dose levels (with 5.3 adverse events per subject in the MSC-treated group vs. 7.0 in the placebo group). No deaths were reported and no serious adverse events were attributed to MSC administration. Improvements were seen in subjects receiving MSCs as compared with those receiving placebo in the frequency of arrhythmic events and premature ventricular contractions, post-event ejection fraction for subjects with major anterior wall infarctions, overall clinical status, and notably post-infusion pulmonary function as measured by FEV1 percent predicted (increased 17% in the MSC-treated group vs. 6% for placebo group, $p < 0.05$).

Allogeneic MSC infusion has also been studied in a phase II trial of MSCs for the treatment of severe acute graft versus host disease. In this study, 55 subjects received 1-5 intravenous infusions of 1.4×10^6 cells/kg body weight from HLA matched and mis-matched donors. A complete response was seen in 30 subjects and improvement was seen in 9 subjects. Of note, response rates were not associated with HLA-matching. No infusion related side effects were noted and no long-term adverse events were observed.

The safety and efficacy of allogeneic MSCs for the treatment of refractory lupus has also been explored[86]. Fifteen subjects received a single intravenous infusion of 1×10^6 cells/kg body weight. MSCs were derived from family members but were not HLA-matched. At 12 months, all subjects had improvement in disease activity as measured by 24 hour proteinuria (decreased from 2505.0±1323.9 to 858.0±800.7 mg/24hr, $p<0.05$) and SLE Disease Activity Index scores (decreased from 12.2±3.3 to 3.2±2.8, $p<0.05$). No serious adverse events were noted in any of the subjects.

The Poseidon trial (IND #13568; NCT01087996), has provided additional validity that MSCs reduce infarct size and reverse remodeling of the myocardium[85]. Study data also reveal that MSCs are immunoprivileged and immunosuppressive, and do not cause acute immunogenic reactions. In this regard, no subject reported symptoms or indications of a reaction. Only one of 15 subjects receiving ahMSCs mounted a donor specific alloreaction, of low antibody titer[18]. Furthermore, subjects treated with allogeneic MSCs suffered fewer AE's versus autologous MSCs subjects. It is also hypothesized that the function of autologous MSCs could be impaired in subjects with co-mobility or advanced

age[87;88]. Reverse cardiac remodeling was a prominent factor subjects showed as assessed by the study CT scan results[18]. Allogeneic MSCs were shown in this trial to be a safer and more effective form of cell-therapy than autologous MSCs. Figures 19 and 20 depict functional and quality of life improvements in subjects received both allogeneic and autologous MSCs.

We randomized 30 subjects in an open-label study of either allogeneic or autologous hMSCs at 3 doses; 20, 100, or 200 million total hMSCs. The study was designed as a pilot study to compare the safety and efficacy of allogeneic versus autologous hMSCs in subjects with chronic ischemic left ventricular dysfunction secondary to myocardial infarction. Autologous MSCs were derived from a sample of the subject's bone marrow approximately 4-6 weeks prior to cardiac catheterization. Allogeneic MSCs were supplied from a human MSC source manufactured by the University of Miami. All 30 subjects tolerated the procedure well and have been followed-up with serial cardiac CT at 13 months post injection. The serious adverse event rate (SAE) was lower among subjects treated with allogeneic MSCs as compared to autologous MSCs (6-months: 20%-allogeneic MSCs and 40%-autologous MSCs). The injection of MSCs into the hearts of these subjects produced reverse remodeling, with reductions in EDV of ~23 ml and reduction in spherecity index of 0.8.

Together, these preliminary data support our hypothesis that allogeneic MSC administration is a safe and successful option for cellular cardiomyoplasty. We have demonstrated long-term MSC survival, engraftment, and differentiation into myocardial, vascular, and endothelial lineages following transplantation into chronically scarred porcine myocardium. These cells' capacity for cardiomyogenesis and vasculogenesis both likely contribute to their ability to repair chronically scarred myocardium.

Figure 19: Functional Outcomes

A) Six-Minute Walk Test (meters)

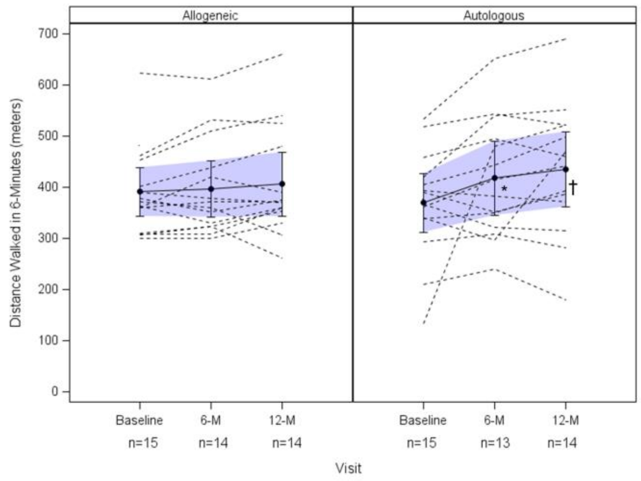

B) Peak VO_2 (ml/kg/min)

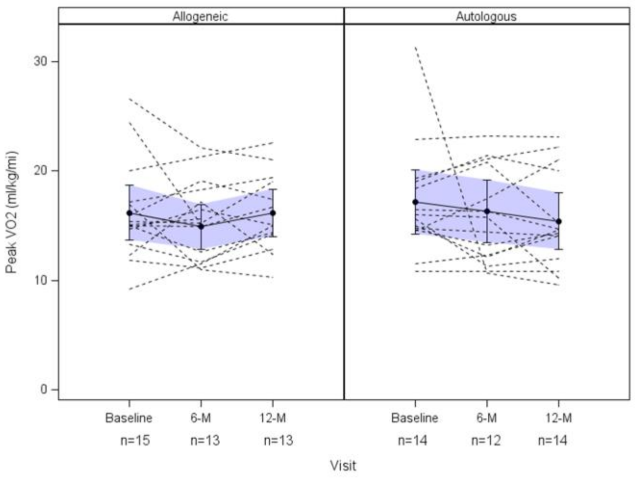

C) Minnesota Living with Heart Failure

D) New York Heart Association Class

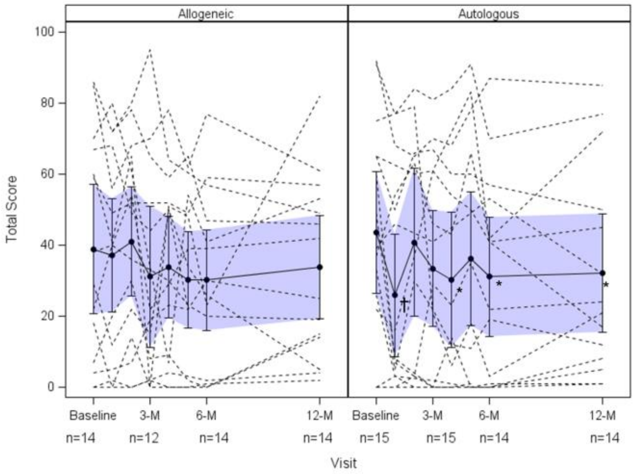

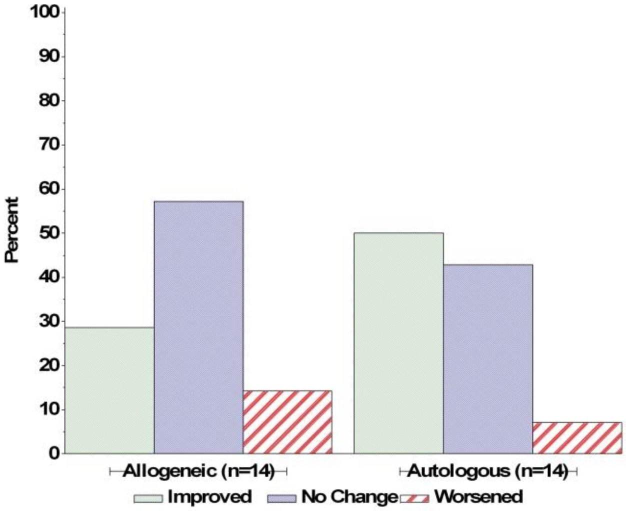

Figure 20: CT Parameters Change from Baseline

A) Left Ventricular Ejection Fraction (%)

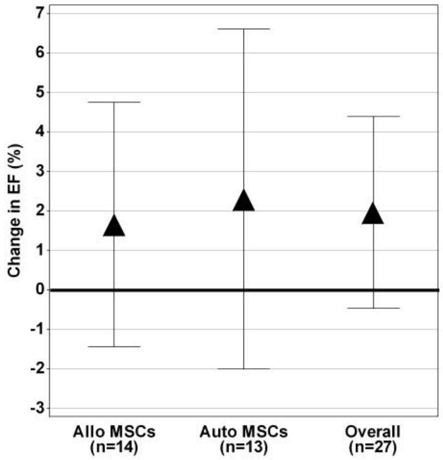

B) End Diastolic Volume (ml)

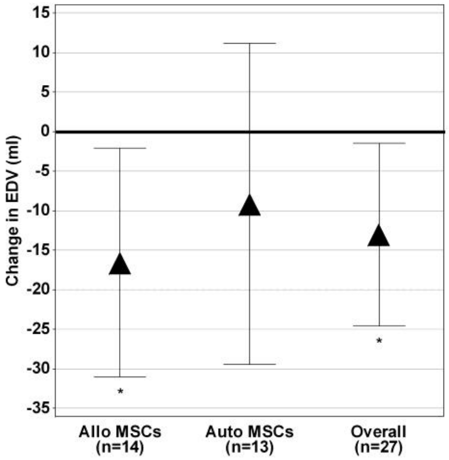

D) End Diastolic Myocardial Volume (cm^3)

C) End Systolic Volume (ml)

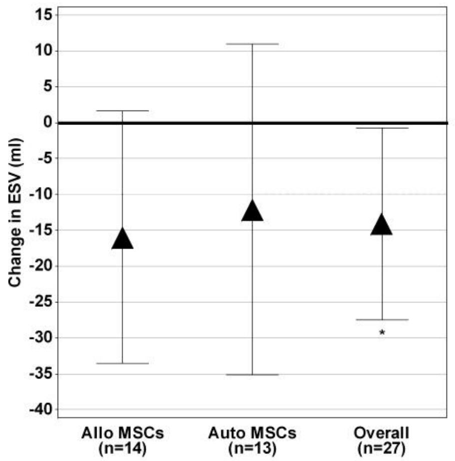

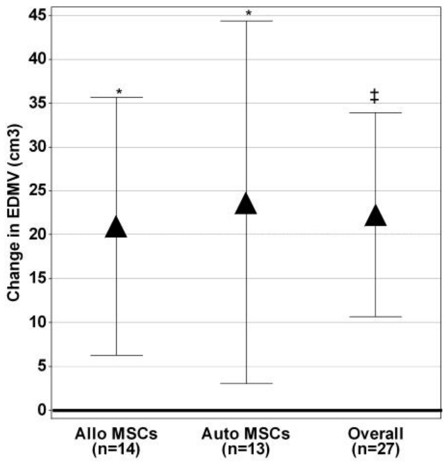

E) Sphericity Index

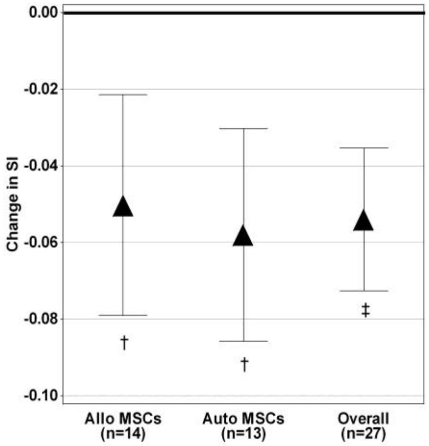

F) MI Size (Early enhancement defect; grams)

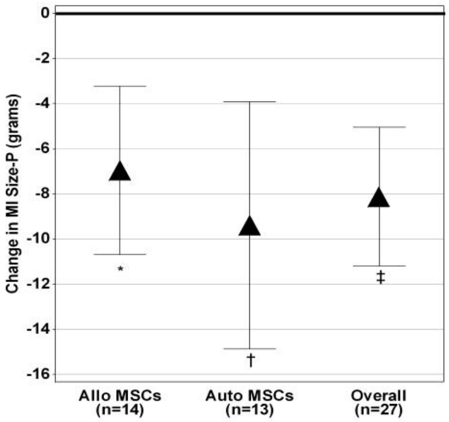

G)

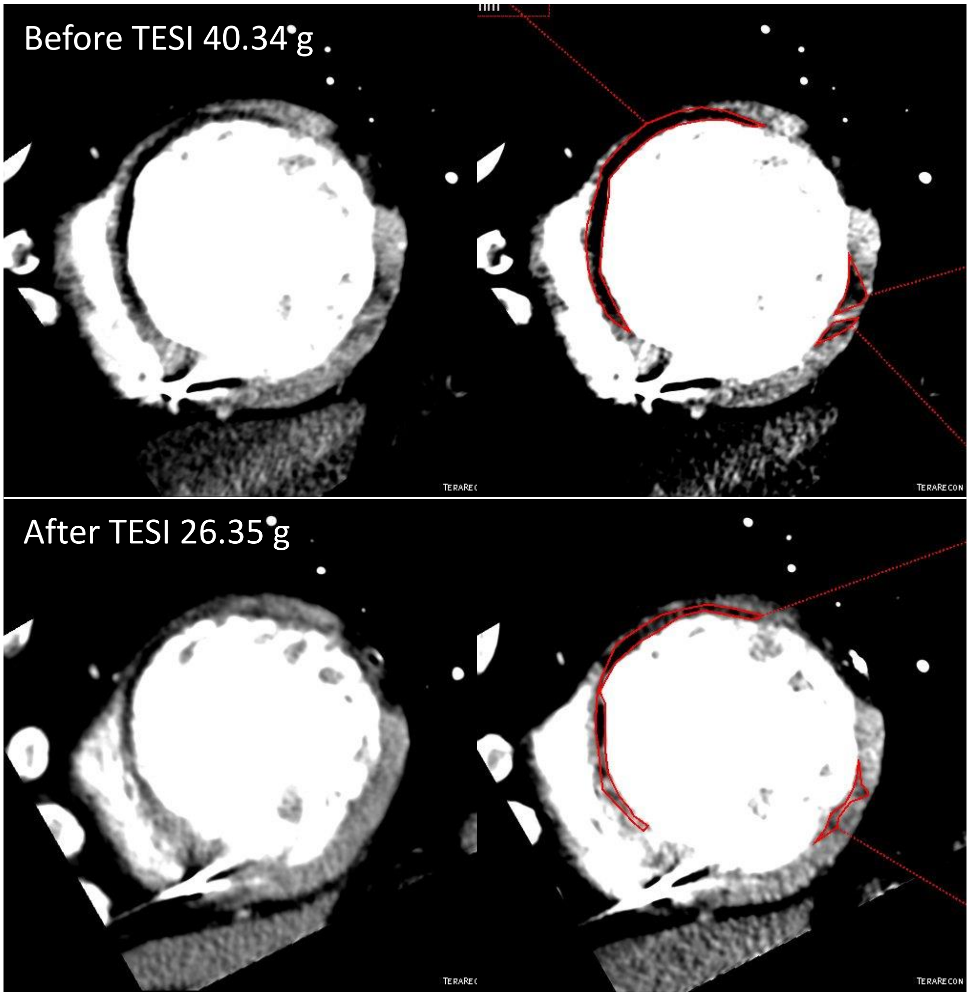

The study by Weiss et al, which investigated ahMSC therapy for COPD, reflected similar trial results indicating as the Poseidon trial that allogeneic MSCs did not cause acute immuogeneic reactions in subjects participating in the trial. Importantly, ahMSCs produced a decrease in the inflammatory marker CRP. Systemic administration of MSCs appears to be safe and decrease inflammation in an older and co-morbid population of subjects with compromised lung function due to moderate COPD[19].

Preliminary Results from the Transendocardial Autologous Cells in Ischemic Heart Failure Trial (TAC-HFT): We enrolled 8 subjects in an open-label run-in phase of the TAC-HFT trial to assess the safety and preliminary efficacy of bone marrow mononuclear cells (MNCs) and MSCs in subjects with ischemic cardiomyopathy[24, 25]. At baseline each subject underwent a bone marrow aspiration and transendocardial injection (Helix Infusion Catheter; Biocardia, Inc., CA) of bone marrow derived MNCs or MSCs to the infarct and border zone using biplane fluoroscopic guidance. All 8 subjects tolerated the procedure well and have been followed-up with serial cardiac MRI at 3, 6, and 12 months post injection (Figure 21). The injection of bone marrow derived cells into the hearts of these subjects produced reverse remodeling, with reductions in EDV of ~12% and improved regional function as measured by –Ecc, a cardiac MRI derived index (Figure 21). As described above, we have also treated subjects with ahMSCs in the POSEIDON trial and have demonstrated an outstanding safety profile.

A. **B.**

C.

D. **E.**

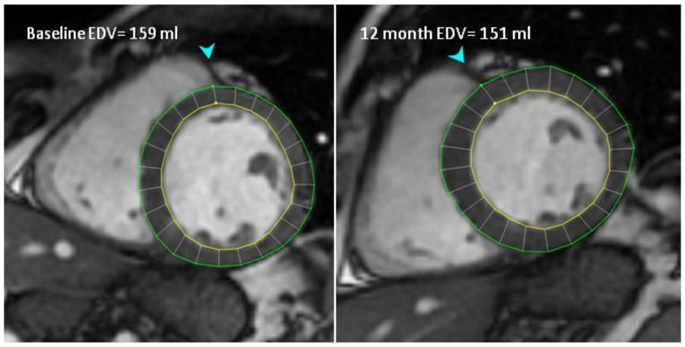

F. **G.**

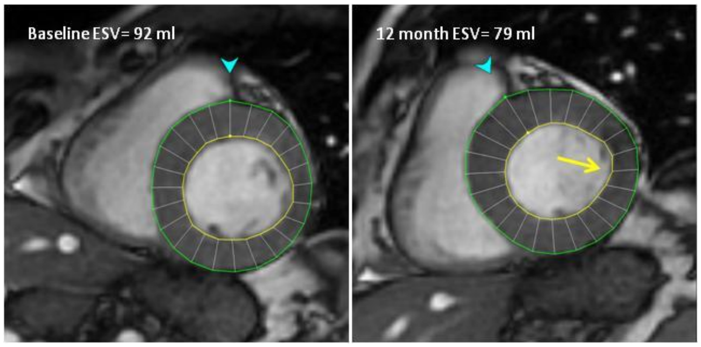

Figure 13. Cardiac MRI follow-up data of (A) end-diastolic volume, (B) end-systolic volume, and (C) peak Eulerian circumferential strain (Ecc) of the infarct zone from tagged imaging. Example cardiac MRI images of change in EDV from (D) baseline to (E) 1 year, and change in ESV from (F) baseline to (G) 1 year after stem cell injection. As depicted, EDV and ESV are reduced by 6 months following injection, and do not return towards baseline by 12 months. Peak Ecc is

dramatically reduced by 3 months following injection (the more negative Ecc corresponds to *improving* regional LV function). *p<0.05 in post-hoc analysis compared to baseline. Yellow Arrow: Note improved systolic thickening in lateral wall, the site of cell therapy in this example.

1.5. Pharmacology and Toxicology Studies of Mesenchymal Stem Cells

Preclinical data suggest that MSCs may safely be used in the treatment of Frailty. Using the murine bleomycin model, several groups have shown that the administration of stem cells ameliorates bleomycin induced lung injury with no significant adverse effects.

Lee et al intravenously administered $1x10^6$ bone marrow derived MSCs to rats treated with bleomycin and found a decrease in bleomycin induced lung edema, neutrophil infiltration, collagen deposition, and overall mortality with no adverse effects reported[55]. Similarly, Ortiz et al intravenously administered 5×10^5 bone marrow derived MSCs to mice with no adverse effects reported. They found that after bleomycin exposure, MSCs home to sites of lung injury, lead to decreased fibrosis and extracellular matrix collagen deposition, and contribute to tissue repair[51]. Rojas et al intravenously administered 5×10^5 allogeneic bone marrow derived MSCs to mice treated with bleomycin and observed that lung injury attracts bone marrow derived MSCs via the production of soluble factors like G-CSF and GM-CSF which lead to MSC proliferation and migration and ultimately improved survival with no reported adverse effects[56].

These findings appear to extend beyond bone marrow derived MSCs. Cargnoni et al administered fetal membrane derived cells (both allogeneic murine and xenogeneic human) to bleomycin treated mice and found that placental derived stem cells, like MSCs, localize to the lung and reduce tissue damage associated with bleomycin exposure regardless of source or route of administration (intravenous, intraperitoneal, or intratracheal)[50]. They did find that intraperitoneal and intratracheal administration of cells led to mild to moderate lung inflammation but no fibrosis in the absence of bleomycin injury. Importantly, this was not seen with intravenous administration of cells. In another murine model, xenogeneic human umbilical cord derived MSCs were also shown to home to sites of bleomycin induced lung injury, inhibit the production of pro-inflammatory cytokines, and reduce lung injury and collagen deposition with no adverse effects reported[52].

Table 1. Results of preclinical animal studies of mesenchymal stem cell therapy for IPF.

Study	Model	Cell Type	Cell Delivery and Dose	Safety Results	Efficacy Results
**Lee at al, 2006**	**Rat**	**Allogeneic**<br><br>**BM-MSCs**	**Intravenous, 1 x 10^6 cells**	**No adverse effects reported.**	**Reduced edema, neutrophil infiltratration, collagen deposition.**<br><br>**Improved survival.**

**Ortiz et al, 2003**	**Mouse**	**Allogeneic**<br><br>**BM-MSCs**	**Intravenous, 5 x 10^5 cells**	**No adverse effects reported.**	**Reduced inflammation and collagen deposition.**
**Rojas et al, 2005**	**Mouse**	**Allogeneic**<br><br>**BM-MSCs**	**Intravenous, 5 x 10^5 cells**	**No adverse effects reported.**	**Reduced pro-inflammatory cytokines.**<br><br>**Improved survival.**
**Cargnoni et al, 2009**	**Mouse**	**Fetal membrane derived**<br><br>**(Allogeneic murine and xenogeneic human)**	**Intra-peritoneal,**<br><br>**4 x 10^6 cells**<br><br>**Intratracheal, 1 x 10^6 cells**<br><br>**Intravenous, 1 x 10^6 cells**	**Mild to moderate lung inflammation but no fibrosis induced by intraperitoneal or intratracheal administration.**<br><br>**No adverse effects reported with I.V. administration.**	**Decreased neutrophil infiltration and fibrosis regardless of route of administration or source of cells.**
**Moodley et al, 2009**	**Mouse**	**Xenogeneic human umbilical cord derived MSCs**	**Intravenous, 1 x 10^6 cells**	**No adverse effects reported.**	**Reduced inflammation, pro-inflammatory cytokine production, and collagen deposition.**

In addition to safety data from preclinical animal studies, many subjects have received allogeneic MSCs in clinical trials and infusions have all been well tolerated.

A multi-center, randomized, double-blind, placebo-controlled study was performed to evaluate the safety and preliminary efficacy of allogeneic MSCs administered after myocardial infarction[17]. In this study, 53 subjects were treated with one of three cell-dose levels of allogeneic MSCs (0.5, 1.6 and 5.0 cells/kg body weight) or placebo administered intravenously. No HLA matching was performed in this study and administration was found to be safe and well tolerated at all dose levels (with 5.3 adverse events per subject in the MSC-treated group vs. 7.0 in the placebo group). No deaths were reported and no serious adverse events were attributed to MSC administration. Improvements were seen in subjects receiving MSCs as compared with those receiving placebo in the frequency of arrhythmic events and premature ventricular contractions, post-event ejection fraction for subjects with major anterior wall infarctions, overall clinical status, and notably post-infusion pulmonary function as measured by FEV1 percent predicted (increased 17% in the MSC-treated group vs. 6% for placebo group, $p < 0.05$).

Allogeneic MSC infusion has also been studied in a phase II trial of MSCs for the treatment of severe acute graft versus host disease[31]. In this study, 55 subjects received 1-5 intravenous infusions of 1.4 x 10^6 cells/kg body weight from HLA matched and mismatched donors. A complete response was seen in 30 subjects and improvement was seen in 9 subjects. Of note, response rates were not associated with HLA-matching. No infusion related side effects were noted and no long term adverse events were observed.

The safety and efficacy of allogeneic MSCs for the treatment of refractory lupus has also been explored[86]. Fifteen subjects received a single intravenous infusion of 1 x 10^6 cells/kg body weight. MSCs were derived from family members but were not HLA-matched. At 12 months, all subjects had improvement in disease activity as measured by 24 hour proteinuria (decreased from 2505.0±1323.9 to 858.0±800.7 mg/24hr, $p<0.05$) and SLE Disease Activity Index scores (decreased from 12.2±3.3 to 3.2±2.8, $p<0.05$). No serious adverse events were noted in any of the subjects.

2. STUDY OBJECTIVES AND ENDPOINTS

2.1 Study Objectives

2.1.1 Primary Objective

- To demonstrate the safety of intravenous allogeneic hMSCs administered in subjects with Frailty and to explore treatment efficacy (decrease in frailty, frequency of acute exacerbations, change in symptom related quality of life, improved cardiovascular status, decrease in inflammatory biomarkers, endothelial function and 1 year survival).

2.1.2 Secondary Objectives

- To explore effects of allo-hMSCs on symptom related quality of life, cardiovascular performance, endothelial function and inflammation.

2.2 Study Endpoints

2.2.1 Primary Endpoints (Safety)

- Safety (Primary): Incidence (at one month post infusion) of any treatment-emergent serious adverse events (TE-SAEs), defined as the composite of: death, non-fatal pulmonary embolism, stroke, hospitalization for worsening dyspnea and clinically significant laboratory test abnormalities, determined per the Investigator’s judgment.

  - Serum chemistry: chloride, sodium, Carbon Dioxide, BUN, creatinine, glucose, calcium, AST/SGOT, ALT/SGPT, total bilirubin (fractionate if total >1.5 times normal), alkaline phosphatase, albumin,
  - Hematology (CBC): hemoglobin, hematocrit, platelets, WBC, WBC differential

2.2.2. Secondary Endpoints (Efficacy)

The following efficacy endpoints will be evaluated in this trial (during baseline, 3 and 6 months follow-up visits):

1. Difference in rate of decline of Frailty defined as:
    a. Reduced Activity (assessed via CHAMPS questionnaire)
    b. Slowing of Mobility (assessed via a 4 meter gait speed test and SPPB assessment)
    c. Weight Loss
    d. Diminished handgrip strength (assessed via dynamometer)
    e. Exhaustion (assessed via the MFI questionnaire)
    f. Decrease in subject quality of life assessment(s)
2. Death from any cause.
3. Change between baseline and 6 months in dobutamine stress echo induced ejection fraction.
4. Change between baseline and 6 months for the following panel of inflammatory markers: CRP, IL-6, D-dimer, fibrinogen,

CBC with differential, DNA, and TNFα

3. STUDY DESIGN

3.1 Description of the Study

A Pilot Phase will be performed to test the safety of dose and volume escalation of cells administered via peripheral intravenous infusion. The randomized portion of the study will be conducted after a full review of the safety data from the Pilot Phase by the DSMB.

Following the pilot study thirty (30) subjects will be scheduled to undergo peripheral intravenous infusion and meeting all inclusion/exclusion criteria will be evaluated at baseline.

In the pilot phase, the first three (3) subjects in each treatment group will not be treated less than 5 days apart and will each undergo full evaluation for 5 days to demonstrate there is no evidence of treatment emergent SAE’s, defined as the composite of: death, non-fatal pulmonary embolism, stroke, hospitalization for worsening dyspnea and clinically significant laboratory test abnormalities, prior to proceeding with the treatment of further subjects.

In the pilot phase, the fifteen (15) subjects will be able to receive additional administrations of the study product. See Addendum A for rationale and description.

3.2. RANDOMIZATION STUDY

This Phase I/II, randomized, blinded, placebo-controlled study is designed to evaluate the safety and tolerability of allo-hMSCs in subjects with Frailty and to explore potential efficacy at baseline, 3 and 6 months.

Approximately fifteen (15) subjects will be enrolled in the pilot phase and thirty (30) subjects with Frailty will be enrolled in the randomized phase for a total of up to forty-five (45) subjects. In the randomized phase, subjects will then be enrolled and randomized 1:1:1 to an active arm or placebo. Additional subjects may be enrolled if deemed appropriate.

Eligible subjects must have a diagnosis or symptoms of frailty as defined by the Canadian Study on Health & Aging[2]. Following informed consent before or at the screening visit, the diagnosis of FRAILTY will be confirmed by investigator review of medical history.

In the randomized phase of the trial, electronic randomization will be performed using the Advantage EDC system and communicated to cellular laboratory personnel who have no contact with the investigators or subjects. At the time of administration, opaque tubing will be used to maintain double blinding. Treatments will be administered once and will consist of 1×10^8 allo-hMSCs (100 million cells), 2×10^8 allo-hMSCs (200 million cells), or placebo. After each infusion, subjects will be monitored for immediate complications.

Continued safety and tolerability with review of adverse events (AEs) will be monitored at each visit. Efficacy parameters (pulmonary function tests, 6MWT, and QOL questionnaires) will be assessed following Day 0 until study completion. Clinical laboratory tests to assess safety will be performed at every clinic visit, excluding the baseline visit.

In the randomized phase of the trial, subjects who received placebo will have the option to receive 1 x 108 (100 million) cells of allogeneic hMSCs, if all study endpoints are met. If all endpoints are met then the subject will be administered the study drug and follow the study schedule from Day 1 to Month 12 after receiving the infusion of allogeneic hMSCs.

4. SUBJECT SELECTION

4.1 Inclusion Criteria

In order to participate in this study, a subject <u>MUST</u>:

1. Provide written informed consent.
2. Subjects age $\geq$60 and $\leq$95 years at the time of signing the Informed Consent Form.
3. Show signs of frailty apart from a concomitant condition as assessed

by the Investigator with a frailty score of 4 to 7 using the Canadian Clinical Frailty Scale

4. Female subjects must have an FSH $\geq$ 25.8 mIU/mL, if not currently on hormone replacement therapy.

4.2 Exclusion Criteria

In order to participate in this study, a subject MUST NOT:

1. Score of $\leq$24 on the Mini Mental State Examination (MMSE)
2. Inability to perform any of the assessments required for endpoint analysis (report safety or tolerability concerns, perform PFTs, undergo blood draws, read and respond to questionnaires.
3. Active listing (or expected future listing) for transplant of any organ.
4. Clinically important abnormal screening laboratory values, including but not limited to: hemoglobin <8 g/dl, white blood cell count <3000/mm^3, platelets<80,000/mm^3, INR > 1.5 not due to a reversible cause (i.e. Coumadin), aspartate transaminase, alanine transaminase, or alkaline phosphatase > 3 times upper limit of normal, total bilirubin > 1.5 mg/dl.5.
5. Serious comorbid illness that, in the opinion of the investigator, may compromise the safety or compliance of the subject or preclude successful completion of the study. Including, but not limited to: HIV, advanced liver or renal failure, class III/IV congestive heart failure, myocardial infarction, unstable angina, or cardiac revascularization within the last six months, or severe obstructive ventilatory defect.
6. Any other condition that, in the opinion of the investigator, may compromise the safety or compliance of the subject or preclude successful completion of the study.
7. Be an organ transplant recipient.
8. Have a clinical history of malignancy within 3 years (i.e., subjects with prior malignancy must be disease free for 3 years), except curatively-treated basal cell carcinoma, squamous cell carcinoma, melanoma in situ or cervical carcinoma if recurrence occurs.
9. Have a non-pulmonary condition that limits lifespan to < 1 year.
10. Have a history of drug or alcohol abuse within the past 24 months.
11. Be serum positive for HIV, hepatitis BsAg or Viremic hepatitis C.
12. Be currently participating (or participated within the previous 30 days) in an investigational therapeutic or device trial.
13. Be a female who is pregnant, nursing, or of childbearing potential while not practicing effective contraceptive methods. Female subjects must

undergo a blood or urine pregnancy test at screening and within 36 hours prior to infusion.

14. Hypersensitivity to dimethyl sulfoxide (DMSO).

4.3 Concomitant Treatments, Procedures, and Nondrug Therapies

All concomitant medications (prescription or over-the counter) as well as procedures or nondrug therapies (e.g. continuous positive airway pressure, pulmonary rehabilitation) will be recorded at the initial screening visit and updated at each subsequent visit. Except for other experimental treatments or medications with putative disease modifying effects in FRAILTY, subjects will continue all prior concomitant medications for comorbid diseases to ensure optimal general medical care.

4.4 Withdrawal Criteria

Subjects will be informed that they have the right to withdraw from the study at any time and for any reason without prejudice to future or continued medical care. Subjects must be withdrawn for the following reasons:

1. Subject request.
2. Subject is unable or unwilling to comply with the protocol.
3. Medical reasons, at the discretion of the investigator.

Reason for withdrawal will be recorded in the subject's case report form. In order to adequately monitor for safety and potential efficacy outcomes, subjects who are withdrawn for any reason after receiving the first infusion should be encouraged to return for all assessments through the end of the study period. All efforts should be made to continue to record safety data and lung function parameters for all withdrawn subjects. Subjects who withdraw for reasons unrelated to the study or study drug (e.g. withdrawal of consent or loss to follow-up) may be replaced if deemed necessary to meet study objectives. Replacement subjects will be assigned unique identification numbers.

5. MESENCHYMAL STEM CELL DONORS

The availability of allogeneic hMSCs (allo-hMSC) offers the potential for an "off the shelf" product for subjects. Significant data has been generated to demonstrate that the allogeneic hMSCs are immunoprivileged and can be infused without immune rejection despite disparate HLA phenotypes.

Screening of allogeneic donors will follow standard transplant practices and all allogeneic donors will meet allogeneic donor eligibility criteria as outlined in 21 CFR Part 1271.

Allogeneic donor testing will include anti-HIV-1/2, anti-HTLV I/II, anti-HCV, HIV-1 nucleic acid testing, HCV nucleic acid testing, HBsAg, anti-HBc(IgG and IgM), CMV, West Nile Virus nucleic acid, *T. cruzi* ELISA (Chagas), Zika Virus and RPR. Potential donors testing positive for any of these infectious diseases with the exception of CMV, will be ineligible.

Bone marrow (BM) aspirates will be obtained from a maximum of 15 normal individuals and allo-hMSCs will be isolated and expanded.

5.1 Bone Marrow Aspiration for Generation of MSCs

A total of approximately 60mL to 120mL of BM will be obtained from each normal volunteer. BM will be aspirated from the posterior iliac crest into heparinized syringes. The mononuclear cell fraction will be isolated using a density gradient with Lymphocyte Separation Media (specific gravity 1.077). The low-density cells will be collected and washed with Plasma-LyteA containing 1% HSA. The washed cells will be sampled and viable cell numbers determined. The MNCs will be prepared antibiotic free (ie. No penicillin or streptomycin). The BM mononuclear cells will be seeded into 175 cm^2 tissue culture flasks in alpha MEM containing 20% FBS. After 14 days of culture, passage zero (P0) cells will be harvested by trypsin treatment and expanded into 60 flasks. These flasks are incubated for a further 7 to 10 days and then the MSCs are harvested by trypsin treatment (P1 cells).

5.2 Normal Donor Eligibility

Donors (male or female) between the ages of 18 to 45 (inclusive) will be screened as potential BM donors. Donors will be evaluated by history and physical examination. The history will include:

- History of malignancy
- Bleeding abnormalities
- Prior deep venous thrombosis
- Known cardiac or pulmonary conditions
- Prior blood transfusions
- Vaccinations
- Questions to identify persons at risks of infectious disease transmission
- Questions to identify persons at risk of transmitting hematological or immunological disease
- A physician will administer the National Marrow Donor Program (NMDP) Questionnaire (a donor health history screening questionnaire).

The physical examination will include evaluation for potential risks associated with the BM aspiration procedure. Prospective donors will have infectious disease testing including:

- Hepatitis B surface antigen (HBsAg)
- Anti-Hepatitis B core antibody (HBcAb)
- Anti-Hepatitis C virus antibody (HCV Ab)
- Anti-Human Immunodeficiency Virus (HIV) antibody (HIV 1/2)
- Cytomegalovirus antibody (CMV)
- HCV/HIV Nucleic Acid test
- West Nile Virus Nucleic Acid test
- Rapid Plasma Reagin (RPR)

- Human T-lymphotropic Virus I/II (HTLV I/II)
- *T. cruzi* ELISA test (Chagas disease)
- *Zika Virus (RNA qualitative Real Time RT-PCR)*

Prospective donors will also have the following blood and urine tests:

- Complete blood count with differential
- Complete metabolic panel, magnesium, calcium, and uric acid
- Urinalysis

Eligibility Criteria for Normal Donors will include:

- Male and female gender
- No history of malignancy
- No active coagulopathy and/or hypocoagulable state
- No history of cardio/pulmonary conditions
- Negative tests for Hepatitis B, Hepatitis C, RPR, Chagas, HIV ½, HTLV I/II and NAT for HCV, HIV, Zika Virus, and WNV.
- Hemoglobin ≥ 13.0 g/dL if male; and if female donor hemoglobin ≥ 11.0 g/dL
- Platelet count 140,000 to 440,000/ul
- WBC 3.0 to 11.0 K/ul
- No anomalies on the CBC and differential suggestive of a hematopoietic disorder
- Creatinine ≤1.5 mg/dL
- ALT≤ 112 IU/L
- Bilirubin < 1.5 mg/dL
- No diabetes
- Systolic blood pressure ≤ 170
- Diastolic blood pressure ≤ 90
- No history of autoimmune disorders
- Negative serum or urine pregnancy test for female donors

Female donors would need to be screened for pregnancy as the procedure may be an added risk to a fetus.

5.3 Donor Consent

Informed consent will be obtained from all potential donors. The procedure will be explained in terms the donor can understand, and will include information about the significant risks of the procedure. Potential donors will have an opportunity to ask questions, the right to refuse or withdraw consent, and access to the results of all tests.

5.4 Follow-up Schedule for Donors

After discharge from the hospital, the bone marrow donor will be contacted by the study team with a follow-up telephone call to determine the well-being and health status of the

donor. The donor will be provided with contact telephone numbers in the consent form for any questions or comments.

5.5 Biomarker Assessment

All samples will be identified so that they can be linked to individual subjects. These samples may be stored indefinitely. Individual results will not be returned to the subject or the study physician. The samples will be linked to subjects, but there will be no recontact. Data presented in publications will not contain individual subjects' clinical characteristics or outcomes; only aggregate data from the entire study will be disclosed.

6. TREATMENT OF SUBJECTS

6.1 Study Investigational Product

The investigational product (IP) consists of hMSCs obtained from donor bone marrow. Screening of allogeneic donors will follow standard transplant practices and all allogeneic donors will meet allogeneic donor eligibility criteria as outlined in 21 CFR Part 1271. BM will be obtained from normal volunteers with approximately 60 ml to 120 ml aspirated from the posterior iliac crest. The BM will be aspirated into heparinized syringes. The MNC fraction will be isolated using a density gradient with Lymphocyte Separation Media (specific gravity 1.077). The low-density cells will be collected and washed with Plasma-LyteA containing 1% HSA. The MNCs will be prepared antibiotic free (ie. No penicillin or streptomycin). The washed cells will be samples and viable cell numbers determined. The BM MNC will be seeded into 175 cm^2 tissue culture flasks in alpha MEM containing 20% FBS. After 14 days of culture, passage zero (P0) cells will be harvested by trypsin treatment and expanded into 60 flasks (P1 cells). After 7 to 10 days P1 cells are harvested by trypsin treatment (P1 cells). Cells from P1 will be cultured for 7 to 10 days and harvested by trypsin treatment and expanded into 180 flasks (P2 cells). After 7-10 days P2 cells are harvested by trypsin treatment and cryopreserved. This process does not contain penicillin and/or streptomycin. Any subject that test positive for a penicillin or streptomycin allergy will be provided with an antibiotic free MSC product. The existing product prepared with antibiotic and stored under this BB-IND #15679 will be used only for subjects who are non-allergic to penicillin or streptomycin until all MSCs are depleted.

6.2 Dosing

During the pilot phase of the study 15 subjects will receive a single infusion of hMSCs

Group 1 will consist of Five (5) subjects and will be treated with a single administration of allogeneic hMSCs: 2×10^7 (20 million) cells delivered via peripheral intravenous infusion.

Group 2 will consist of Five (5) subjects and will be treated with a single administration of allogeneic hMSCs: 1×10^8 (100 million) cells delivered via peripheral intravenous infusion.

Group 3 will consist of Five (5) subjects and will be treated with a single administration of allogeneic hMSCs: 2 x 10^8 (200 million) cells delivered via peripheral intravenous infusion.

After subjects complete their Month 12 follow-up phone call visit in the pilot phase, all 15 subjects will then have the option of receiving a second single infusion of allogeneic hMSCs: 1 x 10^8 (100 million) cells delivered via peripheral intravenous infusion. The option will be provided if subjects in the pilot phase continue to meet all inclusion / exclusion criteria's. See Addendum A for follow-on phase for initial 15 subjects.

In the randomized phase of allo-hMSCs or matched placebo 30 subjects will be randomized in a 1:1:1 ratio to one of two Treatment Strategies or placebo following successful completion of the Pilot Phase.

Group A will consist of 10 subjects who will receive 100 million Allogeneic hMSCs delivered via peripheral intravenous infusion.

Group B will consist of 10 subjects who will receive 200 million Allogeneic hMSCs delivered via peripheral intravenous infusion.

Group C will consist of 10 subjects who will receive placebo via peripheral intravenous infusion.

The Allo-hMSCs will be derived from donors meeting criteria for allogeneic unrelated human bone marrow stem cell source manufactured by the University of Miami.

6.3 Dosage Rationale

A safety profile for IV infusion of hMSCs was based on results from previous completed toxicology results[17]. The results from previous studies demonstrate that the product can be administered intravenously without toxic events at up to 65 x 10^6hMSC/kg dose delivered in one bolus infusion or at 100 x 10^6hMSC/kg cumulative dose delivered by 5 infusions (20 x 10^6hMSC/kg per infusion).

The evidence supports the conclusion that it is feasible to dose subjects in this study based on a standard dose of hMSCs rather than per kilogram of body weight. The total cell number corresponds to a range of 1.3 - 4.4 x 10^6 hMSCs per kg per infusion for subjects with 45 to 150kg body weight, the weight range for this study.

Therefore, results from previous trials support the rationale on the safety and potential efficacy of the selected maximum dose of 200 x10^6 allo hMSCs.

6.4 Administration Rate

Prior clinical trials have used rates up to 30×10^6 hMSC/min where no infusion related toxicity was observed.

In the proposed study, the cell dose to be delivered is 20x 10^6, and 100×10^6hMSC/infusion, and 200x10^6 reconstituted with the 2.5 million hMSC/ml, in the following total volume

- 25 ml for 20 million dose (5 million hMSC/min)
- 40ml for 100 million dose (5 million hMSC/min)
- 80ml for 200 million dose (5 million hMSC/min)

Cell will be delivered at a rate of 2ml/min, and delivered at a maximum rate of 16×10^6 hMSC/minute and will last approximately:

- 12.5 minutes for 25ml for 20 million dose
- 40 minutes for 40ml for 100 million dose
- 40 minutes for 80ml for 200 million dose.

The infusion bag will be flushed with an additional 25 ml of 0.9% normal saline at the completion of allo-hMSC infusion and delivered at a rate of 2ml/min.

- ***For the randomized double blinded phase we will prepare the following doses of 100 million, 200 million and Placebo in an 80ml bag.***
- ***During the randomized phase each dose will take place over approximately 40 minutes in order to maintain the blind.***

6.4.1. Infusion Monitoring

Subjects will be monitored in the ICU for two hours prior to infusion to establish baseline vital signs (oxygen saturation, heart rate, blood pressure, and temperature) every 15 minutes. Monitoring will also continue throughout the infusion.

Once the infusion is begun, 2L/min oxygen via nasal cannula will be provided if the oxygen saturation drops below 90% on room air. The infusion will be stopped if the oxygen saturation does not return to >93% within 3 minutes of initiating supplemental oxygen or if the subject requires greater than 2L/min supplemental oxygen to achieve the required saturation of >93%. If a subject requires the addition of oxygen, it will be continued for 4 hours after the completion of the infusion. At that time, oxygen will be weaned off to maintain a saturation >93% on room air.

6.5 Concomitant Therapy

6.5.1 Permitted therapy

Concomitant medications will be recorded on the case report form (CRF), which includes all FDA-approved medications, therapies, and dietary supplements.

6.5.2 Excluded therapy

Medications and therapies not approved by the FDA are prohibited for the duration of this trial, including participation with any investigational drug or device.

6.5.3. Subject monitoring

All aspects of the study will be conducted in accordance with Good Clinical Practice (GCP) as described in the ICH Guideline (CFR ICH Selected Regulations and Guidance for Drug Studies, CFR Title 21 Food and Drugs Revised as of April 1, 2002) all applicable national and local regulations. Monitoring will be conducted by a qualified outside source at the study site.

Monitoring of key safety endpoints will be conducted. If rates significantly exceed the pre-set threshold, then the DSMB will be advised.

6.6 Blinding and Unblinding

Subjects will be randomized into active groups. Only designated technicians in the ISCI Cell Processing Laboratory will be unblinded to treatment. The investigator, study staff, subject and anyone involved in the care of the subject will not be made aware of the assigned treatment regimen. Before dispensing the investigational product, Cell Therapy Lab staff will confirm the CMV status of eligible recipient. This information will be used to select Allo- MSC product. CMV status of the recipient and donor of the Allo-MSC product will be matched. CMV positive Allo-MSC product will only be infused to a CMV positive recipient. All CMV negative recipients will receive CMV negative Allo-MSC product[91].

The designated cell-processing technicians will prepare both the allogeneic hMSCs infusions. The investigational agent infusions will be prepared in identical infusion bags and labeled with the identical investigational drug labels. A brown plastic slip cover will be placed over the infusion lines as well as the bags to maintain the blind. The designated technicians in the ISCI Cell Processing Laboratory (or designee) will be responsible for maintaining the investigational product records including randomized treatment assignments by subject identification.

If for important medical reasons unblinding is thought to be necessary, the Investigator may identify the treatment assignment by obtaining the randomization assignment by contacting the Director of Experimental and Clinical Cell Based Therapies at ISCI who is responsible for maintaining randomization records for all subjects.

6.7 Study Investigational Therapy Management

6.7.1 Investigational Product Labeling and Storage

The product label contains the elements required by the CFR and other national and local authorities for investigational products. ISCI GMP. Cell Processing Facility (CPF) will directly store and deliver the designated cell processing technologist in the CPL/CPF, and will be kept cryopreserved in liquid nitrogen vapor phase until shortly before

administration must be stored in a securely locked enclosure. Access is strictly limited to unblinded CPL/CPF personnel prior to preparation for infusion. After preparation for infusion, the Investigator and his or her designees are permitted to administer the Investigational Product only to subjects participating in this protocol.

6.7.2 Investigational Product Accountability Procedures

In accordance with all applicable regulatory requirements, the Cell Processing Laboratory/Cell Processing Facility will maintain a record of the investigational products hMSCs received, dispensed, administered, destroyed, or returned. The final disposition of all unused, empty, and partially used Cryocyte™ bags will be handled in accordance with the drug preparation manual. An independent unblinded clinical research associate (monitor) or auditor will perform compliance monitoring during the study.

Only unblinded personnel may access accountability records until the study blind has been broken.

7. STUDY PROCEDURES

7.1. Time and Events Schedule

The Time and Events Schedule for the conduct of this study is shown in Table 2

Schedule of Assessments

Table 2: Time and Events Table

VISIT	Screening Day -56 ± 28 days	Baseline (-4 weeks)	Day 1	Week 2 (Day 14) +/- 2 days	Month 1 (Day 30) (+/-2 weeks)	Month 3 (Day 90) (±/-2 weeks)	Month 6 (Day 180) (+/-2 weeks)	Month 12 (Day 365) (±/-2 weeks) *Phone Call Follow-up
Informed Consent	x							
Full Medical History	x							
Physical Exam	x	x	x	x	x	x	x	
12-lead (ECG)	x	x	x	x	x	x	x	
Concomitant Medications	x	x	x	x	x	x	x	x
Mini Mental State Examination (MMSE)	x						x	
Randomization		x						
Infusion Treatment (IP)			x					
Dobutamine Stress Echo Test (DSE)	x						x	
Bone Density Scan (DEXA)[8]		x					x	
FEV-1		x				x	x	
6 Minute Walk Test		x				x	x	
4 Meter Gait Speed Test[7]		x				x	x	
SPPB Assessment		x				x	x	
Dynamometer (handgrip)		x				x	x	
Smell Identification Test (UPSIT)		x				x	x	
IIEF, SQOL-F Questionnaires		x			x	x	x	
QOL Questionnaires (ICECAP, EQ-5D, SF-36,CHAMPS, MFI)		x			x	x	x	
Questionnaires (Subject and Physician Global Assessment[9])							x	
Urinalysis	x				x	x	x	
Hemat., Chem., CBC, LFTs, INR, and other labs[1]	x		x	x	x	x	x	
HIV 1, HIV 2, Hep. B & C, and CMV	x							
Serum or Urine Pregnancy Test[2]	x		x					
Donor Screening Tests	x							
Review Adverse Events			x	x	x	x	x	x
Immune Monitoring[4]			x	x	x	x	x	
Biomarker Assessment[3]			x				x	
Optional: Brachial Ultrasound [5]		x				x		
Optional: Endothelial blood samples[6]		x				x		

Time and Events Table Key:

1 - The minimal laboratory requirements for hematological, liver function and renal function include:

Hematology Tests: white blood cell count, platelet count, hemoglobin and hematocrit.

Liver Function Tests: Albumin, alkaline phosphatase, alanine transaminase, asparate aminotransferase, prothrombin time / activated partial thromboplastin time, and bilirubin(fractionate if total >1.5 times normal).

Renal Function Tests: creatinine, creatinine clearance, blood urea nitrogen (BUN), glomerular filtration rate, sodium, potassium, chloride, calcium, carbon dioxide, and glucose.

Serum Uric Acid, Pro-BNP, and C-reactive protein (CRP), IL6, fibrinogen, D-Dimer, TNFα, testosterone (males only) and estrogen (females only).

2 - A serum or urine pregnancy test will be completed within 36 hours prior to infusion for females of childbearing potential.

3 - The following biomarkers will be analyzed:

- **Cell-surface markers**: CXCR4, C-Kit, & Connexin 43
- **Transcriptomic/Proteome:** RNA, miRNA, protein samples, and telomerase, akt
- **Growth factors**: Sdf-1, notch,
- **Functional Assays:** cell growth rate, VEGF, and CFU assay

4 - Immune monitoring for graft rejection. The following markers will be used for analysis to assess for activated T-cells based upon a $CD3^{+}CD25^{+}$ or $CD3^{+}CD69^{+}$ phenotype:

- CD3, CD25, CD69

5 - Optional brachial ultrasound to assess endothelial function.

6 - Optional: An additional 5 lavender top tubes (EDTA) will be drawn.

7 – 4 meter gait speed test will be performed twice per visit and the average of the exams will be taken.

8 – DEXA scan will be performed twice at each visit. The first scan will be of the hip and spine for bone density and the second will be to assess the total body composition.

9 – Subject and Physician Global Assessments are not applicable to pilot subjects in this phase of the trial.

7.2 Study Phases and Visits

7.2.1 Screening Visit

See Table 2 for the procedures and assessment to be performed during the screening visit of the study. All screening visit test and procedures will occur upon signing the informed consent form (ICF). No screening exams will take place until the subject is fully informed of the research and signs the consent form.

Physical exam

A complete physical examination will include general appearance, skin, head and neck, Lymph nodes, musculoskeletal/extremities, cardiovascular, chest/lungs, abdomen, and neurological assessment. At screening information about the physical examination and any significant findings must be recorded in the source documentation at the study site. Weight should be measured at each physical exam on a calibrated scale with the subject wearing only light clothing and no shoes. Height (in bare feet or wearing only thin socks) will be measured at the Screening visit.

Vital signs

Vital sign measurements will be performed at least once on each study visit up to time of discharge. These measurements will consist of respiratory rate, heart rate, blood pressure, and temperature. Respiratory rate, heart rate, and blood pressure should be measured in a sitting position after 5 minutes of rest.

Dobutamine Stress Echocardiography (DSE)

A Dobutamine Stress Echocardiography will be performed twice during the study, once at screening and at the Month 6 follow-up visit. This exam will assist in mimicking the effect of exercise on subjects to assess the heart muscle when under stress to better evaluate ejection fraction in frail subjects.

7.2.2 Baseline Visit

See Table 2 for the procedures and assessment to be performed during the baseline visit of the study. Once all screening exams are completed and it has been determined that the subject remains eligible for the study, subjects will be enrolled into the study. The baseline visit will take place within four weeks from treatment. The listed procedures should all be performed as soon as practicable.

Bone density Dexa scan will be performed at the baseline visit (if not done within three months prior to enrollment).

The Smell Identification Test (UPSIT) test will be performed once at baseline and once at the Month 3 and Month 6 follow-up visits. Age-related olfactory dysfunction is felt to be due to cumulative inflammatory damage affecting the olfactory mucosa. The Smell Identification Test (UPSIT) is a self-administered 40-item olfactory test. It provides an absolute indication of smell loss (anosmia, mild, moderate, or severe microsomia) as well as an index to detect malingering.

Endothelial function (Optional Assessments) will occur upon the subject signing the optional section of the informed consent form (ICF). No endothelial function tests will take place until the subject is fully informed of the research and signs the optional portion of the consent form.

- Brachial ultrasound testing and blood collection will be performed to assess endothelial function in the aging frailty population at baseline and 3 months post stem cell infusion. This will help provide cumulative data in assessing whether or not stem cell infusion improves endothelial function.

- Flow Mediated Diameter percent change (FMD%): All measurements of the brachial artery diameter and FMD will be performed in the morning, in a quiet and dark room and at controlled ambient temperatures between 20°C and 26°C. Studies will be conducted after an overnight fast of at least 10 hours (water is permitted), with the subjects supine and after 10 minutes of rest. The subject's right arm will be comfortably immobilized in an extending position, allowing for ultrasound scanning of the brachial artery 5–10 cm above the antecubital fossa. In each examination, recording of vessel images will be followed by inflation of a cuff to supra-systolic pressure (40 to 50 mmHg above systolic pressure) for 5 minutes. Then the cuff will be deflated and the brachial artery diameter will be imaged and recorded for 3 minutes. FMD% more than 10% is considered a normal response. Lower than 10% FMD% reflects endothelial dysfunction, which means a high likelihood to develop cardiovascular event in the future. Subjects with negative FMD% results (the artery is constricted after stress and not dilated as was expected) have the worst prognosis.

- Blood drawn from fasting subjects will be separated and the serum will be frozen until processed as one batch towards the end of the study. Blood will be processed twice – in the beginning of the study and after 3 months.

- Biochemical analysis: soluble pro inflammatory cytokines (interleukin-1, interleukin-6, interleukin-10, VEGFR2, TNF-a).

- Assay of colony forming units: Fresh blood will be processed for cell culture assays for endothelial progenitor stem cells colonies counting (a 5 days' protocol). Fifty milliliter of blood will be processed; peripheral-blood mononuclear cells will be isolated by Ficoll density-gradient centrifugation, will

be washed twice in phosphate buffered saline with 5% fetal bovine serum and re-suspended in media (EndoCult basal media with supplements; StemCell Technologies, Vancouver, British Columbia, Canada) for EPC colony-forming assay. Cells will be planted on human fibronectin-coated plates (BIOCOAT; Becton Dickenson Labware, Bedford, Massachusetts) at a density of $5X10^6$ cells/well and incubated at 37^0C in humidified 5% CO_2. After 48 hours, the non-adherent cells will be re-plated onto fibronectin-coated 24 well plates at a density of $1X10^6$ cells/well. After 5 days, colony forming units (defined as a central core of rounded cells surrounded by elongated and spindle-shaped cells) will be counted manually in 8 wells out of a 24-well plate.

7.2.3 Day 1 Visit

See Table 2 for the procedures and assessment to be performed during the Day 1 visit of the study. The Day 1 visit will occur after all baseline tests are completed and it has been determined that the subject remains eligible. Once the subject is deemed eligible to continue in the study the subject will be administered the investigational product.

7.2.4 Week 2 Visit

See Table 2 for the procedures and assessment to be performed for week 2 through month 6 visit of the study. Outpatient visits should be completed as close to the scheduled visit dates as possible. There will be a +/- window of 2 days for the week 2 study visits.

7.2.5 Month 1 – Month 6 Visits

See Table 2 for the procedures and assessment to be performed for week 2 through month 6 visit of the study. Outpatient visits should be completed as close to the scheduled visit dates as possible. There will be a +/- window of 2 weeks for the Month 1 through month 6 study visits.

7.2.6 Month 12 Visit

See Table 2 for the procedures and assessments to be performed for Month 12 visit. This visit will be conducted via a phone interview with the subject. A phone script will be provided to the study personnel to use when interviewing the subject. There will be a +/- window of 2 weeks for this visit. All fifteen (15) pilot phase subjects will be informed with the option of having additionaladministrations of allogeneic hMSCs at this visit.

7.2.7 Biomarker Assessment

All samples will be identified so that they can be linked to individual subjects. These samples may be stored indefinitely. Individual results will not be returned to the subject or the study physician. The samples will be linked to subjects, but there will be no

recontact. Data presented in publications will not contain individual subjects' clinical characteristics or outcomes; only aggregate data from the entire study will be disclosed.

7.2.8 Immune Monitoring for Graft Rejection

The studies planned in the Cratus protocol will utilize allogeneic mesenchymal stem cells (MSC) in subjects with frailty syndrome. The use of an allogeneic graft raises the potential of graft rejection through immune cells resulting in failure of the therapy. MSCs are ideal candidates for allogeneic transplantation because they show minimal MHC class II and ICAM expression and lack B-7 co-stimulatory molecules necessary for T-cell mediated immune responses[57, 58]. Indeed MSCs do not stimulate a proliferative response from alloreactive T-cells even when the MSCs have differentiated into other lineages or are exposed to proinflammatory cytokines. Previous studies have demonstrated that MSCs have significant immunomodulatory effects, inhibiting T-cell proliferation, prolonging skin allograft survival, and decreasing graft-versus-host disease (GVHD). Recently human MSCs were shown to alter the cytokine secretion profile of dendritic cells, T cells, and natural killer cells in vitro, inhibiting secretion of proinflammatory cytokines (e.g. TNF-a, IFN-γ) and increasing expression of suppressive cytokines (e.g.IL-10), possibly via a prostaglandin E2 mediated pathway.

In vivo studies of the fate of MSCs have shown that, when transplanted into fetal sheep, human MSCs engraft, undergo site-specific differentiation into various cell types, including myocytes and cardiomyocytes and persist in multiple tissues for as long as 13 months after transplantation in non-immunosuppressed immunocompetent hosts. Further, in vivo studies using rodents, dogs, goats, and baboons demonstrate that allogeneic MSCs can be engrafted into these species without stimulating systemic alloantibody production or eliciting a proliferative response from recipient lymphocytes. These findings, coupled with our demonstration of efficacy of these cells for cardiac repair, solidify the notion of using MSCs as an allograft for successful tissue regeneration.

As part of the Cratus protocol we will obtain peripheral blood samples from all subjects to evaluate the presence of activated T cells. Two heparinized (green top) vacutainer tubes (approx. 15 cc total blood) will be collected at different time points during the study: at day one prior to infusion of MSC and at month 6. Peripheral blood mononuclear cells (PBMC) will be isolated from heparinized blood by ficoll sedimentation and will be viably cryopreserved for planned assessments of T cell activation.

Two of the best-accepted markers of T cell activation are CD69 and CD25 (IL-2 receptor α). We will monitor the activation of T cells by flow cytometric analysis of CD3+CD25+CD69+ cells in thawed PBMC. CD69 is an immediate/early marker of CD3+ T cell activation while CD25 expression increases within 1-2 days of activation and remains sustained over the intermediate-long term during chronic immune activation. Given the differences in the kinetics of CD69 and CD25 up regulation, assessment of both activation phenotypes (CD3+CD69+ and CD3+CD25+) will maximize the sensitivity of detection of T cell activation following autologous or allogeneic MSC infusion.

8. SAFETY

8.1 Safety Variables

1. Vital signs
2. Physical examination
3. Clinical laboratory tests
4. Adverse events

8.2 Laboratory Evaluations

At screening, the HIV-1 and HIV-2 tests, CMV, hepatitis screen and β-HCG serum pregnancy tests (only for women of child bearing potential) will be performed locally at the study site. Laboratory safety tests will consist of the following:

Serum chemistry: sodium, potassium, chloride, bicarbonate, BUN, creatinine, glucose, calcium, phosphate, AST/SGOT, ALT/SGPT, total bilirubin (fractionate if total >1.5 times normal), alkaline phosphatase, albumin, fibrinogen, IL6, D-Dimer, Coagulation studies

Hematology (CBC): hemoglobin, hematocrit, platelets, WBC, WBC differential

The Investigator will review all clinically relevant laboratory results requested in the protocol. The diagnosis associated with any clinically significant laboratory deviations should be recorded as an AE and should indicate the underlying abnormality or diagnosis (such as renal insufficiency) as opposed to the observed deviation in laboratory results (such as elevated creatinine). If there is no underlying abnormality linked to a clinically significant abnormal laboratory value, the observed deviation should be reported as the AE.

8.2.1 Pulse Oximetry

Pulse oximetry will be used to observe oxygen saturation when measuring vital signs at screening. Pulse oximetry will also be used throughout infusions and 2 hours following infusions. Subjects requiring oxygen, need the peripheral artery oxygen saturation (SaO_2) to be ≥93% when given a maximum of 2L/minute supplemental O_2 via nasal cannula. Infusion toxicity will be assessed based on decreases in oxygen saturation during infusion. The infusion will be stopped if the oxygen saturation does not return to >93% within 3 minutes of initiating supplemental oxygen or if the subject requires greater than 2L/min supplemental oxygen to achieve the required saturation of >93%. If this occurs then subjects will be admitted to the hospital for observation.

8.2.2 Pregnancy

There is no information allogeneic hMSCs and its effects or potential risks to a fetus or unborn child. The Principal Investigator and DSMB must be notified within twenty-four hours of investigator's awareness of the pregnancy via facsimile if a study subject

becomes pregnant during the study. Males and females of non-childbearing potential ≥ 60 to ≤ 95 years of age at the time of signing the Informed Consent Form with documented FRAILTY must practice any one of the enumerated forms of contraception. Items will be acceptable for meeting the studies contraceptive requirements as listed in section 8.2.2. Females will be defined as non-childbearing potential if surgically sterilized (i.e. bilateral tubal litigation, bilateral oophorectomy, or complete hysterectomy) or post-menopausal (defined as 12 months no menses with an alternative medical cause and with a follicle stimulating hormone (FSH ≥ 25.8 mIU/mL). Non-sterilized males who are sexually active with a female partner of childbearing potential must use any one of the enumerated contraceptive items as listed in section

8.2.2 throughout the study.

Acceptable forms of contraception include: 1) abstinence, 2) condoms (male or female) with a spermicidal agent, 3) diaphragm or cervical cap with spermicidal agent, 4) intrauterine device (IUD), 5) oral contraceptive, 6) injectable or transdermal hormonal contraceptive, 7) successful vasectomy with resulting azoospermia or azoospermia for any other reason, and 8) hysterectomy, bilateral oophorectomy, or tubal ligation.

Prior to study enrollment, women of childbearing potential must be advised of the importance of avoiding pregnancy during trial participation and the potential risk factors for a pregnancy. The subject must sign an informed consent and written authorization for use and disclosure of PHI document stating that the above-mentioned risk factors and the consequences were discussed with her.

8.2.3 Determination of Infusional Toxicity

Infusional toxicity will be evaluated by continuously monitoring the subject's vital signs and O_2 saturation by pulse oximetry from the time of allogeneic hMSCs administration until two hours after infusion is complete. Since there is no specific or antidotal therapy for AEs arising from allogeneic hMSCs, any toxicity that may arise during a subject's participation in this study should be managed with supportive measures at the discretion of the treating physician.

8.2.4 Subject Stopping Guidelines

This guideline is to be used to indicate boundaries requiring discussion by the Data and Safety Monitoring Board (DSMB) and is designed to assist the independent DSMB in overseeing the study. The DSMB may also request additional interim analyses and develop other criteria including provision for monitoring of potential late effects to determine when to intervene in the enrollment or treatment of subjects in the study. The first more conservative stopping guideline is to monitor subjects for unexpected SAEs where there is a reasonable possibility that the study product or administration procedure caused the event within 30 days of administration including subject death, grade 3 myocardial infarction, or grade 3 hemodynamically unstable ventricular

tachycardia. Study accrual and further treatment of subjects will be put on hold if any subjects experience one of these events. The DSMB will be notified within 24 hours of the occurrence of these events and will be convened within 3 business days to review the event and study.

The following are subject stopping guidelines:

1. Any subject who develops persistent (that is, still existing more than 3 hours after the end of IP infusion) cardiorespiratory signs or symptoms (for example, shortness of breath, tachypnea, tachycardia, hypotension, or palpitations) will continue with all scheduled follow-up if such follow-up is considered safe in the opinion of the Investigator.

2. Any subject whose infusion is stopped due to cardiorespiratory distress will receive no further IP infusions but will continue with all scheduled follow-up if such follow-up is considered safe in the opinion of the Investigator.

3. Any subject who develops any sign or symptom that, at the discretion of the Investigator, warrants the discontinuation of infusion will receive no further IP infusions but will continue with all scheduled follow-up if such follow-up is considered safe in the opinion of the Investigator.

4. Infusion of the IP may be stopped if there is an adverse event that the Investigator believes is related to the IP or if there is an issue with the IP infusion.

5. The proportion of subjects experiencing TE-SAE as defined in Section 2.2.1 will be monitored within 30 days of infusion. This guideline is designed to assist the independent DSMB in overseeing the study and indicate boundaries needing discussion by the DSMB. The DSMB may also request additional interim analyses and develop other criteria including provision for monitoring of potential late effects to determine when to intervene in the enrollment or treatment of subjects in the study.

6. Monitoring of key safety endpoints will be conducted. If rates significantly exceed the pre-set threshold, then the DSMB will be advised.

7. The stopping guidelines serve as a mechanism for consultation with the DSMB for additional review, and are not formal “stopping rules” that would mandate automatic closure of study enrollment. It is designed to assist the independent DSMB in overseeing the study. The DSMB may also request additional interim analyses and develop other criteria including provision for monitoring of potential late effects to determine when to intervene in the enrollment or treatment of subjects in the study.

8.2.5 Subject observation and discontinuation after IP administration

The IP administration guidelines in **Appendix 1** list the study requirements for subject observation and discharge after IP administration.

8.3 Definition of an Adverse Event

An Adverse Event (AE) is any untoward medical occurrence in a subject or clinical investigation subject temporally associated with the use of a medicinal product, whether or not considered related to the medicinal product. The occurrence does not necessarily have to have a causal relationship to the treatment received in the study. An AE can therefore be any unfavorable and unintended sign (including an abnormal laboratory finding, for example), symptom, or disease (new or exacerbated) temporally associated with the use of a medicinal product, whether or not considered related to the medicinal product.

Examples of an AE include:

1. Exacerbation of a chronic or intermittent pre-existing condition including either an increase in frequency or intensity of the condition.

2. Significant or unexpected worsening or exacerbation of the condition/indication under study.

3. A new condition detected or diagnosed after study therapy administration even though it may have been present prior to the start of the study.

4. Pre- or post-treatment events that occur as a result of protocol-mandated procedures (e.g., invasive protocol-defined procedures, modification of a subject's previous treatment regimen).

An AE does **not** include:

1. Medical or surgical procedures (e.g., colonoscopy, biopsy). The medical condition that leads to the procedure is an AE.

2. Social or convenience hospital admissions where an untoward medical occurrence did not occur.

3. Day to day fluctuations of pre-existing disease or conditions present or detected at the start of the study that do not worsen.

4. The disease/disorder being studied or expected progression, signs, or symptoms of the disease/disorder being studied unless more severe than expected for the subject's condition.

8.4 Definition of Adverse Reaction

An adverse reaction is any adverse event caused by a drug. Adverse reactions are a subset of all suspected adverse reactions for which there is reason to conclude that the drug caused the event.

8.5 Definition of Suspected Adverse Reaction

Suspected adverse reaction means any adverse event for which there is a reasonable possibility that the drug caused the adverse event. For the purposes of IND safety reporting, 'reasonable possibility' means there is evidence to suggest a causal relationship between the drug and the adverse event. A suspected adverse reaction implies a lesser degree of certainty about causality than adverse reaction, which means any adverse event caused by a drug.

8.6 Definition of Serious

An adverse event (AE) or suspected adverse reaction is considered "serious" if it:

1. results in death
2. is life-threatening (at risk of death at the time of the event)
3. requires in inpatient hospitalization or prolongation of existing hospitalization

   NOTE: Complications that occur during hospitalization are AEs. If a complication prolongs hospitalization or fulfills any other serious criteria, the event is serious. Hospitalization for elective treatment of a pre-existing condition that did not worsen from baseline is not considered to be an AE.
4. results in disability/incapacity

   NOTE: The term disability means a substantial disruption of a person's ability to conduct normal life functions. This definition is not intended to include experiences of relatively minor medical significance such as uncomplicated headache, nausea, vomiting, diarrhea, influenza, accidental trauma (i.e., sprained ankle) that may interfere or prevent everyday life functions but do not constitute a substantial disruption.
5. Is a congenital anomaly/birth defect.

Important medical events that may not result in death, be life-threatening, or require hospitalization may be considered an SAE when, based upon appropriate medical judgment, they may jeopardize the subject and may require medical or surgical intervention to prevent one of the outcomes listed in the above definition.

8.7 Definition of Unexpected

An adverse event or suspected adverse reaction is considered "unexpected" if it is not listed in the investigator brochure or is not listed at the specificity or severity that has been observed; or, if an investigator brochure is not required or available, is not consistent with

the risk information described in the general investigational plan or elsewhere in the current application.

8.8 Clinical Laboratory Assessments and Other Abnormal Assessments as Adverse Events and Serious Adverse Events

Abnormal laboratory findings (e.g. clinical chemistry, hematology) or other abnormal assessments (e.g., vital signs) that are judged by the Investigator as clinically significant will be recorded as AEs or SAEs if they meet the definition of an AE as defined in Section 8.3 (“Definition of an Adverse Event”) or SAE, as defined in Section 8.6 (“Definition of a Serious Adverse Event”). Clinically significant abnormal laboratory findings or other abnormal assessments that are detected during the study or are present at screening and significantly worsen following the start of the study will be reported as AEs or SAEs. However, clinically significant abnormal laboratory findings or other abnormal assessments that are associated with the disease being studied, unless judged by the Investigator as more severe than expected for the subject’s condition, or that are present or detected at the start of the study but do not worsen, will not be reported as AEs or SAEs.

The Investigator will exercise medical judgment in deciding whether abnormal laboratory values are clinically significant.

8.9 Recording of Adverse Events and Serious Adverse Events

The Investigator should review all documentation (e.g., hospital progress notes, laboratory, or diagnostic reports) relative to the event being reported. The Investigator will then record all relevant information regarding an AE/SAE into the electronic data system. It is not acceptable for the Investigator to send photocopies of the subjects’ medical records in lieu of completion of the appropriate AE/SAE pages.

The Investigator will attempt to establish a diagnosis of the event based on signs, symptoms, and/or other clinical information. In such cases, the diagnosis should be documented as the AE/SAE and not the individual signs and symptoms.

SAEs will be reported to the IRB within 10 working days or within 24 hours if the event is life-threatening or results in death.

Pregnancies

Subject pregnancy must be reported to the Principal Investigator within 1 working day of knowledge of the event. Any subject that becomes pregnant during the study must be promptly withdrawn from the study. Follow-up information regarding the outcome of the pregnancy and any postnatal sequelae in the infant will be required.

8.10 Intensity of Adverse Events and Serious Adverse Events

The Investigator will make an assessment of intensity for each AE and SAE reported during the study. The assessment will be based on the Investigator's clinical judgment. The intensity of each AE and SAE should be assigned to one of the following categories:

Mild:	An event that is easily tolerated by the subject, causing minimal discomfort and not interfering with everyday activities.
Moderate:	An event that is sufficiently discomforting to interfere with normal everyday activities.
Severe:	An event that prevents normal everyday activities.
Life-threatening:	Immediate risk of death.

An AE that is assessed as severe should not be confused with an SAE. Severity is a category utilized for rating the intensity of an event; and both AEs and SAEs can be assessed as severe. An event is described as 'serious' when it meets one of the pre-defined outcomes as described in Section 8.6, "Definition of Serious."

8.11 Causality of Adverse Events and Serious Adverse Events

The Investigator is obligated to assess the causality between study therapy and the occurrence of each AE/SAE. The Investigator will use clinical judgment to determine if there is a reasonable possibility that the biological action of the study therapy was responsible for AE/SAE being reported. Alternative causes such as natural history of the underlying diseases, concomitant therapy, other risk factors, and the temporal relationship of the event to the study therapy will be considered and investigated. The Investigator will also consult the Clinical Investigator's Brochure and/or Product Information, for marketed products, in the determination of his/her assessment.

The Investigator will use the following questions when assessing causality of an adverse event to study therapy.

Is there a reasonable possibility that the study therapy caused the event? Reasonable possibility implies that there is evidence that the vent was caused by the study product. An affirmative answer designates the event as a suspected adverse reaction.

There may be situations when an SAE has occurred and the Investigator has minimal information to include in the initial report. However, it is very important that the Investigator always make an assessment of causality.

The relationship between AEs and the study exposure will be classified by the investigator as:

1. None: No relationship. Related to other known etiologies, conditions, or exposures.
2. Unlikely: Current knowledge suggests that a relationship is unlikely.

3. Possible: A plausible temporal sequence or response pattern exists but the AE may be related to other known etiologies, conditions, or exposures.
4. Probable: A plausible temporal sequence or response pattern exists and the AE cannot be related other known etiologies, conditions, or exposures.

5. Definite: A plausible temporal sequence or response pattern exists and the AE can be confirmed by re-challenge or with other supporting data.

8.12 Follow-Up of Adverse Events and Serious Adverse Events

After the initial AE/SAE report, the Investigator is required to proactively follow each subject and provide further information on the subject's condition. All AEs and SAEs documented at a previous visit/contact that are designated as ongoing will be reviewed at subsequent visits/contacts.

Adverse events and SAEs will be followed until resolution, until no further changes in the event are expected (i.e. the point at which a subject experiencing a critical adverse event is treated successfully and stabilized even though they may continue to experience lingering sequelae that may never resolve), until the subject is lost to follow-up, or until it is agreed that further follow-up of the event is not warranted (e.g. non-serious, study therapy unrelated, mild or moderate adverse events ongoing at a subject's final study visit). If a subject dies during participation in the study or during a recognized follow-up period, the Investigator will provide a copy of any post-mortem findings, including histopathology.

New or updated information will be recorded by modifying the AE forms in the electronic data system

8.13 Timeframes for Submitting SAE Reports

Once an Investigator becomes aware that an SAE has occurred in a study subject, he/she will record the information in the electronic data record within 48 hours. Any fatal or life-threatening event must be reported within 24 hours. If the Investigator does not have all information regarding an SAE, he/she will not wait to receive additional information before recording the event in the data system and completing as much information known at the time of the submission. The reporting timeframes for any SAE occurring during the study are summarized in Table 3.

<u>TABLE 3</u>
Serious Adverse Event Reporting Requirements

	Initial Reports		Follow-Up Reports
**Type of SAE**	Fatal or Life-Threatening	Other SAEs	Any SAE
**Reporting Timeframes**	24 hours	48 hours	48 hours
**Documents Required**	24 hours:<br>Complete as much information in the electronic data system that is known.<br>48 hours:<br>Fully complete all AE forms	Fully completed AE forms	Updated AE Forms

8.14 Post-Study Adverse Events and Serious Adverse Events

The Investigator should report any death or SAE occurring at any time after a subject has completed or terminated a clinical trial, when such death or SAE may reasonably be related to the study therapy used in an investigational trial. Investigators are not obligated to actively seek AEs from former study participants.

8.15 Regulatory Aspects of Adverse Event Reporting

The Investigator will promptly report all SAEs within the timeframes specified in Section 8.13. Prompt notification of SAEs by the Investigator is essential so that UMMSM can meet legal obligations and fulfill ethical responsibilities towards the safety of all subjects participating in UMMSM-sponsored investigational trials.

The Investigator will comply with the applicable local regulatory requirements related to reporting of SAEs to his or her Institutional Review Board (IRB) or Independent Ethics Committee (IEC).

This protocol has been filed under an Investigational New Drug (IND) application with the FDA. A given SAE may qualify as an Expedited Safety Report (ESR) if the SAE is both at least possibly attributable to study therapy and unexpected. In this case, all Investigators participating in an IND study will receive an ESR.

The ESRs are prepared according to UMMSM policy and are forwarded to the Investigator as necessary. The purpose of the ESR is to fulfill specific regulatory and Good Clinical Practice (GCP) requirements regarding the product under investigation. Based on previous trials involving intravenous infusion of allogeneic human MSCs, no AEs have been attributed to treatment administration; therefore all AEs will be considered and documented as unexpected AEs.

All AEs occurring at any time during the trial will be collected, documented, and reported by the investigator. For each AE, the investigator will provide the date of onset and resolution, intensity, treatment required, outcome, seriousness, and potential causality with regards to the study exposure.

9. STATISTICAL ANALYSIS

9.1 Determination of Sample Size and Analysis Population

No formal statistical justification was performed to determine sample size in the Phase I study. Cohort size was determined based on expected requirements for safety analyses and projected enrollment rates. Study participants will be randomized according to a fixed allocation permuted block randomization schema. The allocation ratio will be 1:1:1 and the block size will be 4. All enrolled subject who received at least one treatment dose will be included in summaries of baseline characteristics, safety, and efficacy. Reasons for study discontinuation will be tabulated.

9.2 General Statistical Methods

All statistical tests will be performed at an $\propto$=0.05 level of significance, using two-sided tests. Because this is a Phase I study with only exploratory efficacy outcomes, no adjustments will be made for multiple analyses. Continuous variables will be presented by descriptive statistics. Categorical variables will be presented by counts. Two sided 95% confidence intervals will be calculated and presented where appropriate.

Analysis of AEs will include tabulation by frequency, severity, organ system affected, and relationship to study exposure. Lung function data will be summarized descriptively. Subject reported outcome data will be summarized according to the guidelines of each questionnaire.

9.3 Interim Analyses

Interim analyses will be conducted at times coincident with regularly scheduled meetings of the Data and Safety Monitoring Board (DSMB) at approximately six-month intervals. The DSMB Chair will be notified each time an SAE occurs. After all subjects in phase I have been followed for 30 days, at that time an independent DSMB will review all available data to make an independent recommendation to either keep the specified randomized dose 1:1:1 or to recommend a dose modification for the randomized placebo study.

These should not be considered formal interim analyses as no hypothesis testing will be done.

Policies of the DSMB will be described in the DSMB Charter, which will be prepared by the DSMB prior to study initiation. The stopping guidelines serve as a trigger for consultation with the DSMB for additional review, and are not formal “stopping rules” that would mandate automatic closure of study enrollment.”

9.4 Data and Safety Monitoring Board (DSMB)

9.4.1. ROLE OF THE DSMB

This study is designed to test the safety of hMSCs in subjects with frailty.

The purpose of the data and safety monitoring board (DSMB) is to advise the investigators regarding the continuing safety of study subjects and those yet to be recruited to the study, as well as the continuing validity and scientific merit of the study.

This section describes the roles, responsibilities and operating procedures of the DSMB, and includes guidelines for communications and interactions between the DSMB and the investigators to schedule and format for meetings; format for presentation of data; specification of who will have access to interim data and who may attend all or part of DSMB meetings; procedures for assessing conflict of interest of potential DSMB members; and the method and timing of providing interim reports to the DSMB.

9.4.2. Purpose of the DSMB

The primary function of the DSMB is to review the accumulating unblinded safety data from each study group and using the data as the basis for recommendations concerning the continuation and/or modification of the study. This will be accomplished through regularly scheduled formal meetings and/or additional meetings to review interim summaries of safety and efficacy data. The DSMB will make recommendations regarding modification or termination of the study in the event of significant study conduct issues or safety concerns. The DSMB will not stop the study based on efficacy results favorable to hMSCs, other than for all-cause mortality as outlined below. The selected primary and secondary endpoints were chosen to measure major morbidity in subjects with FRAILTY, a fatal disease. Given the importance of mortality in FRAILTY, a stopping boundary based on the all-cause mortality rate will be implemented to guide the DSMB. This stopping boundary will not be applied until after all subjects have enrolled in the study.

9.4.2.1 <u>DSMB MEMBERSHIP</u>

The DSMB is an independent, multidisciplinary group consisting of four members (inclusive of the DSMB chair). The members include a clinical trialist, a biostatistician, an expert gerontologist and an expert cardiologist.

The DSMB will meet until the study’s database has been locked and a final data review has been completed. If a member withdraws from the DSMB, the DSMB chairperson will be responsible for selecting an appropriate replacement.

9.4.2.2 <u>Financial Disclosure and Conflict of Interest</u>

DSMB membership is restricted to individuals without significant potential or perceived conflicts of interest. The source of these conflicts may be financial, scientific, or regulatory in nature.

Members must disclose to the DSMB chairperson their consultancies (direct or indirect) in excess of $5,000 or financial interests in any pharmaceutical companies, biotechnology companies, or CROs, if these relationships could lead to any conflict of interest or these companies' products involve hMSCs.

The DSMB chairperson will be responsible for deciding whether consultancies or financial interests of the members materially impact their objectivity. This decision is to be based on the reasonable belief that their objectivity is not in doubt. DSMB members will be responsible for advising the DSMB chairperson of any changes in financial interests in pharmaceutical companies, biotechnology companies, including consultancies, during the course of their membership. Members of the DSMB who develop significant potential or perceived conflicts of interest that may materially impact their objectivity will be asked to resign from the DSMB.

9.4.2.3 DSMB Responsibilities

The DSMB has the responsibility to:

1. Review the protocol and all amendments. The DSMB will also be provided the Investigator's Brochure, as it is updated, that includes preclinical and clinical efficacy and safety data.

2. Review the statistical analysis plan with particular attention to the portions describing the data to be provided to the DSMB.

3. Evaluate the conduct of the study including the selection and retention of subjects, extent of protocol deviations, treatment adherence, and the quality of the data. The DSMB shall make recommendations regarding study conduct as necessary to protect the scientific integrity of the study. The DSMB shall not make recommendations regarding the efficacy outcomes or associated analyses.

4. Review unblinded periodic safety summaries including adverse events, serious adverse events, discontinuations, and post-baseline laboratory results. In addition, efficacy and mortality data will be reviewed, although the DSMB shall not recommend stopping the study for efficacy.

5. Evaluate the conduct of the study including enrollment rates, the selection and retention of subjects, protocol deviations, treatment adherence and quality and completeness of the data

6. The DSMB will make recommendations regarding modification or termination of the study in the event that significant safety concerns arise during study conduct.

9.4.2.4 Confidentiality

The DSMB will be unblinded in its assessment of safety and efficacy data to ensure that the DSMB is fully informed in its primary mission of safeguarding the interest of participating subjects. The DSMB will have sole access to comparative results of safety data aggregated by treatment arm. The DSMB will take all necessary and appropriate steps to safeguard the confidentiality of unblinded treatment information it receives to minimize the potential for premature conclusions regarding the study results as well as the potential for introducing bias into the study.

9.4.2.5 Study Conduct and Termination

The DSMB will provide recommendations following review and assessment of the quality of study conduct. More specifically, the DSMB will review enrollment rates, consistency in complying with eligibility requirements, compliance with the study protocol as well as the completeness of the data. In their review of the data, the DSMB will be responsible for protecting the safety of the enrolled subjects. If any potential question of safety arises, the DSMB will use the efficacy data to assess the possible safety risk in the context of the benefit-to-risk profile of study treatment. Based on this information, the DSMB may make recommendations to terminate the study if members believe that an undue risk (relative to benefit) would be incurred by allowing the study to continue to completion. Otherwise the study will be completed to allow investigators to complete the protocol mandated assessments to evaluate the safety of hMSCs in FRAILTY subjects.

9.4.2.6 Investigator Responsibilities

The investigator has the responsibility to:

1. Make decisions based on DSMB recommendations in a timely fashion.
2. Notify study centers of the outcome of the DSMB meetings, and any DSMB recommendations addressing actions to be taken to ensure the integrity of the study.
3. Notify regulatory agencies of DSMB recommendations addressing any emerging safety concern not recognized at the start of the study.
4. Ensure that the unblinded DSMB support team is provided with the data necessary for the chosen analyses and reports.
5. Provide DSMB members with the current protocols and Investigator’s Brochure.
6. Provide DSMB members with PSURs as published
7. Attend the open session of each DSMB data review meeting.

9.4.3 COMMITTEE MEETINGS

9.4.3.1 Organizational Meeting

At an organizational meeting the DSMB will discuss the operational aspects of the committee. This meeting will include DSMB members and the clinical monitor. The documents to be provided before this meeting are:

1. Study protocol
2. Preliminary DSMB Statistical Analysis Plan (SAP)
3. Preliminary list of tables and listings to be provided for interim assessments
4. Investigator's Brochure
5. Food and Drug Administration Guidance: *Establishment and Operation of Clinical Trial Data Monitoring Committees.*

For all DSMB meetings, a quorum is defined as at least two members of the DSMB in addition to the DSMB chairperson.

9.4.3.2 Review of Periodic Safety Update Reports

As part of ongoing safety review and obligation to regulatory agencies, safety reports will be furnished. These reports will be available to the DSMB, who shall review them in the context of providing additional information to assist the committee's consideration of subject safety. The DSMB members may choose to discuss the PSURs during an ad hoc teleconference.

9.4.3.3 Data Review Meetings

After all subjects enrolled in the Pilot Phase have received the study therapy infusion and been followed for 30 days, the DSMB will conduct a full review of all cumulative safety data before the trial proceeds to the Randomized Phase. As part of the cumulative safety data review meeting for the pilot phase, the DSMB will recommend that the trial proceed to the protocol-specified randomized phase or recommend a dose modification for the randomized placebo study.

Ongoing Monitoring During Randomized Phase

Formal data review meetings that include the entire DSMB will be conducted via teleconference approximately every three months. The timeline for the quarterly DSMB data review meetings will begin after approximately 25% of subjects are enrolled in the randomized phase. Meetings may be postponed due to accrual rates at the DSMB's discretion. The purpose of the data review is for safety evaluation, and the study may be stopped because of significant safety concerns. SAEs which are related to stopping rules will be continuously evaluated and the full DSMB will be informed of any extra risk.

Other safety data available at each evaluation, such as 12-Lead ECGs and laboratory data will also be evaluated by the DSMB as appropriate (see Table 4).

TABLE 4

Additional Safety Data

Study Procedure	Screening<br>Day -56 +/- 28 Days	Baseline Day -14 (Weeks -4 to -2)	Day 1	Week 2 (Day 14)	Month 1<br>Week 4	Month 3<br>Week 12	Month 6<br>Week 24	Month 12<br>Week 48
History and Physical	x	x	x	x	x	x	x	
Vital Signs	x	x	x	x	x	x	x	
12-Lead ECG	x	x	x	x	x	x	x	
Concomitant Medications	x	x	x	x	x	x	x	x
FEV-1, 6 Minute Walk Test		x				x	x	
QOL Questionnaires		x			x	x	x	
Hematology, Clinical Chemistry	x		x	x	x	x	x	
Urinalysis	x				x	x	x	
Serum or Urine Pregnancy Test	x		x					
Adverse Events			x	x	x	x	x	x

The DSMB will evaluate all safety data available for each subject as appropriate. In addition to those assessments listed in Table 4, data for DEXA scan, Dobutamine stress echocardiogram test, dynamometer test, and specific biomarkers may be available (See Table 2, CRATUS clinical protocol).

The EMMES Corporation will coordinate the scheduling of the teleconferences to review safety data during the randomized phase.

Open session

Blinded data will be provided to the DSMB approximately 1 week before each data review meeting. The report will contain:

1. Protocol status including any protocol changes
2. Data sources and cutoff dates
3. Analysis methods applied specifically to the open session report
4. Subject enrollment by month
5. Protocol deviations
6. Early treatment discontinuations and study withdrawals
7. Demographic and baseline characteristics
8. Duration of follow-up at time of data cutoff

Closed Session

Only the DSMB members will participate in closed sessions. Unblinded data will be presented to the DSMB in closed session and discussed by the DSMB. "Unblinded" means that the name of actual treatment arm is associated with individual subject data listings and summaries of data. At the chairperson’s discretion, the DSMB may discuss or vote on potential study conduct recommendations at closed session.

The closed session report will contain data separated and identified by treatment group. This report (hard copy) will be provided to the DSMB approximately 1 week before each data review meeting and will include:

1. Data sources and cutoff dates
2. Analysis methods applied specifically to the closed session report
3. Subject enrollment
4. Protocol deviations
5. Early treatment discontinuations and study withdrawals
6. Demographic and baseline characteristics
7. Primary and secondary efficacy outcome measures
8. Prohibited concomitant medications
9. Adverse events
10. Serious adverse events

Following the closed session, the DSMB chairperson will issue one of the following recommendations as determined by the DSMB:

1. Continue the study, with or without modifications.
2. Terminate the study for safety concerns.

Separate meeting minutes for the open and closed sessions will be prepared by the DSMB support team, then reviewed and issued by the DSMB chairperson. The DSMB support team will distribute the finalized minutes. The DSMB support team will maintain copies of the meeting minutes for both the open and closed meetings. To preserve the integrity of the study, the detailed rationale and discussion of comparative unblinded data will be included only in the closed meeting minutes

Follow-Up Open Session

Immediately following the closed session, the DSMB will meet with the investigators to discuss any study conduct concerns. This follow-up open session may be attended by DSMB Members, clinical monitor, and study staff. Potential recommendations from the DSMB regarding study discontinuation or continuation, with or without modification, will not be communicated in this open session.

Closed Executive Session

At the discretion of the DSMB chairperson, a closed executive session may be held. Closed executive sessions will include only DSMB members. Discussion of unblinded study data and potential DSMB recommendations and voting may take place in closed executive session. The DSMB may choose whether to write minutes of the closed executive sessions.

9.4.4 DATA FLOW

9.4.4.1 Communications and Reports

For each DSMB meeting, the open session and closed session reports will be prepared by the DSMB support team. Open session reports will be circulated to all attendees of the open session. The closed session reports will be circulated to DSMB members only. The closed session reports will be retrieved from the DSMB members by the DSMB support team and destroyed after the closed session. The DSMB support team will maintain copies of all reports from the open and closed sessions.

9.4.4.2 Review of Unblinding Requests

Except as required by regulatory authorities for safety reporting, individual subjects' treatment assignments will not be unblinded during the conduct of the study, unless a subject safety issue arises in which unblinding is necessary to ensure optimal subject management. It is not anticipated that unblinding will be necessary, given the hMSCs can be safely discontinued at any time a safety concern arises. The DSMB will be informed in a timely manner of any case for which unblinding was requested and performed.

9.4.4.3 DSMB Additional Analysis Requests

The DSMB may request additional analyses from the statistician if deemed necessary to fulfill the mission of the DSMB. If based on the additional data the DSMB feels there is a need for an unscheduled formal meeting, the DSMB chairperson will arrange.

9.4.4.4 Confidentiality

All documents will be held in strict confidence by the DSMB, and all documents provided to the DSMB will be collected and destroyed at the end of all DSMB meetings by the DSMB support team.

9.4.5 COMMUNICATION

9.4.5.1 DSMB Minutes

The DSMB chairperson is responsible for issuing minutes of the open and closed sessions. Minutes of the open session will be distributed to all open meeting attendees within two weeks of the meeting. Minutes of the closed session will be distributed to the members of the DSMB within two weeks of the meeting. At the conclusion of the study, the DSMB support team will send a complete set of the open and closed reports,

minutes of the open and closed sessions with the tables and listings, all presentations and copies of the recommendation forms to the investigators.

9.4.5.2 DSMB Recommendations

If the DSMB recommends modification to, or termination of, the study, the chairperson of the DSMB will contact the investigators within 24 hours of making the decision.

Recommendations for modifications other than termination should be accompanied by the rationale for the recommendation and the minimum amount of data required to make a decision. The investigator will be responsible for promptly reviewing the DSMB recommendations and determining whether amendments to the protocol or changes regarding the study conduct are required and if reporting to regulatory authorities is warranted (FDA, 2006).

Should there be disagreement between the DSMB and the investigators around the decision to stop or modify the study a separate compliance committee will be appointed. This committee is comprised of individuals who have extensive experience in the pharmaceutical industry and a deep appreciation of the ethical issues surrounding the conduct of clinical studies and are responsible for the investigator's code of ethics. The Compliance Committee is charged with independently evaluating differing opinions that may arise between the investigator and DSMB and applying the highest ethical standards with respect to determining the best interests of subjects enrolled in the study.

10. STUDY ADMINISTRATION

10.1 Regulatory Authority Approval

This study will be conducted in accordance with Good Clinical Practice (GCP) requirements described in the current revision of International Conference on Harmonization of Technical Requirements of Pharmaceuticals for Human Use (ICH) Guidelines and all applicable regulations, including current United States Code of Federal Regulations (CFR), Title 21, Parts 11, 50, 54, 56, and 312 and Title 45, Part 164. Compliance with these regulations and guidelines also constitutes compliance with the ethical principles described in the current revision of the Declaration of Helsinki. This study will also be carried out in accordance with local legal requirements.

10.2 Ethics Approval

It is the Investigator's responsibility to ensure that prior to initiating this study; this protocol is reviewed and approved by the appropriate local IRB. The composition and conduct of this committee must conform to the United States CFR.

The IRB/IEC must also review and approve the site's informed consent form (ICF), other written information provided to the subject and all advertisements that may be used for subject recruitment.

If it is necessary to amend the protocol or the ICF during the study, the Investigator will be responsible for ensuring that the IRB/IEC reviews and approves these amended documents. An IRB/IEC approval of the amended protocol and/or ICF must be obtained in writing before implementation of the amended procedures and before new subjects are consented to participate in the study using the amended version of the ICF.

10.3 Subject Informed Consent

Before being admitted to the clinical study, all subjects must consent in writing to participate. An ICF will be given to each subject, which will contain all United States federally required elements, all ICH-required elements, and Health Insurance Portability and Accountability Act Authorization (HIPAA) information in language that is understandable to the subject.

The process of obtaining the informed consent will be in compliance with all federal regulations, ICH requirements, and local laws.

The investigator or designee will review the study with each subject. The review will include the nature, scope, procedures, and possible consequences of the subject's participation in the study. The ICF and review must be in a form understandable to the subject. The Investigator or designee and the subject must both sign and date the ICF after review and before the subject can participate in the study. The subject will receive a copy of the signed and dated form, and the original will be retained in the site study files. The Investigator or his/her designee must emphasize to the subject that study participation is entirely voluntary and that consent regarding study participation may be withdrawn at any time without penalty or loss of benefits to which the subject is otherwise entitled.

If the ICF is amended during the study, the Investigator must follow all applicable regulatory requirements pertaining to approval of the amended ICF by the IRB/IEC. The site must use the amended consent form for all new subjects and repeat the consent process with the amended ICF for any ongoing subjects.

In cases where a new ICF is issued between the Month 6 (Office Visit) and Month 12 (Telephone visit) the subject will be contacted and informed of the changes. The subject will be asked if they are available to come to the site. If the subject is unable to come to the site the subject may be reconsented via phone.

10.4 Confidentiality of Information

Subjects' names will remain confidential and will not be included in the database. Only subject number, subject initials, and birth date will be recorded in the data system. If the subject name appears on any other document collected (e.g., hospital discharge

summary), the name must be deleted before the document is transmitted. All study findings will be stored in electronic databases. The subjects will give explicit permission for representatives of regulatory authorities and the IRB/IEC to inspect their medical records to verify the information collected.

Subjects will be informed that all personal information made available for inspection will be handled in the strictest confidence and in accordance with all state, local, and federal data protection/privacy laws, including, without limitation, the HIPAA.

Participants will be asked to voluntarily provide written authorization prior to requesting or disclosing private health information either as part of the written ICF or as a separate authorization form. The authorization will contain all required elements specified by 45 CFR 164, and will allow the site to access study-related private health information until the conclusion of the clinical study. The authorization will remain valid and in full force and effect until the first to occur of (1) the expiration of two years after the study therapy is approved for the indication being studied, or (2) the expiration of two years after the research program is discontinued. Individual subject medical information obtained during this study is confidential and its disclosure to third parties (other than those mentioned in this Section) is strictly prohibited. In addition, medical information obtained during this study may be provided to the subject's personal physician or to other appropriate medical personnel when required in connection with the subject's continued health and welfare.

The investigator will maintain a personal subject identification list (subject and treatment numbers with the corresponding subject names) to enable records to be identified.

10.5 Payments to Subjects

Subjects will be reimbursed $25 at the end of each follow-up visit (Month 1 – Month 6) for a total remuneration of $75. These disbursements are meant to cover the time required to complete these study visits and all necessary travel and parking expenses.

Normal donors for generation of allo-MSC will be reimbursed $350 at the end of BM aspiration. This payment will compensate donors for lost time, parking, and travel expenses.

APPENDIX 1: Infusion Guidelines

Prior to the start of the infusion the following procedures and assessments will be conducted on the study subject:

1. Vital Signs: Blood pressure, heart rate, respiratory rate, and temperature, will be measured within 15 minutes prior to the initiation of the infusion.
2. Oxygen saturation will be continuously monitored by pulse oximetry for at least 30 minutes prior to initiation of IP infusion.
3. Confirm that IV access is established and that the IV catheter is no smaller than 20 gauge
4. Study personnel needs to verify that the following pre-medications have been administered 30 minutes prior to infusion per protocol:

   - Hydrocortisone 25 – 50 mg IV
   - Diphenhydramine (Benadryl) 25 – 50 mg IV

   Note: No other medications should be given during the infusion unless determined medically necessary by the Investigator.

5. Document pre-medications given prior to infusion on the source documents
6. Required IV Infusion materials as follows:
   - 0.9 % normal saline IV infusion bag
   - IV Pump tubing
   - IV extension tubing (unless using a central line)
   - Volumetric infusion pump
   - Gloves
7. Remove 0.9% normal saline infusion bag and connect IV tubing to the volumetric infusion pump
8. Cover the IV tubing with the blinding material provided with the infusion bag by the drug preparation technician.

During the IP infusion the following procedures and assessments will be conducted on the study subject:

1. Monitor the subject continuously with pulse oximetry
2. Hang the blinded infusion bag. Investigational product (IP) should not be “piggybacked” through another line
3. Intravenously administer the IP at a rate of 2ml/min.
   Note: Study personnel administering the IP must be present throughout the infusion process. The Investigator must be available at the site during the infusion process in case an emergency should arise.
4. Record the start time of the infusion bag
5. Gently squeeze the infusion bag several times every 15 minutes to assure uniform dispersion of contents

6. Vital signs and O_2 saturation will be measured every 15 minutes until the end of IP infusion
7. Record the total volume infused from the IP bag
8. At the end of the IP infusion, close the line and flush 25ml of 0.9% normal saline into the luer lock connector on the bottom of the IP bag, reopen line and allow to infuse at a rate of 2mL/min until completion.
9. Discard IV tubing according to established guidelines
10. Return the blinded IP infusion bag to the cell-processing technician.

Procedures post-infusion:

1. Vital signs will be monitored at 15 minutes, 30 minutes, 1 hour, and 2 hours post IP infusion
2. The subject will be monitored for a minimum of 2 hours post IP infusion with continuous pulse oximetry
3. If the O_2 saturation decreases to < 90% over a continual period of 3 – 5 minutes then supplemental oxygen may be added or increased during the two-three hours post-infusion observation period.
4. If at the end of the 2 hour observation period, if a subject's O_2 saturation stays below 90% then the subject will be provided additional oxygen to maintain a saturation of >90% at room air up to 4 hours post infusion.
5. After the minimum two hour observation period, the subject will be continuously monitored and discharged, if no complaints are experienced, such as shortness of breath or other objective signs of cardiorespiratory compromise.
6. Subjects not meeting criteria for discharge will be assessed by the Investigator during the observation period to further determine hospitalization otherwise not specified in the protocol.

<u>Subject Stopping Guidelines:</u>

1. Any subject who develops persistent (that is, still existing more than 3 hours after the end of IP infusion) cardiorespiratory signs or symptoms including hypoxemia (defined per oxygenation criteria of 93% on room air at rest, or shortness of breath, tachypnea, tachycardia, hypotension, or palpitations) will continue with all scheduled follow-up if such follow-up is considered safe in the opinion of the Investigator. The infusion will be stopped if the oxygen saturation does not return to >93% within 3 minutes of initiating supplemental oxygen or if the subject requires greater than 2L/min supplemental oxygen to achieve the required saturation of >93%. If a subject requires the addition of oxygen, it will be continued for 4 hours after the completion of the infusion. At that time, oxygen will be weaned off to maintain a saturation >93% on room air.

2. Any subject whose infusion is stopped due to cardiorespiratory distress will receive no further IP infusions but will continue with all scheduled follow-up if such follow-up is considered safe in the opinion of the Investigator.

3. Any subject who develops any sign or symptom that, at the discretion of the Investigator, warrants the discontinuation of infusion will receive no further IP infusions but will continue with all scheduled follow-up if such follow-up is considered safe in the opinion of the Investigator.

4. Infusion of the IP may be stopped if there is an adverse event that the Investigator believes is related to the IP or if there is an issue with the IP infusion.

Addendum A: Pilot Subjects Optional Follow-on Phase

A.1 Rationale and Description of second infusion for first 15 subjects on pilot phase

This addendum protocol is designed to test the safety of a second infusion of allogeneic mesenchymal stem cells 12 to 18 months following a first infusion.

Subjects in the run-in phase received one of 3 doses of allogeneic MSCs and are eligible to participate in this optional follow-on phase. Because several studies, reviewed below, have now shown safety of repeat doses of MSCs in a variety of medical conditions, additional safety testing in the subject population of aging frailty enrolled in the CRATUS trial is required[92]. All study specific processes and procedures included in the pilot phase of the protocol apply in this addendum unless otherwise noted.

A.2 Demonstrated Safety and Increased Efficacy of Multiple Infusions of Allogeneic Mesenchymal Stem Cells

Active clinical trials and ongoing preclinical work provide an accumulating data set supporting the safety and, in some cases, therapeutic efficacy of allogeneic mesenchymal stem cells (MSCs). Allogeneic mesenchymal stem cells are both immunoprivileged and immunosuppressive, thereby enabling their use as an allograft[93]. Cellular therapy with allogeneic MSCs has abundant support from both animal studies and human clinical trials for a wide range of disorders (Table 1). For example, the POSEIDON study addressed the major issue of the use of allogeneic MSCs as a cell-based therapeutic[18]. In this study, subjects with cardiac failure were randomized to receive either autologous MSCs or identically prepared allogeneic MSCs from healthy donors.
Accumulating evidence also supports the concept that repeated doses and/or co-administration of allogeneic MSCs could further enhance therapeutic outcomes. This is an extremely important area for medical investigation, since repeat dosing could potentially have an additive effect and/or reverse disease pathology, depending on the disorder.

Recently, several clinical studies have demonstrated that multiple infusions of allogeneic MSCs are well tolerated with minimal side-effects. Importantly, repeat dosing has no increased level of side effects compared with single doses. For example, results from Franco Locatelli's group[92] clearly demonstrate the safety and efficacy of multiple allogeneic MSC infusions in children with steroid-refractory acute graft versus host disease (aGvHD). Doses in these studies were 1-2x10^6 MSCs/kg recipient body mass, and each child received on average 2 doses (range was 1-13 doses) separated on average by 15 days (range 3-43 days). The results of this study indicated increased effectiveness when the therapy was commenced early in the disease. Furthermore, the study indicated the therapeutic benefits of repeat doses to subjects who did not achieve complete remission after a single dose.

Koc et al[94]; further investigated Hurler syndrome (mucopolysaccharidosis type-IH) and metachromatic leukodystrophy (MLD) and studied allogeneic MSCs based on their potential to differentiate into cells of bone, cartilage, tendon, muscle and other adventitial tissues and offer potential for corrective cellular therapy[95]. Their results demonstrated no infusion-related toxicity, improvement in nerve conduction, and bone mineral density was either maintained or improved in all subjects.

A Phase II study on the 3-year efficacy of allogeneic MSCs in the treatment of system lupus erythrematosus (SLE) was reported by Lingyun Sun's group[96]. The results of this study demonstrated the therapeutic efficacy of allogeneic MSCs in treating SLE. However, no advantage was found by administering a second or higher dose of allogeneic MSCs. Despite this lack of additional efficacy over a single dose, the results of this study confirmed the safety of multiple infusions of allogeneic MSCs. The doses ($1x10^6$ MSCs/kg body mass) were administered 1 week apart. Another study reported on the use of repeated dosing of allogeneic MSCs in subjects with chronic obstructive pulmonary disease. The study showed minimal therapeutic efficacy after 2 years[19]. Importantly, in this subject population repeated allogeneic MSC infusions, which were given 4 times at 30 day intervals ($1x10^8$ MSCs/infusion) were safe.

Taken together, these various clinical studies provide a solid rationale for repeat allogeneic MSC infusions. In no case did the administration of multiple doses lead to a significant increase in the frequency of adverse effects, or to a decrease in therapeutic efficacy over a single dose. In many cases, repeated infusions indeed improved clinical outcomes, demonstrating an additive effect of allogeneic MSC therapy. Given these promising results, investigations are now warranted to determine optimal dosing frequency and total doses of allogeneic MSCs to administer.

It is particularly important to establish safety of repeat doses of allogeneic MSCs in the subject population enrolled in the CRATUS study. These individuals are of older age and are not represented in the previous studies described. In this follow-on study participants in an earlier open label run-in study will be asked to participate in this repeat dosing protocol.

A.3 STUDY OBJECTIVES AND ENDPOINTS

A.3.1 Study Objectives

A.3.1.1 Primary Objective

1. Demonstrate the safety and tolerability of a second intravenous infusion of allo-hMSCs in subjects with aging frailty who had previously received an infusion of allogeneic MSCs as part of the pilot phase of the protocol.

A.3.1.2 Secondary Objectives

1. To explore treatment efficacy (decrease in frailty, frequency of acute exacerbations, change in symptom related quality of life, improved cardiovascular status, decrease in inflammatory biomarkers, endothelial function and 1 year survival).

2. To explore effects of allo-hMSCs on symptom related quality of life, cardiovascular performance, endothelial function and inflammation.

A.3.2 Study Endpoints

A.3.2.1 Primary Endpoints (Safety)

1. Safety (Primary): Incidence (at one month post second infusion) of any treatment-emergent serious adverse events (TE-SAEs), defined as the composite of: death, non-fatal pulmonary embolism, stroke, hospitalization for worsening dyspnea and clinically significant laboratory test abnormalities, determined per the Investigator's judgment.

   Laboratory tests included in the primary endpoint include the following:

   - Serum chemistry: chloride, sodium, Carbon Dioxide, BUN, creatinine, glucose, calcium, AST/SGOT, ALT/SGPT, total bilirubin (fractionate if total >1.5 times normal), alkaline phosphatase, albumin,
   - Hematology (CBC): hemoglobin, hematocrit, platelets, WBC, WBC differential

2. The incidence of Adverse Events and Serious Adverse Events will be described at 30-days and 6-months post second infusion. All adverse events will be classified into system organ class and preferred term according to the Medical Dictionary for Regulatory Activities.

A.3.2.2 Secondary Endpoints (Efficacy)

The following efficacy endpoints will be evaluated in this trial (during baseline and 6 month follow-up visits):

1. Difference in rate of decline of Frailty defined as:
   - Reduced Activity (assessed via CHAMPS questionnaire)
   - Slowing of Mobility (assessed via a 4 meter gait speed test and SPPB assessment)
   - Weight Loss
   - Diminished handgrip strength (assessed via dynamometer)
   - Exhaustion (assessed via the MFI questionnaire)
   - Decrease in subject quality of life assessment(s)
2. Death from any cause.

3. Change between baseline and 6 months in dobutamine stress echo induced ejection fraction.
4. Change between baseline and 6 months for the following panel of inflammatory markers: CRP, IL-6, D-dimer, fibrinogen, CBC with differential, DNA, and TNFα.
5. The incidence of each component of the primary endpoint including non-fatal pulmonary embolism, stroke, hospitalization for worsening dyspnea and clinically significant laboratory test abnormalities
6. Change in Smell Identification Test (UPSIT)

A.4 Inclusion and Exclusion Criteria

A.4.1 Inclusion Criteria for Follow-on Phase

In order to participate in this study, a subject MUST:

1. Provide written informed consent.
2. Subjects age ≥60 and ≤95 years at the time of signing the Informed Consent Form.
3. Have previously participated in the pilot phase of this trial
4. Female subjects must have an FSH ≥ 25.8 mIU/mL, if not currently on hormone replacement therapy.

A.4.2 Exclusion Criteria for Follow-on Phase

In order to participate in this study, a subject MUST NOT have any of the following:

1. Score of ≤24 on the Mini Mental State Examination (MMSE)
2. Inability to perform any of the assessments required for endpoint analysis (report safety or tolerability concerns, perform PFTs, undergo blood draws, read and respond to questionnaires.
3. Active listing (or expected future listing) for transplant of any organ.

4. Clinically important abnormal screening laboratory values, including but not limited to: hemoglobin <8 g/dl, white blood cell count <3000/mm^3, platelets<80,000/mm^3, INR > 1.5 not due to a reversible cause (i.e. Coumadin), aspartate transaminase, alanine transaminase, or alkaline phosphatase > 3 times upper limit of normal, total bilirubin > 1.5 mg/dl.

5. Serious comorbid illness that, in the opinion of the investigator, may compromise the safety or compliance of the subject or preclude successful completion of the study. Including, but not limited to: HIV, advanced liver or renal failure, class III/IV congestive heart failure, myocardial infarction, unstable angina, or cardiac revascularization within the last six months, or severe obstructive ventilatory defect.

6. Any other condition that, in the opinion of the investigator, may compromise the safety or compliance of the subject or preclude successful completion of the study.
7. Be an organ transplant recipient.
8. Have a clinical history of malignancy within 3 years (i.e., subjects with prior malignancy must be disease free for 3 years), except curatively-treated basal cell carcinoma, squamous cell carcinoma, melanoma in situ or cervical carcinoma if recurrence occurs.

9. Have a non-pulmonary condition that limits lifespan to < 1 year.
10. Have a history of drug or alcohol abuse within the past 24 months.
11. Be serum positive for HIV, hepatitis BsAg or Viremic hepatitis C.
12. Be currently participating (or participated within the previous 30 days) in an investigational therapeutic or device trial.
13. Be a female who is pregnant, nursing, or of childbearing potential while not practicing effective contraceptive methods. Female subjects must undergo a blood or urine pregnancy test at screening and within 36 hours prior to injection.
14. Have hypersensitivity to dimethyl sulfoxide (DMSO)

A.5 Dosing

After subjects complete their Month 12 follow-up phone call visit in the pilot phase, all 15 subjects will then have the option of receiving additional infusions of allogeneic hMSCs: 1×10^8 (100 million) cells delivered via peripheral intravenous infusion.

The Allo-hMSCs will be derived from donors meeting criteria for allogeneic unrelated human bone marrow stem cell source manufactured by the University of Miami.

A.6 Dosage Rationale

A safety profile for IV infusion of hMSCs was based on results from previous completed toxicology results (Hare et al. 2277-86). The results from previous studies demonstrate that the product can be administered intravenously without toxic events at up to 65 x 10^6hMSC/kg dose delivered in one bolus infusion or at 100 x 10^6hMSC/kg cumulative dose delivered by 5 infusions (20 x 10^6hMSC/kg per infusion).

The evidence supports the conclusion that it is feasible to dose subjects in this study based on a standard dose of hMSCs rather than per kilogram of body weight. The total cell number corresponds to a range of 1.3 - 4.4 x 10^6 hMSCs per kg per infusion for subjects with 45 to 150kg body weight, the weight range for this study.

Therefore, results from previous trials support the rationale on the safety and potential efficacy of a second infusion of the selected maximum dose of 100 x10^6 allo-hMSCs.

A.7 Administration Rate

Prior clinical trials have used rates up to 30×10^6 hMSC/min where no infusion related toxicity was observed.

In the proposed study, the cell dose to be delivered is 20x 10^6, and 100×10^6hMSC/infusion, and 200x10^6 reconstituted with the 2.5 million hMSC/ml, in the following total volume

- 40ml for 100 million dose (5 million hMSC/min)

Cell will be delivered at a rate of 2ml/min, and delivered at a maximum rate of 16×10^6 hMSC/minute and will last approximately:

- 40 minutes for 40ml for 100 million dose

A.8 Data and Safety Monitoring Board (DSMB)

DSMB will continue to provide the same oversight as noted in Section 9.4 of the Main protocol.

A.9 Concomitant Treatments, Procedures, and Nondrug Therapies

Refer to section 4.3 of the main study protocol

A.10 Infusion Monitoring Guidelines

Refer to section 6.4.1 and Appendix 1 of the main protocol.

A.11 Adverse Events and Serious Adverse Events

Refer to section 8.9 through 8.15 of the main study protocol.

A.12 Stopping Guidelines

Refer to section 8.2.4 of the main study protocol for further stopping guidelines.

A.13 Payments to Subjects in the Follow-on Phase

Subjects will be reimbursed $25 at the end of each follow-up visit (Month 1 – Month 6 for a total remuneration of $75. These disbursements are meant to cover the time required to complete these study visits and all necessary travel and parking expenses.

Normal donors for generation of allo-MSC will be reimbursed $350 at the end of BM aspiration. This payment will compensate donors for lost time, parking, and travel expenses.

A.14 Study Procedures for Pilot Phase Optional Second Infusion

Table A1: Pilot Phase Optional second infusion Schedule

Visit Schedule for Second Infusion	Month 13 Screening ± 6 Months	Baseline (-4 weeks)	Day 1	Month 1 (Day 30) (+/- 7 days)	Month 3 (Day 90) (+/- 30 days)	Month 6 (Day 180) (+/-30 days)	Month 12 (Day 365) (+/-2 weeks) *Phone Call Follow-up
Informed Consent	x						
Full Medical History	x						
Physical Exam	x	x	x	x	x	x	
12-lead (ECG)	x	x	x	x	x	x	
Concomitant Medications	x	x	x	x	x	x	x
Mini Mental State Examination (MMSE)	x					x	
Infusion Treatment (IP)			x				
Dobutamine Stress Echo Test (DSE)	x					x	
Bone Density Scan (DEXA)[8]		x				x	
FEV-1		x				x	
6 Minute Walk Test		x				x	
4 Meter Gait Speed Test[7]		x				x	
SPPB Assessment		x				x	
Dynamometer (handgrip)		x				x	
Smell Identification Test (UPSIT)		x				x	
IIEF, SQOL-F Questionnaires		x		x	x	x	
QOL Questionnaires (ICECAP, EQ-5D, SF-36,CHAMPS, MFI)		x		x	x	x	
Urinalysis	x			x	x	x	
Hemat., Chem., CBC, LFTs, INR, and other labs[1]	x		x	x	x	x	
HIV 1, HIV 2, Hep. B & C, and CMV	x						
Serum or Urine Pregnancy Test[2]	x		x				
Donor Screening Tests	x						
Review Adverse Events			x	x	x	x	x
Immune Monitoring[4]			x	x	x	x	
Biomarker Assessment[3]			x			x	
Optional: Brachial Ultrasound [5]		x			x		
Optional: Endothelial blood samples[6]		x			x		

Time and Events Table Key:

1 - The minimal laboratory requirements for hematological, liver function and renal function include:

Hematology Tests: white blood cell count, platelet count, hemoglobin and hematocrit.

Liver Function Tests: Albumin, alkaline phosphatase, alanine transaminase, asparate aminotransferase, prothrombin time / activated partial thromboplastin time, and bilirubin(fractionate if total >1.5 times normal).

Renal Function Tests: creatinine, creatinine clearance, blood urea nitrogen (BUN), glomerular filtration rate, sodium, potassium, chloride, calcium, carbon dioxide, and glucose.

Serum Uric Acid, Pro-BNP, and C-reactive protein (CRP), IL6, fibrinogen, D-Dimer, TNFα, testosterone (males only) and estrogen (females only).

- *Laboratory work may be completed at a diagnostic center or home health agency should the subject be unable to come to the site.*

2 - A serum or urine pregnancy test will be completed within 36 hours prior to infusion for females of childbearing potential.

3 - The following biomarkers will be analyzed:

- **Cell-surface markers**: CXCR4, C-Kit, &Connexin 43
- **Transcriptomic/Proteome:** RNA, miRNA, protein samples, and telomerase, akt
- **Growth factors**: Sdf-1, notch,
- **Functional Assays:** cell growth rate, VEGF, and CFU assay

4 - Immune monitoring for graft rejection. The following markers will be used for analysis to assess for activated T-cells based upon a $CD3^+CD25^+$ or $CD3^+CD69^+$ phenotype:

- CD3, CD25, CD69

5 - Optional brachial ultrasound to assess endothelial function.

6 - Optional: An additional 5 lavender top tubes (EDTA) will be drawn.

7 – 4 meter gait speed test will be performed twice per visit and the average of the exams will be taken.

8 – DEXA scan will be performed twice at each visit. The first scan will be of the hip and spine for bone density and the second will be to assess the total body composition.

A.15 Study Visits

A.15.1 Screening Visit for second infusion (Applicable to pilot subjects)

See Table A1 for the procedures and assessments to be performed during the screening visit of the study for the 15 pilot subjects that consent to participate in a second infusion. All screening visit test and procedures will occur upon signing the informed consent form (ICF). No screening exams will take place until the subject is fully informed of the research and signs the consent form. There will be up to a 6 month window from the subjects Month 12 follow-up visit to the subject's additional infusion.

A.15.2 Baseline Visit for second infusion (Applicable to pilot subjects)

See Table A1 for the procedures and assessment to be performed during the baseline visit of the study. Once all screening exams are completed and it has been determined that the subject remains eligible for the study, subjects will be enrolled into the study. The baseline visit will take place within four weeks from treatment. The listed procedures should all be performed as soon as practicable.

Endothelial function (Optional Assessments) will occur upon the subject signing the optional section of the informed consent form (ICF). No endothelial function tests will take place until the subject is fully informed of the research and signs the optional portion of the consent form.

- Brachial ultrasound testing and blood collection will be performed to assess endothelial function in the aging frailty population at baseline and 3 months post stem cell infusion. This will help provide cumulative data in assessing whether or not stem cell infusion improves endothelial function.

- Flow Mediated Diameter percent change (FMD%): All measurements of the brachial artery diameter and FMD will be performed in the morning, in a quiet and dark room and at controlled ambient temperatures between 20°C and 26°C. Studies will be conducted after an overnight fast of at least 10 hours (water is permitted), with the subjects supine and after 10 minutes of rest. The subject's right arm will be comfortably immobilized in an extending position, allowing for ultrasound scanning of the brachial artery 5–10 cm above the antecubital fossa. In each examination, recording of vessel images will be followed by inflation of a cuff to supra-systolic pressure (40 to 50 mmHg above systolic pressure) for 5 minutes. Then the cuff will be deflated and the brachial artery diameter will be imaged and recorded for 3 minutes. FMD% more than 10% is considered a normal response. Lower than 10% FMD% reflects endothelial dysfunction, which means a high likelihood to develop cardiovascular event in the future. Subjects with negative FMD% results (the artery is constricted after stress and not dilated as was expected) have the worst prognosis.

- Blood drawn from fasting subjects will be separated and the serum will be frozen until processed as one batch towards the end of the study. Blood will be processed twice – in the beginning of the study and after 3 months.

- Biochemical analysis: soluble pro inflammatory cytokines (interleukin-1, interleukin-6, interleukin-10, VEGFR2, TNF-a).

- Assay of colony forming units: Fresh blood will be processed for cell culture assays for endothelial progenitor stem cells colonies counting (a 5 days' protocol). Fifty milliliter of blood will be processed; peripheral-blood mononuclear cells will be isolated by Ficoll density-gradient centrifugation, will be washed twice in phosphate buffered saline with 5% fetal bovine serum and re-suspended in media (EndoCult basal media with supplements; StemCell Technologies, Vancouver, British Columbia, Canada) for EPC colony-forming assay. Cells will be planted on human fibronectin-coated plates (BIOCOAT; Becton Dickenson Labware, Bedford, Massachusetts) at a density of $5X10^6$ cells/well and incubated at 37^0C in humidified 5% CO_2. After 48 hours, the non-adherent cells will be re-plated onto fibronectin-coated 24 well plates at a density of $1X10^6$ cells/well. After 5 days, colony forming units (defined as a central core of rounded cells surrounded by elongated and spindle-shaped cells) will be counted manually in 8 wells out of a 24-well plate.

A.15.3 Day 1 Visit for second infusion (Applicable to pilot subjects)

See Table A1 for the procedures and assessment to be performed during the Day 1 visit of the study. The Day 1 visit will occur after all baseline tests are completed and it has been determined that the subject remains eligible. Once the subject is deemed eligible to continue in the study the subject will be administered the investigational product. The subject will be monitored for 2-3 hours, following administration of the investigational product, and will be sent home the same day.

A.15.4 Month 1 Visit for second infusion (Applicable to pilot subjects)

See Table A1 for the procedures and assessment to be performed for the Month 1 visit of the study. Subject visits should be completed as close to the scheduled visit dates as possible. There will be a +/- window of 2 weeks for the month 1 study visit.

A.15.5 Month 3 and Month 6 Visit for second infusion (Applicable to pilot subjects)

See Table A1 for the procedures and assessment to be performed for month 3 and 6 visit of the study. Subject visit should be completed as close to the scheduled visit dates as possible. There will be a +/- window of 30 days for the month 3 and 6 study visit.

A.15.6 Month 12 Visit

See Table A1 for the procedures and assessments to be performed for Month 12 visit. This visit will be conducted via a phone interview with the subject. A phone script will be provided to the study personnel to use when interviewing the subject. There will be a +/- window of 2 weeks for this visit.

A.16 Statistical Considerations

All subjects will be offered the re-infusion and formal statistical considerations regarding sample size and power are not provided. Based on prior studies, a second infusion of stem cells are safe but strict safety monitoring will be employed to continue the safety assessment of allogeneic infusion of MSCs. Detailed analyses will be described in the separate statistical analysis plan.

A.17 Safety Monitoring of 30-Day Rate of TE-SAEs

Monitoring of the rate of TE-SAEs by 30-days post-second infusion among subjects who received the injection of allogeneic MSCs will be employed to assist the DSMB in prospective monitoring of this study. The guideline is to be used to indicate boundaries requiring discussion by the DSMB and is designed to assist the independent DSMB in overseeing the study. The DSMB may also request additional interim analyses and develop other criteria including provision for monitoring of potential late effects to determine when to intervene in the enrollment or treatment of subjects in the study. Monitoring of key safety endpoints will be conducted. If rates significantly exceed the pre-set threshold, then the DSMB will be notified.

A Bayesian motivated safety stopping guideline for monitoring the 30-day TE-SAE rate will be used for this trial. The expected underlying rate of TE-SAE at 30 days post-second infusion is assumed to be 13.3% based on the assumption that 2 out of 15 subjects experience a TE-SAE. It would then be assumed that a rate of greater than 40.0% is unacceptable.

A Beta distribution can be used as the prior distribution of θ; where θ is the proportion of subjects who experience an TE-SAE by 30-days post-second infusion. The stopping rule is based on the beta-binomial methodology and assumes a prior expected failure rate. This leads to prior Beta parameters where a=0.8 and b=5.2. The Beta distribution will have a prior mean of 0.13 and a prior probability of <0.05 of exceeding 0.40. The guideline is derived such that there is strong evidence (posterior probability >0.95) that the probability of the event is greater than 40%, the trial will be stopped. The resulting boundaries tabulated in table 4 were rounded to be conservative with the stopping guideline and is considered after 5 subjects are enrolled on the study.

<u>TABLE A2</u>

Bayesian Stopping Guideline for Event Rate of 13% *

# Events	**# Subjects in Study**
3	5
4	6-9
5	10-14
6	15

* The stopping guidelines serve as a trigger for consultation with the DSMB for additional review, and are not formal “stopping rules” that would mandate automatic closure of study enrollment.

A simulation study was conducted to evaluate the operating characteristics of this stopping rule. Data were generated from the binomial distribution with varying probabilities of failure (θ) and assuming a sample size of 15 subjects. Table 5 shows the probability of stopping the trial early and the average sample size (N), conditional on stopping early, at which the boundary is crossed for each value of θ. The unconditional average sample size of the trials for each value of θ is displayed.

Table A3
Operating Characteristics for Bayesian Motivated Stopping Guideline

Mean of Prior Distribution	θ	Probability of stopping	Conditional Average Sample Size (N)	Unconditional Average Sample Size of Trials (N)
0.13	0.13	0.05	8.4	14.7
	0.18	0.12	8.8	14.2
	0.23	0.25	8.9	13.5
	0.28	0.40	8.8	12.5
	0.33	0.56	8.5	11.4
	0.40	0.75	8.0	9.8

Although the motivation for the boundary is Bayesian, the operating characteristics can be evaluated from a frequentist perspective of Type I error and power. The stopping rule for a 13% event rate has a 5% chance (“Type I error”) of suggesting early termination when the true rate is 0.13, and a 75% chance (“power”) when the true rate is 0.40.

Limitations

A major limitation to the second infusion approach is the small sample size. While it is assumed that all 15 subjects will be eligible for a second infusion, there is a chance that subjects will not want to undergo a second infusion and thus would further limit the amount of information received. However, the second infusion strategy is being implemented to obtain information on the safety of a second infusion of allogeneic MSCs to help guide future studies.

Addendum B: Penicillin/streptomycin free cell safety study

B.1 Rationale and Description for penicillin/streptomycin free cell safety study

This addendum to the protocol is designed to gain additional safety of penicillin/streptomycin free intravenous infusion of allo-hMSCs in subjects with aging frailty. Up to twenty subjects will be recruited to participate in a single dose of allogeneic

MSCs in this follow-on phase. All study specific processes and procedures included in the protocol apply in this addendum unless otherwise noted. This population will not be included in the original 30 subject randomized, double-blinded cohort.

B.2 STUDY OBJECTIVES AND ENDPOINTS

B.2.1 Study Objectives

B.2.1.1 Primary Objective

1. Demonstrate the safety and tolerability of penicillin/streptomycin free intravenous infusion of allo-hMSCs in subjects with aging frailty.

B.2.1.2 Secondary Objectives

2. To explore treatment efficacy (decrease in frailty, frequency of acute exacerbations, change in symptom related quality of life, improved cardiovascular status, decrease in inflammatory biomarkers, endothelial function and 1 year survival).

B.2.2 Study Endpoints

B.2.2.1 Primary Endpoints (Safety)

1. Safety (Primary): Incidence (at one month post second infusion) of any treatment-emergent serious adverse events (TE-SAEs), defined as the composite of: death, non-fatal pulmonary embolism, stroke, hospitalization for worsening dyspnea and clinically significant laboratory test abnormalities, determined per the Investigator's judgment.

2. Laboratory tests included in the primary endpoint include the following:
   - Serum chemistry: chloride, sodium, Carbon Dioxide, BUN, creatinine, glucose, calcium, AST/SGOT, ALT/SGPT, total bilirubin (fractionate if total >1.5 times normal), alkaline phosphatase, albumin,
   - Hematology (CBC): hemoglobin, hematocrit, platelets, WBC, WBC differential

3. The incidence of Adverse Events and Serious Adverse Events will be described at 30-days and 6-months post penicillin/streptomycin free infusion. All adverse events will be classified into system organ class and preferred term according to the Medical Dictionary for Regulatory Activities.

B.2.2.2 Secondary Endpoints (Efficacy)

The following efficacy endpoints will be evaluated in this trial (during baseline and 6 month follow-up visits):

1. Difference in rate of decline of Frailty defined as:
    - Reduced Activity (assessed via CHAMPS questionnaire)
    - Slowing of Mobility (assessed via a 4 meter gait speed test and SPPB assessment)
    - Weight Loss
    - Diminished handgrip strength (assessed via dynamometer)
    - Exhaustion (assessed via the MFI questionnaire)
    - Decrease in subject quality of life assessment(s)
2. Death from any cause.
3. Change between baseline and 6 months in dobutamine stress echo induced ejection fraction.
4. Change between baseline and 6 months for the following panel of inflammatory markers: CRP, IL-6, D-dimer, fibrinogen, CBC with differential, DNA, and TNFα
5. The incidence of each component of the primary endpoint including non-fatal pulmonary embolism, stroke, hospitalization for worsening dyspnea and clinically significant laboratory test abnormalities
6. Change in Smell Identification Test (UPSIT)

B.3 Inclusion and Exclusion Criteria

B.3.1 Inclusion Criteria for penicillin/streptomycin free cell safety study

In order to participate in this study, a subject MUST:

1. Provide written informed consent.
2. Subjects age ≥ 60 and ≤95 years at the time of signing the Informed Consent Form.
3. Show signs of frailty apart from a concomitant condition as assessed by the Investigator with a frailty score of 4 to 7 using the Canadian Clinical Frailty Scale
4. Female subjects must have an FSH ≥ 25.8 mIU/mL, if not currently on hormone replacement therapy.

B.3.2 Exclusion Criteria for penicillin/streptomycin free cell safety study

In order to participate in this study, a subject MUST NOT have any of the following:

1. Score of $\leq$24 on the Mini Mental State Examination (MMSE)

2. Inability to perform any of the assessments required for endpoint analysis (report safety or tolerability concerns, perform PFTs, undergo blood draws, read and respond to questionnaires.

3. Active listing (or expected future listing) for transplant of any organ.

4. Clinically important abnormal screening laboratory values, including but not limited to: hemoglobin <8 g/dl, white blood cell count <3000/mm^3, platelets<80,000/mm^3, INR > 1.5 not due to a reversible cause (i.e. Coumadin), aspartate transaminase, alanine transaminase, or alkaline phosphatase > 3 times upper limit of normal, total bilirubin > 1.5 mg/dl.

4. Serious comorbid illness that, in the opinion of the investigator, may compromise the safety or compliance of the subject or preclude successful completion of the study. Including, but not limited to: HIV, advanced liver or renal failure, class III/IV congestive heart failure, myocardial infarction, unstable angina, or cardiac revascularization within the last six months, or severe obstructive ventilatory defect.

    - Any other condition that, in the opinion of the investigator, may compromise the safety or compliance of the subject or preclude successful completion of the study.
    - Be an organ transplant recipient.
    - Have a clinical history of malignancy within 3 years (i.e., subjects with prior malignancy must be disease free for 3 years), except curatively-treated basal cell carcinoma, squamous cell carcinoma, melanoma in situ or cervical carcinoma if recurrence occurs.
    - Have a non-pulmonary condition that limits lifespan to < 1 year.
    - Have a history of drug or alcohol abuse within the past 24 months.
    - Be serum positive for HIV, hepatitis BsAg or Viremic hepatitis C.
    - Be currently participating (or participated within the previous 30 days) in an investigational therapeutic or device trial.
    - Be a female who is pregnant, nursing, or of childbearing potential while not practicing effective contraceptive methods. Female subjects must undergo a blood or urine pregnancy test at screening and within 36 hours prior to injection.
    - Have hypersensitivity to dimethyl sulfoxide (DMSO)

B.4 Dosing

After subjects complete have completed their screening and continue to meet inclusion/exclusion criteria subjects will receive a single infusion of allogeneic penicillin/streptomycin free hMSCs: 1 x 10^8 (100 million) cells delivered via peripheral intravenous infusion.

The penicillin/streptomycin free Allo-hMSCs will be derived from donors meeting criteria for allogeneic unrelated human bone marrow stem cell source manufactured by the University of Miami.

B.5 Dosage Rationale

A safety profile for IV infusion of hMSCs was based on results from previous completed toxicology results[Hare et al. 2277-86]. The results from previous studies demonstrate that the product can be administered intravenously without toxic events at up to 65 x 10^6hMSC/kg dose delivered in one bolus infusion or at 100 x 10^6hMSC/kg cumulative dose delivered by 5 infusions (20 x 10^6hMSC/kg per infusion).

The evidence supports the conclusion that it is feasible to dose subjects in this study based on a standard dose of hMSCs rather than per kilogram of body weight. The total cell number corresponds to a range of 1.3 - 4.4 x 10^6 hMSCs per kg per infusion for subjects with 45 to 150kg body weight, the weight range for this study.

Therefore, results from previous trials support the rationale on the safety and potential efficacy of an infusion of the selected maximum dose of 100 x10^6 allo-hMSCs.

B.6 Administration Rate

Prior clinical trials have used rates up to 30×10^6 hMSC/min where no infusion related toxicity was observed.

In the proposed study, the cell dose to be delivered is 20x 10^6, and 100×10^6hMSC/infusion, and 200x10^6 reconstituted with the 2.5 million hMSC/ml, in the following total volume

- 40ml for 100 million dose (5 million hMSC/min)

Cell will be delivered at a rate of 2ml/min, and delivered at a maximum rate of 16×10^6 hMSC/minute and will last approximately:

- 40 minutes for 40ml for 100 million dose

B.7 Data and Safety Monitoring Board (DSMB)

DSMB will continue to provide the same oversight as noted in Section 9.4 of the Main protocol.

B.8 Concomitant Treatments, Procedures, and Nondrug Therapies

Refer to section 4.3 of the main study protocol

B.9 Infusion Monitoring Guidelines

Refer to section 6.4.1 and Appendix 1 of the main protocol.

B.10 Adverse Events and Serious Adverse Events

Refer to section 8.9 through 8.15 of the main study protocol.

B.11 Stopping Guidelines

Refer to section 8.2.4 of the main study protocol for further stopping guidelines.

B.12 Payments to Subjects in the Follow-on Phase

Subjects will be reimbursed $25 at the end of each follow-up visit (Month 1 – Month 6) for a total remuneration of $75. These disbursements are meant to cover the time required to complete these study visits and all necessary travel and parking expenses.

Normal donors for generation of allo-MSC will be reimbursed $350 at the end of BM aspiration. This payment will compensate donors for lost time, parking, and travel expenses.

B.13 Study Procedures for Penicillin/Streptomycin Free hMSCs Infusion

Table B1: Time and Events Schedule

Visit Schedule	Screening + 45 days	Baseline (0 to -4 weeks)	Day 1	Month 1 (Day 30) (+/- 7 days)	Month 3 (Day 90) (+/- 30 days)	Month 6 (Day 180) (+/-30 days)	Month 12 (Day 365) (+/-2 weeks) *Phone Call Follow-up
Informed Consent	x						
Full Medical History	x						
Physical Exam	x	x	x	x	x	x	
12-lead (ECG)	x	x	x	x	x	x	
Concomitant Medications	x	x	x	x	x	x	x
Mini Mental State Examination (MMSE)	x					x	
Infusion Treatment (IP)			x				
Dobutamine Stress Echo Test (DSE)	x					x	
Bone Density Scan (DEXA)[8]		x				x	
FEV-1		x				x	
6 Minute Walk Test		x				x	
4 Meter Gait Speed Test[7]		x				x	
SPPB Assessment		x				x	
Dynamometer (handgrip)		x				x	
Smell Identification Test (UPSIT)		x				x	
IIEF, SQOL-F Questionnaires		x		x	x	x	
QOL Questionnaires (ICECAP, EQ-5D, SF-36,CHAMPS, MFI)		x		x	x	x	
Urinalysis	x			x	x	x	
Hemat., Chem., CBC, LFTs, INR, and other labs[1]	x		x	x	x	x	
HIV 1, HIV 2, Hep. B & C, and CMV	x						
Serum or Urine Pregnancy Test[2]	x		x				
Donor Screening Tests	x						
Review Adverse Events			x	x	x	x	x
Immune Monitoring[4]			x	x	x	x	
Biomarker Assessment[3]			x			x	
Optional: Brachial Ultrasound [5]		x			x		
Optional: Endothelial blood samples[6]		x			x		

Time and Events Table Key:

1 - The minimal laboratory requirements for hematological, liver function and renal function include:

Hematology Tests: white blood cell count, platelet count, hemoglobin and hematocrit.

Liver Function Tests: Albumin, alkaline phosphatase, alanine transaminase, asparate aminotransferase, prothrombin time / activated partial thromboplastin time, and bilirubin(fractionate if total >1.5 times normal).

Renal Function Tests: creatinine, creatinine clearance, blood urea nitrogen (BUN), glomerular filtration rate, sodium, potassium, chloride, calcium, carbon dioxide, and glucose.

Serum Uric Acid, Pro-BNP, and C-reactive protein (CRP), IL6, fibrinogen, D-Dimer, TNFα, testosterone (males only) and estrogen (females only).

- *Laboratory work may be completed at a diagnostic center or home health agency should the subject be unable to come to the site.*

2 - A serum or urine pregnancy test will be completed within 36 hours prior to infusion for females of childbearing potential.

3 - The following biomarkers will be analyzed:

- **Cell-surface markers**: CXCR4, C-Kit, &Connexin 43
- **Transcriptomic/Proteome:** RNA, miRNA, protein samples, and telomerase, akt
- **Growth factors**: Sdf-1, notch,
- **Functional Assays:** cell growth rate, VEGF, and CFU assay

4 - Immune monitoring for graft rejection. The following markers will be used for analysis to assess for activated T-cells based upon a $CD3^+CD25^+$ or $CD3^+CD69^+$ phenotype:

- CD3, CD25, CD69

5 - Optional brachial ultrasound to assess endothelial function.

6 - Optional: An additional 5 lavender top tubes (EDTA) will be drawn.

7 – 4 meter gait speed test will be performed twice per visit and the average of the exams will be taken.

8 – DEXA scan will be performed twice at each visit. The first scan will be of the hip and spine for bone density and the second will be to assess the total body composition.

B.14 Study Visits

B.14.1 Screening Visit

See Table B1 for the procedures and assessments to be performed during the screening visit. Screening visit test and procedures will occur upon signing the informed consent form (ICF). No screening exams will take place until the subject is fully informed of the research and signs the consent form. There will be up to a 45 day window from the time the subject signs the informed consent form to the baseline visit to complete screening procedures.

B.14.2 Baseline Visit

See Table B1 for the procedures and assessment to be performed during the baseline visit of the study. Once all screening exams are completed and it has been determined that the subject remains eligible for the study, subjects will be enrolled into the study. The baseline visit will take place within four weeks from treatment. The listed procedures should all be performed as soon as practicable.

Endothelial function (Optional Assessments) will occur upon the subject signing the optional section of the informed consent form (ICF). No endothelial function tests will take place until the subject is fully informed of the research and signs the optional portion of the consent form.

- Brachial ultrasound testing and blood collection will be performed to assess endothelial function in the aging frailty population at baseline and 3 months post stem cell infusion. This will help provide cumulative data in assessing whether or not stem cell infusion improves endothelial function.

- Flow Mediated Diameter percent change (FMD%): All measurements of the brachial artery diameter and FMD will be performed in the morning, in a quiet and dark room and at controlled ambient temperatures between 20°C and 26°C. Studies will be conducted after an overnight fast of at least 10 hours (water is permitted), with the subjects supine and after 10 minutes of rest. The subject's right arm will be comfortably immobilized in an extending position, allowing for ultrasound scanning of the brachial artery 5–10 cm above the antecubital fossa. In each examination, recording of vessel images will be followed by inflation of a cuff to supra-systolic pressure (40 to 50 mmHg above systolic pressure) for 5 minutes. Then the cuff will be deflated and the brachial artery diameter will be imaged and recorded for 3 minutes. FMD% more than 10% is considered a normal response. Lower than 10% FMD% reflects endothelial dysfunction, which means a high likelihood to develop cardiovascular event in the future. Subjects with negative FMD% results (the artery is constricted after stress and not dilated as was expected) have the worst prognosis.

  Blood drawn from fasting subjects will be separated and the serum will be frozen until processed as one batch towards the end of the study. Blood will be processed twice – in the beginning of the study and after 3 months.

- Biochemical analysis: soluble pro inflammatory cytokines (interleukin 1, interleukin-1 receptor antagonist, interleukin-2, interleukin-6, tumor necrosis factor alpha, high-sensitivity C Reactive Protein, Pro-Brain Natriuretic Peptide, von-Willebrand Factor) and soluble cell adhesion molecules (E-selectin, P-selectin, Inter Cellular Adhesion Molecule 1, Vascular Cell Adhesion Molecule 1), and apoptotic factors (Anexin 5) using immunological and ELISA methods.

- Assay of colony forming units: Fresh blood will be processed for cell culture assays for endothelial progenitor stem cells colonies counting (a 5 days' protocol). Fifty milliliter of blood will be processed; peripheral-blood mononuclear cells will be isolated by Ficoll density-gradient centrifugation, will be washed twice in phosphate buffered saline with 5% fetal bovine serum and re-suspended in media (EndoCult basal media with supplements; StemCell Technologies, Vancouver, British Columbia, Canada) for EPC colony-forming assay. Cells will be planted on human fibronectin-coated plates (BIOCOAT; Becton Dickenson Labware, Bedford, Massachusetts) at a density of $5X10^6$ cells/well and incubated at 37^0C in humidified 5% CO_2. After 48 hours, the non-adherent cells will be re-plated onto fibronectin-coated 24 well plates at a density of $1X10^6$ cells/well. After 5 days, colony forming units (defined as a central core of rounded cells surrounded by elongated and spindle-shaped cells) will be counted manually in 8 wells out of a 24-well plate.
- Flow Cytometry: characterization of EPCs from peripheral blood will be done by Flow Cytometry (FACS). EPCs will be defined as CD34-, Cd19-, CD3-, CD133+/KDR+ cells.

B.14.3 Day 1 Visit

See Table B1 for the procedures and assessment to be performed during the Day 1 visit of the study. The Day 1 visit will occur after all baseline tests are completed and it has been determined that the subject remains eligible. Once the subject is deemed eligible to continue in the study the subject will be administered the investigational product. The subject will be monitored for 2 – 3 hours, following administration of the investigational product, and will be sent home the same day.

B.14.4 Month 1 Visit

See Table B1 for the procedures and assessment to be performed for the Month 1 visit of the study. Subject visits should be completed as close to the scheduled visit dates as possible. There will be a +/- window of 2 weeks for the month 1 study visit.

B.14.5 Month 3 and Month 6 Visit

See Table B1 for the procedures and assessment to be performed for month 3 and 6 visit of the study. Subject visit should be completed as close to the scheduled visit dates as possible. There will be a +/- window of 30 days for the month 3 and 6 study visit.

B.14.6 Month 12 Visit

See Table B1 for the procedures and assessments to be performed for Month 12 visit. This visit will be conducted via a phone interview with the subject. A phone script will be

provided to the study personnel to use when interviewing the subject. There will be a +/- window of 2 weeks for this visit.

B.15 Statistical Considerations

All subjects will be offered the penicillin/streptomycin free hMSCs and formal statistical considerations regarding sample size and power are not provided. Based on prior studies, stem cells are safe but strict safety monitoring will be employed to continue the safety assessment of penicillin/streptomycin free allogeneic infusion of MSCs. No detailed analyses will be described in the statistical analysis plan.

B.16 Safety Monitoring of 30-Day Rate of TE-SAEs

Monitoring of the rate of TE-SAEs by 30-days post infusion among subjects who received the injection of penicillin/streptomycin free allogeneic MSCs will be employed to assist the DSMB in prospective monitoring of this study. The guideline is to be used to indicate boundaries requiring discussion by the DSMB and is designed to assist the independent DSMB in overseeing the study. The DSMB may also request additional interim analyses and develop other criteria including provision for monitoring of potential late effects to determine when to intervene in the enrollment or treatment of subjects in the study. Monitoring of key safety endpoints will be conducted. If rates significantly exceed the pre-set threshold, then the DSMB will be notified.

A Bayesian motivated safety stopping guideline for monitoring the 30-day TE-SAE rate will be used for this trial. The expected underlying rate of TE-SAE at 30 days post-infusion is assumed to be 13.3% based on the assumption that 2 out of 15 subjects experience a TE-SAE. It would then be assumed that a rate of greater than 40.0% is unacceptable.

A Beta distribution can be used as the prior distribution of θ; where θ is the proportion of subjects who experience an TE-SAE by 30-days post infusion. The stopping rule is based on the beta-binomial methodology and assumes a prior expected failure rate. This leads to prior Beta parameters where a=0.8 and b=5.2. The Beta distribution will have a prior mean of 0.13 and a prior probability of <0.05 of exceeding 0.40. The guideline is derived such that there is strong evidence (posterior probability >0.95) that the probability of the event is greater than 40%, the trial will be stopped. The resulting boundaries tabulated in table B2 were rounded to be conservative with the stopping guideline and is considered after 5 subjects are enrolled on the study.

TABLE B2

Bayesian Stopping Guideline for Event Rate of 13% *

# Events	**# Subjects in Study**
3	5
4	6-9
5	10-14
6	15

* The stopping guidelines serve as a trigger for consultation with the DSMB for additional review, and are not formal "stopping rules" that would mandate automatic closure of study enrollment.

A simulation study was conducted to evaluate the operating characteristics of this stopping rule. Data were generated from the binomial distribution with varying probabilities of failure (θ) and assuming a sample size of 15 subjects. Table B3 shows the probability of stopping the trial early and the average sample size (N), conditional on stopping early, at which the boundary is crossed for each value of θ. The unconditional average sample size of the trials for each value of θ is displayed.

<u>Table B3</u>
Operating Characteristics for Bayesian Motivated Stopping Guideline

Mean of Prior Distribution	θ	Probability of stopping	Conditional Average Sample Size (N)	Unconditional Average Sample Size of Trials (N)
0.13	0.13	0.05	8.4	14.7
	0.18	0.12	8.8	14.2
	0.23	0.25	8.9	13.5
	0.28	0.40	8.8	12.5
	0.33	0.56	8.5	11.4
	0.40	0.75	8.0	9.8

Although the motivation for the boundary is Bayesian, the operating characteristics can be evaluated from a frequentist perspective of Type I error and power. The stopping rule for a 13% event rate has a 5% chance ("Type I error") of suggesting early termination when the true rate is 0.13, and a 75% chance ("power") when the true rate is 0.40.

Limitations

A major limitation to this approach is the small sample size. However, the penicillin streptomycin free infusion strategy is being implemented to obtain information on the safety of a penicillin/streptomycin free allogeneic MSCs to help guide future studies.

Addendum C: Pilot and Penicillin/Streptomycin Free Subjects Optional Follow-on Phase

C.1 Rationale and Description of additional infusions for subjects who have received one or two infusions in the phase I open-label phases of this protocol.

This addendum protocol is designed to test the safety of sequential infusions of allogeneic mesenchymal stem cells 12 to 18 months following previous infusion. Subjects in this protocol have previously participated in protocols receiving open label infusions (phase I subjects). There is an important need to assess the safety and immunologic tolerability of additional doses of intravenous allogeneic MSCs in the target population. Previously a group of 12 subjects has received two doses delivered 12-18 month apart with excellent safety and no adverse effects attributed to the study agent. A second group of 20 subjects received a single dose of MSCs, in the penicillin/streptomycin free cohort. Subjects receiving either one or two sequential doses have not mounted significant immunologic reactions to the study agent.

Subjects in the run-in phase received one of 3 doses of allogeneic MSCs and are eligible to participate in this optional follow-on phase. Additionally, subjects receiving one dose (100 Million cells) in the penicillin/streptomycin free protocol are eligible. Because several studies, reviewed below, have now shown safety of repeat doses of MSCs in a variety of medical conditions, additional safety testing in the subject population of aging frailty enrolled in the CRATUS trial is required[92]. All study specific processes and procedures included in the pilot phase and penicillin free follow-on phase of the protocol apply in this addendum unless otherwise noted.

Subjects participating in this protocol will receive a total of four infusions of MSCs (100 Million cells) administered 12-18 months apart. The MSCs provided to all study subjects in this addendum will be penicillin/streptomycin free. Altogether, subjects will have received 4 doses over a time span ranging approximately 4 to 6 years. Subjects will have ongoing safety monitoring that will include detailed immunologic assessments.

C.2 Demonstrated Safety and Increased Efficacy of Multiple Infusions of Allogeneic Mesenchymal Stem Cells

Active clinical trials and ongoing preclinical work provide an accumulating data set supporting the safety and, in some cases, therapeutic efficacy of allogeneic mesenchymal stem cells (MSCs). Allogeneic mesenchymal stem cells are both immunoprivileged and immunosuppressive, thereby enabling their use as an allograft[93]. Cellular therapy with allogeneic MSCs has abundant support from both animal studies and human clinical trials for a wide range of disorders (Table 1). For example, the POSEIDON study addressed the major issue of the use of allogeneic MSCs as a cell-based therapeutic[18]. In this study, subjects with cardiac failure were randomized to receive either autologous MSCs or identically prepared allogeneic MSCs from healthy donors.

Accumulating evidence also supports the concept that repeated doses and/or co-administration of allogeneic MSCs could further enhance therapeutic outcomes. This is an extremely important area for medical investigation, since repeat dosing could potentially have an additive effect and/or reverse disease pathology, depending on the disorder.

Recently, several clinical studies have demonstrated that multiple infusions of allogeneic MSCs are well tolerated with minimal side-effects. Importantly, repeat dosing has no increased level of side effects compared with single doses. For example, results from Franco Locatelli's group[92] clearly demonstrate the safety and efficacy of multiple allogeneic MSC infusions in children with steroid-refractory acute graft versus host disease (aGvHD). Doses in these studies were 1-2x10^6 MSCs/kg recipient body mass, and each child received on average 2 doses (range was 1-13 doses) separated on average by 15 days (range 3-43 days). The results of this study indicated increased effectiveness when the therapy was commenced early in the disease. Furthermore, the study indicated the therapeutic benefits of repeat doses to subjects who did not achieve complete remission after a single dose.

Koc et al[94]; further investigated Hurler syndrome (mucopolysaccharidosis type-IH) and metachromatic leukodystrophy (MLD) and studied allogeneic MSCs based on their potential to differentiate into cells of bone, cartilage, tendon, muscle and other adventitial tissues and offer potential for corrective cellular therapy[95]. Their results demonstrated no infusion-related toxicity, improvement in nerve conduction, and bone mineral density was either maintained or improved in all subjects.

A Phase II study on the 3-year efficacy of allogeneic MSCs in the treatment of system lupus erythrematosus (SLE) was reported by Lingyun Sun's group[96]. The results of this study demonstrated the therapeutic efficacy of allogeneic MSCs in treating SLE. However, no advantage was found by administering a second or higher dose of allogeneic MSCs. Despite this lack of additional efficacy over a single dose, the results of this study confirmed the safety of multiple infusions of allogeneic MSCs. The doses (1x10^6 MSCs/kg body mass) were administered 1 week apart. Another study reported on the use of repeated dosing of allogeneic MSCs in subjects with chronic obstructive pulmonary disease. The study showed minimal therapeutic efficacy after 2 years[19]. Importantly, in this subject population repeated allogeneic MSC infusions, which were given 4 times at 30 day intervals (1x10^8 MSCs/infusion) were safe.

In the pilot phase of this trial, subjects (N=12) have been administered two doses of MSCs 12 to 18 months apart with no adverse effects.

Taken together, these various clinical studies provide a solid rationale for repeat allogeneic MSC infusions. In no case did the administration of multiple doses lead to a significant increase in the frequency of adverse effects, or to a decrease in therapeutic efficacy over a single dose. In many cases, repeated infusions indeed improved clinical outcomes, demonstrating an additive effect of allogeneic MSC therapy. Given these promising results, investigations are now warranted to determine optimal dosing frequency and total doses of allogeneic MSCs to administer.

It is particularly important to establish safety of repeat doses of allogeneic MSCs in the subject population enrolled in the CRATUS study. These individuals are of older age and are not represented in the previous studies described. In this follow-on study participants in earlier open label run-in study and penicillin/streptomycin free follow-on study will be asked to participate in this repeat dosing protocol. Subjects in the pilot-phase or penicillin/streptomycin free phase, which received either one or two infusions previously will be eligible, and all subjects will get a total of four infusions. Each infusion will be 12 to 18 months apart.

C.3 STUDY OBJECTIVES AND ENDPOINTS

C.3.1 Study Objectives

C.3.1.1 Primary Objective

1. Demonstrate the safety and tolerability of up to four intravenous infusions of allo-hMSCs in subjects with aging frailty who had previously received an infusion of allogeneic MSCs as part of an open label phase (phase I) of the protocol.

2. To determine immunologic safety of sequential infusions of allo-hMSCs in subjects with aging frailty.

C.3.1.2 Secondary Objectives

1. To explore treatment efficacy (decrease in frailty, frequency of acute exacerbations, change in symptom related quality of life, improved cardiovascular status, decrease in inflammatory biomarkers, endothelial function and 1 year survival).

C.3.2 Study Endpoints

C.3.2.1 Primary Endpoints (Safety)

1. Safety (Primary): Incidence (at one month post second infusion) of any treatment-emergent serious adverse events (TE-SAEs), defined as the composite of: death, non-fatal pulmonary embolism, stroke, hospitalization for worsening dyspnea and clinically significant laboratory test abnormalities, determined per the Investigator’s judgment.

   Laboratory tests included in the primary endpoint include the following:

   - Serum chemistry: chloride, sodium, Carbon Dioxide, BUN, creatinine, glucose, calcium, AST/SGOT, ALT/SGPT, total bilirubin (fractionate if total >1.5 times normal), alkaline phosphatase, albumin,
   - Hematology (CBC): hemoglobin, hematocrit, platelets, WBC, WBC differential

2. The incidence of Adverse Events and Serious Adverse Events will be described at 30-days and 6-months post second infusion. All adverse events will be classified into system organ class and preferred term according to the Medical Dictionary for Regulatory Activities.

C.3.2.2 Secondary Endpoints (Efficacy)

The following efficacy endpoints will be evaluated in this trial (during baseline and 6 month follow-up visits):

1. Difference in rate of decline of Frailty defined as:
   - Reduced Activity (assessed via CHAMPS questionnaire)
   - Slowing of Mobility (assessed via a 4 meter gait speed test and SPPB assessment)
   - Weight Loss
   - Diminished handgrip strength (assessed via dynamometer)
   - Exhaustion (assessed via the MFI questionnaire)
   - Difference in subject quality of life assessment(s)
2. Death from any cause.
3. Change between baseline and 6 months in dobutamine stress echo induced ejection fraction.
4. Change between baseline and 6 months for the following panel of inflammatory markers: CRP, IL-6, D-dimer, fibrinogen, CBC with differential, and TNFα
5. The incidence of each component of the primary endpoint including non-fatal pulmonary embolism, stroke, hospitalization for worsening dyspnea and clinically significant laboratory test abnormalities
6. Change in Smell Identification Test (UPSIT)

C.4 Inclusion and Exclusion Criteria

C.4.1 Inclusion Criteria for Follow-on Phase

In order to participate in this study, a subject MUST:

1. Provide written informed consent.
2. Subjects age ≥60 and ≤95 years at the time of signing the Informed Consent Form.
3. Have previously participated in the pilot phase of the trial, or have previously participated in the penicillin free (open label) phase of the trial.

4. Female subjects must have an FSH $\geq$ 25.8 mIU/mL, if not currently on hormone replacement therapy.

C.4.2 Exclusion Criteria for Follow-on Phase

In order to participate in this study, a subject MUST NOT have any of the following:

1. Score of $\leq$24 on the Mini Mental State Examination (MMSE)

2. Inability to perform any of the assessments required for endpoint analysis (report safety or tolerability concerns, perform PFTs, undergo blood draws, read and respond to questionnaires.

3. Active listing (or expected future listing) for transplant of any organ.

4. Clinically important abnormal screening laboratory values, including but not limited to: hemoglobin <8 g/dl, white blood cell count <3000/mm^3, platelets<80,000/mm^3, INR > 1.5 not due to a reversible cause (i.e. Coumadin), aspartate transaminase, alanine transaminase, or alkaline phosphatase > 3 times upper limit of normal, total bilirubin > 1.5 mg/dl.

5. Serious comorbid illness that, in the opinion of the investigator, may compromise the safety or compliance of the subject or preclude successful completion of the study. Including, but not limited to: HIV, advanced liver or renal failure, class III/IV congestive heart failure, myocardial infarction, unstable angina, or cardiac revascularization within the last six months, or severe obstructive ventilatory defect.

6. Any other condition that, in the opinion of the investigator, may compromise the safety or compliance of the subject or preclude successful completion of the study.

7. Be an organ transplant recipient.

8. Have a clinical history of malignancy within 3 years (i.e., subjects with prior malignancy must be disease free for 3 years), except curatively-treated basal cell carcinoma, squamous cell carcinoma, melanoma in situ or cervical carcinoma if recurrence occurs.

9. Have a non-pulmonary condition that limits lifespan to < 1 year.

10. Have a history of drug or alcohol abuse within the past 24 months.

11. Be serum positive for HIV, hepatitis BsAg or Viremic hepatitis C.
12. Be currently participating (or participated within the previous 30 days) in an investigational therapeutic or device trial.
13. Be a female who is pregnant, nursing, or of childbearing potential while not practicing effective contraceptive methods. Female subjects must undergo a blood or urine pregnancy test at screening and within 36 hours prior to injection.
14. Have hypersensitivity to dimethyl sulfoxide (DMSO)

C.5 Dosing

Subjects participating in this protocol will have received either one or two infusions of study agent. After subjects complete their Month 12 follow-up phone call visit after their last infusion, all subjects will then have the option of receiving an additional infusion of allogeneic hMSCs: 1 x 10^8 (100 million) cells delivered via peripheral intravenous infusion. Subjects will be eligible to receive a total of four (n=4) infusions, 12 to 18 months apart.

The penicillin/streptomycin free Allo-hMSCs will be derived from donors meeting criteria for allogeneic unrelated human bone marrow stem cell source manufactured by the University of Miami.

C.6 Dosage Rationale

A safety profile for IV infusion of hMSCs was based on results from previous completed toxicology results(Hare et al. 2277-86). The results from previous studies demonstrate that the product can be administered intravenously without toxic events at up to 65 x 10^6hMSC/kg dose delivered in one bolus infusion or at 100 x 10^6hMSC/kg cumulative dose delivered by 5 infusions (20 x 10^6hMSC/kg per infusion).

The evidence supports the conclusion that it is feasible to dose subjects in this study based on a standard dose of hMSCs rather than per kilogram of body weight. The total cell number corresponds to a range of 1.3 - 4.4 x 10^6 hMSCs per kg per infusion for subjects with 45 to 150kg body weight, the weight range for this study.

Therefore, results from previous trials support the ratio nale on the safety and potential efficacy of a second infusion of the selected maximum dose of 100 x10^6 allo-hMSCs.

C.7 Administration Rate

Prior clinical trials have used rates up to 30×10^6 hMSC/min where no infusion related toxicity was observed.

In the proposed study, the cell dose to be delivered is 20x 10^6, and 100×10^6hMSC/infusion, and 200x10^6 reconstituted with the 2.5 million hMSC/ml, in the following total volume

- 40ml for 100 million dose (5 million hMSC/min)

Cell will be delivered at a rate of 2ml/min, and delivered at a maximum rate of 16×10^6 hMSC/minute and will last approximately:

- 40 minutes for 40ml for 100 million dose

C.8 Data Safety Monitoring Board (DSMB)

DSMB will continue to provide the same oversight as noted in Section 9.4 of the Main protocol.

C.9 Concomitant Treatments, Procedures, and Nondrug Therapies

Refer to section 4.3 of the main study protocol

C.10 Infusion Monitoring Guidelines

Refer to section 6.4.1 and Appendix 1 of the main protocol.

C.11 Adverse Events and Serious Adverse Events

Refer to section 8.9 through 8.15 of the main study protocol.

C.12 Stopping Guidelines

Refer to section 8.2.4 of the main study protocol for further stopping guidelines.

C.13 Payments to Subjects

Subjects will be reimbursed $25 at the end of each follow-up visit (Month 1 – Month 6 for a total remuneration of $75. These disbursements are meant to cover the time required to complete these study visits and all necessary travel and parking expenses.

Normal donors for generation of allo-MSC will be reimbursed $350 at the end of BM aspiration. This payment will compensate donors for lost time, parking, and travel expenses.

C.14 Study Procedures for Optional Additional Infusions (See Table C1)

Table C1: Pilot Phase Optional additional infusion(s) Schedule

Visit Schedule for Additional Infusions (repeating for up to 4 doses every 12-18 months)	Month 13 Screening ± 6 Months	Baseline (-4 weeks)	Day 1	Month 1 (Day 30) (+/- 7 days)	Month 3 (Day 90) (+/- 30 days)	Month 6 (Day 180) (+/-30 days)	Month 12 (Day 365) (+/- 6 months) *Phone Call Follow-up
Informed Consent	x						
Full Medical History	x						
Physical Exam	x	x	x	x	x	x	
12-lead (ECG)	x	x	x	x	x	x	
Concomitant Medications	x	x	x	x	x	x	x
Mini Mental State Examination (MMSE)	x					x	
Infusion Treatment (IP)			x				
Dobutamine Stress Echo Test (DSE)	x					x	
Bone Density Scan (DEXA)[8]		x				x	
FEV-1		x				x	
6 Minute Walk Test		x				x	
4 Meter Gait Speed Test[7]		x				x	
SPPB Assessment		x				x	
Dynamometer (handgrip)		x				x	
Smell Identification Test (UPSIT)		x				x	
IIEF, SQOL-F Questionnaires		x		x	x	x	
QOL Questionnaires (ICECAP, EQ-5D, SF-36,CHAMPS, MFI)		x		x	x	x	
Urinalysis	x			x	x	x	
Hemat., Chem., CBC, LFTs, INR, and other labs[1]	x		x	x	x	x	
HIV 1, HIV 2, Hep. B & C, and CMV	x						
Serum or Urine Pregnancy Test[2]	x		x				
Donor Screening Tests	x						
Review Adverse Events			x	x	x	x	x
Immune Monitoring[4]			x	x	x	x	
Biomarker Assessment[3]			x			x	
Optional: Brachial Ultrasound [5]		x			x		
Optional: Endothelial blood samples[6]		x			x		

CONFIDENTIAL AND PROPRIETARY

Time and Events Table Key:

1 - The minimal laboratory requirements for hematological, liver function and renal function include:

Hematology Tests: white blood cell count, platelet count, hemoglobin and hematocrit.

Liver Function Tests: Albumin, alkaline phosphatase, alanine transaminase, asparate aminotransferase, prothrombin time / activated partial thromboplastin time, and bilirubin (fractionate if total >1.5 times normal).

Renal Function Tests: creatinine, creatinine clearance, blood urea nitrogen (BUN), glomerular filtration rate, sodium, potassium, chloride, calcium, carbon dioxide, and glucose.

Serum Uric Acid, Pro-BNP, and C-reactive protein (CRP), IL6, fibrinogen, D-Dimer, TNFα, testosterone (males only) and estrogen (females only).

- *Laboratory work may be completed at a diagnostic center or home health agency should the subject be unable to come to the site.*

2 - A serum or urine pregnancy test will be completed within 36 hours prior to infusion for females of childbearing potential.

3 - The following biomarkers will be analyzed:

- Telomere Length
- DNA
- RNA

4 - Immune monitoring for graft rejection. The following markers will be used for analysis to assess for activated T-cells based upon a $CD3^+CD25^+$ or $CD3^+CD69^+$ phenotype:

- CD3, CD25, CD69

5 - Optional brachial ultrasound to assess endothelial function.

6 - Optional: An additional 5 lavender top tubes (EDTA) will be drawn.

7 – 4 meter gait speed test will be performed twice per visit and the average of the exams will be taken.

8 – DEXA scan will be performed twice at each visit. The first scan will be of the hip and spine for bone density and the second will be to assess the total body composition.

C.15 Study Visits

C.15.1 Screening Visit for additional infusion

See Table C1 for the procedures and assessments to be performed during the screening visit of the study for the subjects that consent to participate in the additional infusions. All screening visit test and procedures will occur upon signing the informed consent form (ICF). No screening exams will take place until the subject is fully informed of the research and signs the consent form. There will be up to a 6 month window from the subjects Month 12 follow-up visit from the subject's initial infusion.

C.15.2 Baseline Visit for additional infusion

See Table C1 for the procedures and assessment to be performed during the baseline visit of the study. Once all screening exams are completed and it has been determined that the subject remains eligible for the study, subjects will be enrolled into the study. The baseline visit will take place within four weeks from treatment. The listed procedures should all be performed as soon as practicable.

Endothelial function (Optional Assessments) will occur upon the subject signing the optional section of the informed consent form (ICF). No endothelial function tests will take place until the subject is fully informed of the research and signs the optional portion of the consent form.

- Brachial ultrasound testing and blood collection will be performed to assess endothelial function in the aging frailty population at baseline and 3 months post stem cell infusion. This will help provide cumulative data in assessing whether or not stem cell infusion improves endothelial function.

- Flow Mediated Diameter percent change (FMD%): All measurements of the brachial artery diameter and FMD will be performed in the morning, in a quiet and dark room and at controlled ambient temperatures between 20°C and 26°C. Studies will be conducted after an overnight fast of at least 10 hours (water is permitted), with the subjects supine and after 10 minutes of rest. The subject's right arm will be comfortably immobilized in an extending position, allowing for ultrasound scanning of the brachial artery 5–10 cm above the antecubital fossa. In each examination, recording of vessel images will be followed by inflation of a cuff to supra-systolic pressure (40 to 50 mmHg above systolic pressure) for 5 minutes. Then the cuff will be deflated and the brachial artery diameter will be imaged and recorded for 3 minutes. FMD% more than 10% is considered a normal response. Lower than 10% FMD% reflects endothelial dysfunction, which means a high likelihood to develop cardiovascular event in the future. Subjects with negative FMD% results (the artery is constricted after stress and not dilated as was expected) have the worst prognosis.

- Blood drawn from fasting subjects will be separated and the serum will be frozen until processed as one batch towards the end of the study. Blood will be processed twice – in the beginning of the study and after 3 months.

- Biochemical analysis: soluble pro inflammatory cytokines (interleukin 1, interleukin-1 receptor antagonist, interleukin-2, interleukin-6, tumor necrosis factor alpha, high-sensitivity C Reactive Protein, Pro-Brain Natriuretic Peptide, von-Willebrand Factor) and soluble cell adhesion molecules (E-selectin, P-selectin, Inter Cellular Adhesion Molecule 1, Vascular Cell Adhesion Molecule 1), and apoptotic factors (Anexin 5) using immunological and ELISA methods.

- Assay of colony forming units: Fresh blood will be processed for cell culture assays for endothelial progenitor stem cells colonies counting (a 5 days' protocol). Fifty milliliter of blood will be processed; peripheral-blood mononuclear cells will be isolated by Ficoll density-gradient centrifugation, will be washed twice in phosphate buffered saline with 5% fetal bovine serum and re-suspended in media (EndoCult basal media with supplements; StemCell Technologies, Vancouver, British Columbia, Canada) for EPC colony-forming assay. Cells will be planted on human fibronectin-coated plates (BIOCOAT; Becton Dickenson Labware, Bedford, Massachusetts) at a density of $5X10^6$ cells/well and incubated at 37^0C in humidified 5% CO_2. After 48 hours, the non-adherent cells will be re-plated onto fibronectin-coated 24 well plates at a density of $1X10^6$ cells/well. After 5 days, colony forming units (defined as a central core of rounded cells surrounded by elongated and spindle-shaped cells) will be counted manually in 8 wells out of a 24-well plate.
- Flow Cytometry: characterization of EPCs from peripheral blood will be done by Flow Cytometry (FACS). EPCs will be defined as CD34-, Cd19-, CD3-, CD133+/KDR+ cells.

C.15.3 Day 1 Visit for additional infusion

See Table C1 for the procedures and assessment to be performed during the Day 1 visit of the study. The Day 1 visit will occur after all baseline tests are completed and it has been determined that the subject remains eligible. Once the subject is deemed eligible to continue in the study the subject will be administered the investigational product. The subject will be monitored for 2-3 hours, following administration of the investigational product, and will be sent home the same day.

C.15.4 Month 1 Visit for additional infusion

See Table C1 for the procedures and assessment to be performed for the Month 1 visit of the study. Subject visits should be completed as close to the scheduled visit dates as possible. There will be a +/- window of 2 weeks for the month 1 study visit.

C.15.5 Month 3 and Month 6 Visit for additional infusion

See Table C1 for the procedures and assessment to be performed for month 3 and 6 visit of the study. Subject visit should be completed as close to the scheduled visit dates as possible. There will be a +/- window of 30 days for the month 3 and 6 study visit.

C.15.6 Month 12 Visit

See Table C1 for the procedures and assessments to be performed for Month 12 visit. This visit will be conducted via a phone interview with the subject. A phone script will be provided to the study personnel to use when interviewing the subject. There will be a +/- window of 2 weeks for this visit.

C.16 Statistical Considerations

All subjects will be offered the re-infusion and formal statistical considerations regarding sample size and power are not provided. Based on prior studies, additional infusions of stem cells are safe but strict safety monitoring will be employed to continue the safety assessment of allogeneic infusion of MSCs. Detailed analyses will be described in the separate statistical analysis plan.

11. Reference List

(1) Morley JE, Vellas B, Abellan van KG et al. Frailty consensus: a call to action. *J Am Med Dir Assoc* 2013;14:392-397.

(2) Koller K, Rockwood K. Frailty in older adults: implications for end-of-life care. *Cleve Clin J Med* 2013;80:168-174.

(3) Ebrahimi Z, Wilhelmson K, Eklund K, Moore CD, Jakobsson A. Health despite frailty: Exploring influences on frail older adults' experiences of health. *Geriatr Nurs* 2013.

(4) Ekerstad N, Swahn E, Janzon M et al. Frailty is independently associated with 1-year mortality for elderly patients with non-ST-segment elevation myocardial infarction. *Eur J Prev Cardiol* 2013.

(5) Gale CR, Baylis D, Cooper C, Sayer AA. Inflammatory markers and incident frailty in men and women: the English Longitudinal Study of Ageing. *Age (Dordr)* 2013.

(6) Fabbricotti IN, Janse B, Looman WM, de KR, van Wijngaarden JD, Reiffers A. Integrated care for frail elderly compared to usual care: a study protocol of a quasi-experiment on the effects on the frail elderly, their caregivers, health professionals and health care costs. *BMC Geriatr* 2013;13:31.

(7) Fried LP, Tangen CM, Walston J et al. Frailty in older adults: evidence for a phenotype. *J Gerontol A Biol Sci Med Sci* 2001;56:M146-M156.

(8) Kanapuru B, Ershler WB. Inflammation, coagulation, and the pathway to frailty. *Am J Med* 2009;122:605-613.

(9) Marchionni N, Fattirolli F, Fumagalli S et al. Improved exercise tolerance and quality of life with cardiac rehabilitation of older patients after myocardial infarction: results of a randomized, controlled trial. *Circulation* 2003;107:2201-2206.

(10) Roger VL, Weston SA, Redfield MM et al. Trends in heart failure incidence and survival in a community-based population. *JAMA* 2004;292:344-350.

(11) Barker WH, Mullooly JP, Getchell W. Changing incidence and survival for heart failure in a well-defined older population, 1970-1974 and 1990-1994. *Circulation* 2006;113:799-805.

(12) Newman AB, Gottdiener JS, Mcburnie MA et al. Associations of subclinical cardiovascular disease with frailty. *J Gerontol A Biol Sci Med Sci* 2001;56:M158-M166.

(13) Bouillon K, Batty GD, Hamer M et al. Cardiovascular disease risk scores in identifying future frailty: the Whitehall II prospective cohort study. *Heart* 2013;99:737-742.

(14) Matsuzawa Y, Konishi M, Akiyama E et al. Association between gait speed as a measure of frailty and risk of cardiovascular events after myocardial infarction. *J Am Coll Cardiol* 2013;61:1964-1972.

(15) Fried LP, Kronmal RA, Newman AB et al. Risk factors for 5-year mortality in older adults: the Cardiovascular Health Study. *JAMA* 1998;279:585-592.

(16) Raggi C, Berardi AC. Mesenchymal stem cells, aging and regenerative medicine. *Muscles Ligaments Tendons J* 2012;2:239-242.

(17) Hare JM, Traverse JH, Henry TD et al. A randomized, double-blind, placebo-controlled, dose-escalation study of intravenous adult human mesenchymal stem cells (prochymal) after acute myocardial infarction. *J Am Coll Cardiol* 2009;54:2277-2286.

(18) Hare JM, Fishman JE, Gerstenblith G et al. Comparison of allogeneic vs autologous bone marrow-derived mesenchymal stem cells delivered by transendocardial injection in patients with ischemic cardiomyopathy: the POSEIDON randomized trial. *JAMA* 2012;308:2369-2379.

(19) Weiss DJ, Casaburi R, Flannery R, Leroux-Williams M, Tashkin DP. A Placebo-Controlled, Randomized Trial of Mesenchymal Stem Cells in COPD. *Chest* 2013;143:1590-1598.

(20) Pittenger MF, Mackay AM, Beck SC et al. Multilineage potential of adult human mesenchymal stem cells. *Science* 1999;284:143-147.

(21) Ferreira-Martins J, Ogorek B, Cappetta D et al. Cardiomyogenesis in the developing heart is regulated by c-kit-positive cardiac stem cells. *Circ Res* 2012;110:701-715.

(22) Orlic D, Kajstura J, Chimenti S et al. Bone marrow cells regenerate infarcted myocardium. *Nature* 2001;410:701-705.

(23) Salven P, Mustjoki S, Alitalo R, Alitalo K, Rafii S. VEGFR-3 and CD133 identify a population of CD34+ lymphatic/vascular endothelial precursor cells. *Blood* 2003;101:168-172.

(24) Reya T, Morrison SJ, Clarke MF, Weissman IL. Stem cells, cancer, and cancer stem cells. *Nature* 2001;414:105-111.

(25) Asahara T, Murohara T, Sullivan A et al. Isolation of putative progenitor endothelial cells for angiogenesis. *Science* 1997;275:964-967.

(26) Asahara T, Masuda H, Takahashi T et al. Bone marrow origin of endothelial progenitor cells responsible for postnatal vasculogenesis in physiological and pathological neovascularization. *Circ Res* 1999;85:221-228.

(27) Gehling UM, Ergun S, Schumacher U et al. In vitro differentiation of endothelial cells from AC133-positive progenitor cells. *Blood* 2000;95:3106-3112.

(28) Peichev M, Naiyer AJ, Pereira D et al. Expression of VEGFR-2 and AC133 by circulating human CD34(+) cells identifies a population of functional endothelial precursors. *Blood* 2000;95:952-958.

(29) Huss R. Isolation of primary and immortalized CD34-hematopoietic and mesenchymal stem cells from various sources. *Stem Cells* 2000;18:1-9.

(30) Murry CE, Soonpaa MH, Reinecke H et al. Haematopoietic stem cells do not transdifferentiate into cardiac myocytes in myocardial infarcts. *Nature* 2004;428:664-668.

(31) Le BK, Frassoni F, Ball L et al. Mesenchymal stem cells for treatment of steroid-resistant, severe, acute graft-versus-host disease: a phase II study. *Lancet* 2008;371:1579-1586.

(32) Lee RH, Seo MJ, Reger RL et al. Multipotent stromal cells from human marrow home to and promote repair of pancreatic islets and renal glomeruli in diabetic NOD/scid mice. *Proc Natl Acad Sci U S A* 2006;103:17438-17443.

(33) Nemeth K, Leelahavanichkul A, Yuen PS et al. Bone marrow stromal cells attenuate sepsis via prostaglandin E(2)-dependent reprogramming of host macrophages to increase their interleukin-10 production. *Nat Med* 2009;15:42-49.

(34) Parekkadan B, van PD, Suganuma K et al. Mesenchymal stem cell-derived molecules reverse fulminant hepatic failure. *PLoS One* 2007;2:e941.

(35) Togel F, Hu Z, Weiss K, Isaac J, Lange C, Westenfelder C. Administered mesenchymal stem cells protect against ischemic acute renal failure through differentiation-independent mechanisms. *Am J Physiol Renal Physiol* 2005;289:F31-F42.

(36) Iso Y, Spees JL, Serrano C et al. Multipotent human stromal cells improve cardiac function after myocardial infarction in mice without long-term engraftment. *Biochem Biophys Res Commun* 2007;354:700-706.

(37) Schuleri KH, Feigenbaum GS, Centola M et al. Autologous mesenchymal stem cells produce reverse remodelling in chronic ischaemic cardiomyopathy. *Eur Heart J* 2009;30:2722-2732.

(38) Miyahara Y, Nagaya N, Kataoka M et al. Monolayered mesenchymal stem cells repair scarred myocardium after myocardial infarction. *Nat Med* 2006;12:459-465.

(39) Gong Z, Niklason LE. Use of human mesenchymal stem cells as alternative source of smooth muscle cells in vessel engineering. *Methods Mol Biol* 2011;698:279-294.

(40) Price AP, England KA, Matson AM, Blazar BR, Panoskaltsis-Mortari A. Development of a decellularized lung bioreactor system for bioengineering the lung: the matrix reloaded. *Tissue Eng Part A* 2010;16:2581-2591.

(41) Williams AR, Hare JM. Mesenchymal stem cells: biology, pathophysiology, translational findings, and therapeutic implications for cardiac disease. *Circ Res* 2011;109:923-940.

(42) Mangi AA, Noiseux N, Kong D et al. Mesenchymal stem cells modified with Akt prevent remodeling and restore performance of infarcted hearts. *Nat Med* 2003;9:1195-1201.

(43) Williams AR, Trachtenberg B, Velazquez DL et al. Intramyocardial stem cell injection in patients with ischemic cardiomyopathy: functional recovery and reverse remodeling. *Circ Res* 2011;108:792-796.

(44) Ishizawa K, Kubo H, Yamada M et al. Bone marrow-derived cells contribute to lung regeneration after elastase-induced pulmonary emphysema. *FEBS Lett* 2004;556:249-252.

(45) Spees JL, Pociask DA, Sullivan DE et al. Engraftment of bone marrow progenitor cells in a rat model of asbestos-induced pulmonary fibrosis. *Am J Respir Crit Care Med* 2007;176:385-394.

(46) Spees JL, Whitney MJ, Sullivan DE et al. Bone marrow progenitor cells contribute to repair and remodeling of the lung and heart in a rat model of progressive pulmonary hypertension. *FASEB J* 2008;22:1226-1236.

(47) Bonfield TL, Koloze M, Lennon DP, Zuchowski B, Yang SE, Caplan AI. Human mesenchymal stem cells suppress chronic airway inflammation in the murine ovalbumin asthma model. *Am J Physiol Lung Cell Mol Physiol* 2010;299:L760-L770.

(48) Lee JW, Fang X, Gupta N, Serikov V, Matthay MA. Allogeneic human mesenchymal stem cells for treatment of E. coli endotoxin-induced acute lung injury in the ex vivo perfused human lung. *Proc Natl Acad Sci U S A* 2009;106:16357-16362.

(49) Gao J, Dennis JE, Muzic RF, Lundberg M, Caplan AI. The dynamic in vivo distribution of bone marrow-derived mesenchymal stem cells after infusion. *Cells Tissues Organs* 2001;169:12-20.

(50) Cargnoni A, Gibelli L, Tosini A et al. Transplantation of allogeneic and xenogeneic placenta-derived cells reduces bleomycin-induced lung fibrosis. *Cell Transplant* 2009;18:405-422.

(51) Ortiz LA, Gambelli F, McBride C et al. Mesenchymal stem cell engraftment in lung is enhanced in response to bleomycin exposure and ameliorates its fibrotic effects. *Proc Natl Acad Sci U S A* 2003;100:8407-8411.

(52) Moodley Y, Atienza D, Manuelpillai U et al. Human umbilical cord mesenchymal stem cells reduce fibrosis of bleomycin-induced lung injury. *Am J Pathol* 2009;175:303-313.

(53) Ahn MH, Park BL, Lee SH et al. A promoter SNP rs4073T>A in the common allele of the interleukin 8 gene is associated with the development of idiopathic pulmonary fibrosis via the IL-8 protein enhancing mode. *Respir Res* 2011;12:73.

(54) Kim DY, Kwon EY, Hong GU, Lee YS, Lee SH, Ro JY. Cigarette smoke exacerbates mouse allergic asthma through Smad proteins expressed in mast cells. *Respir Res* 2011;12:49.

(55) Lee SH, Jang AS, Kim YE et al. Modulation of cytokine and nitric oxide by mesenchymal stem cell transfer in lung injury/fibrosis. *Respir Res* 2010;11:16.

(56) Rojas M, Xu J, Woods CR et al. Bone marrow-derived mesenchymal stem cells in repair of the injured lung. *Am J Respir Cell Mol Biol* 2005;33:145-152.

(57) Jiang Y, Jahagirdar BN, Reinhardt RL et al. Pluripotency of mesenchymal stem cells derived from adult marrow. *Nature* 2002;418:41-49.

(58) Kotton DN, Ma BY, Cardoso WV et al. Bone marrow-derived cells as progenitors of lung alveolar epithelium. *Development* 2001;128:5181-5188.

(59) Yamada M, Kubo H, Kobayashi S et al. Bone marrow-derived progenitor cells are important for lung repair after lipopolysaccharide-induced lung injury. *J Immunol* 2004;172:1266-1272.

(60) Chang JC, Summer R, Sun X, Fitzsimmons K, Fine A. Evidence that bone marrow cells do not contribute to the alveolar epithelium. *Am J Respir Cell Mol Biol* 2005;33:335-342.

(61) Kotton DN, Fabian AJ, Mulligan RC. Failure of bone marrow to reconstitute lung epithelium. *Am J Respir Cell Mol Biol* 2005;33:328-334.

(62) Amado LC, Saliaris AP, Schuleri KH et al. Cardiac repair with intramyocardial injection of allogeneic mesenchymal stem cells after myocardial infarction. *Proc Natl Acad Sci U S A* 2005;102:11474-11479.

(63) Shake JG, Gruber PJ, Baumgartner WA et al. Mesenchymal stem cell implantation in a swine myocardial infarct model: engraftment and functional effects. *Ann Thorac Surg* 2002;73:1919-1925.

(64) Kocher AA, Schuster MD, Szabolcs MJ et al. Neovascularization of ischemic myocardium by human bone-marrow-derived angioblasts prevents cardiomyocyte apoptosis, reduces remodeling and improves cardiac function. *Nat Med* 2001;7:430-436.

(65) Toma C, Pittenger MF, Cahill KS, Byrne BJ, Kessler PD. Human mesenchymal stem cells differentiate to a cardiomyocyte phenotype in the adult murine heart. *Circulation* 2002;105:93-98.

(66) Hatzistergos KE, Quevedo H, Oskouei BN et al. Bone marrow mesenchymal stem cells stimulate cardiac stem cell proliferation and differentiation. *Circ Res* 2010;107:913-922.

(67) Beltrami AP, Urbanek K, Kajstura J et al. Evidence that human cardiac myocytes divide after myocardial infarction. *N Engl J Med* 2001;344:1750-1757.

(68) Kraitchman DL, Wilke N, Hexeberg E et al. Myocardial perfusion and function in dogs with moderate coronary stenosis. *Magn Reson Med* 1996;35:771-780.

(69) Kraitchman DL, Young AA, Bloomgarden DC et al. Integrated MRI assessment of regional function and perfusion in canine myocardial infarction. *Magn Reson Med* 1998;40:311-326.

(70) Garot J, Bluemke DA, Osman NF et al. Transmural contractile reserve after reperfused myocardial infarction in dogs. *J Am Coll Cardiol* 2000;36:2339-2346.

(71) Kraitchman DL, Bluemke DA, Chin BB, Heldman AW, Heldman AW. A minimally invasive method for creating coronary stenosis in a swine model for MRI and SPECT imaging. *Invest Radiol* 2000;35:445-451.

(72) Serfaty JM, Atalar E, Declerck J et al. Real-time projection MR angiography: feasibility study. *Radiology* 2000;217:290-295.

(73) Yang X, Atalar E. Intravascular MR imaging-guided balloon angioplasty with an MR imaging guide wire: feasibility study in rabbits. *Radiology* 2000;217:501-506.

(74) Osman NF, McVeigh ER, Prince JL. Imaging heart motion using harmonic phase MRI. *IEEE Trans Med Imaging* 2000;19:186-202.

(75) Osman NF, Prince JL. Visualizing myocardial function using HARP MRI. *Phys Med Biol* 2000;45:1665-1682.

(76) Garot J, Bluemke DA, Osman NF et al. Fast determination of regional myocardial strain fields from tagged cardiac images using harmonic phase MRI. *Circulation* 2000;101:981-988.

(77) Jones JR, Mata JF, Yang Z, French BA, Oshinski JN. Left ventricular remodeling subsequent to reperfused myocardial infarction: evaluation of a rat model using cardiac magnetic resonance imaging. *J Cardiovasc Magn Reson* 2002;4:317-326.

(78) Wise RG, Huang CL, Al-Shafei AI, Carpenter TA, Hall LD. Geometrical models of left ventricular contraction from MRI of the normal and spontaneously hypertensive rat heart. *Phys Med Biol* 1999;44:2657-2676.

(79) Liang J, Zhang H, Hua B et al. Allogenic mesenchymal stem cells transplantation in refractory systemic lupus erythematosus: a pilot clinical study. *Ann Rheum Dis* 2010;69:1423-1429.

(80) Le BK, Frassoni F, Ball L et al. Mesenchymal stem cells for treatment of steroid-resistant, severe, acute graft-versus-host disease: a phase II study. *Lancet* 2008;371:1579-1586.

(81) Psaltis PJ, Zannettino AC, Worthley SG, Gronthos S. Concise review: mesenchymal stromal cells: potential for cardiovascular repair. *Stem Cells* 2008;26:2201-2210.

(82) Schuleri KH, Amado LC, Boyle AJ et al. Early improvement in cardiac tissue perfusion due to mesenchymal stem cells. *Am J Physiol Heart Circ Physiol* 2008;294:H2002-H2011.

(83) Schuleri KH, Feigenbaum GS, Centola M et al. Autologous mesenchymal stem cells produce reverse remodelling in chronic ischaemic cardiomyopathy. *Eur Heart J* 2009;30:2722-2732.

(84) Quevedo HC, Hatzistergos KE, Oskouei BN et al. Allogeneic mesenchymal stem cells restore cardiac function in chronic ischemic cardiomyopathy via trilineage differentiating capacity. *Proc Natl Acad Sci U S A* 2009;106:14022-14027.

(85) Williams AR, Suncion VY, McCall F et al. Durable scar size reduction due to allogeneic mesenchymal stem cell therapy regulates whole-chamber remodeling. *J Am Heart Assoc* 2013;2:e000140.

(86) Liang J, Zhang H, Hua B et al. Allogenic mesenchymal stem cells transplantation in refractory systemic lupus erythematosus: a pilot clinical study. *Ann Rheum Dis* 2010;69:1423-1429.

(87) Kinkaid HY, Huang XP, Li RK, Weisel RD. What's new in cardiac cell therapy? Allogeneic bone marrow stromal cells as "universal donor cells". *J Card Surg* 2010;25:359-366.

(88) Zhuo Y, Li SH, Chen MS et al. Aging impairs the angiogenic response to ischemic injury and the activity of implanted cells: combined consequences for cell therapy in older recipients. *J Thorac Cardiovasc Surg* 2010;139:1286-94, 1294.

(89) Castillo E AL, Gerber B et al. Myocardial Infarction size assessment with single breath-hold three-dimensional inversion-recovery-prepared MRI. J Cardiovasc Magn Reson. 2002;4:25.

(90) Foo T CE, Kraitchman DL, Wu K, Bluemke DA, Lima JA. Three dimensional breath-held imaging of myocardial infarction using variable sampling in time (VAST). J Cardiovasc Magn Reson. 2002;4:123

(91) Sundin M., Orvell C., Rasmusson I, Sundberg B., Rngden O., Le BK. Mesenchymal stem cells are susceptible to human herpesviruses, but viral DNA cannot be detected in the healthy seropositive individual. Bone Marrow Transplant 2006; 37:1051-1059.

(92) Ball, L. M., et al. "Multiple infusions of mesenchymal stromal cells induce sustained remission in children with steroid-refractory, grade III-IV acute graft-versus-host disease." Br.J.Haematol. 163.4 (2013): 501-09.

(93) He, Z., J. Hua, and Z. Song. "Concise Review: Mesenchymal Stem Cells Ameliorate Tissue Injury via Secretion of Tumor Necrosis Factor-alpha Stimulated Protein/Gene 6." Stem Cells Int. 2014 (2014): 761091.

(94) Koc, O. N., et al. "Allogeneic mesenchymal stem cell infusion for treatment of metachromatic leukodystrophy (MLD) and Hurler syndrome (MPS-IH)." Bone Marrow Transplant. 30.4 (2002): 215-22.

(95) Pittenger, M. F., et al. "Multilineage potential of adult human mesenchymal stem cells." Science 284.5411 (1999): 143-47

(96) Wang, D., et al. "Double allogenic mesenchymal stem cells transplantations could not enhance therapeutic effect compared with single transplantation in systemic lupus erythematosus." Clin.Dev.Immunol. 2012 (2012): 273291.

Change Document CRATUS Protocol

From July 22, 2013 Version 1.0 to August 19, 2013 Version 1.1

**Location in Document**	**Details of Change**
Cover Page	Changed the date from to July 22, 2013 to August 19, 2013.
All Pages	Changed header to reflect new version date
Page 10: Synopsis	Added: "by the Investigator with a frailty score of 4 to 7 using the Clinical Frailty Scale."
Page 37: Section 4.1 – Inclusion Criteria	Added: "by the Investigator with a frailty score of 4 to 7 using the Clinical Frailty Scale."
Page 43: Section 6.4.1 – Infusion Monitoring	Added:<br><br>"6.4.1. Infusion Monitoring<br><br>Subjects will be monitored in the ICU for two hours prior to infusion to establish baseline vital signs (oxygen saturation, heart rate, blood pressure, and temperature) every 15 minutes. Monitoring will also continue throughout the infusion.<br><br>Once the infusion is begun, 2L/min oxygen via nasal cannula will be provided if the oxygen saturation drops below 90% on room air. The infusion will be stopped if the oxygen saturation does not return to >93% within 3 minutes of initiating supplemental oxygen or if the patient requires greater than 2L/min supplemental oxygen to achieve the required saturation of >93%. If a patient requires the addition of oxygen, it will be continued for 4 hours after the completion of the infusion. At that time, oxygen will be weaned off to maintain a saturation >93% on room air."
Page 46: Section 7.1 – Time and Events Schedule, Table 2	Added Dobutamine Stress Echo Test (DSE) to Month 6 follow-up Visit on the Time and Events Table
Page 46: Section 7.1 – Time and Events Schedule, Table 2	Added Immune Monitoring to Week 2, Month 1, and Month 3 visits on the Time and Events Table
Page 48: Section 7.2.1 – Screening Visit	Added:<br><br>"Dobutamine Stress Echocardiography (DSE)<br><br>A Dobutamine Stress Echocardiography will be performed twice during the study, once at screening and at the Month 6 follow-up visit. This exam will assist in mimicking the effect of exercise on subjects to assess the heart muscle when under stress to better evaluate ejection fraction in frail patients."
Page 50: Section 7.2.6 – Immune Monitoring for Graft Rejection	Added "Additionally, in female patients who receive allogeneic hMSCs, the stored baseline serum will be analyzed to evaluate the antibody responses to HLA and H-Y antigens."
Page 52: Section 8.2.4 – Subject Stopping Guidelines	Added "The first more conservative stopping guideline is to monitor patients for unexpected SAEs where there is a reasonable possibility that the study

	product or administration procedure caused the event within 30 days of administration including patient death, grade 3 myocardial infarction, grade 3 hemodynamically unstable ventricular tachycardia or grade 3 LV perforation. Study accrual and further treatment of patients will be put on hold if any patients experience one of these events. The DSMB will be notified within 24 hours of the occurrence of these events and will be convened within 3 business days to review the event and study.”
Page 53: Section 8.2.4 – Subject Stopping Guidelines	Added “It is designed to assist the independent DSMB in overseeing the study. The DSMB may also request additional interim analyses and develop other criteria including provision for monitoring of potential late effects to determine when to intervene in the enrollment or treatment of patients in the study.”
Page 70: Appendix 1 – Infusion Guidelines	Added “Procedures post-infusion:<br><br>1.The subject will be monitored for 2 hours post IP infusion with continuous pulse oximetry<br><br>2.Vital signs will be monitored every 15 minutes, 30 minutes, 1 hour, and 2 hours post IP infusion<br><br>3. If the O_2 saturation decreases to < 90% over a continual period of 3 minutes then supplemental oxygen may be added or increased during the two hours post-infusion observation period.<br><br>4. Minimum observation for subjects post-infusion is two hours.<br><br>5. After the minimum two hour observation period, subject may be discharged from the site if no complaints are experienced, such as shortness of breath or other objective signs of cardiorespiratory compromise.<br><br>6. Subjects not meeting criteria for discharge, after the initial two hour post-infusion observation period, will be delayed discharge for a minimum of an additional hour.<br><br>7. The investigator will assess at the end of the three hour observation period if the subject will be discharged or require hospitalization.<br><br>8. At the end of the three hour observation period if a subject’s O2 saturation stays below 90% then the subject will require admission to the hospital unless specified by the Investigator.<br><br>Individual Stopping Guidelines:<br><br>Subjects still experiencing cardiorespiratory signs or symptoms, such as shortness of breath, tachypnea, tachycardia, hypotension, palpitations, or other objective signs of cardiorespiratory compromise, more than three hours after post-infusion will continue with all scheduled follow-up procedures, unless considered unsafe in the Investigator’s opinion.<br>If the subject experiences cardiorespiratory signs or symptoms during the infusion, the procedure will be stopped and no further IP will be delivered to the subject. The subject will continue with all scheduled follow-up procedures if considered safe in the opinion of the Investigator.”

Change Document CRATUS Protocol

From August 19, 2013 Version 1.1 to September 16, 2013 Version 1.2

**Location in Document**	**Details of Change**
Cover Page	Changed the date from to August 19, 2013 to September 16, 2013.
All Pages	Changed header to reflect new version date
Page 46: Section 7.1 – Time and Events Table	Bone Density Scan was added to Month 6 of the study

Change Document CRATUS Protocol

From September 16, 2013 Version 1.2 to November 13, 2013 Version 1.3

**Location in Document**	**Details of Change**
Cover Page	Changed the date from to September 16, 2013 to November 13, 2013.
All Pages	Changed header to reflect new version date
Cover Page	Version date changed from Version 1.2 to 1.3
Page 8:	Added "The first three (3) patients in each treatment group will not be treated less than 5 days apart and will each undergo full evaluation for 5 days to demonstrate there is no evidence of treatment emergent SAE's, defined as the composite of: death, non-fatal pulmonary embolism, stroke, hospitalization for worsening dyspnea and clinically significant laboratory test abnormalities, prior to proceeding with the treatment of further patients."
Page 37: Section 3.1 – Study Design	Added "The first three (3) patients in each treatment group will not be treated less than 5 days apart and will each undergo full evaluation for 5 days to demonstrate there is no evidence of treatment emergent SAE's, defined as the composite of: death, non-fatal pulmonary embolism, stroke, hospitalization for worsening dyspnea and clinically significant laboratory test abnormalities, prior to proceeding with the treatment of further patients."
Page 41: Section 5.5 – Biomarker Assessment	Added new section, "A separate 7 mL blood sample for gene expression profiling of WBC RNA will be obtained at the donation visit. All samples will be identified so that they can be linked to individual patients. These samples may be stored indefinitely. Individual results will not be returned to the patient or the study physician. The samples will be linked to patients, but there will be no recontact. Data presented in publications will not contain individual patients' gene expression or clinical characteristics or outcomes; only aggregate data from the entire study will be disclosed."
Page 60: Section 9.3 – Interim Analyses	Removed "Safety data analysis will be performed on the first 3 patients enrolled at Week 4 after infusion. After the first interim safety analysis, summaries of adverse events will be reviewed each week and detailed safety data will be reviewed every three months by the DSMB."
Page 60: Section 9.3 – Interim Analyses	Added "Interim analyses will be conducted at times coincident with regularly scheduled meetings of the Data and Safety Monitoring Board (DSMB) at approximately six-month intervals. The DSMB Chair will be notified each time an SAE occurs. After all patients in phase I have been followed for 30 days, at that time an independent DSMB will review all available data to make an independent recommendation to either keep the specified randomized dose 1:1:1 or to recommend a dose modification for the randomized placebo study.

	Policies of the DSMB will be described in the DSMB Charter, which will be prepared by the DSMB prior to study initiation. The stopping guidelines serve as a trigger for consultation with the DSMB for additional review, and are not formal "stopping rules" that would mandate automatic closure of study enrollment."

Change Document CRATUS Protocol

From November 13, 2013 Version 1.3 to November 19, 2013 Version 1.4

**Location in Document**	**Details of Change**
Cover Page	Changed the date from to November 13, 2013 to November 19, 2013.
All Pages	Changed header to reflect new version date
Cover Page	Version date changed from Version 1.3 to 1.4
Page 7: List of Abbreviations	Added “MMSE-Mini Mental State Examination”
Page 10: Synopsis – Exclusion criteria	Added “Score of $\leq$24 on the Mini Mental State Examination (MMSE)”
Page 36: Section 4.2 – Exclusion Criteria	Added “Score of $\leq$24 on the Mini Mental State Examination (MMSE)”
Page 46: Section 7.1 – Table 2:Time and Events Table	Added “+/- 2 days” to week 2
Page 46: Section 7.1 – Table 2:Time and Events Table	Added Mini Mental State Examination to the time and events table as a screening procedure.

Change Document CRATUS Protocol

From November 19, 2013 Version 1.4 to December 9, 2013 version 2

**Location in Document**	**Details of Change**
Title page and Page Headers	Changed the date from to November 19, 2013 to December 9, 2013.
All Pages	Protocol version number updated to version 2
Page 39: Section 5 – Mesenchymal Stem Cell Donors	Removed "…anti…"<br>Added "…with the exception of CMV…"
Page 40: Section 5.2 – Normal Donor Eligibility	Females donors are being added and the following language was added in response "Donors (male and female)…"
Page 40: Section 5.2 – Normal Donor Eligibility	Donor age was increased from "35" to "45".
Page 40: Section 5.2 – Normal Donor Eligibility	Removed "Women will not be eligible to be normal donors because women who have had prior pregnancies may acquire antibodies to male antigens.
Page 41: Section 5.2 – Normal Donor Eligibility	Added "… and female…" under eligibility criteria for normal donors
Page 41: Section 5.2 – Normal Donor Eligibility	Added "Negative serum or urine pregnancy test for female donors"<br>"Female donors would need to be screened for pregnancy as the procedure may be an added risk to a fetus."
Page 44: Section 5.6 – Blinding and Unblinding	Added "Before dispensing the investigational product, Cell Therapy Lab staff will confirm the CMV status of eligible recipient. This information will be used to select Allo- MSC product. CMV status of the recipient and donor of the Allo-MSC product will be matched. CMV positive Allo-MSC product will only be infused to a CMV positive recipient. All CMV negative recipients will receive CMV negative Allo-MSC product."
Page 46: Section 7.1 – Table 2:Time and Events Table	Added Mini Mental State Examination to the time and events table for Month 6 visit, previously only completed at screening.
Page 46: Section 7.1 – Table 2:Time and Events Table	Added "…and CMV" to the procedures table
Page 50: Section 8.2 – Laboratory Evaluations	Added "…CMV…"
Page 69: Appendix 1 – Infusion Guidelines	Revised bullet #3 from "IA" to "IP" to clarify
Page 69: Appendix 1 – Infusion Guidelines	Added to bullet #6 "IV Infusion" and removed "Three-way Stopcock"
Page 69: Appendix 1 – Infusion Guidelines	Prior to the start of IP infusion section of the guidelines, the following bullets were removed:<br><br>"7. Connect stopcock to IV tubing and connect extension tubing to the stopcock.

<table>
<tr><td></td><td colspan="2">8. Hang 0.9% normal saline infusion bag and flush the IV line such that it flows directly through a stopcock

9. Connect IV line to subject’s IV access.”</td></tr>
<tr><td>Page 70: Appendix 1 – Infusion Guidelines</td><td colspan="2">During the IP infusion section of the guidelines, the following additional language was added, “8. At the end of the IP infusion, close the line and flush 25ml of 0.9% normal saline into the luer lock connector on the bottom of the IP bag, reopen line and allow to infuse at a rate of 2mL/min until completion.”</td></tr>
<tr><td>Page 70: Appendix 1 – Infusion Guidelines</td><td>Original Language under

“Procedures post infusion”

1. The subject will be monitored for 2 hours post IP infusion with continuous pulse oximetry.

2. Vital signs will be monitored every 15 minutes, 30 minutes, 1 hour, and 2 hours post IP infusion.

3. If the O2 saturation decreases to < 90% over a continual period of 3 minutes then supplemental oxygen may be added or increased during the two hours post-infusion observation period.

4. Minimum observation for subjects post-infusion is two hours.

5. After the minimum two hour observation period, subject may be discharged from the site if no complaints are experienced, such as shortness of breath or other objective signs of cardiorespiratory compromise.

6. Subjects not meeting criteria for discharge, after the initial two hour post-infusion observation period, will be delayed discharge for a minimum of an additional hour.

7. The investigator will assess at the end of the three hour observation period if the subject will be discharged or require hospitalization.

8. At the end of the three hour observation period if a subject’s O2 saturation stays below 90% then the subject will require</td><td>Revised Language under

“Procedures post infusion”

1. Vital signs will be monitored at 15 minutes, 30 minutes, 1 hour, and 2 hours post IP infusion.

2. The subject will be monitored for a minimum of 2 hours post IP infusion with continuous pulse oximetry.

3. If the O2 saturation decreases to < 90% over a continual period of 3 – 5 minutes then supplemental oxygen may be added or increased during the two hours post-infusion observation period.

4. If at the end of the 2 hour observation period, if a subject’s O2 saturation stays below 90% then the subject will be provided additional oxygen to maintain a saturation of >90% at room air up to 4 hours post infusion.

5. After the minimum two hour observation period, the subject will be continuously monitored and discharged the following day, if no complaints are experienced, such as shortness of breath or other objective signs of cardiorespiratory compromise.

6. Subjects not meeting criteria for discharge will be assessed by the Investigator during the observation period to further determine hospitalization otherwise not</td></tr>
</table>

	admission to the hospital unless specified by the Investigator.	specified in the protocol.

Change Document CRATUS Protocol

From December 9, 2013 Version 2 to January 22, 2014 Version 2.1

**Location in Document**	**Details of Change**	
Cover Page	Changed the date from to December 9, 2013 to January 22, 2014.	
Cover Page	Changed the version from 2 to version 2.1	
All Pages	Changed header to reflect new version date	
Page 10: Synopsis	Revised inclusion criteria to reflect inclusion criteria in section 4.1	
	<u>Original Language</u><br><br>• Diagnosis or symptoms of frailty by the Investigator with a frailty score of 4 to 7 using the Clinical Frailty Scale.<br>• Provide written informed consent.<br>• Subjects age 60≥ years at the time of signing the Informed Consent<br>• Female subjects must be surgically sterile or post-menopausal (>1 year).	<u>Revised Language</u><br><br>• Provide written informed consent.<br>• Subjects age ≥60 and ≤95 years at the time of signing the Informed Consent Form.<br>• Show signs of frailty apart from a concomitant condition as assessed by the Investigator with a frailty score of 4 to 7 using the Canadian Clinical Frailty Scale.<br>• Female subjects must be surgically sterile or post-menopausal (>1 year).
Page 47: Section 7.1 – Time and Events Table	Added Smell Identification Test (UPSIT) to baseline, Month 3 and Month 6	
Page 49: Section 7.2.1 – Baseline Visit	Added:<br><br>"The Smell Identification Test (UPSIT) test will be performed once at baseline and once at the Month 3 and Month 6 follow-up visits. Age-related olfactory dysfunction is felt to be due to cumulative inflammatory damage affecting the olfactory mucosa. The Smell Identification Test (UPSIT) is a self-administered 40-item olfactory test. It provides an absolute indication of smell loss (anosmia, mild, moderate, or severe microsomia) as well as an index to detect malingering."	

Change Document CRATUS Protocol

From January 22, 2014 Version 2.1 to March 5, 2014 Version 2.2

**Location in Document**	**Details of Change**
Cover Page	Changed the date from January 22, 2014 to March 5, 2014.
Cover Page	Changed the version from 2.1 to version 2.2
All Pages	Changed header to reflect new version date
Page 6: List of Abbreviations	Removed "GGT : Y-glutamyl transaminase"
Page 8: Study objectives	Added "…endothelial function…"
Page 35: Section 2.1.2 – Secondary objectives	Added "…endothelial function…"
Page 36: Section 2.2.1 – Primary endpoints (Safety)	Removed "…GGT (γ-glutamyl transaminase),…"
Page 38: Section 4.1 – Inclusion Criteria	Added "…Canadian…"
Page 38: Section 4.1 – Inclusion Criteria	Added "Female subjects must be surgically sterile or post-menopausal (>1 year).", which was initially listed on the synopsis inclusion/exclusion criteria but not in the synopsis.
Page 46: Section 7.1 – Time and events table	Removed "Day – 14" from the baseline visit
Page 46: Section 7.1 – Time and events table	Revised baseline window to read "(-4weeks)" instead of "(-2 to 4 weeks)"
Page 46: Section 7.1 – Time and events table	Added the following optional assessments to baseline and month 3 visits:<br>"Optional: Brachial Ultrasound<br>Optional: Endothelial blood samples%"
Page 47: Section 7.1 – Time and events key	Added:<br>% - Optional: An additional 5 lavender top tubes (EDTA) will be drawn.<br>& - Optional brachial ultrasound to assess endothelial function.
Page 49: Section 7.1 – Time and events key	Added:<br><br>"Endothelial function (Optional Assessments) will occur upon the subject signing the optional section of the informed consent form (ICF). No endothelial function tests will take place until the patient is fully informed of the research and signs the optional portion of the consent form.<br><br>- Brachial ultrasound testing and blood collection will be performed to assess endothelial function in the aging frailty population at baseline and 3 months post stem cell infusion. This will help provide cumulative data in assessing whether or not stem cell infusion improves endothelial function.<br><br>- Flow Mediated Diameter percent change (FMD%): All measurements of the brachial artery diameter and FMD will be performed in the morning, in a quiet and dark room and at controlled ambient temperatures between 20°C and 26°C. Studies will be conducted after an overnight fast of at least 10 hours (water is permitted), with the subjects supine and after 10 minutes of rest. The subject's right arm will be comfortably immobilized in an extending position, allowing for ultrasound scanning of the brachial artery 5–10 cm above the antecubital fossa. In each examination, recording of vessel images will be followed by inflation of a

	cuff to supra-systolic pressure (40 to 50 mmHg above systolic pressure) for 5 minutes. Then the cuff will be deflated and the brachial artery diameter will be imaged and recorded for 3 minutes. FMD% more than 10% is considered a normal response. Lower than 10% FMD% reflects endothelial dysfunction, which means a high likelihood to develop cardiovascular event in the future. Subjects with negative FMD% results (the artery is constricted after stress and not dilated as was expected) have the worst prognosis.<br><br>- Blood drawn from fasting patients will be separated and the serum will be frozen until processed as one batch towards the end of the study. Blood will be processed twice – in the beginning of the study and after 3 months.<br><br>- Biochemical analysis: soluble pro inflammatory cytokines (interleukin 1, interleukin-1 receptor antagonist, interleukin-2, interleukin-6, tumor necrosis factor alpha, high-sensitivity C Reactive Protein, Pro-Brain Natriuretic Peptide, von-Willebrand Factor) and soluble cell adhesion molecules (E-selectin, P-selectin, Inter Cellular Adhesion Molecule 1, Vascular Cell Adhesion Molecule 1), and apoptotic factors (Anexin 5) using immunological and ELISA methods.<br><br>- Assay of colony forming units: Fresh blood will be processed for cell culture assays for endothelial progenitor stem cells colonies counting (a 5 days' protocol). Fifty milliliter of blood will be processed; peripheral-blood mononuclear cells will be isolated by Ficoll density-gradient centrifugation, will be washed twice in phosphate buffered saline with 5% fetal bovine serum and re-suspended in media (EndoCult basal media with supplements; StemCell Technologies, Vancouver, British Columbia, Canada) for EPC colony-forming assay. Cells will be planted on human fibronectin-coated plates (BIOCOAT; Becton Dickenson Labware, Bedford, Massachusetts) at a density of 5X106 cells/well and incubated at 370C in humidified 5% CO2. After 48 hours, the non-adherent cells will be re-plated onto fibronectin-coated 24 well plates at a density of 1X106 cells/well. After 5 days, colony forming units (defined as a central core of rounded cells surrounded by elongated and spindle-shaped cells) will be counted manually in 8 wells out of a 24-well plate.<br><br>- Flow Cytometry: characterization of EPCs from peripheral blood will be done by Flow Cytometry (FACS). EPCs will be defined as CD34-, Cd19-, CD3-, CD133+/KDR+ cells.
Page 48: Section 7.2.2 – Baseline visit	Removed "…the screening visit…" and replaced with "…treatment…"
Page 50: Section 8.2 – Laboratory Evaluations	Removed "…GGT (γ-glutamyl transaminase),…"
Page 51: Section 7.2.6 – Biomarker assessment	Added section 7.2.6<br><br>"Biomarker Assessment<br>A separate 7 mL blood sample for gene expression profiling of WBC RNA will be obtained at the donation visit. All samples will be identified so that they can be linked to individual patients. These samples may be stored indefinitely. Individual results will not be returned to the patient or the study physician. The samples will be linked to patients, but there will be no recontact. Data presented in publications will not contain individual patients' gene expression or clinical characteristics or outcomes; only aggregate data from the entire study will be disclosed. "

Change Document CRATUS Protocol

From March 5, 2014 Version 2.2 to April 10, 2014 Version 2.3

**Location in Document**	**Details of Change**	
Cover Page	Changed the date from March 5, 2014 to April 10, 2014 Version 2.3.	
Cover Page	Changed the version from 2.2 to version 2.3	
All Pages	Changed header to reflect new version date	
Page 43: Section 6.4 – Administration Rate	<u>Original Language</u>:<br><br>In the proposed study, the cell dose to be delivered is 1x 10^{8} , and 2×10^{8}hMSC/infusion, reconstituted in a total volume of 25ml, delivered at a rate of 2 ml/min, and delivered at a maximum rate of 16×10^{6} hMSC/minute and will last approximately 12.5 minutes +/- 25 minutes. The infusion bag will be flushed with an additional 25 ml of 0.9% normal saline at the completion of allo-hMSC infusion and delivered at a rate of 2ml/min.	<u>Revised Language</u>:<br><br>In the proposed study, the cell dose to be delivered is 20x 10^{6} , and 100×10^{6}hMSC/infusion, and 200X10^{6} reconstituted with the 2.5 million hMSC/ml, in the following total volume<br><br>- 25 ml for 20 million dose (5million hMSC/min)<br>- 40ml for 100 million dose (5million hMSC/min)<br>- 80ml for 200 million dose (5million hMSC/min)<br><br>Cell will be delivered at a rate of 2ml/min, and delivered at a maximum rate of 16×10^{6} hMSC/minute and will last approximately:<br><br>- 12.5 minutes for 25ml for 20 million dose<br>- 20 minutes for 40ml for 100 million dose<br>- 40 minutes for 80ml for 200 million dose<br><br>The infusion bag will be flushed with an additional 25 ml of 0.9% normal saline at the completion of allo-hMSC infusion and delivered at a rate of 2ml/min.

Change Document CRATUS Protocol

From April 10, 2014 Version 2.3 to May 7, 2014 Version 2.4

<table>
<tr><th>Location in Document</th><th colspan="2">Details of Change</th></tr>
<tr><td>Cover Page</td><td colspan="2">Changed the date from April 10, 2014 Version 2.3 to May 7, 2014 Version 2.4.</td></tr>
<tr><td>Cover Page</td><td colspan="2">Changed the version from 2.3 to version 2.4</td></tr>
<tr><td>All Pages</td><td colspan="2">Changed header to reflect new version date</td></tr>
<tr><td>Page 10: Synopsis – Exclusion</td><td colspan="2">Added "…melanoma in situ… if recurrence occurs."</td></tr>
<tr><td rowspan="2">Page 38: Section 4.2 – Exclusion Criteria</td><td colspan="2">Added "…melanoma in situ…if recurrence occurs."</td></tr>
<tr><td><u>Original Language</u>

Have a clinical history of malignancy within 5 years (i.e., patients with prior malignancy must be disease free for 5 years), except curatively-treated basal cell carcinoma, squamous cell carcinoma or cervical carcinoma.</td><td>Revised Language

Have a clinical history of malignancy within 5 years (i.e., patients with prior malignancy must be disease free for 5 years), except curatively-treated basal cell carcinoma, squamous cell carcinoma, melanoma in situ or cervical carcinoma if recurrence occurs.</td></tr>
<tr><td>Page 48: Section 7.1 – Time and Events table</td><td colspan="2">Added "…VEGF.." as a functional assay</td></tr>
</table>

Change Document CRATUS Protocol

From May 7, 2014 Version 2.4 to July 10, 2014 Version 2.5

**Location in Document**	**Details of Change**
Cover Page	Changed the date from May 7, 2014 Version 2.4 to July 10, 2014 Version 2.5.
Cover Page	Changed the version from 2.4 to version 2.5
All Pages	Changed header to reflect new version date
Page 44: Section 6.4 – Administration rate	Added "***For the randomized double blinded phase we will prepare the following doses of 100 million, 200 million and Placebo in an 80ml bag.***"

Change Document CRATUS Protocol

From July 10, 2014 Version 2.5 to July 31, 2014 Version 2.6

**Location in Document**	**Details of Change**
Cover Page	Changed the date from July 10, 2014 Version 2.5 to July 31, 2014 Version 2.6.
Cover Page	Changed the version from 2.5 to version 2.6
All Pages	Changed header to reflect new version date
Page 38: Section 4.2 – Exclusion Criteria	Added the following language to bullet #4 "…not due to a reversible cause (i.e. Coumadin)…"

Change Document CRATUS Protocol

From July 31, 2014 Version 2.6 to September 12, 2014 Version 3

Location in Document	Details of Change
Cover Page	Changed the date from July 31, 2014 Version 2.5 to September 12, 2014 Version 3
All Pages	Changed header to reflect new version date
Page 2-4: Table of Contents	Table of Contents pages updated
Page 6: List of Abbreviations	Added the following abbreviations:<br>- CHAMPS: Community Healthy Activities Model Program for Seniors questionnaire<br>- MFI: Multi-dimensional Fatigue Inventory<br>- SPPB: Standard Physical Performance Battery
Page 9: Definition of Endpoints	Added "…During the baseline, 3 and 6 month visits." Additionally added language next to the following end points:<br>- Reduced Activity (assessed via CHAMPS questionnaire)<br>- Slowing of Mobility (assessed via gait speed test)<br>- Diminished handgrip strength (assessed via dynamometer and SPPB questionnaire)<br>- Exhaustion (assessed via the MFI questionnaire)
Page 36: Section 2.2.2 – Secondary Endpoints (Efficacy)	Added "…During the baseline, 3 and 6 month visits." Additionally added language next to the following end points:<br>- Reduced Activity (assessed via CHAMPS questionnaire)<br>- Slowing of Mobility (assessed via gait speed test)<br>- Diminished handgrip strength (assessed via dynamometer and SPPB questionnaire)<br>- Exhaustion (assessed via the MFI questionnaire)
Page 37: Section 3.2 – Randomization Study	Removed from the last paragraph "…manual…sealed, opaque envelopes…" and replaced with "…electronic…the Advantage EDC system…"
Page 37: Section 3.2 – Randomization Study	In the randomized phase of the trial, subjects which received placebo will have the option to receive allogeneic hMSCs , if all study endpoints are met. If all endpoints are met then the subject will be administered the study drug and follow the study schedule from Day 1 to Month 12 after receiving the infusion of allogeneic hMSCs.
Page 47: Section 7.1 – Time and Events Table	Added the following assessments:<br>- 4 meter gait speed test was added to baseline, month 3 and month 6 visits<br>- QOL questionnaires added to assess endpoints were added (CHAMPS,

	SPPB, MFI)<br>- Time and event key was revised from symbols to numbers for easier reference
Page 48: Section 7.1 – Time and Events Key	Added the following:<br>- Time and event key was revised from symbols to numbers for easier reference<br>- 7 – The following Qol Questionnaires will only be assessed at baseline, month 3 and 6 visits:<br>• CHAMPS, SPPB, and MFI<br>- 8 – 4 meter gait speed test will be performed twice per visit and the average of the exams will be taken.<br>- 9 – If the visit is completed 2 weeks from the week 2 visit then a follow-up phone call will be made to the patient.
Page 48: Section 7.1 – Time and Events Key	An average of the two (2) exams performed for the 4 meter walk test will be used to assess the exam.
Page 51: Section 7.2.5 – Month 12 Visit	Added additional language based on minutes, "A phone script will be provided to the study personnel to use when interviewing the subject."

Change Document CRATUS Protocol
From September 12, 2014, Version 3 to September 23, 2014, Version 3.1

Location in Document	Details of Change
Cover Page	Changed the date from September 12, 2014, Version 3 to September 23, 2014, Version 3.1
All Pages	Changed header to reflect new version date
Page 2-4: Table of Contents	Table of Contents pages updated
Page 9: Synopsis – Investigational Plan	Added "…one..." month to the last paragraph.
Page 9: Synopsis – Duration of study endpoints	Added "…one..." month, which is another follow-up visit
Page 9: Synopsis – Definition of endpoints	Removed to be consistent with language in section 2.2.1 "…MI, stroke, hospitalization."
Page 9: Synopsis – Definition of endpoints	Replaced to be consistent with language present in section 2.2.1 "pulmonary embolism, stroke, hospitalization for worsening dyspnea and clinically significant laboratory test abnormalities."
Page 9: Synopsis – Definition of endpoints	Replaced next to SPPB "questionnaire" with SPPB "assessment" as this exam is a series of exams administered to the subject by a study team member.
Page 10: Synopsis – Inclusion Criteria	Removed inclusion criteria as it conflicts with exclusion criteria "•Female subjects must be surgically sterile or post-menopausal (>1 year)."
Page 10: Synopsis – Exclusion criteria	Deleted "Female subjects capable of childbearing, currently pregnant or nursing." and replaced with the following to be consistent with language present in section 4.2 "• Be a female who is pregnant, nursing, or of childbearing potential while not practicing effective contraceptive methods. Female patients must undergo a blood or urine pregnancy test at screening and within 36 hours prior to injection.<br>• Female subjects must have an FSH < 25.8 IU/L"
Page 36: Section 2.1.1 – Primary objective	Primary objective was listed in the synopsis but not added to the body of the protocol. The following language from the synopsis was added<br>"- To demonstrate the safety of allogeneic hMSCs administered in patients with Frailty and to explore treatment efficacy (decrease in frailty, frequency of acute exacerbations, change in symptom related quality of life, improved cardiovascular status, decrease in inflammatory biomarkers, endothelial function and 1 year survival)."
Page 37: Section 2.2.1 – Primary endpoints (Safety)	Removed "…phosphate…" per phone call with the CRO on June 11, 2014.
Page 37: Section 2.2.2 – Secondary endpoints (Efficacy)	Replaced next to SPPB "questionnaire" with SPPB "assessment" as this exam is a series of exams administered to the subject by a study team member.
Page 37: Section 2.2.2 – Secondary endpoints (Efficacy)	Removed "…CMV…" from inflammatory markers, as per the call with the CRO on June 11, 2014.
Page 38: Section 3.1 – Description of the study	Added to the last paragraph "In the pilot phase…" to clarify that this is relative only to the pilot phase.
Page 38: Section 3.2 – Randomization Study	Clarified to coincide with language in Section 2.2.2 regarding secondary efficacy endpoints by adding the following to the first paragraph "…at

	baseline, 3 and 6 months."
Page 39: Section 4.2 – Exclusion criteria	Removed inclusion criteria as it conflicts with exclusion criteria "•Female subjects must be surgically sterile or post-menopausal (>1 year)."
Page 43: Section 5.4 – Follow-up Schedule for Donors	Removed following language "…periodic…over a period of one year…"
Page 48: Section 7.1 – Time and Events table	Replaced "walk" with "gait speed" for the 4 meter exam.
Page 48: Section 7.1 – Time and Events table	SPPB Assessment added as a separate assessment.
Page 49: Section 7.1 – Time and Events table	In the top row of the table grid, the week numbers were revised per the January 27, 2014 study call as follows:<br>Month 1 (Week 4) now reads **Month 1 (Day 30)**<br>Month 3 (Week 12) now reads **Month 3 (Day 90)**<br>Month 6 (Week 24) now reads **Month 6 (Day 180)**<br>Month 12 (Week 48) now reads **Month 12 (Day 365)**
Page 49: Section 7.1 – Time and Events table	Added "(fractionate if total >1.5 times normal)" to be consistent with section 8.2 – Laboratory evaluations.<br>Added "…carbon dioxide…"
Page 49: Section 7.1 – Time and Events table	Removed "…bicarbonate…"
Page 49: Section 7.1 – Time and Events table	Removed due to error "7 – The following Qol Questionnaires will only be assessed at baseline, month 3 and 6 visits:<br>• CHAMPS, SPPB, and MFI"
Page 49: Section 7.1 – Time and Events table	Removed bullet #9 in the time and events table "If the visit is completed 2 weeks from the week 2 visit then a follow-up phone call will be made to the patient."
Page 49: Section 7.1 – Time and Events table	Added bullet #9 as follows "The first scan will be of the hip and spine for bone density and the second will be to assess the total body composition."

Change Document CRATUS Protocol
From September 23, 2014, Version 3.1 to March 4, 2015, Version 4

Location in Document	Details of Change
Cover Page	Changed the date from September 23, 2014, Version 3.1 to March 4, 2015, Version 4
All Pages	Changed header to reflect new version date
Throughout the entire study protocol	Language was revised through the entire protocol to reflect "subject" instead of "patient"
Page 2-4: Table of Contents	Table of Contents pages updated
Page 9: Synopsis – Investigational Plan	Added Additional Language "At the pilot subjects one year phone call visit, all fifteen (15) subjects will be provided with the option of having a second administration of allogeneic hMSCs: 1 x 10^8 (100 million) cells delivered via peripheral intravenous infusion."
Page 10: Synopsis – Duration of Study Participants	Added "Pilot subjects will have an optional additional 12 month follow-up period for a second infusion."
Page 10: Synopsis: Definition of Endpoints	Added per Emmes team call to clarify, "…determined per the Investigator's judgment."
Page 11: Synopsis: Definition of Endpoints	Removed "CMV" to match information in the body of main protocol
Page 11: Synopsis – Exclusion criteria	Contradictor statement moved to the inclusion criteria "…with an FSH > 25.8 IU/L" instead of "…must have an FSH < 25.8 IU/L"
Page 37: Section 2.2.1 – Primary Endpoints (Safety)	Added per Emmes team call to clarify, "…determined per the Investigator's judgment."
Page 37: Section 2.2.1 – Primary Endpoints (Safety)	Per the October 3, 2014 team call, Removed "bicarbonate" and replaced with "carbon dioxide", CO2 should be captured rather than bicarbonate.
Page 37: Section 2.2.2 – Secondary Endpoints	Revised last 2 bullet's to match synopsis information
Page 38: Section 3.1 – Description of the Study	Added "In the pilot phase, the fifteen (15) subjects will be able to receive an optional second administration of the study product."
Page 38: Section 3.2 – Randomization Study	Added to second to last paragraph "…clinic…, excluding the baseline visit."
Page 39: Section 4.2 – Exclusion criteria	Contradictor statement revised to read"…with an FSH > 25.8 IU/L" instead of "…must have an FSH < 25.8 IU/L"
Page 42: Section 6.1 – Study Investigational Product	Revised wording to be consistent throughout the protocol with "investigational product" instead of "investigational agent"
Page 43: Section 5.2 - Normal Donor Eligibility	Added the following additional language regarding donor hemoglobin results, "…if male; and if female donor hemoglobin > 11.0 g/dL"
Page 44: Section 6.2 – Dosing	Added additional language for the second infusion, "After subjects complete their Month 12 follow-up phone call visit in the pilot phase, all 15 subjects will then have the option of receiving a second single infusion of allogeneic hMSCs: 1 x 10^8 (100 million) cells delivered via peripheral intravenous infusion. The option will be provided if subjects in the pilot phase continue to meet all inclusion / exclusion criteria's."
Page 44: Section 6.4 – Administration Rate	Per the October 3, 2014 call the following language was added to maintain the blind during the randomized portion of the trial "- During the randomized phase each dose will take place over approximately 40 minutes in order to maintain the blind."

Page 46: Section 6.5.1 – Permitted therapy	Per recent CRO suggestion added "…and dietary supplements."
Page 47: Section 6.6 – Blinding and Unblinding	Revised wording to be consistent throughout the protocol with "investigational product" instead of "investigational agent"
Page 47: Section 6.6 – Blinding and Unblinding	Added specific blinding language for the randomized portion of the trial "A brown plastic slip cover will be placed over the infusion lines as well as the bags to maintain blind."
Page 47: Section 6.7.1 – Investigational Product Labeling and Storage	Revised wording to be consistent throughout the protocol with "investigational product" instead of "investigational agent"
Page 47: Section 7.1 – Schedule of Assessments	On the table the header under the screening column was revised from "-56 – 86 day window" to "-56 day window".
Page 49: Section 7.1 – Schedule of Assessments	Added Table 3 with additional visits for the modification to the protocol to allow for a second injection and follow-up visits for the 15 patients that participated in the pilot phase of the trial.
Page 50: Section 7.1 – Time and Events Key	Added "…calcium…" to renal function tests as it is included in the primary endpoint.
Page 50: Section 7.1 – Time and Events Key	Added "…and TNFα…" as it is included in the primary endpoint.
Page 53: Section 7.2.4 – Week 2 visit	Week 2 visit was separated from section 7.2.5 due to its separate study visit window.
Page 54: Section 7.2.7 – Biomarker Assessment	Added "All fifteen (15) pilot phase subjects will be informed with the option of having a second administration of allogeneic hMSCs at this visit." For addition of second infusion.
Page 56: Section 8.2.1 – Pulse Oximetry	Removed "At Screening, oxygen saturation must be ≥93% to meet inclusion criterion in order to minimize risk of 93% infusional toxicity.
Page 56: Section 8.2.1 – Pulse Oximetry	Added "The infusion will be stopped if the oxygen saturation does not return to >93% within 3 minutes of initiating supplemental oxygen or if the subject requires greater than 2L/min supplemental oxygen to achieve the required saturation of >93%."
Page 63: Section 8.15 – Regulatory Aspects of Adverse Event Reporting	Removed "(from the time of informed consent through 28 days after study completion)"
Page 65: Section 9.4.2 – Purpose of the DSMB	Added additional language to coincide with DSMB charter "review the accumulating unblinded safety data from each study group and using the data as the basis for recommendations concerning the continuation and/or modification of the study." And removed "provide unblinded assessments of patient safety throughout the study."
Page 66: Section 9.4.2.1 – DSMB membership	Revised and added the following language to match DSMB charter, "members (inclusive of the DSMB chair). who The members include a clinical trialist, a biostatistician, an expert gerontologist and an expert cardiologist."
Page 67: Section 9.4.2.3 – DSMB responsibilities	Added bullet #6 to match DSMB charter, "Evaluate the conduct of the study including enrollment rates, the selection and retention of patients, protocol deviations, treatment adherence and quality and completeness of the data"
Page 69: Section 9.4.3.3 – Data Review Meetings	Revised language in first paragraph to match DSMB charter.
Page 79: Addendum A	Added new section for the addition of second infusion option for pilot patients.

Change Document CRATUS Protocol
From March 4, 2015, Version 4 to May 22, 2015 Version 4.1

Location in Document	Details of Change
Cover Page	Changed the date from March 4, 2015, Version 4 to May 22, 2015, Version 4.1
All Pages	Changed header to reflect new version date
Addendum A	Added reference numbers for the sections
Page 10: Synopsis – Definition of endpoints	Added "…and SPPB assessment)" to the second bullet and removed from the forth bullet.
Page 11: Synopsis – Inclusion Criteria	Moved from the exclusion to inclusion, to allow inclusion of subjects with known or unknown allergies the following criteria, "Have known allergies to penicillin or streptomycin."
Page 11: Synopsis – Exclusion Criteria	Removed "Have known allergies to penicillin or streptomycin."
Page 39: Section 4.1 – Inclusion criteria	Moved from the exclusion to inclusion, to allow inclusion of subjects with known or unknown allergies the following criteria, "Have known allergies to penicillin or streptomycin."
Page 40: Section 4.2 – Exclusion criteria	Removed "Have known allergies to penicillin or streptomycin."
Page 41: Section 5.1 – Bone marrow aspiration for generation of MSCs	Added additional information after fifth sentence:<br>"The MNCs will be prepared antibiotic free (ie. No penicillin or streptomycin)." And revised in now the seventh sentence "…225…" changed to "…175…"
Page 41: Section 5.2 – Normal Donor Eligibility	Reduced donor age range from "20" to "18" to match NDMP
Page 43: Section 6.1 – Study Investigational Product	Added the following additional language regarding penicillin free IP for subjects:<br><br>Seventh sentence in the paragraph, "The MNCs will be prepared antibiotic free (ie. No penicillin or streptomycin)."<br><br>End of paragraph "This process does not contain penicillin and/or streptomycin. Any subject that test positive for a penicillin or streptomycin allergy will be provided with an antibiotic free MSC product. The existing product prepared with antibiotic and stored under this BB-IND #15679 will be used only for subjects who are non-allergic to penicillin or streptomycin until all MSCs are depleted."
Page 49: Section 7.1 – Time and Events Table	Additional laboratory assessments are being added "…testosterone (males only) and estrogen (females only)."
Page 73: Section 10.3 – Subject Informed Consent	Added additional language for Reconsenting, "In cases where a new ICF is issued between the Month 6 (Office Visit) and Month 12 (Telephone visit) the subject will be contacted and informed of the changes. If the subject will be asked if they are available to come to the site. If the subject in unable to come to the site the subject may be reconsented via phone."
Page 80: Section A.3.2.2 – Secondary endpoints (Efficacy)	Removed from "…and SPPB assessment)" from the fourth bullet and added to the second bullet.
Page 81: Section A.4.2 – Inclusion criteria for follow-on phase	Moved from the exclusion to inclusion, to allow inclusion of subjects with known or unknown allergies the following criteria, "Have known allergies to penicillin or streptomycin."

Page 81: Section A.4.1 – Inclusion criteria for Follow-on Phase	Removed "• Show signs of frailty apart from a concomitant condition as assessed by the Investigator with a frailty score of 4 to 7 using the Canadian Clinical Frailty Scale"
Page 82: Section A.4.2 – Inclusion criteria for follow-on phase	Removed "Have known allergies to penicillin or streptomycin."
Page 84: Addendum A – Payments to subjects in the Follow-on Phase	Clerical error missed Month 1 and Month 3 visit payments, which subject receives "$25.00" for that visit. Total remuneration as a result was revised to read "$75" instead of "$25".
Page 85: Section A.14 – Pilot phase optional second infusion schedule	Added questionnaires and urinalysis to Month 1 inadvertently not added to previous version.
Page 85: Section A.14 – Pilot phase optional second infusion schedule	Removed Optional Brachial Ultrasound and Endothelial blood samples from the Month 6 visit
Page 86: Section A.14 – Time and Events Table Key	Added "• Laboratory work may be completed at a diagnostic center or home health agency should the subject be unable to come to the site."
Page 86: Section A.14 – Time and Events Table Key	Additional laboratory assessments are being added "…testosterone (males only) and estrogen (females only)."
Page 88: Section A.15.4 – Month1	Removed week 4 and added Month 1 to match the study procedure table.

Change Document CRATUS Protocol
From May 22, 2015, Version 4.1 to June 30, 2015 Version 4.2

Location in Document	Details of Change
Cover Page	Changed the date from May 22, 2015, Version 4.1 to June 30, 2015 Version 4.2
All Pages	Changed header to reflect new version date
Page 8: List of Abbreviations	Added "LFT – Liver Function tests" and "INR – International Normalized Ratio"
Page 9: Synopsis - Investigational Plan	Increased enrollment amount "…up to forty-five (45) subjects in the randomized phase to include up to 15 more additional subjects.
Page 10: Synopsis - Investigational Plan	Revised to increase to "…up to 45 subjects…"
Page 10: Synopsis – Treatment strategies	Group A, B, and C were revised to increase number of subjects from "10" to "15"
Page 10: Synopsis – Subject Population	Revised to "Up to sixty (60)…"
Page 36: Section 3.2 – Randomized Study	Second paragraph revised as follows for the increase in enrollment, "…forty-five (45)… total up to sixty (60) subjects."
Page 56: Section 8.2.4 – Subject stopping guidelines	Corrected spelling error in sentence 3 of the first paragraph

Change Document CRATUS Protocol

From June 30, 2015 Version 4.2 to August 28, 2015 version 4.3

Location in Document	Details of Change
Cover Page	Changed the date from June 30, 2015 Version 4.2 to August 28, 2015 version 4.3
All Pages	Changed header to reflect new version date
Page 2: Table of Contents	Updated table of contents
Page 10: Synopsis – Investigational Plan	Removed “up to forty-five (45) subjects” and replaced with “thirty (30)”
Page 11: Synopsis – Investigational Plan	Group A, B, and C were revised to reduce the number of subjects back from “15” to “10”
Page 11: Synopsis – Subject population	Removed “up to forty-five (45) subjects” and replaced with “thirty (30)”
Page 11: Synopsis – Subject population	Added “Only 45 subjects will be included in the statistical analysis plan.”
Page 12: Synopsis – Inclusion criteria	Removed “Have known allergies to penicillin or streptomycin.” From the inclusion criteria as subjects with or without penicillin allergies can participate in the trial.
Page 12: Synopsis – Inclusion criteria	Corrected units for FSH and added “, if not currently on hormone replacement therapy.”
Page 39: Section 3.2 – Randomization Study	Removed “up to forty-five (45) subjects” and replaced with “thirty (30)”
Page 40: Section 4.1 – Inclusion criteria	Removed “Have known allergies to penicillin or streptomycin.” From the inclusion criteria as subjects with or without penicillin allergies can participate in the trial.
Page 40: Section 4.1 – Inclusion criteria	Corrected units for FSH and added “, if not currently on hormone replacement therapy.”
Page 44: Section 5.5 – Biomarker Assessment	Removal of gene expression language need to verify language with lab:<br><br>“A separate 7 mL blood sample for gene expression profiling of WBC RNA will be obtained at the donation visit.” “…gene expression or…”
Page 45: Section 6.2 – Dosing	Administrative error listed “15” instead of “30” subjects.
Page 49: Section 7.1 – Time and Events Schedule	Addition of 2 new questionnaires – “subject global assessment and physician global assessment” to Month 6 of the time and events table
Page 54: Section 7.2.8 – Biomarker assessment	Removal of gene expression language need to verify language with lab:<br><br>“A separate 7 mL blood sample for gene expression profiling of WBC RNA will be obtained at the donation visit.” “…gene expression or…”
Page 56: Section 8.2.2 - Pregnancy	Corrected units for FSH
Page 81: Section A.4.1 – Inclusion criteria	Removed “Have known allergies to penicillin or streptomycin.” From the inclusion criteria as subjects with or without penicillin allergies can participate in the trial.
Page 85: Section A.14 – Table 3, Visit schedule	Addition of physician global assessment at Month 6
Page 85: Section A.14 – Table 3, Visit schedule	Administrative error – Screening window for the second infusion should be 6 months not 3 months as is listed in A.15.1
Page 91: Addendum B	Added new addendum to study penicillin / streptomycin free cell safety

CRATUS Protocol
Change Document

Location in Document	Details of Change
Cover Page	Changed the date from August 28, 2015 version 4.3 to December 21, 2015 version 4.4
All Pages	Changed header to reflect new version date
Page 12: Synopsis – exclusion criteria	Administrative error replaced “injection” with “infusion”
Page 41: Section 4.2 – exclusion criteria	Administrative error replaced “injection” with “infusion”
Page 43: Section 5.2 – Normal Donor Eligibility	Added “…urine tests” and “Urinalysis” to tests prospective donors will have.
Page 50: Section 7.1 – Time and Events Table	Bullet items #5 and #6 on the time and events key were reversed as “Optional brachial ultrasound to assess endothelial function” applies to the optional brachial ultrasound and “Optional: An additional 5 lavender tubes (EDTA) will be drawn” applies to the endothelial blood samples procedure on the Time and Events table on page 49 of the protocol.
Page 60: Section 8.7: Definition of Unexpected	Removed language as there is no investigator brochure “…in the investigator brochure or is not listed…”
Page 86: Section A.14 – Time and Events Table	Bullet items #5 and #6 on the time and events key were reversed as “Optional brachial ultrasound to assess endothelial function” applies to the optional brachial ultrasound and “Optional: An additional 5 lavender tubes (EDTA) will be drawn” applies to the endothelial blood samples procedure on the Time and Events table on page 49 of the protocol.
Page 97: Section B.13: Time and Events schedule	Original Language:<br><br>There will be up to a 21 day window from the time the subject signs the informed consent form to the infusion.<br><br>Revised Language:<br><br>There will be up to a 45 day window from the time the subject signs the informed consent form to the baseline visit to complete screening procedures.
Page 97: Section B.13: Time and Events schedule	Removed Subject global assessment as they are not applicable as Subjects will be aware of the dose they are receiving.
Page 98: Section B.13 – Time and Events Table	Bullet items #5 and #6 on the time and events key were reversed as “Optional brachial ultrasound to assess endothelial function” applies to the optional brachial ultrasound and “Optional: An additional 5 lavender tubes (EDTA) will be drawn” applies to the endothelial blood samples procedure on the Time and Events table on page 49 of the protocol.
Page 99: Section B.14.1 – Screening visit	Removed “21” and replaced with “45” as the window. Additionally infusion was removed and language added to clarify the screening window is 30 days till baseline And replacing with “…baseline visit to complete screening procedures.”

CRATUS Protocol
Change Document

Location in Document	Details of Change
Cover Page	Changed the date from December 21, 2015 version 4.4 to June 14, 2016 Version 5
All Pages	Changed header to reflect new version date
Page 45: Section 6.2 – Dosing	Corrected to read that "15 subjects" not "30 subjects" will be in the pilot phase.
Page 45: Section 6.2 – Dosing	Corrected to read that "30 subjects" not "15 subjects" will be in the randomized phase.
Page 51: Time events table and key	Added bullet #9 as the form is not applicable to pilot subjects, "Subject Global Assessments are not applicable to pilot subjects in this phase of the trial."
Page 80: Section A.2	Corrected from "immun**e**privileged" to "immun**o**privileged"
Page 101: Section B.14.3 – Day 1 Visit	Language revised to clarify that "4 – 6" revised to read "2 – 4" hours that a subject will be monitored on Day 1.
Page 104 – 115: Addendum C	Addition of Addendum C to give additional infusions for subjects in the pilot and penicillin/streptomycin free subjects

CRATUS Protocol
Change Document

Location in Document	Details of Change
Cover Page	Changed the date from June 14, 2016 Version 5 to August 1, 2016 Version 5.1
All Pages	Changed header to reflect new version date
Page 11 - Synopsis of Pilot Phase	Changed from “a second administration” to “additional administrations”
Page 12 – Synopsis of Randomized Phase	Changed “In the randomized phase of allo-hMSCs or matched placebo” from “up to 45 subjects” to “up to 30 subjects"
Page 18 – Preclinical Experience	Changed Figure 1 to correctly reflect referenced image
Page 19 – Preclinical Experience	Changed Figure 2 to correctly reflect referenced image
Page 20 – Preclinical Experience	Changed Figure 3 to correctly reflect referenced image
Page 39 – 3.1 Description of Study	Changed “receive an optional second additional administration” to “receive additional administrations”
Page 40 – 3.2 Randomization Study	Added “1 x 10^8 (100 Million)” to In the randomized phase of the trial, subjects which received placebo will have the option to receive 1 x 10^8 (100 Million) allogeneic hMSCs, if all study endpoints are met.
Page 42 – 5. MSC Donors	Added Zika Virus to allogeneic donor testing paragraph.
Page 42 – 5.2 Normal Donor Eligibility	Removed portion “A maximum of 15 subjects” from Donor Eligibility
Page 43 – 5.2 Normal Donor Eligibility	Added Zika Virus to infectious disease testing portion and Eligibility Criteria.
Page 45 – 6.2 Dosing	Removed portion “approximately 15 normal…” donors
Page 54 – 7.2.6 Month 12	Changed from “a second administration” to “additional administrations”
Page 82 – A.5 Dosing	Changed “subjects will then have the option of receiving a second single additional infusions” to “subjects will then have the option of receiving additional infusions”
Page 83 – A.5 Dosing	Changed “derived from approximately 2-3 normal donors” to “derived from donors”
Page 85 – Table 3	Removed physician global assessment from the table as it is only applicable to subjects in the randomized phase of the trial.
Page 91 – B.1 Rationale & Description for PCN/Streptomycin Free	Changed from “Up to fifteen subjects” to “Up to twenty subjects”
Page 95 – B.4 Dosing	Changed “derived from approximately 2-3 normal donors” to “derived from donors”
Page 98 – Table B3	Removed physician global assessment from the table as it is only applicable to subjects in the randomized phase of the trial.
Page 108 – C.5 Dosing	Changed “derived from approximately 5-10 normal donors” to “derived from donors”
Page 111 – Table C3	Removed physician global assessment from the table as it is only applicable to subjects in the randomized phase of the trial.

CRATUS Protocol
Change Document

Location in Document	Details of Change
Cover Page	Changed the date from August 3rd, Version 5.1 to September 20th, Version 5.2
All Pages	Changed header to reflect new version date
Throughout the Protocol	Replaced patient with subject
Throughout the Protocol	Added "…and/or Longeveron, LLC." to become phrase: "…source manufactured by the University of Miami and/or Longeveron, LLC."
Throughout the Protocol	Added "and" to Data and Safety Monitoring Board
Throughout the Protocol	Changed "exercise induced ejection fraction" to "Dobutamine stress echo induced ejection fraction"
Throughout the Protocol	Updated window for M1 Visit to 2 weeks.
Throughout the Protocol	Changed "history of malignancy" from 5 years to 3 years.
Page 9 – List of Abbreviations	Removed "BDI – Monoclonal Antibody Against Human Bladder Carcinoma"<br><br>Added "CPF – Cell Processing Facility"
Page 11 – Protocol Synopsis	Changed "evaluate" to "evaluated"
Page 13 – Protocol Synopsis	Changed from Difference to Decrease in phrase "-Decrease in subject quality of life assessment(s)"
Page 14 –Synopsis: Exclusion Criteria	Added: 14. Any other condition that, in the opinion of the investigator, may compromise the safety or compliance of the subject or preclude successful completion of the study.
Page 21 – Section 1.3 – MSC: Preclinical Experience	Added Figure 5, 6, 7, and 8 to reflect figure references.
Page 22 – Section 1.3 – MSC: Preclinical Experience	Added Figure 9 to page 22 to reflect figure references.
Page 23 – Section 1.3 – MSC: Preclinical Experience	Added Figures 10, 11, and 12 to reflect figure references.
Page 24 – Section 1.3 – MSC: Preclinical Experience	Changed "Figure 21" to "Figure 13" to reflect graphic. Added Figure 13.
Page 37 – Section 2.2.2 – Secondary Endpoints	Made "Exhaustion (assessed via the MFI questionnaire) orphaned under Point 1<br><br>Changed from Difference to Decrease in phrase "-Decrease in subject quality of life assessment(s)"
Page 38 – Section 3.2 – Randomization Study	Added "in the randomized phase" and "In the randomized phase," to clarify phases.
Page 41 – Section 3.2 – Exclusion Criteria	Replaced "every 12 weeks" with "following Day 0" in the phrase: "Efficacy parameters (pulmonary function tests, 6MWT, and QOL questionnaires) will be assessed following Day 0 until study completion."
Page 39 – Section 3.2 – Randomization Study	Replaced "which" with "who"
Page 41 – Section 4.2 – Exclusion Criteria	Changed "Have hypersensitivity to…" to "Hypersensitivity to…"
Page 41 – Section 5.1: BMA for generation of MSCs	Added to first sentence that "approximately 60ml to 120 ml" will be aspirated
Page 42 – Section 5.1: BMA for generation of MSCs	Added additional information regarding what specific type of Zika test will be drawn.
Page 41 – Section 5.2:	Added "inclusive" to phrase "Donors (male or female) between the ages of 18 to

Normal Donor Eligibility	45 (inclusive) will be screened as potential BM donors." to reflect that Donors include 18 and 45 year-olds.
Page 43 – Section 6.1: Study IP	Added to first sentence that "approximately 60mL to 120 mL" will be aspirated
Page 44 – Section 6.3 – Dosage Rationale	Changed maximum dose from 100x10^6 to 200X10^6 to reflect the study maximum.
Page 45 – Section 6.2 - Dosing	Replaced "that" with "who"
Page 47 – Section 6.7.1 & 6.7.2 - Investigational Product Labeling and Storage	Added "and/or Longeveron, LLC. Cell Processing Facility (CPF)" regarding storage and delivery of cells throughout the section and 6.7.2
Page 51 – Section 7.2.2 – Baseline Visit	Replaced "(interleukin 1, interleukin-10, interleukin-6, tumor necrosis factor alpha, high-sensitivity C Reactive Protein, Pro-Brain Natriuretic Peptide, von-Willebrand Factor) and soluble cell adhesion molecules (E-selectin, P-selectin, Inter Cellular Adhesion Molecule 1, Vascular Cell Adhesion Molecule 1), and apoptotic factors (Anexin 5) using immunological and ELISA methods" with (interleukin-1, interleukin-6, interleukin-10, VEGFR2, TNF-a)
Page 52 – Section 7.2.2 – Baseline Visit	Removed "- Flow Cytometry: characterization of EPCs from peripheral blood will be done by Flow Cytometry (FACS). EPCs will be defined as CD34-, Cd19-, CD3-, CD133+/KDR+ cells."
Page 53 – Section 7.2.8 – Immune Monitoring for Graft Rejection	Deleted "Additionally, in female subjects who receive allogeneic hMSCs, the stored baseline serum will be analyzed to evaluate the antibody responses to HLA and H-Y antigens."
Page 55 – Section 8.2.1 – Pulse Oximetry	Removed "Subjects should have a resting oxygen saturation of ≥93% in order to be randomly assigned."
Page 55 – Section 8.2.2 – Pregnancy	Replaced ">40" with "≥60";<br><br>Added "must practice any" and "forms of contraception" to: "Males and females of non-childbearing potential > 60 to < 95 years of age at the time of signing the Informed Consent Form with documented FRAILTY must practice any one of the enumerated forms of contraception. Items will be acceptable for meeting the studies contraceptive requirements as listed in section 8.2.2."
Page 56 – Section 8.2.4 – Subject Stopping Guidelines	Removed "or grade 3 LV perforation" as this study is an infusion.<br><br>Replaced the word "injection" with "infusion"
Page 64 – 9.3 – Interim Analyses	Added "These should not be considered formal interim analyses as no hypothesis testing will be done."
Page 64 – 9.4 – Data and Safety Monitoring Board	Added "and" to Data "and" Safety Monitoring Board.
Page 65 – 9.4.2.3 DSMB Responsibilities	Removed "3. Review Periodic Safety Update Reports (PSURs). "
Page 67 – 9.4.3.2: Review of Periodic Safety Update Reports	Replaced "reported every month" with "furnished" in phrase: "As part of ongoing safety review and obligation to regulatory agencies, safety reports will be furnished."
Page 67 – Section 9.4.3.3: Data review meetings	Corrected sequence of tables to read as table 4 instead of 2
Page 69 – 9.4.3.3 – Closed Session	Removed "within 24 hours" from phrase: "…the DSMB chairperson will issue one of the following recommendations as determined by the DSMB…"<br><br>Added "support team" to Separate meeting minutes for the open and closed

	sessions will be prepared by the DSMB support team, then reviewed and issued by the DSMB chairperson.<br><br>Added the phrase "The DSMB support team will distribute the finalized minutes."
Page 72 – 10.3 – Subject Informed Consent	Changed "If the subject" to "The subject" and replaced "in" with "is" in : If the subject is unable to come to the site the subject may be reconsented via phone.
Page 75 – Appendix 1 – Procedures Post-Infusion	Changed Post-Infusion point 3 to reflect the post infusion observation period of t-3 hours.<br><br>Removed "the following day," from discharge procedure in point 5 as subjects will no longer stay overnight.
Page 78 – A.3.1.2 – Secondary Objectives	Removed point 2 for redundancy: "2.To explore effects of allo-hMSCs on symptom related quality of life, cardiovascular performance, endothelial function and inflammation."
Page 79 – Section A.3..2.2 – Secondary Endpoints	Made "Exhaustion (assessed via the MFI questionnaire) orphaned under Point 1<br><br>Changed from Difference to Decrease in phrase "-Decrease in subject quality of life assessment(s)"
Page 80 – A.3.2.2 – Secondary Endpoints (Efficacy)	Changed "exercise" to "Dobutamine" in sentence: "4. Change between baseline and 6 months in dobutamine induced ejection fraction."
Page 84 – Section A14: Visit Schedule	Revised from table 3 to A1
Page 84 – Table A1 – Pilot Phase Optional 2nd Infusion Schedule	Changed Month 13 Screening from +/- 6 months to +6 months.
Page 86 – Section A.15.1 – Screening Visit for Second Infusion (Applicable to pilot subjects)	Changed phrase from "There will be up to a 6 month window from the subjects Month 12 follow-up visit from the subject's initial infusion" to "There will be up to a 6 month window from the subjects Month 12 follow-up visit to the subject's additional infusion."
Page 87 – Section A.15.2 – Baseline Visit n-10, VEGFR2, TNF-a)	Replaced "(interleukin 1, interleukin-10, interleukin-6, tumor necrosis factor alpha, high-sensitivity C Reactive Protein, Pro-Brain Natriuretic Peptide, von-Willebrand Factor) and soluble cell adhesion molecules (E-selectin, P-selectin, Inter Cellular Adhesion Molecule 1, Vascular Cell Adhesion Molecule 1), and apoptotic factors (Anexin 5) using immunological and ELISA methods" with (interleukin-1, interleukin-6, interleukin—10, VEGFR2, TNF-a)
Page 87 – Section A.15.2 – Baseline Visit	Removed "- Flow Cytometry: characterization of EPCs from peripheral blood will be done by Flow Cytometry (FACS). EPCs will be defined as CD34-, Cd19-, CD3-, CD133+/KDR+ cells."
Page 87 – Section A.15.3 – Day 1	Updated length of monitoring to 2 – 3 Hours
Page 87 – Section A.15.4 – M1 Visit	Updated Window from +/- 7 days to +/- 2 weeks.
Page 88 – A17: Safety Monitoring	Corrected sequence of tables to read as table A2 instead of 4
Page 88 – A17: Safety Monitoring	Corrected sequence of tables to read as table A3 instead of 5
Page 90 – B.2.1.2 – Secondary Objectives	Removed "3. To explore effects of penicillin/streptomycin free allo-hMSCs on symptom related quality of life, cardiovascular performance, endothelial function and inflammation."
Page 91 – Section B.2.2.2 – Secondary Endpoints	Made "Exhaustion (assessed via the MFI questionnaire) orphaned under Point 1

	Changed from Difference to Decrease in phrase "-Decrease in subject quality of life assessment(s)"
Page 96 – Section B14: Visit Schedule	Revised from table B3 to B1
Page 99 – B16: Safety Monitoring	Corrected sequence of tables to read as table B2 instead of 4B
Page 100 – B16: Safety Monitoring	Corrected sequence of tables to read as table B3 instead of 5B
Page 101 – C1: Rationale	Last sentence in first paragraph revised to increase from 15 to 20 subjects in the penicillin/streptomycin free cohort.
Page 103 – C.3.1.2 – Secondary Objectives	Removed "2. To explore effects of penicillin/streptomycin free allo-hMSCs on symptom related quality of life, cardiovascular performance, endothelial function and inflammation."<br><br>Made "Exhaustion (assessed via the MFI questionnaire)" orphaned under Point 1<br><br>Changed from Difference to Decrease in phrase "-Decrease in subject quality of life assessment(s)"
Page 108 – Section C14: Visit Schedule	Corrected sequence of tables to read as table C1 instead of C3
Page 109 – Table C1 – Pilot Phase Additional Infusion Schedule	Changed Month 13 Screening from +/- 6 months to +6 months.
Page 112 – Section C.15.1: Screening	Revised to removed 15 pilot subjects as addendum C will include subjects from both the pilot and penicillin/streptomycin free cohorts.
Page 114 – Section C16: Statistical Considerations	Removed from the second sentence "a second infusion" as subjects will have the option to receive additional infusions.

CRATUS Protocol
Change Document

Location in Document	Details of Change
Cover Page	September 20th, Version 5.2 to November 30, 2016, Version 5.3
All Pages	Changed header to reflect new version date
Page 12: Synopsis – Investigational Plan	Removed "…and/or Longeveron, LLC." as a production facility
Page 44: Section 6.2 - Dosing	Removed "…and/or Longeveron, LLC." as a production facility
Page 47: Section 6.7.1 - Investigational Product Labeling and Storage	Removed "and/or Longeveron, LLC." regarding storage and delivery of cells
Page 81: Section A.5 – Dosing	Removed "…and/or Longeveron, LLC." as a production facility
Page 93: Section B.4 – Dosing	Removed "…and/or Longeveron, LLC." as a production facility
Page 106: Section C.5 – Dosing	Removed "…and/or Longeveron, LLC." as a production facility
