# Supplementary material for: Allogeneic Mesenchymal Stem Cells Ameliorate Aging Frailty: A Phase II Randomized, Double-Blind, Placebo-Controlled Clinical Trial
Source: J Gerontol A Biol Sci Med Sci. 2017 Jul 17;72(11):1513–22. doi: 10.1093/gerona/glx137 (PMC5861900; doi:10.1093/gerona/glx137)
Supplement: Supplementary Material 3 [file glx137_suppl_supplementary_material_3.pdf]

A Phase I/II, Randomized, Blinded, and Placebo-controlled Trial to Evaluate the Safety and Potential Efficacy of Allogeneic Human Mesenchymal Stem Cell Infusion in Participants with Aging Frailty

Allogenei*C* Human Mesenchymal Stem Cells (hMSC) in Participants with Aging F*RA*il*T*y via Intraveno*US* Delivery (CRATUS)

January 20, 2016

CRATUS Randomized Phase Statistical Analysis Plan

Version 1.0

Prepared by

The Emmes Corporation
401 N. Washington Street
7th Floor
Rockville, MD 20850

Authors of Randomized Phase Statistical Analysis Plan

Jill El-Khorazaty, MS
Biostatistician
Emmes

Adam Mendizabal, PhD
Senior Biostatistician
Emmes

Reviewed by:

Joshua Hare, MD
Interdisciplinary Stem Cell Institute
University of Miami Miller School of Medicine

1 Administrative Structure

This study is sponsored by the Interdisciplinary Stem Cell Institute at the University of Miami Miller School of Medicine (The study sponsor is Dr. Joshua M. Hare). The Emmes Corporation (Rockville, MD) serves as the Contract Research Organization (CRO). This analysis plan describes the analysis that is planned for the randomized phase of the study.

1.1 Data Quality Assurance

Data will be collected using the Emmes AdvantageEDC system which includes built in quality control measures. Emmes quality control procedures will be implemented including monthly review of case report form completion and data quality queries.

2 Objectives

Primary Objective

To demonstrate the safety of allogeneic hMSCs administered in participants with frailty.

Secondary Objective

To explore effects of allo-hMSCs on measures of quality of life, cardiovascular performance, functional capacity, muscle strength, bone density, immune-tolerance and inflammation.

3 Investigational Plan

3.1 Overall Study Design and Plan

This Phase I/II, randomized, blinded, placebo-controlled study is designed to evaluate the safety and tolerability of allo-hMSCs in participants with Frailty and to explore potential efficacy at baseline, 3, and 6 months.

Fifteen (15) participants will be enrolled in the pilot phase, followed by thirty (30) participants enrolled in a phase II placebo controlled phase for a total of forty-five (45) participants. In the placebo phase, participants will be randomized 1:1:1 to two active arms of 100 or 200 M cells or placebo. Additional participants may be enrolled if deemed appropriate.

Eligible participants must have a diagnosis or symptoms of frailty as defined by the Canadian Study on Health & Aging[2]. Following informed consent before or at the screening visit, the diagnosis of FRAILTY will be confirmed by investigator review of medical history.

In the randomized phase of the trial, electronic randomization will be performed using the Advantage EDC system and communicated to cellular laboratory personnel who have no contact with the investigators or participants. At the time of administration, opaque tubing will be used to maintain double blinding. Treatments will be administered once and will consist of 1 x 10^8allo-hMSCs (100 million cells), 2 x 10^8 allo-hMSCs (200 million cells), or placebo. After each infusion, participants will be monitored for immediate complications.

Continued safety and tolerability with review of adverse events (AEs) will be monitored at each visit. Efficacy parameters (pulmonary function tests, 6MWT, and QOL questionnaires) will be assessed every 12 weeks until study completion. Clinical laboratory tests to assess safety will be performed at every visit.

In the pilot phase, the fifteen (15) participants will be able to receive an optional second administration of the study product.

In the randomized phase of the trial, participants who received placebo will have the option to receive allogeneic hMSCs, once all study endpoints are met. If all endpoints are met then the participant will be administered the study drug and follow the study schedule from Day 1 to Month 12 after receiving the infusion of allogeneic hMSCs.

Table 1: Overall Schedule of Time and Events

VISIT	Screening Day -56 ± 28 days	Baseline (-4 weeks)	Day 1	Week 2 (Day 14) +/- 2 days	Month 1 (Day 30) (+/-2 weeks)	Month 3 (Day 90) (+/-2 weeks)	Month 6 (Day 180) (+/-2 weeks)	Month 12 (Day 365) (+/-2 weeks) *Phone Call Follow-up
Informed Consent	x							
Full Medical History	x							
Physical Exam	x	x	x	x	x	x	x	
12-lead (ECG)	x	x	x	x	x	x	x	
Concomitant Medications	x	x	x	x	x	x	x	x
Mini Mental State Examination (MMSE)	x						x	
Randomization		x						
Infusion Treatment (IP)			x					
Dobutamine Stress Echo Test (DSE)	x						x	
Bone Density Scan (DEXA)[8]		x					x	
FEV-1		x				x	x	
6 Minute Walk Test		x				x	x	
4 Meter Gait Speed Test[7]		x				x	x	
SPPB Assessment		x				x	x	
Dynamometer (handgrip)		x				x	x	
Smell Identification Test (UPSIT)		x				x	x	
IIEF, SQOL-F Questionnaires		x			x	x	x	
QOL Questionnaires (ICECAP, EQ-5D, SF-36,CHAMPS, MFI)		x			x	x	x	
Urinalysis	x				x	x	x	
Hemat., Chem., CBC, LFTs, INR, and other labs[1]	x		x	x	x	x	x	
HIV 1, HIV 2, Hep. B & C, and CMV	x							
Serum or Urine Pregnancy Test[2]	x		x					
Donor Screening Tests	x							
Review Adverse Events			x	x	x	x	x	x
Immune Monitoring[4]			x	x	x	x	x	
Biomarker Assessment[3]			x				x	
Optional: Brachial Ultrasound [5]		x				x		
Optional: Endothelial blood samples[6]		x				x		

Time and Events Table Key:

1 - The minimal laboratory requirements for hematological, liver function and renal function include:

> **Hematology Tests**: white blood cell count, platelet count, hemoglobin and hematocrit.
>
> **Liver Function Tests**: Albumin, alkaline phosphatase, alanine transaminase, asparate aminotransferase, prothrombin time / activated partial thromboplastin time, and bilirubin(fractionate if total >1.5 times normal).
>
> **Renal Function Tests**: creatinine, creatinine clearance, blood urea nitrogen (BUN), glomerular filtration rate, sodium, potassium, chloride, carbon dioxide, and glucose.
>
> **Serum Uric Acid, Pro-BNP, and C-reactive protein (CRP), IL6, fibrinogen, D-Dimer,**

2 - A serum or urine pregnancy test will be completed within 36 hours prior to infusion for females of childbearing potential.

3 - The following biomarkers will be analyzed:

- **Cell-surface markers**: CXCR4, C-Kit, & Connexin 43
- **Transcriptomic/Proteome:** RNA, miRNA, protein samples, and telomerase, akt
- **Growth factors**: Sdf-1, notch,
- **Functional Assays:** cell growth rate, VEGF, and CFU assay

4 - Immune monitoring for graft rejection. The following markers will be used for analysis to assess for activated T-cells based upon a $CD3^{+}CD25^{+}$ or $CD3^{+}CD69^{+}$ phenotype:

- CD3, CD25, CD69

5 - Optional: An additional 5 lavender top tubes (EDTA) will be drawn.
6 - Optional brachial ultrasound to assess endothelial function.
7 – 4 meter gait speed test will be performed twice per visit and the average of the exams will be taken.
8 – DEXA scan will be performed twice at each visit. The first scan will be of the hip and spine for bone density and the second will be to assess the total body composition.

3.2 Study Endpoints

Primary Endpoint (Safety)

Incidence (at one month post infusion) of any treatment-emergent serious adverse events (TE-SAEs), defined as the composite of: death, non-fatal pulmonary embolism, stroke, hospitalization for worsening dyspnea and clinically significant laboratory test abnormalities.

- Serum chemistry: chloride, bicarbonate, BUN, creatinine, glucose, calcium, AST/SGOT, ALT/SGPT, total bilirubin (fractionate if total >1.5 times normal), alkaline phosphatase, albumin
- Hematology (CBC): hemoglobin, hematocrit, platelets, WBC, WBC differential

Secondary Endpoints (Efficacy)

The following efficacy endpoints will be evaluated in this trial (during baseline, 3 and 6 months follow-up visits):

- Difference in rate of decline of Frailty defined as:
  - Reduced Activity (assessed via CHAMPS questionnaire)
  - Slowing of Mobility (assessed via a 4 meter gait speed test and SPPB assessment)
  - Weight Loss
  - Diminished handgrip strength (assessed via dynamometer)
  -
- Exhaustion (assessed via the MFI questionnaire)
- Difference in participant quality of life assessment(s): (ICECAP, EQ-5D, SF-36)
- Death from any cause.
- Change between baseline and 6 months in exercise ejection fraction
- Change between baseline and 6 months for the following panel of inflammatory markers: CRP, IL-6, D-dimer, fibrinogen, CBC with differential, DNA , and TNFα

3.3 Study Population

Eligible participants must have had a diagnosis or symptoms of frailty as defined by the Canadian Study on Health & Aging. Following informed consent before or at the screening visit, the diagnosis of frailty will be confirmed by investigator review of medical history.

3.4 Duration of Participation

Participant participation duration is approximately 12 months.

3.5 Randomization, Stratification, and Blinding

The pilot phase portion of this study is unblinded; participants are enrolled into each group consecutively. No randomization or stratification is involved.

The randomized phase of this study will randomize participants in a 1:1:1 ratio to one of two doses of MSCs versus placebo. No stratification factors will be used. Permuted block randomization may lead to a small imbalance if the block at the end of randomization is not completed.

Only designated technicians in the ISCI Cell Processing Laboratory will be unblinded to treatment. The investigator, study staff, participant, and anyone involved in the care of the participant will not be made aware of the assigned treatment regimen. The designated cell-processing technicians will prepare both the allogeneic hMSCs infusions. The investigational agent infusions will be prepared in identical infusion bags and labeled with the identical investigational drug labels. The designated technicians in the ISCI Cell Processing Laboratory (or designee) will be responsible for maintaining the investigational agent records including randomized treatment assignments by participant identification.

If for important medical reasons unblinding is thought to be necessary, the investigator may identify the treatment assignment by obtaining the randomization assignment by contacting the Director of Experimental and Clinical Cell Based Therapies at ISCI, who is responsible for maintaining randomization records for all participants.

4 Study Populations

4.1 Eligibility Criteria

4.1.1 Participant Inclusion Criteria

1. Provide written informed consent.
2. Participants age $\geq$60 and $\leq$95 years at the time of signing the Informed Consent Form.
3. Show signs of frailty apart from a concomitant condition as assessed by the Investigator with a frailty score of 4 to 7 using the Canadian Clinical Frailty Scale

4.1.2 Participant Exclusion Criteria

1. Score of $\leq$24 on the Mini Mental State Examination (MMSE)
2. Inability to perform any of the assessments required for endpoint analysis (report safety or tolerability concerns, perform PFTs, undergo blood draws, read and respond to questionnaires)
3. Active listing (or expected future listing) for transplant of any organ.
4. Clinically important abnormal screening laboratory values, including but not limited to: hemoglobin <8 g/dl, white blood cell count <3000/mm^3, platelets<80,000/mm^3, INR > 1.5 not due to a reversible cause (i.e. Coumadin), aspartate transaminase, alanine transaminase, or alkaline phosphatase > 3 times upper limit of normal, total bilirubin > 1.5 mg/dl.
5. Serious comorbid illness that, in the opinion of the investigator, may compromise the safety or compliance of the participant or preclude successful completion of the study. Including, but not limited to: HIV, advanced liver or renal failure, class III/IV congestive heart failure, myocardial infarction, unstable angina, or cardiac revascularization within the last six months, or severe obstructive ventilatory defect.

6. Any other condition that, in the opinion of the investigator, may compromise the safety or compliance of the participant or preclude successful completion of the study.
7. Have known allergies to penicillin or streptomyocin.
8. Be an organ transplant recipient.
9. Have a clinical history of malignancy within 5 years (i.e., participants with prior malignancy must be disease free for 5 years), except curatively-treated basal cell carcinoma, squamous cell carcinoma, melanoma in situ or cervical carcinoma if recurrence occurs.
10. Have a non-pulmonary condition that limits lifespan to < 1 year.
11. Have a history of drug or alcohol abuse within the past 24 months.
12. Be serum positive for HIV, hepatitis BsAg or Viremic hepatitis C.
13. Be currently participating (or participated within the previous 30 days) in an investigational therapeutic or device trial.
14. Be a female who is pregnant, nursing, or of childbearing potential while not practicing effective contraceptive methods. Female participants must undergo a blood or urine pregnancy test at screening and within 36 hours prior to infusion.
15. Female participants must have an FSH < 25.8 IU/L
16. Have hypersensitivity to dimethyl sulfoxide (DMSO)

4.2 Analysis Population

The analysis sample will include all participants who received an infusion. Participants who withdraw for reasons unrelated to the study or study drug (e.g. withdrawal of consent or loss to follow-up) may be replaced if deemed necessary to meet study objectives.

4.2.1 Pilot Phase

All 15 participants who received the study treatment will be included in the pilot phase analyses. Analyses will focus on estimation and therefore no comparisons between groups in the pilot phase are planned. Secondary exploratory analyses will be conducted collapsing all three doses levels as well as comparing outcomes between treatment groups. The pilot phase will be analyzed separately.

4.2.2 Randomized Phase

All 30 participants who received the study treatment will be included in the randomized phase analyses. Treatment group comparisons will be made between the three arms in the randomized portion of the study. If significant, further hypothesis testing to determine which treatments arms differ will be completed.

5 Statistical Considerations

All statistical tests will be performed at an alpha=0.05 level of significance, using two-sided tests, unless otherwise noted. Because of the early phase nature of this study, no adjustments will be made for multiple analyses. Categorical variables will be summarized using counts (n) and percents (%). Baseline continuous data will be summarized in tables listing the mean and standard deviation (SD). Data which are highly skewed will be reported using the median, 25th and 75th percentiles, the minimum and maximum values, and the number of participants with data. Confidence intervals will be presented where appropriate. Two decimal places will be used for all numbers except p-values which will be resolved to 4 decimal places.

A mixed model for repeated measures will be used to compare treatment groups where outcomes are collected at baseline and follow-up visits. The model will include a dose*time interaction. If there are no statistically significant differences by dose, then the doses may be collapsed to determine the overall effect. Spaghetti plots will be used to present the data visually. Dynamite plots will be generated to show the change from baseline by dose.

Analysis of AEs will include tabulation by frequency, severity, organ system affected, and relationship to study exposure. Participant reported outcome data will be summarized according to the guidelines of each questionnaire.

All available data will be included in data listings and tabulations.

If the amount of missing data is substantial, imputation methods may be used.

5.1 Sample Size

No formal statistical justification was performed to determine sample size in the Phase I study. Cohort size was determined based on expected requirements for safety analyses and projected enrollment rates.

5.2 Study Hypothesis

The primary study hypothesis is that allogeneic MSC administration is safe in participants with frailty.

5.3 Interim Analysis for Safety and Efficacy

After all participants enrolled in the pilot phase have received the study therapy infusion and have been followed for 30 days, the DSMB will conduct a full review of all cumulative safety data before the trial proceeds to the randomized phase. As part of the cumulative safety data review meeting for the pilot phase, the DSMB will recommend that the trial proceed to the protocol-specified randomized phase or recommend a dose modification for the randomized placebo study.

Formal data review meetings that include the entire DSMB will be conducted via teleconference approximately every three months. The timeline for the quarterly DSMB data review meetings will begin after approximately 25% of participants are enrolled in the randomized phase. Meetings may be postponed due to accrual rates at the DSMB’s discretion. The purpose of the data review is for safety evaluation, and the study may be stopped because of significant safety concerns. SAEs which are related to stopping rules will be continuously evaluated and the full DSMB will be informed of any extra risk.

Other safety data available at each evaluation, such as 12-Lead ECGs and laboratory daa will also be evaluated by the DSMB as appropriate.

5.4 Final Analyses and Reporting

Final safety and efficacy analyses will be conducted on participants in the randomized phase of the study once all thirty (30) participants have been followed for 6 months. Analyses will be

conducted again once all participants have been followed for 12 months. No adjustments for multiple comparisons will be made due to the exploratory nature of this phase.

5.5 Visit Windows

Study product will be administered on Day 1. Visit windows as specified in the protocol are as follows:

Day 1: No visit window
Week 2 (Day 14): +/- 2 days
Month 1 (Day 30): +/- 2 weeks
Month 3 (Day 90): +/- 2 weeks
Month 6 (Day 180): +/- 2 weeks
Month 12 (Day 365): +/- 2 weeks

5.6 Changes in the Planned Analysis

Changes in the planned analysis will be documented in this section.

6 Description of Study Participants

6.1 Participant Disposition

The disposition of participants will be summarized to include the number of participants achieving study milestones. A flow chart will be created according to the CONSORT statement showing the disposition of the participants considered on the study.

6.2 Demographic Data

Demographics and baseline characteristics will be summarized for all participants. Characteristics to be examined are: age at infusion, gender, race/ethnicity, ejection fraction at screening, six-minute walk test performance, peak VO2, medical history, and CT/MRI values at screening.

6.3 Medical History

Baseline medical history will be described as part of the baseline characteristics table with data obtained from the Medical History form.

6.4 Inclusion and Exclusion Criteria

Data used for the inclusion and exclusion criteria will be presented in listing format only.

6.5 Protocol Deviations

Study protocol deviations will be presented in a listing.

6.6 Allogeneic Cell Processing

The donor number, total nucleated cells, trypan blue viability, gram stain status, microbiology (growth/no growth) and endotoxin concentration will be described.

7 Treatments and Medications

7.1 Concomitant Medications

A listing of concomitant medications will be provided with start and stop dates, where available.

7.2 Study Infusion Compliance

Compliance in receiving the number of infusions and dose will be provided. Deviations from the prescribed regimen will be detailed.

8 Randomized Phase Analyses

Analyses in the randomized phase will focus on comparing each dose level to placebo as well as within dose level comparisons. In general, binomial proportions and 95% confidence intervals will be used. For time-to-event data Kaplan-Meier event rates will be presented. For events with competing risks, cumulative incidence will be presented treating death as a competing event. Repeated measures analyses will be considered for data that are collected over time. Dose will be considered as an effect as well as a dose by time interaction. Within dose level effects will be described and model estimated within-group tests will be performed. Results of the repeated measures models need to be interpreted with caution due to the small sample size in this early phase study.

Data that are non-normal will be analyzed using rank analysis of covariance at each follow-up time point adjusting for baseline. Within-group tests for non-normal data will be computed using Wilcoxon signed rank test.

The following parameters will be examined.

Safety:
- Death
- Hospitalizations
- Non-fatal pulmonary embolism
- Stroke
- Clinical laboratory evaluations
- Hematology
- Serum chemistry
- Urinalysis
- Protein reactive antibodies
- Inflammatory markers
- 12-lead electrocardiogram

Efficacy:
- 4-meter gait speed test

- Six minute walk test
- Short Physical Performance Battery (SPPB)
- FEV1
- Smell identification test
- Recipient weight
- Handgrip strength test
- CHAMPS Questionnaire
- ICECAP-O Questionnaire
- EQ-5D
- SF-36
- Multidimensional Fatigue Inventory (MFI) Questionnaire
- Mini mental state examination (MMSE) Questionnaire
- Dobumatine stress echocardiogram ejection fraction
- DEXA Scan

8.1 Safety Analysis

The following section describes the safety analysis and includes all treated participants.

8.1.1 Infusion Status

Characteristics of the infusion will be described including infusion time, reasons for stopping the infusion, and whether any cardiorespiratory signs or symptoms following the infusion were noted.

8.1.2 Primary Safety Analysis: TE-SAE

The incidence of TE-SAE at one month post-infusion is the primary endpoint. TE-SAE is defined as the composite of: death, non-fatal pulmonary embolism, stroke, hospitalization for worsening dyspnea, and clinically significant laboratory test abnormalities:

- Serum chemistry: chloride, carbon dioxide, BUN, creatinine, glucose, calcium, AST/SGOT, ALT/SGPT, total bilirubin (fractionate if total >1.5 times normal), alkaline phosphatase, albumin
- Hematology (CBC): hemoglobin, hematocrit, platelets, WBC, WBC differential

8.1.3 Adverse Events and Serious Adverse Events

Adverse events (AEs) and serious adverse events (SAEs) will be classified into system organ class (SOC) and preferred term (PT) according to the Medical Dictionary for Regulatory Activities. An Adverse Event (AE) is defined as any untoward medical occurrence in a participant or clinical investigation participant temporally associated with the use of a medicinal product, whether or not considered related to the medicinal product. The occurrence does not necessarily have to have a causal relationship with this treatment. An AE can therefore be any unfavorable and unintended sign (including an abnormal laboratory finding, for example), symptom, or disease (new exacerbated) temporally associated with the use of a medicinal product, whether or not considered related to the medicinal product. A Serious Adverse Event (SAE) is any adverse experience occurring at any dose that has any of the following associations: 1) results in death, 2) is life-threatening (at risk of death at the time of the event),

3) requires inparticipant hospitalization or prolongation of existing, 4) results in disability/incapacity, or 5) is a congenital anomaly/birth defect. AEs/SAEs will be summarized starting at the time of infusion. AEs/SAEs occurring before infusion will be noted in a separate table.

8.1.4 Incidence of AEs/SAEs

The number of AEs/SAEs experienced in each treatment group will be described at 30-days, 6-months and 12-months post-infusion. The incidence of AEs and SAEs (including adverse events, possibly-related MSC adverse events, possibly-related to the catheter adverse events, and serious adverse events) will be described as the proportion of participants experiencing at least one AE or SAE, respectively, along with 95% confidence intervals by 30-days, 6-months, and 12-months post-infusion. The number and incidence of cardiac-disorders (as defined by MedDRA) will also be quantified by 30-days, 6-months, and 12-months.

8.1.5 Relationship of Adverse Events to Study Drug

Relationship of each AE is with respect to the product, device or procedure. For analysis purposes, relationship of each AE/SAE will be considered related or unrelated and described by treatment group.

8.1.6 Severity of Adverse Event

The investigator will make an assessment of intensity for each AE and SAE reported during the study. The assessment will be based on the Investigator's clinical judgment. The intensity of each AE and SAE should be assigned to one of the following categories:

Mild:	An event that is easily tolerated by the participant, causing minimal discomfort and not interfering with everyday activities.
Moderate:	An event that is sufficiently discomforting to interfere with normal everyday activities.
Severe:	An event that prevents normal everyday activities.

AE/SAE severity will be described by treatment group.

8.1.7 Death

All participant deaths during the study, including post-treatment follow-up period, and deaths that resulted from a process that began during the study will be listed by participant similarly to the collection of AEs. The number of days on study and primary cause of death will be included in the data listing. Survival will be estimated using the Kaplan-Meier method if the number of events supports such an analysis.

8.1.8 Hospitalizations

All hospitalizations will be listed by participant. The incidence of all-cause hospitalizations and worsening dyspnea hospitalizations will be described at 30-days, 6-months, and 12-months post-infusion. Point estimates and 95% confidence intervals will be described for each treatment arm, if the number of events permits such an analysis. The probability of hospitalization-free survival and worsening dyspnea hospitalization-free survival will be estimated using the Kaplan-Meier method if the number of events permits. The time to first all-cause hospitalization and

worsening dyspnea hospitalization will be estimated using cumulative incidences treating death as a competing risk and censoring at the last date of follow-up.

8.1.9 Non-fatal pulmonary embolism

The incidence of non-fatal pulmonary embolisms will be described at 30-days, 6-months, and 12-months post-infusion. Point estimates and 95% confidence intervals will be described for each treatment arm. The probability of experiencing a non-fatal pulmonary embolism will be estimated using the Kaplan-Meier method if the number of events permits. The time to first non-fatal pulmonary embolism will be estimated using cumulative incidences treating death as a competing risk and censoring at the last date of follow-up.

8.1.10 Stroke

The incidence of strokes will be described at 30-days, 6-months, and 12-months post-infusion. Point estimates and 95% confidence intervals will be described for each treatment arm. The probability of experiencing a stroke will be estimated using the Kaplan-Meier method if the number of events permits. The time to first stroke will be estimated using cumulative incidences treating death as a competing risk and censoring at the last date of follow-up.

8.1.11 Clinical Laboratory Evaluations

Clinical laboratory evaluations will be displayed using spaghetti plots and listings by treatment group. Abnormal values will be clearly identified. Mean or median values will be included in the spaghetti plots. Analyses will also be displayed as changes over time and individual participant changes via shift tables. Upper limit of normal (ULN) will be provided for each laboratory evaluation.

8.1.12 Hematology

Hematology parameters are analyzed at each time point that the participant is seen and will be described by treatment group.

8.1.13 Serum Chemistry

Serum chemistry parameters are analyzed at each time point that the participant is seen and will be described by treatment.

8.1.14 Urinalysis

Urinalysis parameters are analyzed at each time point that the participant is seen and will be described by treatment group.

8.1.15 Protein Reactive Antibodies

The proportion of participants who had a significant increase in their protein reactive antibodies will be described.

8.1.16 Inflammatory Markers

The following inflammatory markers will be assessed:

1. C-reactive protein, CRP
2. Interleukin-6, IL-6
3. D-dimer
4. Fibrinogen
5. CBC with differential
6. DNA
7. Tumor necrosis factor-alpha, TNF-alpha.

8.1.17 12-Lead Electrocardiogram

A 12-lead electrocardiogram is performed at screening, baseline, day 1, week 2, 1 month, 3 months, and 6 months. The incidence of sinus bradycardia, sinus tachycardia, conduction block, and atrial arrhythmia will be described for each dose level.

8.2 Efficacy Analysis

Efficacy analyses will be conducted to determine whether MSCs change efficacy parameters over a 6 month time interval. Within treatment groups will be assessed using point estimates and 95% confidence intervals. If data are heavily skewed, medians and ranges will be presented in lieu of means and 95% confidence intervals. Within and between dose level analyses are considered exploratory. A mixed model for repeated measures will be used to compare treatment groups when appropriate.

8.2.1 Difference in rate of decline of frailty

Difference in rate of decline of frailty is defined by the following parameters:

- Reduced activity (assessed via CHAMPS Questionnaire)
- Slowing of mobility (assessed via a 4 meter gait speed test and SPPB)
- Weight loss
- Diminished handgrip strength (assessed via dynamometer)

8.2.1.1 CHAMPS Activities Questionnaire for Older Adults

Results of the CHAMPS questionnaire will be summarized into four components:

- Caloric expenditure per week in all exercise-related activities
- Caloric expenditure per week in moderate-intensity exercise-related activities
- Frequency per week of all exercise-related activities
- Frequency per week of moderate-intensity exercise-related activities

Estimates of the each component and 95% confidence intervals will be presented at each time point for all three treatment groups. Change in each component will be assessed over time comparing the 3- and 6-month results to baseline for all three treatment groups. A repeated measures analysis will be conducted to compare within and between treatment groups.

8.2.1.2 4m Gait Speed Test

Estimates of the 4-meter gait speed test and 95% confidence intervals will be presented at each time point for all three treatment groups. The change from baseline at 3 and 6 months will be presented along with 95% confidence intervals. A repeated measures analysis will be conducted to compare within and between treatment groups.

8.2.1.3 Short Physical Performance Battery (SPPB)

Results from SPPB assessments will be tabulated by treatment group at each time point.

8.2.1.4 Recipient Weight

Estimates of the recipient's weight and 95% confidence intervals will be presented at each time point for all three treatment groups. Change in weight from baseline will be estimated at each follow-up visit along with 95% confidence intervals for all three treatment groups. A repeated measures analysis will be conducted to compare within and between treatment groups.

8.2.1.5 Handgrip Strength Test

Estimates of the handgrip strength and 95% confidence intervals will be presented at each time point for all three treatment groups. Change in handgrip strength will be assessed over time comparing the 3- and 6-month results to baseline for all three treatment groups. A higher handgrip strength expressed in pounds is considered better. A repeated measures analysis will be conducted to compare within and between treatment groups. The following analyses will be performed:

- Describe the average of the two scores for the right hand over time
- Describe the average of the two scores for the left hand over time
- Describe the average of the two scores for the dominant hand over time
- Describe the average of the two scores for the non-dominant hand over time

8.2.1.6 FEV1

Estimates of FEV1 expressed in liters and percent predicted and 95% confidence intervals will be presented at each time point for all three treatment groups. The change from baseline at 3 and 6 months will be presented along with 95% confidence intervals for all three treatment groups. A repeated measures analysis will be conducted to compare within and between treatment groups.

8.2.1.7 Six minute walk test (6MWT)

Estimates of the six minute walk test and 95% confidence intervals will be presented at each time point for all three treatment groups. The change from baseline at 3 and 6 months will be presented along with 95% confidence intervals for all three treatment groups. A repeated measures analysis will be conducted to compare within and between treatment groups.

8.2.1.8 Smell Identification Test

Estimates of the smell identification test and 95% confidence intervals will be presented at each time point for all three treatment groups. The change from baseline and 6 months will be presented along with 95% confidence intervals for all three treatment groups. A repeated measures analysis will be conducted to compare within and between treatment groups.

8.2.2 ICECAP-O Questionnaire

Results of the ICECAP-O questionnaire will be summarized by summing the 5 components for a total score of 20. A higher total score on the ICECAP-O questionnaire indicates a more favorable quality of life for the recipient. Estimates of the ICECAP-O score and 95% confidence intervals will be presented at each time point for all three treatment groups. Change in ICECAP-O score will be assessed over time comparing the 3- and 6-month results to baseline for all three treatment groups. A repeated measures analysis will be conducted to compare within and between treatment groups.

8.2.3 EQ-5D

The EQ-5D questionnaire is ascertained at baseline, 1-month, 3-months and 6-months post-infusion. There are two components to the EQ-5D questionnaire as described below.

8.2.3.1 EQ-5D Score

Results of the EQ-5D questionnaire will be summarized by summing the 5 components for a total score of 15. A lower total score on the EQ-5D questionnaire indicates a more favorable quality of life for the recipient. Estimates of the EQ-5D score and 95% confidence intervals will be presented at each time point for all three treatment groups. Change in EQ-5D score will be assessed over time comparing the 1-, 3- and 6-month results to baseline for all three treatment groups. A repeated measures analysis will be conducted to compare within and between treatment groups.

8.2.3.2 EQ-5D Scale

The EQ-5D scale will be described. A higher scale on the EQ-5D scale indicates a more favorable quality of life for the recipient. Estimates of the EQ-5D scale and 95% confidence intervals will be presented at each time point for all three treatment groups. Change in EQ-5D scale will be assessed over time comparing the 1-, 3- and 6-month results to baseline for all three treatment groups. A repeated measures analysis will be conducted to compare within and between treatment groups.

8.2.4 SF-36

The SF-35 version 2.0 was measured at baseline 1-, 3- and 6-months post-infusion. Estimates of the three main components of the SF-26 and 95% confidence intervals will be presented at each time point for all three treatment groups. Change in each component of the SF-35 will be assessed over time comparing the 1-, 3- and 6-month results to baseline for all three treatment groups. A repeated measures analysis will be conducted to compare within and between treatment groups. The three components are as follows:

- Physical Component Summary
- Mental Component Summary

8.2.5 Mini Mental State Examination (MMSE) Questionnaire

Results of the MMSE questionnaire will be summarized by summing all the components for a total score of 30. A higher total score on the MMSE questionnaire indicates a more favorable

quality of life for the recipient. Estimates of the MMSE score and 95% confidence intervals will be presented at each time point for all three treatment groups. Change in MMSE score will be assessed over time comparing the 1-, 3- and 6-month results to baseline for all three treatment groups. A repeated measures analysis will be conducted to compare within and between treatment groups.

8.2.6 Multidimensional Fatigue Inventory (MFI) Questionnaire

Results of the MFI questionnaire will be summarized for each of the five dimensions of fatigue:

- General fatigue
- Physical fatigue
- Reduced motivation
- Reduced activity
- Mental fatigue

Higher total scores correspond with more acute levels of fatigue (Smets et al). Estimates of the MFI score and 95% confidence intervals will be presented at each time point for all three treatment groups. Change in MFI score will be assessed over time comparing the 1-, 3- and 6-month results to baseline for all three treatment groups. A repeated measures analysis will be conducted to compare within and between treatment groups.

8.2.7 Dobutamine Stress Echocardiogram Ejection Fraction

Dose dependent ejection fraction will be compared between baseline and 6-months post-infusion. A repeated measures model will look at within group changes in ejection fraction with dose as the repeated measure. Change in ejection fraction at rest and various doses of dobutamine will be compared between screening and at 6 months post-infusion. Estimates and 95% confidence intervals will be presented for each dose level of resting ejection fraction, ejection fraction at 5, 10, 20, and 30 mcg/kg/min.

8.2.8 DXA Scan

Results of the DXA scan will be presented at baseline and 6-months post-infusion among the following measurements:

- Total Body Bone Mineral Density (BMD) – the average of all the respective BMD measurements of the total body.
- Femur Neck of hip – the BMD of the femur of the neck of the hip
- Total hip BMD – the average BMD of all the hip/femur bones.
- L1-L4 spine BMD – the BMD of lumbar vertebrae (L1-L4)
- T-score – how many standard deviations a patient’s result is above or below the mean of that age group.

Change from baseline in each measurement will be assessed for all three treatment groups. Treatment groups will be compared using ANCOVA.
